# DP4-AI automated NMR data analysis: straight from spectrometer to structure†

**DOI:** 10.1039/d0sc00442a

**Published:** 2020-03-06

**Authors:** Alexander Howarth, Kristaps Ermanis, Jonathan M. Goodman

**Affiliations:** Centre for Molecular Informatics, Department of Chemistry, University of Cambridge Lensfield Road Cambridge CB2 1EW UK ke291@cam.ac.uk jmg11@cam.ac.uk

## Abstract

A robust system for automatic processing and assignment of raw ^13^C and ^1^H NMR data DP4-AI has been developed and integrated into our computational organic molecule structure elucidation workflow. Starting from a molecular structure with undefined stereochemistry or other structural uncertainty, this system allows for completely automated structure elucidation. Methods for NMR peak picking using objective model selection and algorithms for matching the calculated ^13^C and ^1^H NMR shifts to peaks in noisy experimental NMR data were developed. DP4-AI achieved a 60-fold increase in processing speed, and near-elimination of the need for scientist time, when rigorously evaluated using a challenging test set of molecules. DP4-AI represents a leap forward in NMR structure elucidation and a step-change in the functionality of DP4. It enables high-throughput analyses of databases and large sets of molecules, which were previously impossible, and paves the way for the discovery of new structural information through machine learning. This new functionality has been coupled with an intuitive GUI and is available as open-source software at https://github.com/KristapsE/DP4-AI.

## Abstract 2

A robust system for automatic processing and assignment of raw ^13^C and ^1^H NMR data DP4-AI has been developed and integrated into our computational organic molecule structure elucidation workflow.

## Graphical abstract

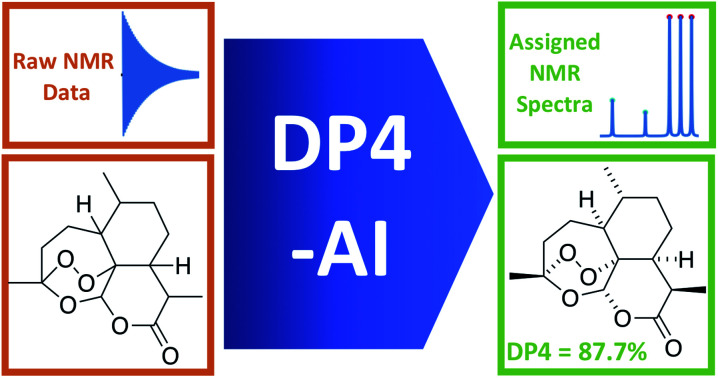

## Introduction

Structural elucidation of molecules is a challenging problem in both synthetic organic and natural product chemistry. Structural near isomers (for example regioisomers and protecting group localisation) and diastereomers usually exhibit only subtle differences in their 1D NMR spectra, making determination of structure and relative stereochemistry very difficult. This can be addressed by additional NMR experiments such as nOeSY spectra or synthesizing isomers of the natural product and comparing the resulting observed NMR spectra with those published. Both approaches are very expensive and time consuming.

An attractive and now established alternative^1,2^ is to use computational NMR prediction. This process uses DFT to calculate NMR shifts for all the diastereomers of the uncertain structure and compare these predications with the published spectra using parameters such as, correlation coefficient, mean absolute error (MAE), corrected mean absolute error (CMAE).^3^ DP4 analysis is particularly powerful as it not only predicts the relative stereochemistry and other variations of the molecule, but also using Bayes theorem gives a probability that each candidate molecule is the correct one (assuming one of the provided or generated structures is correct).^3,4^ DP4 has been successfully used to determine the stereochemistry of many natural product like molecules, synthetic intermediates, natural product fragments and also pharmaceutical compounds.^5–10^ and has been explored further in DP4+ and J-DP4 analyses.^11,12^

Since its publication, the calculation of DP4 has been streamlined and user input minimized as all calculations are now automatically managed by the Python program PyDP4.^11–13^ Only a structure of the molecule with undefined stereochemistry and assigned experimental 1D ^13^C and ^1^H NMR spectra are required as inputs from the user. The most user intensive part of relative stereochemistry elucidation using this program is now the assignment of the NMR spectra. This is not only very time consuming but also often laborious and error prone.^14^ Automated interpretation of NMR spectra has been a major goal of analytical chemistry for many years.^15^ Much of this work has been focused on developing CASE (Computer Aided Structure Elucidation) software^16–18^ for automated 2D structure determination and dereplication rather than automated assignment of atoms in a known structure. Typically a number of 2D NMR spectra in addition to the 1D NMR spectra must be provided.^19^

A small number of commercial software packages offer expert-guided NMR assignment algorithms for ^1^H NMR spectra, notably Mestrelab Mnova.^20^ This software has focused on aiding a user to interpret NMR spectra as opposed to automated processing and assignment of raw NMR data.

**Fig. 1:**
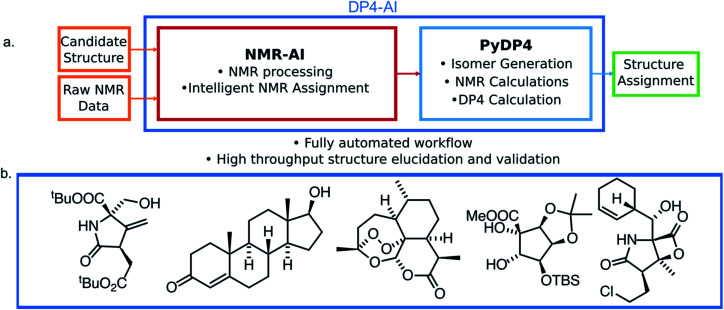
(a) The structure of DP4-AI. This system affords fully automated stereochemistry elucidation, only the raw NMR data is a required input from the user. (b) Example structures with stereochemistry correctly predicted fully automatically using DP4-AI integrated in PyDP4.

**Fig. 2:**
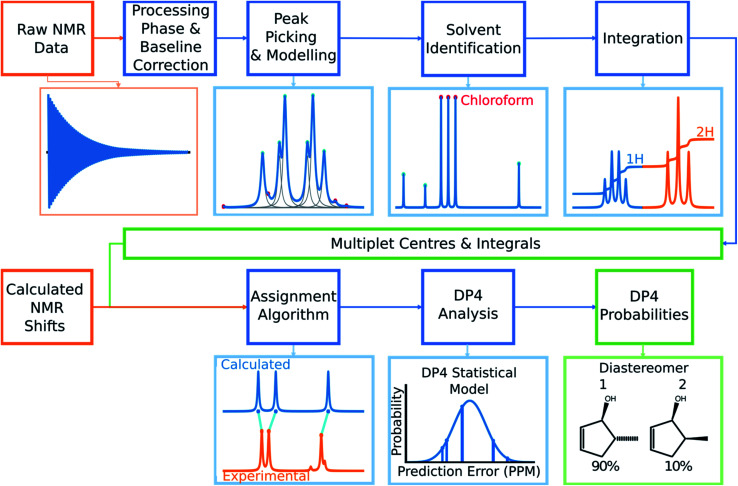
The overall structure of DP4-AI. Raw NMR data is processed in a series of stages to yield experimental multiplet shift values and their integrals. The program then takes shifts calculated using DFT for each atom in the molecule and assigns them to the experimental peaks. This assignment is then used to calculate a DP4 probability for each diastereomer.

In this work a system for fully automatic robust processing and assignment of both ^1^H and ^13^C NMR spectra is presented (Fig. 1). A schematic of this program is displayed in Fig. 2. It provides automated relative stereochemistry and structural ambiguity prediction using 1D ^1^H and ^13^C NMR spectra. Chemical shift values are calculated using the DFT GIAO method. Shift prediction by this method can also be performed using only free and open source software such as NWChem^21^ and Tinker^22^ making this method more accessible than any other software currently available.

The automation DP4-AI affords is exciting as it will allow high-throughput DP4 analysis of databases and large sets of molecules, which was previously impossible. In addition, automatic processing and assignment of NMR spectra will reduce the time constraints of synthesis, allowing for more opportunities in chemical discovery. Moreover, this system will also provide a framework for the development of automated interpretation of more complex NMR experiments in the near future and could be used in conjunction with CASE software to solve structural elucidation problems from analytical data.

## Computational methods

The calculation of DP4 was performed following previous works.^3,11–13^ All molecular mechanics calculations were performed using MacroModel (Version 9.9). All conformational searches were performed in the gas phase utilizing the MMFF force field^23–28^ and a mixture of Low Mode following and Monte Carlo search algorithms.^29,30^ The step count for MacroModel was set so that all low energy conformers were found at least 5 times.

Quantum mechanical calculations were carried out using Gaussian09. NMR shielding constants were found using the GIAO method.^31–33^ The functional mPW1PW91 (ref. 34) was chosen with the 6-311G(d) basis set^35^ for NMR shift prediction as this has been shown to be optimal for DP4 calculation.^12^ For molecules containing iodine, the basis set def2-SVP^36,37^ was chosen. All DFT calculations were performed using the implicit PCM solvent model.^38^ The molecular geometries were also optimized at the DFT level of theory, this was performed using the B3LYP functional^39,40^ with the 6-31G(d) basis set. Finally, single-point energies were separately calculated using M06-2X functional and def2-TZVP basis set.

The calculations were managed by the PyDP4 Python script written in Python 3.7 which is now part of DP4-AI. DP4-AI is available from http://www-jmg.ch.cam.ac.uk/tools/nmr/ and GitHub https://github.com/KristapsE/DP4-AI/. Some elements of NMR processing was performed using the package NMRglue.^41^

## Program description

Automated NMR processing would remove the need for the user to laboriously write an NMR description, radically increasing the productivity of the process. In order to assign atoms in a molecule to peaks in an NMR spectrum, peak locations and integral values must be extracted from the raw NMR data as shown in Fig. 2. Fully automated processing and analysis of NMR data is a complex problem as all NMR spectra are different and each stage in the processing can affect subsequent stages. DP4-AI has been designed to process NMR data as robustly and reliably as possible in spite of these challenges. An overview of this section of the program is given below, a more detailed description is given in the ESI (Section S2.1†).

After performing a Fourier transform the spectrum may display phasing errors which must be corrected prior to further processing. Unfortunately, none of the existing methods phased the test set of spectra as reliably as required. To alleviate this issue, a hybrid method combining, the signal classification method developed by Wang *et al.*,^42^ the entropy based objective function of the phasing algorithm ACME^43^ and the robust framework of weighted linear regression approach (WLR) developed by Zorin *et al.*^44^ was employed.

**Fig. 3:**
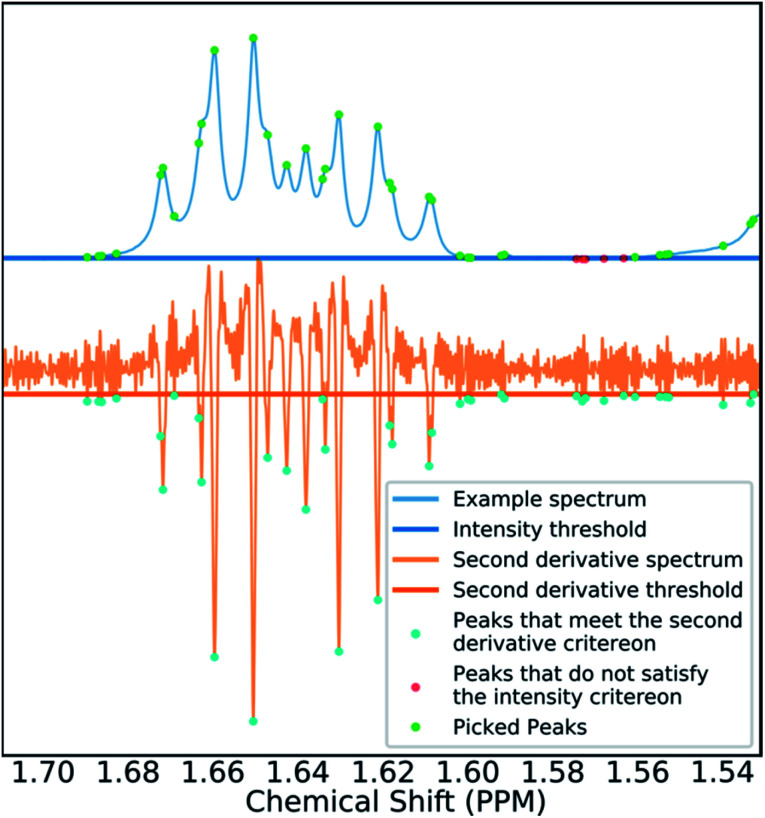
Figure illustrating the gradient peak picking process. Peaks are picked if they are below a threshold in the second derivative (orange) and above an intensity threshold (blue). The final picked peaks are highlighted in green.

Many spectra also display baseline distortions which must be removed. A modified version of the algorithm developed by Wang *et al.*^42^ was incorporated into the final program (ESI Section S2.1.4†). For ^1^H spectra, initially peak picking is performed using first and second derivatives of the spectrum. Potential peaks are found as points that are simultaneously zero in the first derivative and minima in the second derivative. These candidate peaks are picked if they are both, above an amplitude threshold and below a second threshold in the second derivative. These threshold values are adaptive as they are set to multiples of the noise standard deviation values. Peak-picking in this manner allows both threshold values to be set very low, screening out as much noise as possible whilst missing as few signals as possible. In addition, the use of derivative ensures baseline independence. This process is summarized in Fig. 3.

In ^1^H spectra signal peaks must be grouped together to establish where the multiplet centers are located. The maximum coupling constant expected to be seen between protons in ^1^H spectra is around 18 Hz. Any peaks <18 Hz apart can be grouped together as multiplets. To avoid missing any signal peaks, the peak picking threshold for signal to noise ratio is deliberately set very low. However, this increases the probability of noise peaks being mistaken for signal peaks and can cause over grouping of peaks (ESI Section S2.1.6†).

**Fig. 4:**
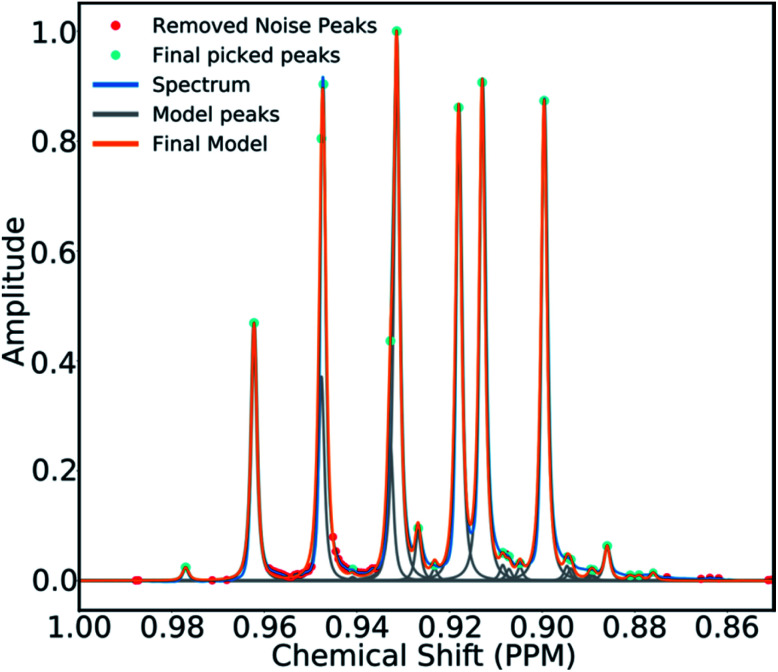
An example multiplet (blue) and deconvolved model (orange). The signal peaks are highlighted in cyan, the peaks determined to be noise are highlighted in red.

To mitigate this issue an algorithm for removing noise utilizing objective model selection was developed. Picked peaks separated by less than 18 Hz are grouped together to define signal containing regions. For each region a line shape model is constructed with multiple generalized Lorentzian line shape functions.^45^ The parameters in the model of each region are varied iteratively until the integral of the model converges to within 1% of the corresponding region of the spectrum. To avoid overfitting, the groups of parameters describing each peak are then tested for their information content. A new model is constructed without each line shape function in turn. If the Bayesian Information Criterion (ESI Section S2.1.6†) of the model is lowered by more than a threshold value, these parameters are assumed to describe a noise peak (as they do not increase the information content of the model) and are deleted. Once all of the peaks have been tested, the remaining signals are regrouped to produce the final multiplets.^46^ An example of this modelling process is displayed in Fig. 4.

Using this modelling process, solvent peaks and other contaminants can also be selectively removed. The solvent used is defined by the user to adjust DFT solvent model. To identify the solvent multiplet in the experimental data, each peak in the region of the spectrum expected to contain the solvent is given a score. This score takes into account how closely the pattern of peak locations and amplitudes around each peak match that of the expected solvent multiplet and also the distance from the expected solvent location. The peaks that most closely match those of the simulated solvent multiplet are removed from the model and the spectrum is referenced (see ESI Section S2.1.9†).

Finally, the multiplets in ^1^H spectra must be integrated. Due to the 100% abundance of the ^1^H isotope of hydrogen the integrals of multiplets in the spectra are proportional to the number of protons in each chemical environment. If this constant of proportionality can be estimated, the assignment algorithm (AA) can be told explicitly how many protons can be assigned to each multiplet.

The algorithm for estimating this constant of proportionality for ^1^H spectra incorporated into the program has been developed from previous work in this area.^20^ The premise of this algorithm is to iterate this constant *k* from the minimum possible number of protons in the spectrum (the number of protons in the structure minus the number of labile protons) to a maximum value (which has been set to twice the total number of protons in the ambiguous structure) and calculate a score based on how integer like the corresponding set of integrals are (the integrals of the multiplets are calculated using the model spectrum as described by Schoenberger *et al.*^45^). The value of *k* producing the highest score is taken as the constant of proportionality, and is used to normalize the integrals (ESI Section S2.1.10†). This scoring method is particularly advantageous as it accounts for deviations from integer integral values that are often observed due to, the choice of shimming parameters or incomplete relaxation for example. Peak-picking of ^13^C spectra is performed using a similar algorithm. The most intense peak in the spectrum is picked and simple Lorentzian function is fitted to it to create an initial model, this is repeated for the next most intense peak. This process continues until all the unpicked peaks fall within three times the standard deviation of the noise of the fitted model. This algorithm has been chosen as it effectively discards noise peaks whilst identifying low intensity signal peaks such as quaternary carbons.

## Assignment algorithm

The final challenge in the development of DP4-AI is the assignment algorithm (AA) which assigns the atoms in each diastereomer of the molecule to observed peaks in the spectra. This assignment is made using the GIAO predicted shifts.

**Fig. 5:**
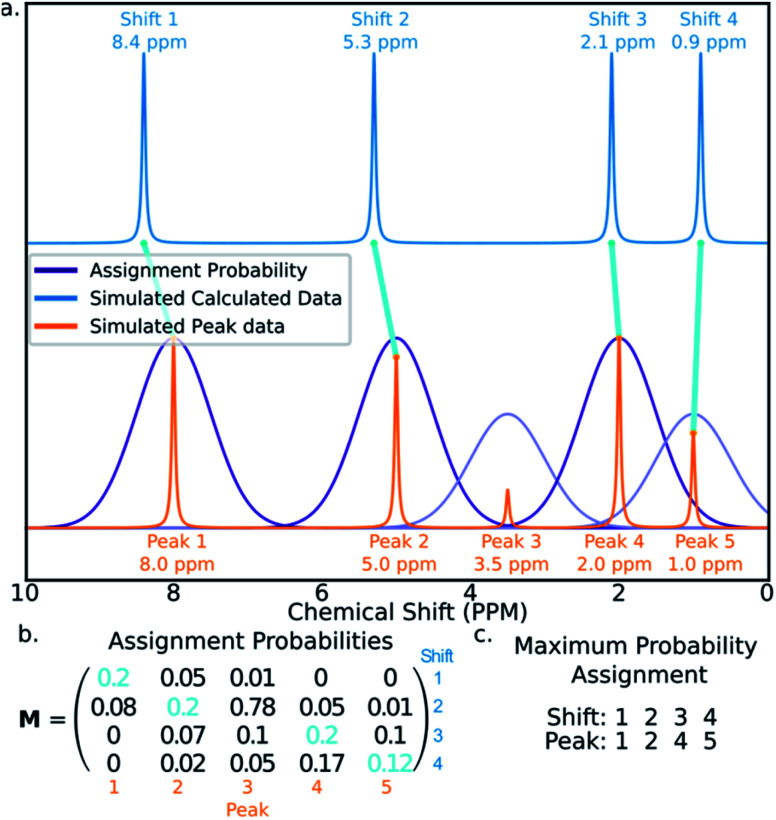
Figure illustrating how calculated shifts can be assigned to experimental peaks using the assignment probability matrix ***M***. (a) The peaks in the simulated calculated spectrum (blue) are assigned to those in the experimental spectrum (orange). (b) The matrix ***M*** is calculated and the optimum assignment (cyan) calculated. (c) The final assignment found in this example.

The core of the AA calculates the assignment probability matrix ***M***. The elements of this matrix *M*_*ij*_ give the probability of calculated shift *i* corresponding to experimental peak *j*. The matrix ***M*** is used to find the most probable assignment by the Hungarian linear sum minimization^47^ method as shown in Fig. 5.

The value ***M*** is calculated using a statistical model (ESI Section S2.2†) that takes into account the distribution of DFT prediction errors observed for the chosen computational conditions and, in the case of ^13^C NMR, also the amplitudes of the experimental peaks.

GIAO shift predictions are subject to systematic errors that vary depending on position within the spectrum and the computational conditions.^12,48^ These systematic errors must be corrected prior to calculation of ***M***. Classical DP4 alleviates this problem by performing an internal scaling process.^3^ It is not possible to use this method in this program as the assignments are unknown.

To mitigate this issue, the assignment process is performed in three stages. In the first round of assignment, prior to calculation of ***M*** a linear scaling is performed using known external scaling factors (ESI Section S2.2.1†). After the first assignment has been completed, the assigned shifts and peaks are used to calculate internal linear scaling factors in a similar fashion to DP4. The calculated shifts are then rescaled and the assignment repeated.

In ^13^C the number of experimental peaks may not be equal to the number of carbon atoms in the molecule. The GIAO shift predictions may also not reflect the degeneracy seen in the spectrum. The ^13^C is provided with additional flexibility to assign peaks in the spectrum multiple times, using a penalty system given by eqn (1).1
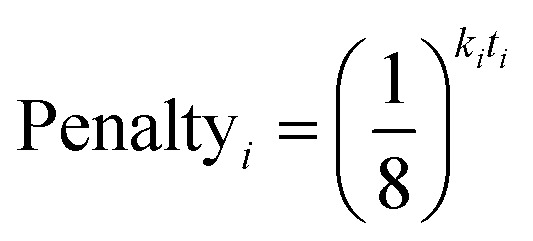


The multiple assignment penalty for experimental peak *i k*_*i*_ depends on the amplitude KDE group peak *i* is in. A value of *k* = 1 is given to the group containing the most intense peaks, then *k* = 2 to the group with the second most intense peak *etc.* The value of *t* represents the number of times the peak has already been assigned.2
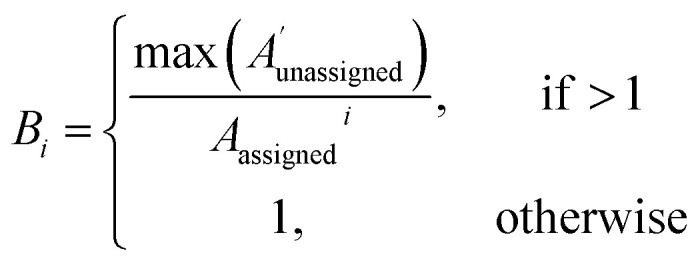


The bias for shift *i* is given above. Where 
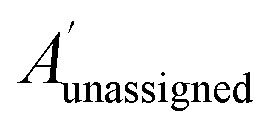
 is a vector containing the amplitudes of all unassigned peaks within ±10 ppm of the peak assigned to calculated shift *i* and *A*_unassigned_^*i*^ is the amplitude weight of the peak assigned to calculated shift *i*

**Fig. 6:**
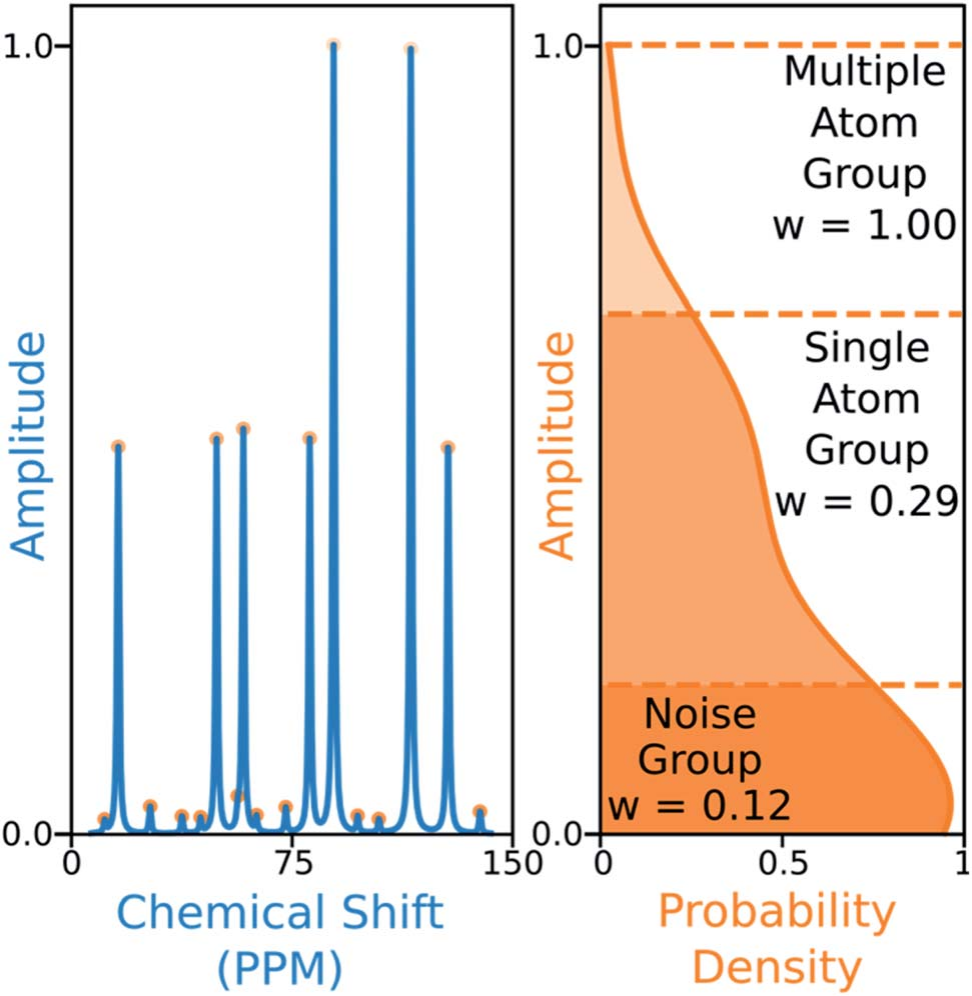
Peaks (left) are grouped by amplitude, depending on the minima in the second derivative of the amplitude probability density function (right) they fall between (dashed lines). In this simulated example, the number of carbon atoms in the structure is nine. The cumulative sum of peaks above each groups lower boundary is calculated, the weight assigned to each group is the number of carbon atoms in the structure divided by this value. The weights are then normalized to fix the largest weight to one.

The ^13^C algorithm also takes into account the amplitudes of experimental peaks. Each element of ***M***, *M*_*ij*_ is multiplied by a weight *A*_*j*_ derived from the amplitude of experimental peak *j*. This has been incorporated to prioritise the assignment of more intense peaks over those more likely to be noise. The peaks in ^13^C spectra typically fall into three groups which can be distinguished by amplitude: noise, 1-atom signals and signals corresponding to multiple equivalent carbon atoms. In order to capture this variation the probability density function of peak amplitudes in the spectra is estimated,^49^ the peaks are grouped by which minima in the second derivative of this function their amplitudes fall between. The amplitude weights are then calculated using the number of peaks in each group and the expected number of carbon atoms in the structure as shown in Fig. 6.

The ^13^C AA is also able to bias the assignment towards position or amplitude information (ESI Section S2.2.2†) by considering the distribution of peak intensities and positions in the local environment around each calculated shift. After the second round of assignment, the unassigned peaks within 10 ppm of the experimental peak assigned to each calculated shift are analyzed. The bias for calculated shift *i* is given by eqn (2). Any shifts with biases above a value of one are reassigned in order of bias to unassigned experimental peaks within 10 ppm in order of amplitude.

The role of the bias is to assess whether any signal peaks have been missed during the initial assignment. This is particularly useful in spectra where a large amount of noise has been carried through, as the AA typically favors assigning close noise peaks rather than more distant intense signal peaks in the first pass.

In contrast the ^1^H AA does not require amplitude weighting, biasing or the multiple assignment penalty as this AA can be told explicitly how many times each peak may be assigned using the integral information. The ^1^H AA also has an additional stage for the assignment of methyl protons. Protons in methyl groups consistently appear as equivalent in ^1^H NMR spectra and hence should be assigned to the same peak. The ^1^H AA assigns these protons in groups to peaks with sufficient integrals prior to the assignment of the remaining protons.

## Graphical user interface

DP4-AI may be run either from the command line to afford a fully automated workflow, or from the accompanying GUI. The GUI allows the user to easily calculate DP4 probabilities, visualize the assignments made by DP4-AI and investigate the populated conformers and prediction errors.

## Results

**Fig. 7:**
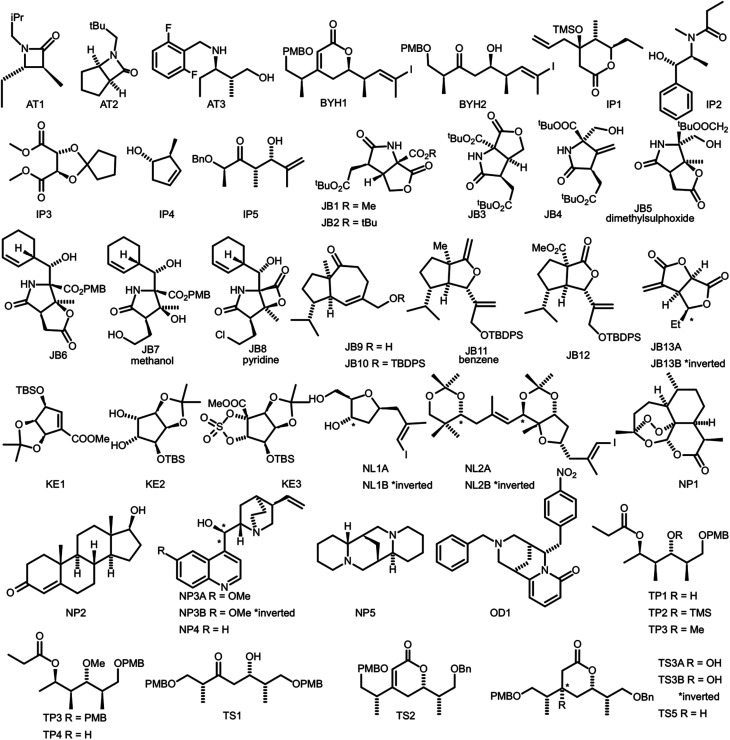
Figure illustrating the 47 molecules utilized to evaluate the performance of DP4-AI. Molecules, AT3, TS3A, TS4 and NL1A have only have corresponding ^1^H NMR data, all other molecules have both ^1^H and ^13^C NMR data. The spectra for molecules JB7, JB11, JB5 and JB8 were taken in solvents methanol, benzene, DMSO and methanol respectively, whilst all others were taken in CDCl_3_. Sources for the spectral data: AT1-3,^50,51^ BYH1-2,^52^ JB1-13B,^53,54^ TP1-4 (personal correspondence), TS1-4 (personal correspondence), OD1 (personal correspondence).

In order to evaluate the performance of NMR-AI a test set of 47 molecules (with an average of 3.49 stereocentres per molecule) with a diverse range of carbon skeletons was constructed (Fig. 7).^50–55^ This test set has been designed to include natural products, synthetic intermediates and natural product fragments to represent a wide cross section of potential use cases for DP4-AI. These molecules display challenging properties for both the AA and DP4. Previous work^12,13^ has demonstrated that flexible structures, particularly five-membered rings, and well-separated stereocentres make spectral interpretation difficult. All of these molecules are expected to present significant challenges to DP4-AI. A dataset of smaller, rigid molecules would have been much more straightforward to analyse. The corresponding spectra have also been determined in a range of solvents, some display very low signal to noise ratio and some contain mixtures of compounds. The use of this test set represents a demanding test of the performance of DP4-AI.

To predict the relative stereochemistry of a molecule in the current release of DP4, the user must provide an NMR description. The minimum amount of information required in the NMR description is, the experimental peak locations and either a description of which atoms in the molecule are chemically equivalent or the number of times each peak can be assigned. With this information DP4 assigns the atoms in the molecule in order of chemical shift to the peaks in the NMR description. We call this approach “the pairwise AA” and it is used as the benchmark for comparison with DP4-AI.

The pairwise AA was performed for all the molecules in the test set. This was very hard work, as it required manual analysis of all of the NMR spectra in order to break the signal into individual peaks and their multiplicities. This is the most time-consuming part of classical DP4, and also has the potential for subjectivity and the introduction of errors. DP4 probabilities were calculated using three different sets of computational conditions. The first level of theory tested was MM derived geometries with GIAO shift predictions utilizing the mPW1PW91 functional, 6-311G(d) basis set (def2-SVP was used for molecules containing iodine) and PCM solvent model as recommend in previous work.^11^ DP4 calculations were also performed after optimizing the geometries at the DFT level using the B3LYP functional prior to GIAO NMR shift predictions. The highest level of theory tested utilized the same DFT optimized geometries, with single point energies calculated using the M06-2X functional and def2-TZVP basis set.

DP4 also requires a statistical model describing the NMR shift prediction error probabilities. As the prediction error distribution is expected to change with computational conditions, a different model is required for each set of conditions used. Four different statistical models were tested (ESI Section S3.1†), it was found that the highest performance was obtained utilizing a single region 3 Gaussian model fitted to an empirical prediction error distribution derived from the test set. As this statistical model was constructed using the molecules in the test set and also used to calculate DP4 probabilities for the same test set, a cross validation study was also completed to assess if any overfitting was occurring. This cross-validation study was performed in a leave-one-class-out fashion for each group of molecules denoted in Fig. 7 by their initials.

**Fig. 8:**
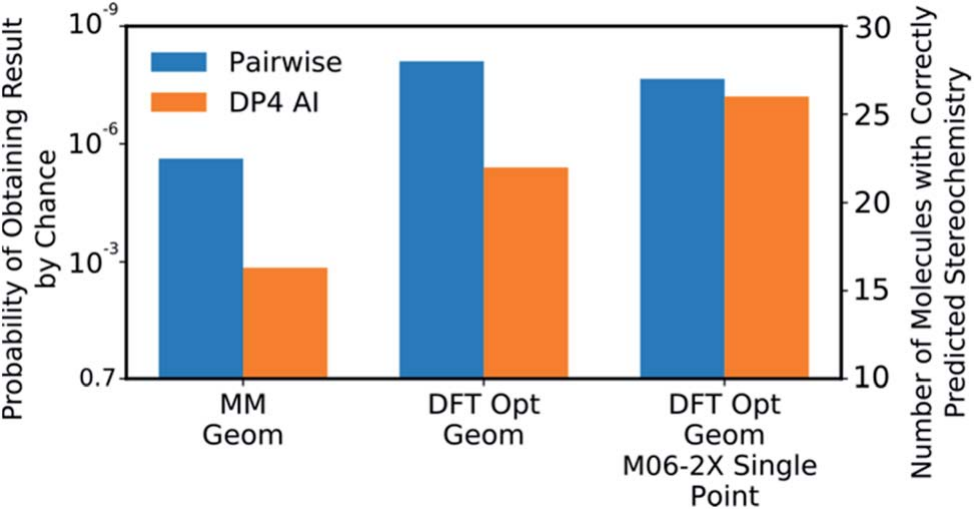
The correct prediction rates for DP4-AI (orange) and the pairwise AA (blue) at the three levels of theory tested for the compounds in Fig. 7 (average number of stereocentres equal to 3.49). These predictions were produced using the fitted 3 Gaussian cross validated statistical model.

DP4-AI was tested at all three levels of theory described with each statistical model (ESI Section S3†). A comparison of DP4-AI and the pairwise AA for the highest level of theory and most reliable statistical model is presented in Fig. 8.

## Discussion

DP4-AI, at the highest level of theory tested, interprets spectra with a similar reliability to the traditional, labour intensive, pairwise AA, which requires a highly-trained chemist to pre-process the spectra (Fig. 8). This is an impressive result given the challenging nature of the dataset. The probability of correctly assigning the stereochemistry this effectively in this data set is about 3 × 10^−8^, indicating DP4-AI is very reliably performing better than chance (ESI Section S3†). Most impressively DP4-AI correctly assigned the relative stereochemistry of molecules NP1 and NP2 out of the 32 and 64 diastereomers. The pairwise AA represents the upper limit of DP4-AIs performance in this study as the NMR descriptions used by the pairwise AA have been meticulously written to remove any errors. In reality errors are often incorporated into NMR descriptions and assignments, in these cases it would be possible for NMR-AI to outperform the pairwise AA.

The performance of DP4-AI, relative to pairwise AA, increases with the level of theory (Fig. 8). As in previous work^13^ shows that as the level of theory is increased in the DP4 calculation, the correct prediction rate of the pairwise AA also increases. DP4-AI shows a greater sensitivity to the level of theory. This is because both the assignment and the DP4 calculation are dependent on the accuracy of the NMR shift calculations. Therefore, it can be concluded that when using DP4-AI, the conditions that produce the most accurate shift predictions should always be used.

**Fig. 9:**
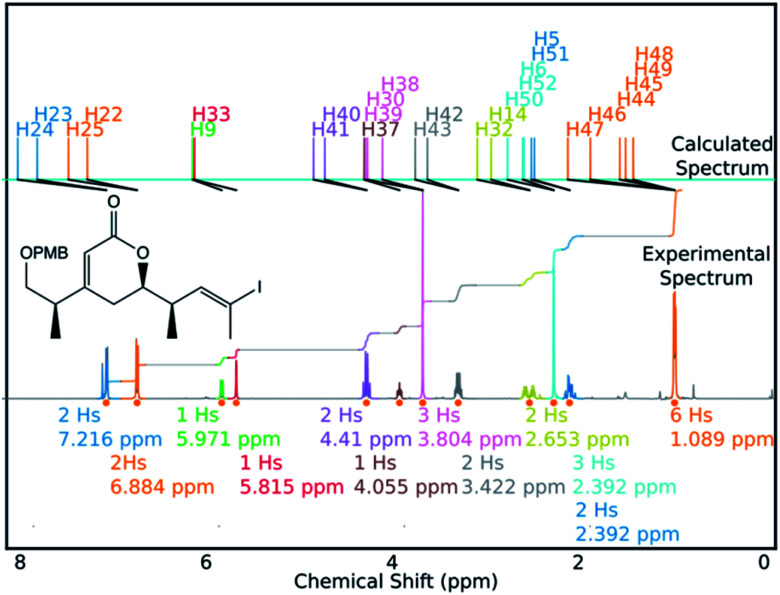
DP4-AI processed and assigned ^1^H spectrum of molecule BYH1 (taken in chloroform).

DP4-AIs performance could be improved even further by robustly addressing some of the remaining challenges in the GIAO NMR prediction, including conformational flexibility, specific solvent interactions and the presence of heavy atoms. The performance may be improved further by adding explicit support for spectra containing mixtures of compounds (such as IP2 see ESI Section S3.2†). These issues will be addressed in future developments of DP4-AI. An example of a spectrum assigned by DP4-AI is given in Fig. 9 (All the processed and assigned spectra are provided in the ESI, Section S4†).

## Conclusion

**Fig. 10:**
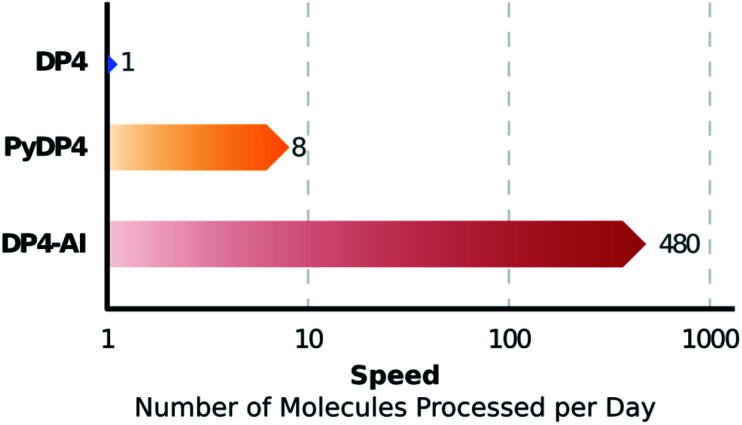
NMR-AI can process a molecule for DP4 calculation in around one minute, a task that previously would require roughly 8 hours of the users time. This corresponds to a ∼60 fold increase in the number of molecules that can be processed per day.

DP4-AI – a robust system for automatic resolution of structural uncertainty utilizing automatic processing and assignment of raw ^13^C and ^1^H NMR spectra has been developed and released as open source software. This automation will allow rapid DP4 analyses of databases and large set of molecules, which was previously impossible (Fig. 10). DP4-AI maintains the same high rate of correct structure elucidation as DP4 utilizing NMR descriptions written by an expert chemist. Moreover, this system can reliably process and assign an NMR spectrum around 60 times faster, releasing time for experimentation and discovery. In addition, this new system provides a robust framework for developing new functionality in the future such as J value analysis, 2D NMR assignment, assigning spectra of complex mixtures and aiding conformational analysis. DP4-AI is available as open source software at https://github.com/KristapsE/DP4-AI.

## Conflicts of interest

There are no conflicts to declare.

## Supplementary Material

SC-011-D0SC00442A-s001

SC-011-D0SC00442A-s002
