# Supplementary material for: DP4-AI automated NMR data analysis: straight from spectrometer to structure
Source: Chem Sci. 2020 Mar 6;11(17):4351–9. doi: 10.1039/d0sc00442a (PMC8152620; doi:10.1039/d0sc00442a)
Supplement: SC-011-D0SC00442A-s002 [file SC-011-D0SC00442A-s002.pdf]

Electronic Supplementary Material (ESI) for Chemical Science.
This journal is © The Royal Society of Chemistry 2020

DP4-AI Automated NMR Data Analysis: Straight from Spectrometer to Structure

Supporting Information

Alexander Howarth, Kristaps Ermanis*, Jonathan M. Goodman*

Centre for Molecular Informatics, Department of Chemistry, University of Cambridge, Lensfield Road, Cambridge CB2 1EW E-mail: jmg11@cam.ac.uk Phone: 01223 336434

Contents

1 Utilising DP4-AI **3**

2 Program Development **9**
2.1 NMR Processing . . . 10
2.1.1 Initial Processing . . . 10
2.1.2 Zero Filling . . . 11
2.1.3 Automatic Phase Correction . . . 12
2.1.4 Automatic Baseline Correction . . . 18
2.1.5 Gradient Peak Picking . . . 23
2.1.6 Spectral Modelling . . . 25
2.1.7 Spectral Line Shape . . . 27
2.1.8 Fitting Process . . . 31
2.1.9 Solvent Peak Identification and Spectrum Referencing . . . 33
2.1.10 Integration . . . 37
2.1.11 Peak Centre Determination . . . 39
2.2 Assignment Algorithm . . . 40
2.2.1 Prediction Error Statistical Models . . . 44
2.2.2 Amplitude Statistical Model . . . 47
2.2.3 Multiple Assignment Penalty . . . 50

3 Results and Discussion **51**
3.1 Results . . . 51
3.2 Discussion . . . 51
3.3 Full Results . . . 60

4 Assigned Spectra **93**

5 Full Carbon NMR Shift Predictions, Assignments and DP4 Probabilities DP4-AI **184**
5.1 AT1 . . . 185
5.2 AT2 . . . 186
5.3 AT3 . . . 187
5.4 BYH1 . . . 188
5.5 BYH2 . . . 189
5.6 IP1 . . . 190
5.7 IP2 . . . 191
5.8 IP3 . . . 192
5.9 IP4 . . . 193
5.10 IP5 . . . 194
5.11 JB10 . . . 195
5.12 JB11 . . . 197
5.13 JB12 . . . 199
5.14 JB13A . . . 200
5.15 JB13B . . . 201
5.16 JB1 . . . 202
5.17 JB2 . . . 203
5.18 JB3 . . . 204
5.19 JB4 . . . 205
5.20 JB5 . . . 206
5.21 JB6 . . . 207
5.22 JB7 . . . 208
5.23 JB8 . . . 209
5.24 JB9 . . . 210
5.25 KE1 . . . 211
5.26 KE2 . . . 212
5.27 KE3 . . . 213
5.28 NL1A . . . 214
5.29 NL1B . . . 215

5.30 NL2A . . . 216
5.31 NL2B . . . 217
5.32 NP1 . . . 218
5.33 NP2 . . . 219
5.34 NP3A . . . 220
5.35 NP3B . . . 221
5.36 NP4 . . . 222
5.37 NP5 . . . 223
5.38 OD1 . . . 224
5.39 TP1 . . . 225
5.40 TP2 . . . 226
5.41 TP3 . . . 228
5.42 TP4 . . . 229
5.43 TS1 . . . 230
5.44 TS2 . . . 231
5.45 TS3A . . . 232
5.46 TS3B . . . 233
5.47 TS4 . . . 234

1 Utilising DP4-AI

DP4-AI can be downloaded from https://github.com/KristapsE/DP4-AI

DP4-AI has been developed for fully automated structure elucidation. DP4-AI can be easily run standalone utilising the command line interface, or integrated into a users workflow using the source code. A single command is required to run NMR-AI for a compound. This command indicates the calculations the user wishes to perform (i.e DFT geometery optimisation, higher level DFT single point energy calculations and NMR calculations) and the desired computational conditions (default conditions will be used if none are provided). Once this command has been entered, DP4-AI will manage all of the required calculations to produce a DP4 probability with no further intervention from the user being required.

DP4-AI maybe run locally on a Desktop PC, DP4-AI also provides support for utilising external clusters.

DP4-AI can also be run utilising the provided GUI. This GUI allows users to run DP4-AI through a more familiar interface (Figure 1). It also allows the user to explore the assignments made by DP4-AI (Figures 2, 3, 4, 5), investigate the conformers (Figure 6) and prediction errors from the DP4 calculations (Figure 7).

DP4-AI has been designed to be as easy to install and use as possible. Full up-to-date instructions for, downloading, installing and using DP4-AI can be found on the Github page https://github.com/KristapsE/DP4-AI.

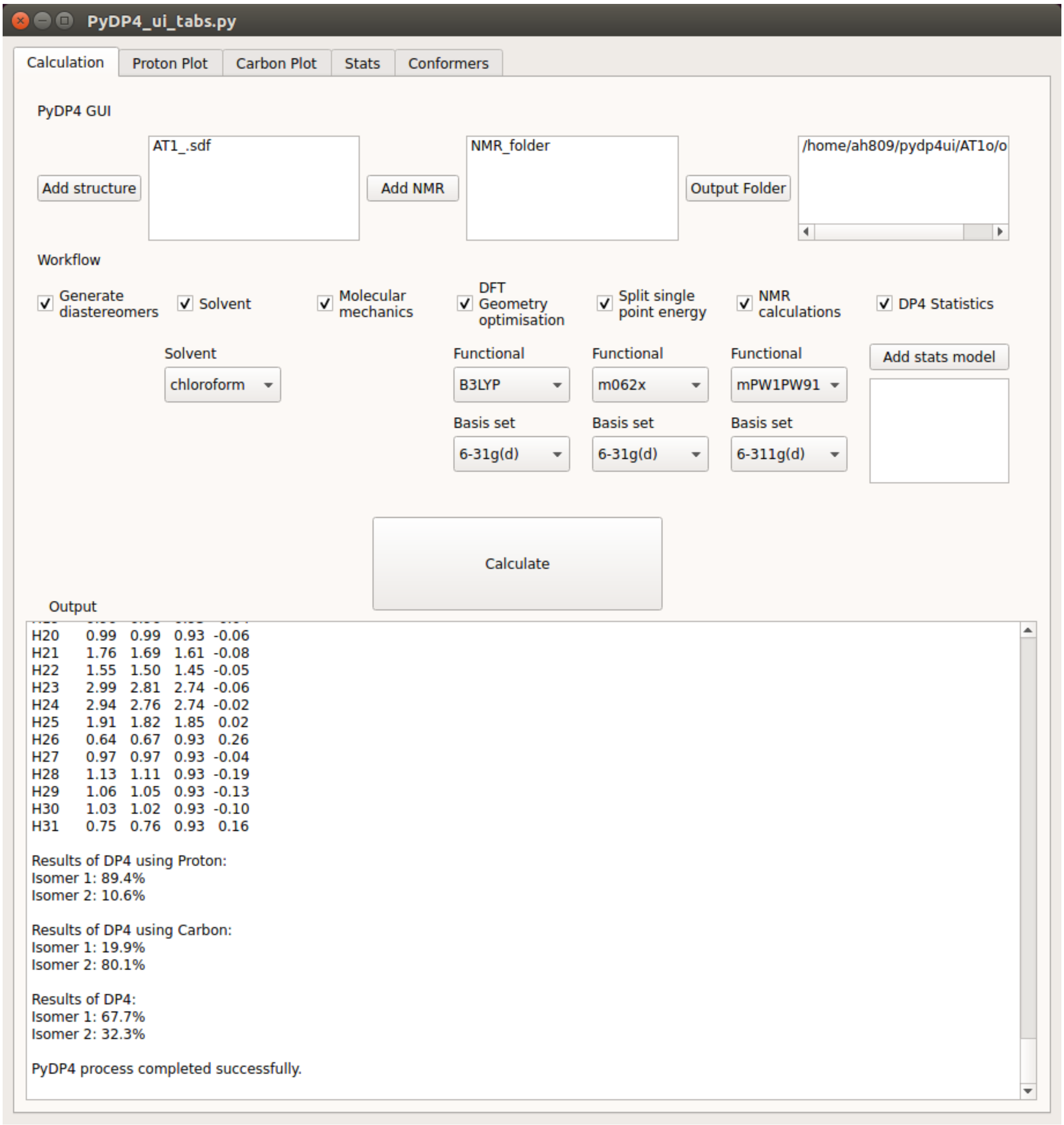

Figure 1: The GUI interface for running DP4-AI

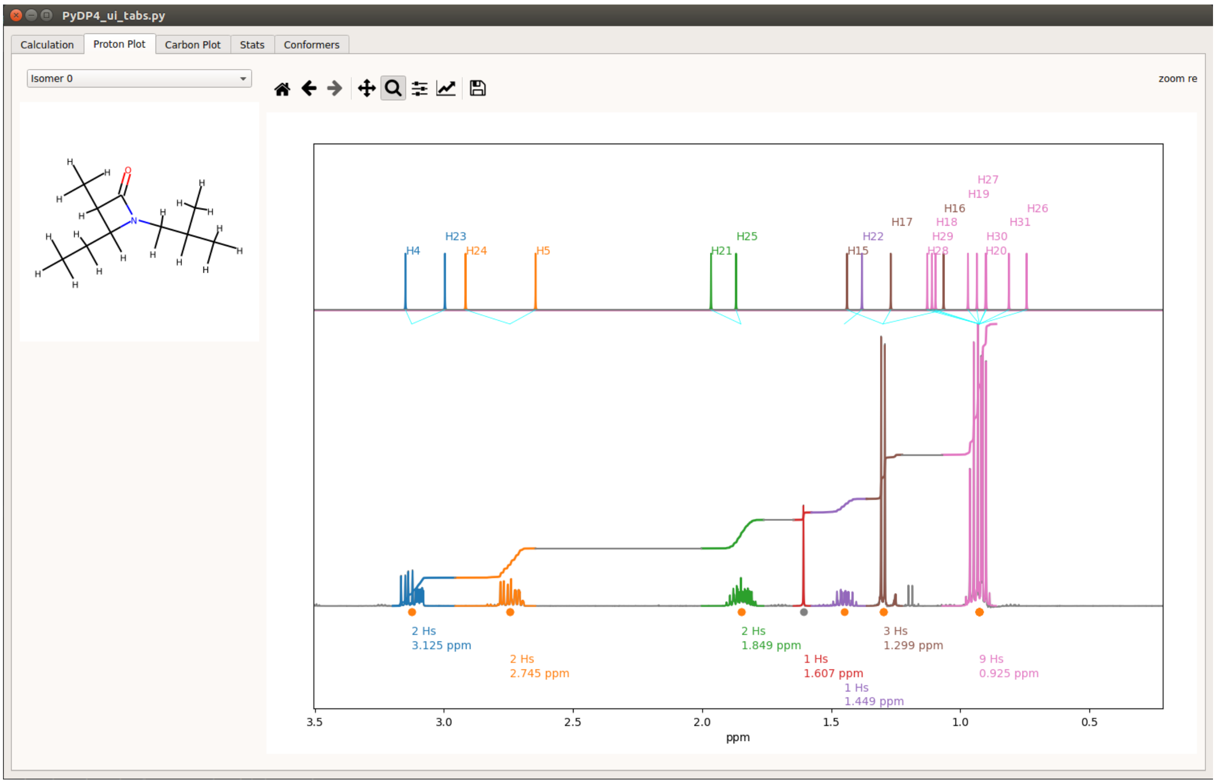

Figure 2: DP4-AIs GUI interface can be used to explore the assignments made by NMR-AI

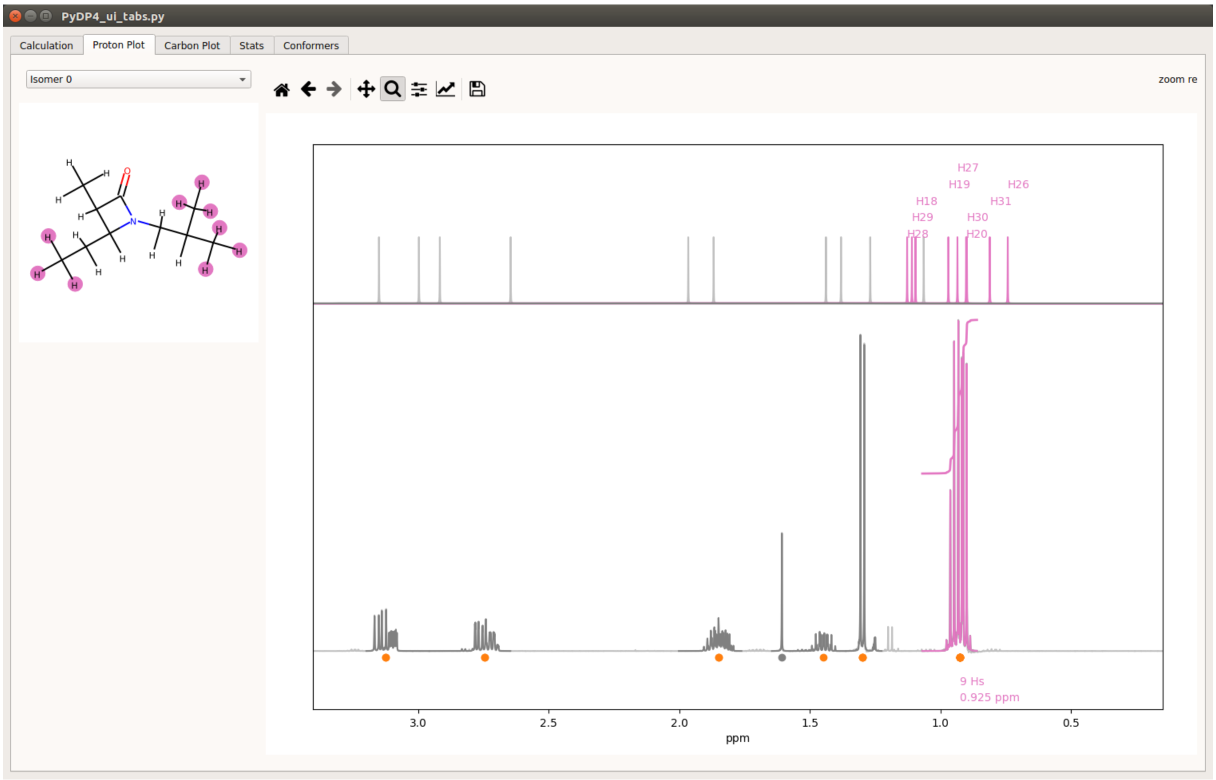

Figure 3: DP4-AIs GUI interface can be used to explore the assignments made by NMR-AI

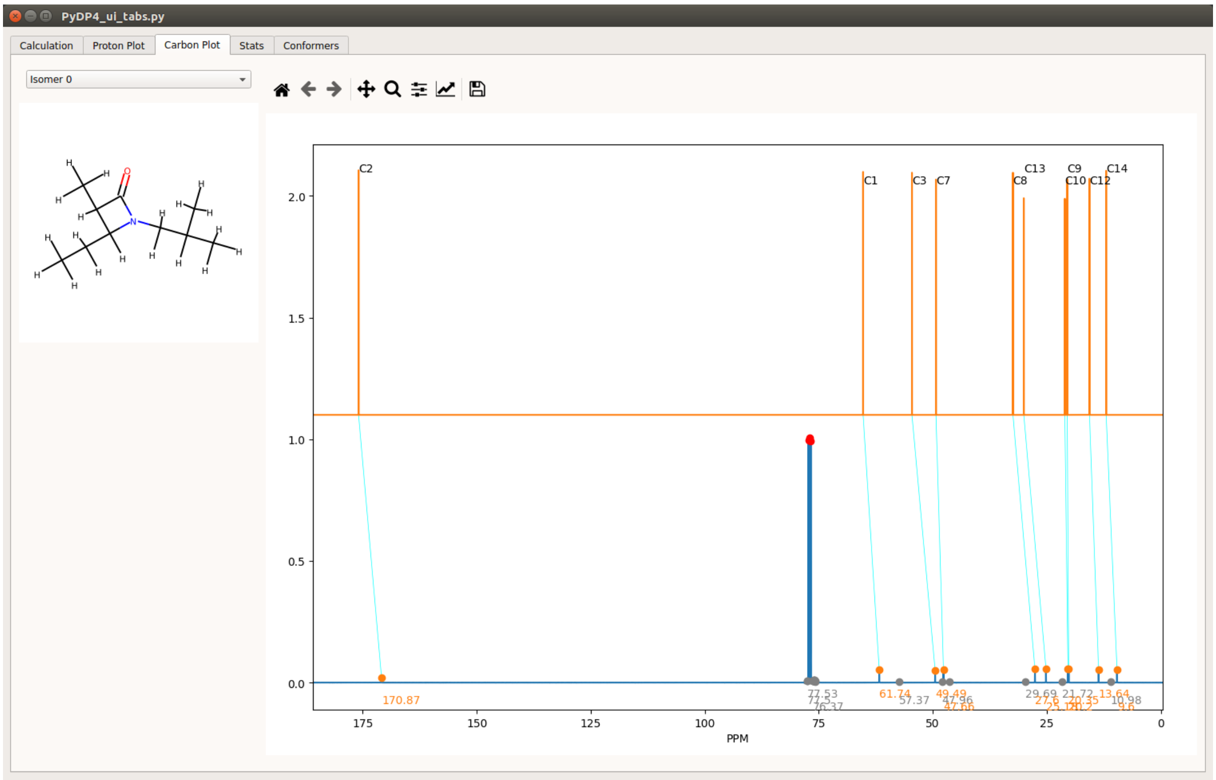

Figure 4: DP4-AIs GUI interface can be used to explore the assignments made by NMR-AI

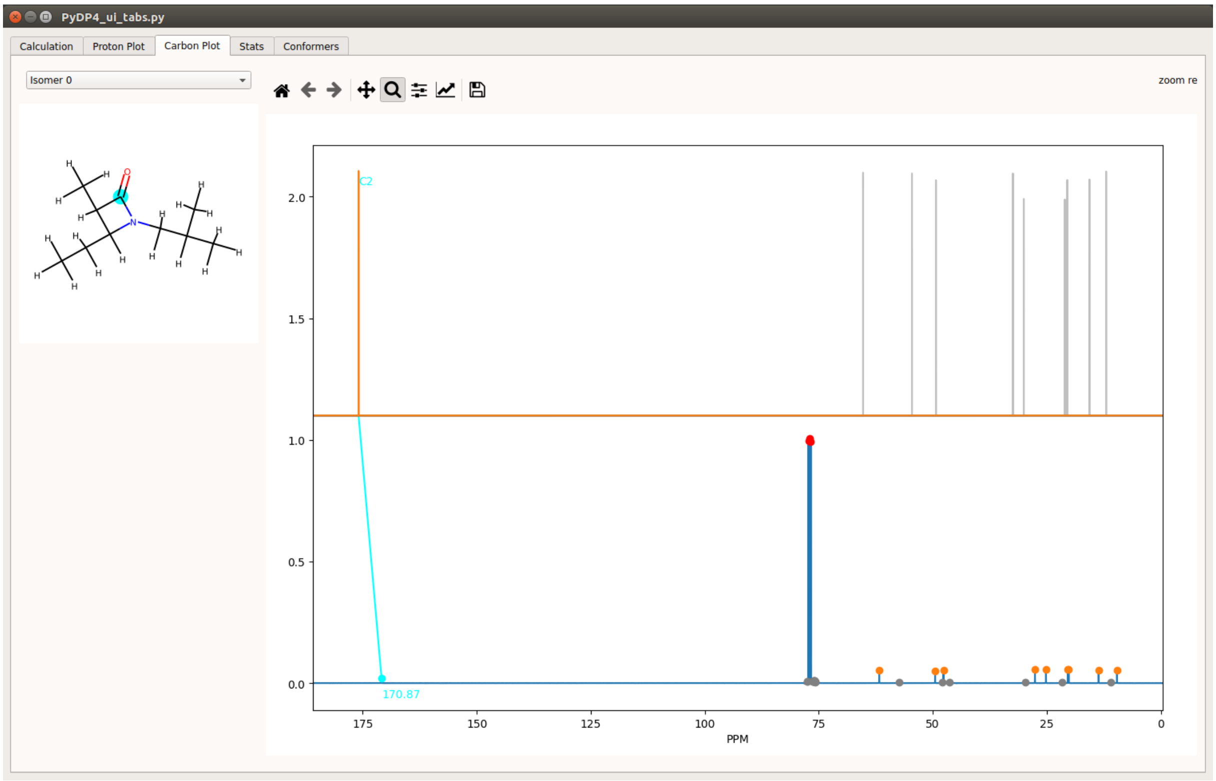

Figure 5: DP4-AIs GUI interface can be used to explore the assignments made by NMR-AI

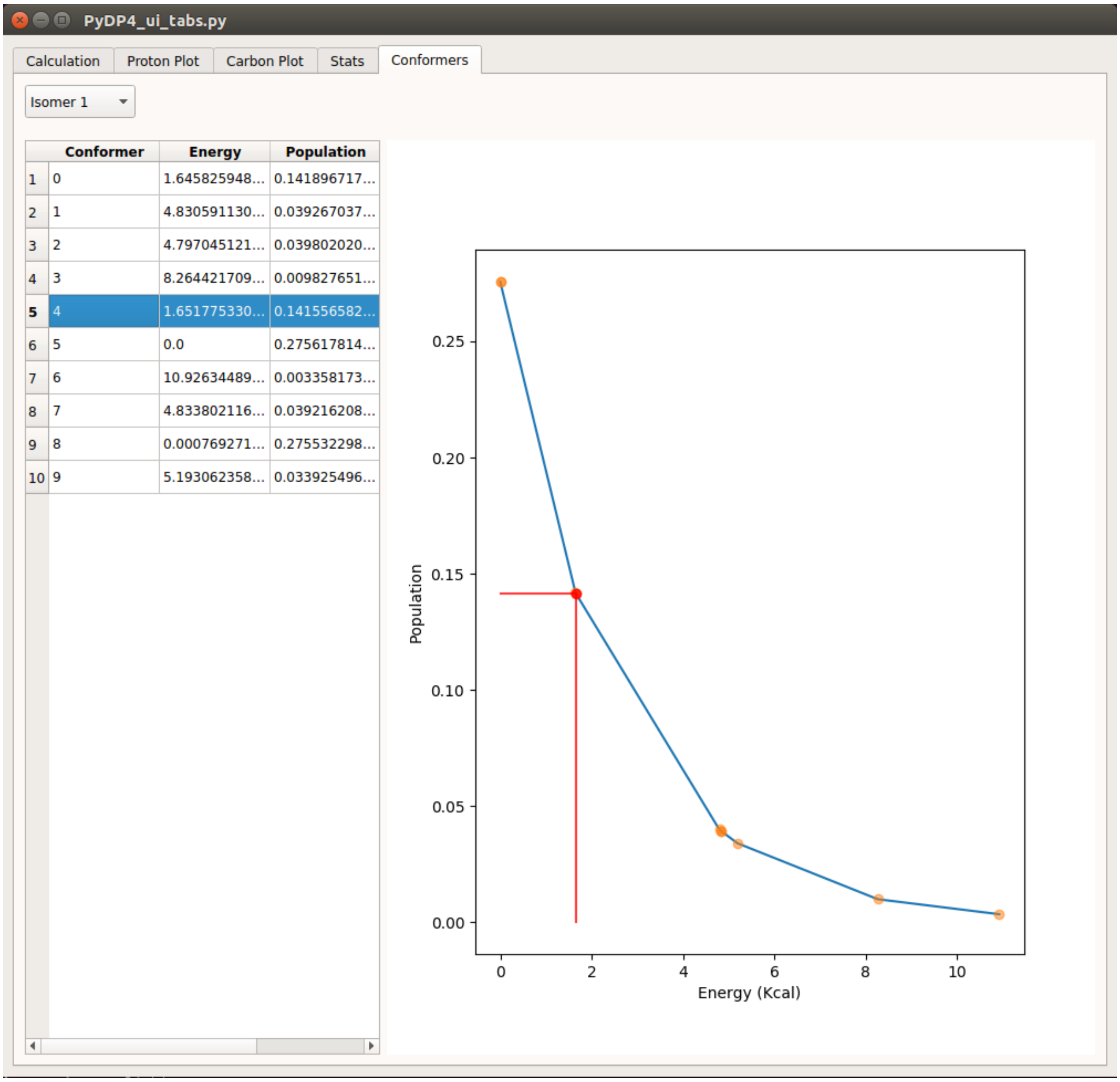

Figure 6: DP4-AIs GUI interface can be used to explore the energies and populations of the conformers generated

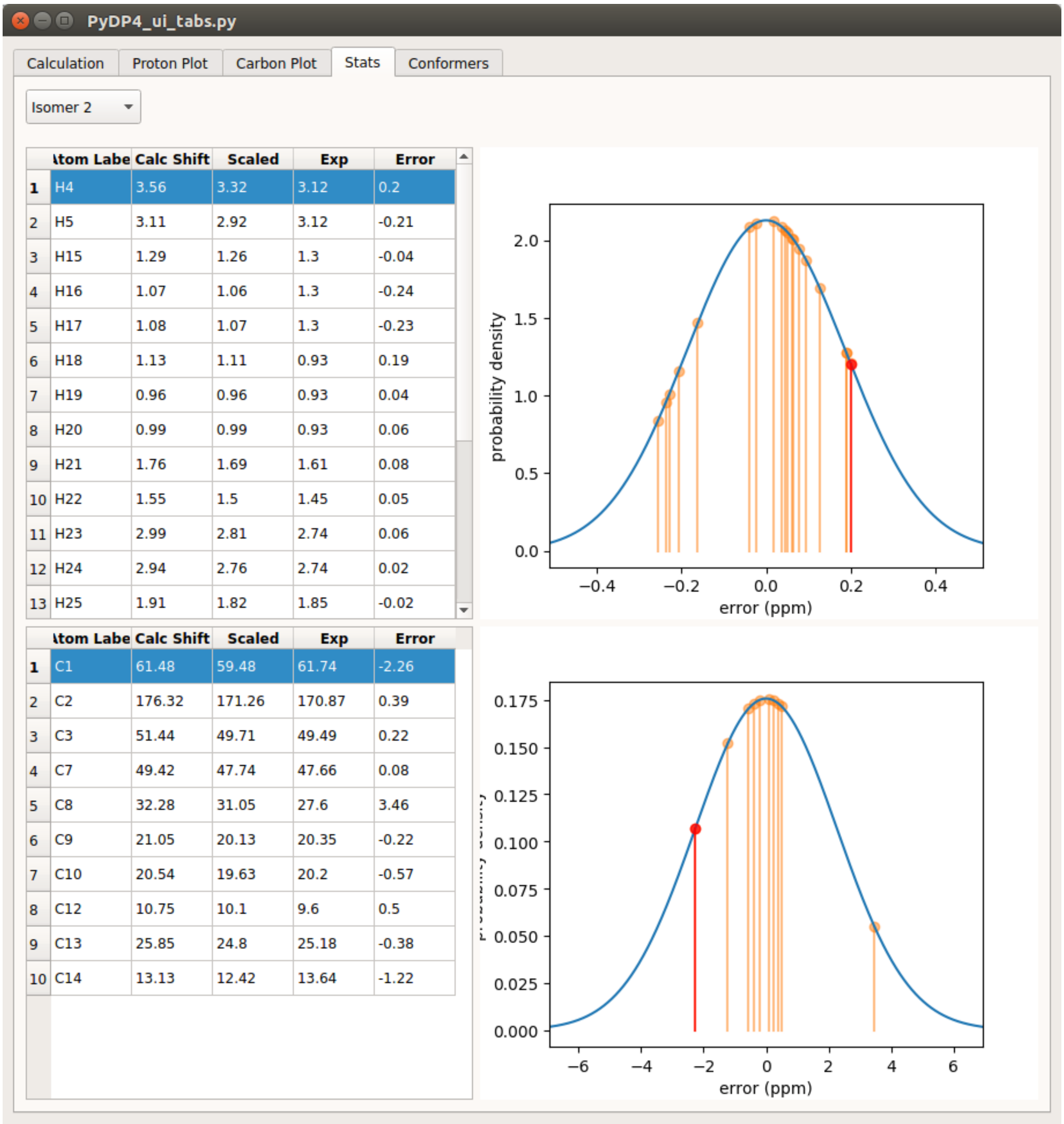

Figure 7: DP4-AIs GUI interface can be used to visualise the statistical model used by DP4 and explore the prediction errors used in the DP4 calculation

2 Program Development

In order to assign the DFT GIAO predicted shifts to peaks in the NMR spectrum, two pieces of information must be extracted from the spectrum. For ^{13}C spectra, the chemical shifts of the peaks and their intensity values. For ^{1}H spectra, the chemical shift values of the multiplet centres and their corresponding integrals. In order to extract this information, the raw NMR data must be processed. The required processing is displayed schematically in Figure 8

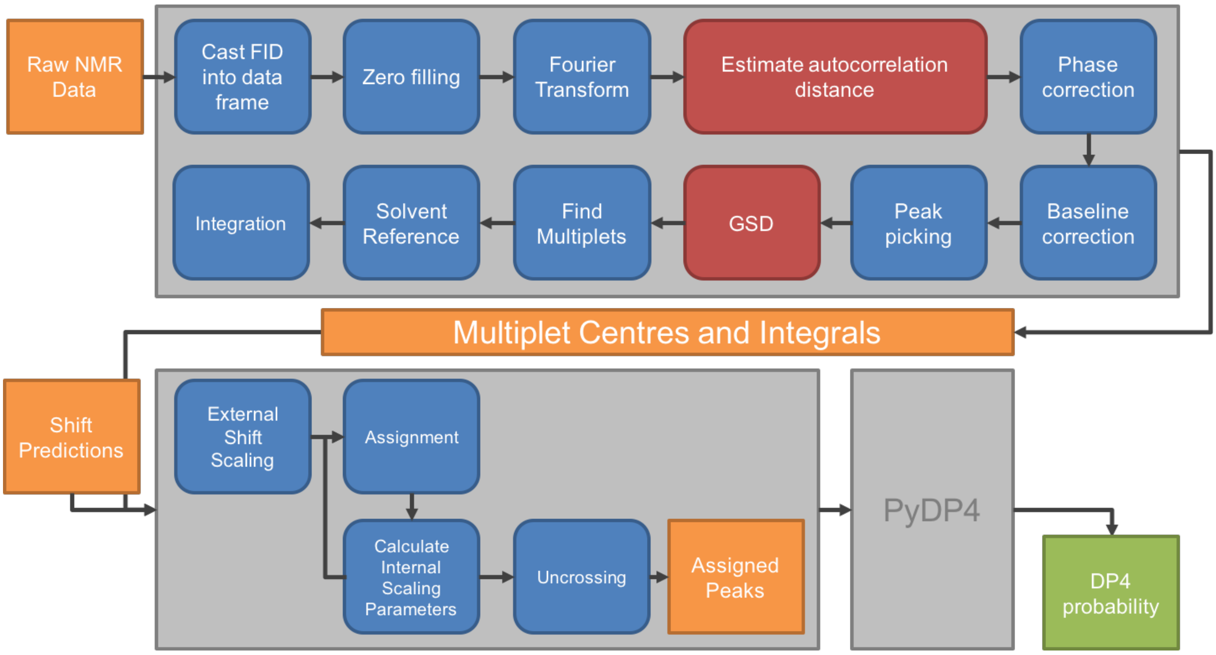

Figure 8: Figure illustrating the processing required to extract the multiplet centres and integral values needed to assign the atoms in the molecule. Following the arrows: (1) casting FID into Python section 2.1.1, (2) zero filling section 2.1.2, (3) Fourier transform section 2.1.1, (4) determining the autocorrelation distance section 2.1.4, (5) automatic phase correction section 2.1.3, (6) automatic baseline correction section 2.1.4, (7) peak picking section 2.1.5, (8) global spectral deconvolution section 2.1.6, (9) finding multiplets section 2.1.6, (10) solvent referencing section 2.1.9, (11) integration section 2.1.10.

2.1 NMR Processing

2.1.1 Initial Processing

The Python package NMRglue[1] was used to perform basic processing of the NMR data. NMRglue provides methods for reading many common NMR data file formats including Bruker, JCAMP and NMRpipe amongst others. This package was also used for basic processing of the free induction decay (FID) including, zero filling and Fourier transformation. In addition, NMRglue was utilised to extract the spectral parameters from the raw NMR data and to create a unit conversion element that is utilised throughout the program.

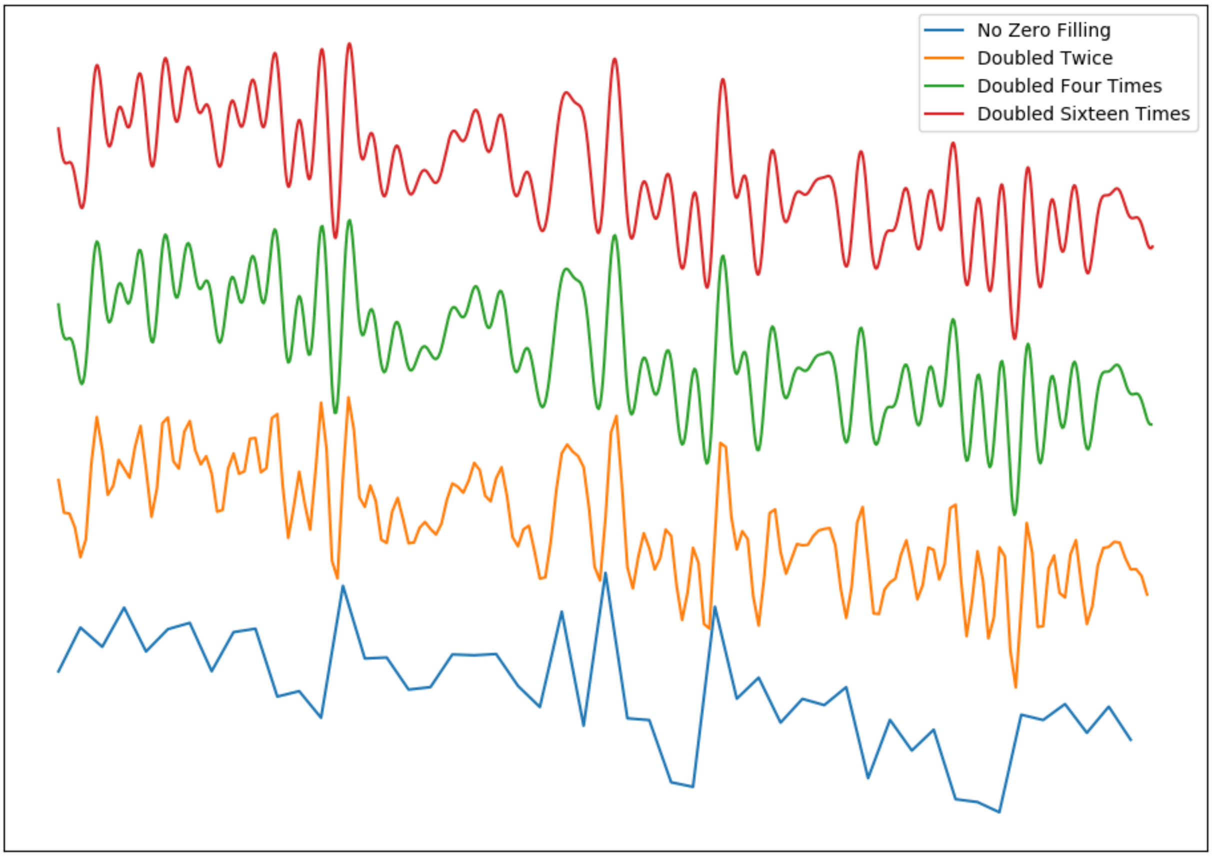

Figure 9: Figure showing a section of an NMR spectrum zero filled different numbers of times. From bottom to top (1 blue) non zero filled data, (2 orange) length of original FID doubled twice, (3 green) doubled four times, (4 red) doubled sixteen times.

2.1.2 Zero Filling

The first stage required in the processing of NMR data is zero filling. Although not often considered in much detail, zero filling has a surprising number of effects in the later processing of the NMR data. In the absence of baseline distortions, the noise in a non zero filled spectrum behaves as true white noise exhibiting no correlation. However, increasing the amount of zero filling has the effect of creating correlation within the noise. This changes the statistical distribution of the noise, which has implications in later processing of the spectrum (see section 2.1.4). Zero filling the FID leads to less information being lost in the Fourier transformed spectrum, and hence improved line shapes. It is very important to conserve these line shapes for the fitting process later in the program (see section 2.1.7). To balance computational time and memory requirements with the improved line shape, a compromise of doubling the length of the FID four times (around 500000 points for a typical ^{1}H spectrum) was incorporated into the program. A desktop computer can easily handle an object this size and little improvement in the line shape is seen with additional zero filling shown in Figure 9.

$$\text{ACME Residual} = S(R_i) + P(R_i) \tag{1}$$

The ACME residual is calculated as a sum of a Shannon entropy term (right) and negative area penalty (left).

$$S = -\sum_{i=0}^{N} h_i \ln h_i \tag{2}$$

The Shannon entropy of a 1D signal. h_i is defined in equation 3.

$$h_i = \frac{|\frac{d}{dx}R_i|}{\sum_i |\frac{d}{dx}R_i|} \tag{3}$$

h_i is the normalised derivative of the NMR data evaluated at point i.

$$P(R_i) = \gamma \sum_i F(R_i) R_i^2 \tag{4}$$

The negative area penalty function, $F(R_i)$ is given in equation 5.

$$F(R_i) = \begin{cases} 0, & \text{if } R_i \geq 0 \\ 1, & \text{if R}_\text{i} < 0 \end{cases} \tag{5}$$

$F(R_i)$ is zero for data points above zero and one otherwise

2.1.3 Automatic Phase Correction

Accurate phase correction of the spectrum is paramount to the further analysis performed by this program, as this greatly affects the peak picking, integration and the line shape fitting processes.

Automatic phasing algorithms estimate the coefficients in the phase correction function, this function is often approximated as linear and therefore there are two parameters that must be estimated, Φ_0 and Φ_1. The most common way of doing this is to optimise these parameters by minimising the residual error produced by some objective function. The automatic phasing algorithm ACME is available in NMRglue. [2] This algorithm is powerful due to its choice of objective function, equation 1.

In 1948 Shannon introduced the concept of information entropy. The Shannon entropy of a string of data is related to the amount of information it contains. As an NMR spectrum is an array of data, it has an associated Shannon entropy.

ACME first calculates the Shannon entropy of the normalised derivative of the spectrum, given by equations 2 and 3. The Shannon entropy is then added to a penalty function as shown in equation 4 to give the overall residual.

There are a number of reasons why this choice of objective function is so powerful in phasing NMR spectra. In using the derivative of the spectrum the objective function becomes independent of the baseline. In addition, a key characteristic of peaks in the absorption spectrum is that they are positive, and thus negative area should be penalised, this is the role of the penalty function. Whilst penalising negative area is common to many phasing algorithms, ACME also minimises the Shannon entropy term.

The derivative of the real NMR data is smoothest when only the absorption mode spectrum is present. The more smoother the derivative of the spectrum, the smaller the information content becomes. Consequently, the Shannon entropy of the spectrum is minimised when the spectrum is properly phased. Figure 10 illustrates this concept.

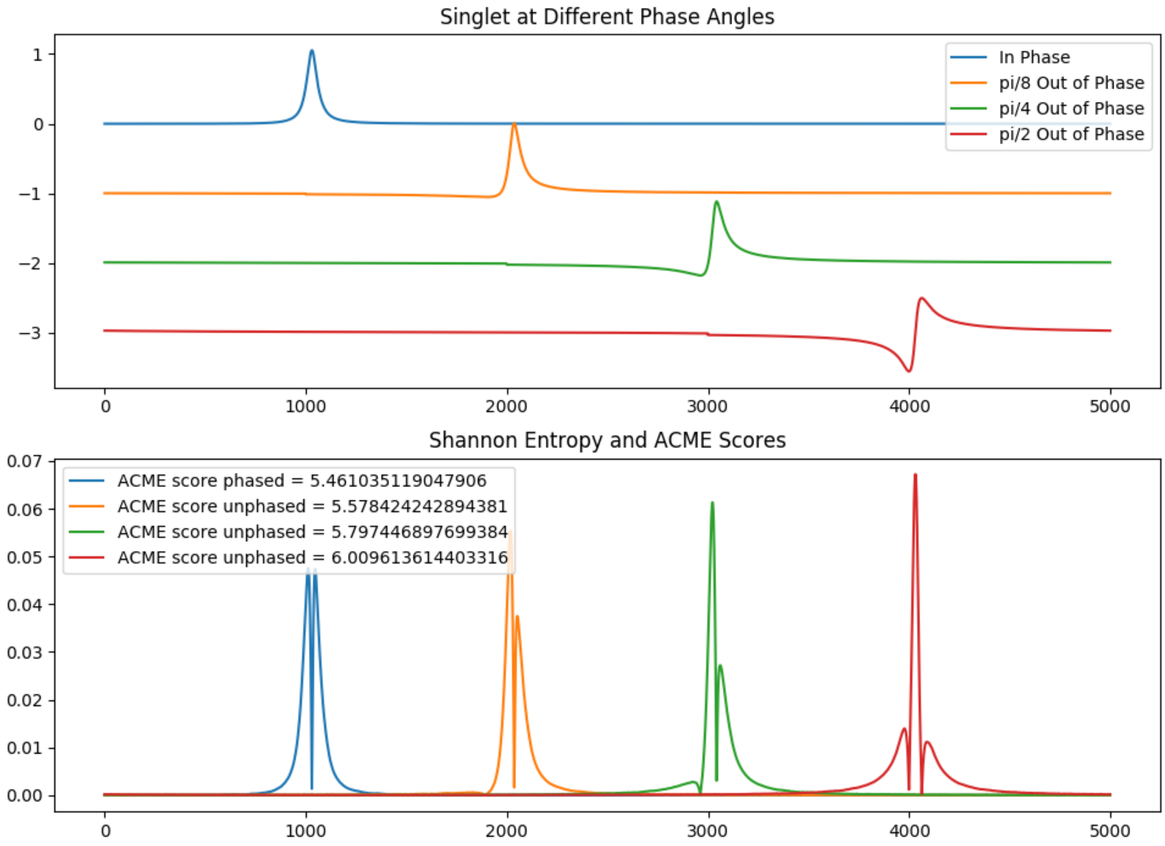

Figure 10: Top: a simulated Lorentzian at different phase angles: (1 blue) adsorption line shape (in phase), (2 orange) $\pi/8$ out of phase, (3 green) $\pi/4$ out of phase, (4 red) the dispersion line shape ($\pi/2$ out of phase).
Bottom: the corresponding Shannon entropy functions. The Shannon entropy of each singlet (the integral of the corresponding Shannon entropy function) is given in the legend.

More recently a phasing method referred to here as WLR has been developed.[3] This method provides a robust framework for estimating the phase parameters using a weighted linear regression approach.

WLR works as follows. The spectrum is divided into peak containing regions and an optimum zeroth order phase correction angle is found for each region independently. These angles are found by minimising the residual error of a different objective function. This objective function calculates a linear baseline for the region and gives the area below this baseline as the residual error. A weighted linear regression is then performed by plotting these phase angles against the corresponding regions relative position within the spectrum, weighting each point by the integral of the corresponding region. The gradient of the resulting line corresponds to Φ_1 and the intercept to Φ_0. This method is particularly robust as it finds the average optimum phase angle for only the signals in the spectrum and as it preferentially reduces negative area in regions containing more signals. This process is summarised in Figure 11.

Following the investigation of these phasing methods, it was proposed that these methods and the robust signal classification technique used in section 2.1.4 could be combined to exploit their mutual benefits, producing a hybrid phasing method. A screen was performed to quantify the efficacy of these phasing methods. A control "LMfit phasing" was also included, this algorithm minimises an objective function that returns the negative area of the spectrum as the residual. To reliably evaluate these phasing methods, the phased spectra produced in this screen have been assigned an ACME score, figure 12 and a negative area score, figure 13.

Both figures 12 and 13 suggest the hybrid phasing method outcompetes both the ACME and WLR phasing algorithms. This method was incorporated into the final program affording robust and fully automated phasing.

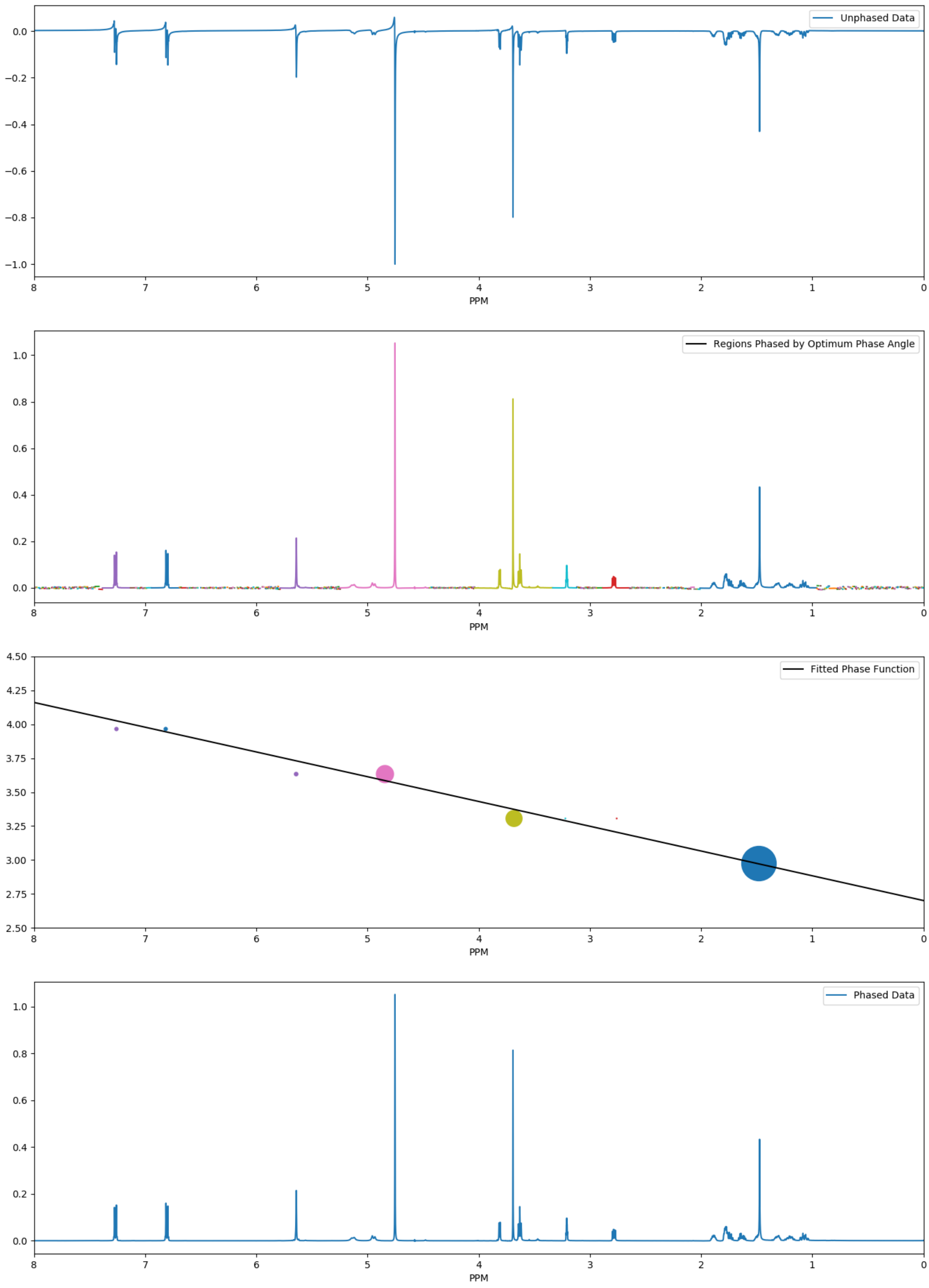

Figure 11: Summary of the WLR algorithm. From top to bottom: (1) an out of phase spectrum, (2) the spectrum divided into signal containing regions, each region phased by its optimum phase angle, (3) the weighted linear regression of phase angle against position in the spectrum, the marker size denotes the weight assigned to each region, (4) the final spectrum is phased using the parameters estimated from the weighted linear regression

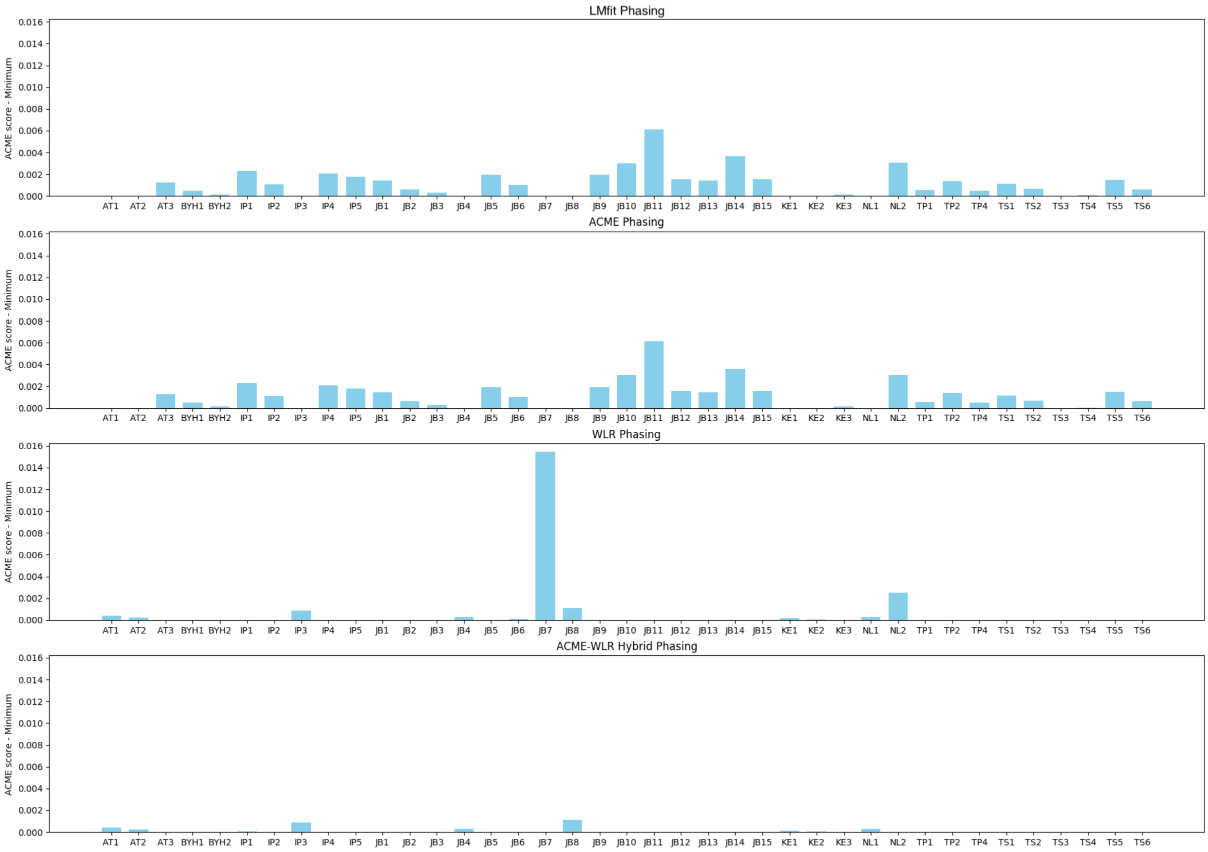

Figure 12: Results of the phasing method screen, this bar chart displays the final ACME residuals as calculated by equation 1 divided by the ACME score of the unphased spectrum. The minimum score for each spectrum is also set to zero. From top to bottom: (1) LMfit phasing control, (2) ACME phasing, (3) WLR phasing, (4) ACME-WLR hybrid phasing.

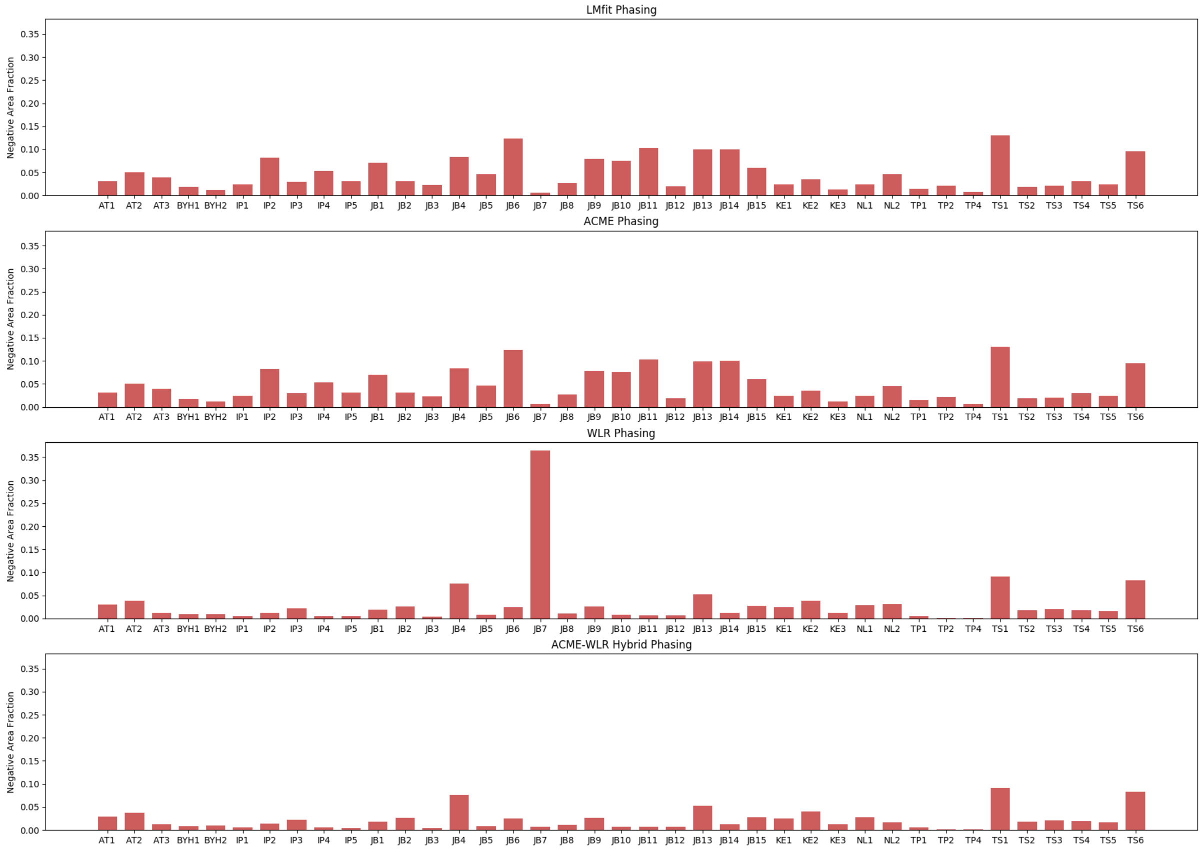

Figure 13: Results of the phasing method screen, this bar chart displays the final negative area integrals expressed as a fraction of the integral of the modulus of the spectrum. From top to bottom: (1) LMfit phasing control, (2) ACME phasing, (3) WLR phasing, (4) ACME-WLR hybrid phasing

2.1.4 Automatic Baseline Correction

A simple method for baseline correction would be to fit a function with a small number of parameters to the baseline of the spectrum and subtract it. The major disadvantage of this method is that the functional form of the baseline must be assumed. However, there is no guarantee that the baselines of all spectra will vary with the same functional form.

To overcome this difficulty a number of baseline correction methods have been developed in the literature.[4–7] These algorithms assume that both the noise and signals in the spectrum are high frequency information, whereas the baseline distortions are typically low frequency information. By progressively smoothing the spectrum using different filtering techniques, the high frequency information will be removed leaving an estimate of the shape of the baseline.

This does leave a difficulty in the treatment of, broad signals, the bases of signals and regions with highly overlapping signals as these also contain low frequency information. If these are erroneously incorporated into the baseline, this information will be lost.

Within NMRglue a method for automatic baseline correction is provided.[7] This method was developed to reliably and automatically correct baseline distortions in the often complex and highly overlapping NMR spectra taken in metabolomics studies. The major advantage of this algorithm is that only a small number of statistically optimised parameters are required for signal classification, and therefore it can afford fully automatic baseline correction. A slightly modified version of this algorithm was incorporated into this program and works as follows.

In order to classify points as signal or noise, the standard deviation of the noise must be estimated. First the standard deviation around each point in the spectrum is calculated. A histogram of these values is then plotted. The resulting distribution is composed of two separate distributions; the normal distribution of the noise region standard deviations, and the distribution of signal and mixed noise-signal regions standard deviations. The later contains far fewer points with a much longer tail. The median value of the overall distribution is found and any points more than twice this value are removed. If this is repeated, the median converges at the value of the noise standard deviation.

This process has proven to be robust and reliable. However, it does not take into account that the noise in a zero filled NMR spectrum is correlated. The correlation of the noise can be characterised by the autocorrelation distance. The autocorrelation function of a 1D signal $y(x)$ is defined by equation 6 which is often approximated as an exponential function as shown in equation 7, giving the autocorrelation distance τ_c.

By making this assumption, the autocorrelation distance of the signal can be estimated as the number of points that gives $\frac{f(\delta t)}{f(0)} = \frac{1}{e}$ which can easily be estimated using a small sample of noise. A slight difficulty is that, any baseline distortions in this sample will lead to large autocorrelation distances, hence a simple baseline correction is made by fitting a polynomial function to this sample as shown in Figure 14. The autocorrelation distance can then be estimated as shown in Figure 15. The value of $f(\delta t)$ can be calculated increasing δt in a stepwise fashion, the value of δt that gives $\frac{f(\delta t)}{f(0)} = \frac{1}{e}$ is taken as the autocorrelation distance.

$$f(\delta t) = \sum_{t=0}^{N} y(t)y(t+\delta t) \tag{6}$$

The autocorrelation function of a 1D signal. Where $y(t)$ is the signal at point t, δt is a small distance and N is the total number of points in the signal.

$$\left(\sum_{t=0}^{N} y(t)^2\right) e^{-\frac{\delta t}{\tau_c}} = \sum_{x=0}^{N} y(t)y(t+\delta t) \tag{7}$$

Autocorrelation function approximated as an exponential function giving τ_c, the autocorrelation distance of the signal

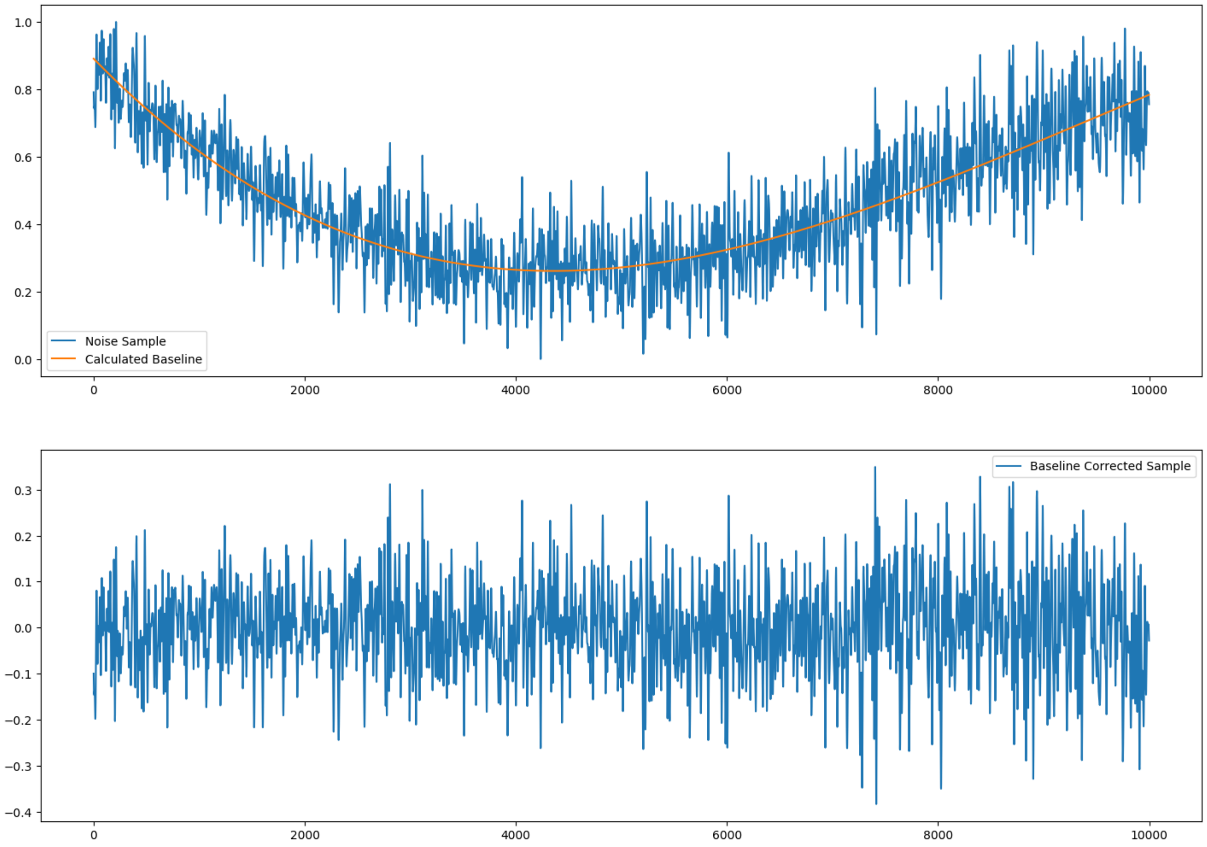

Figure 14: A small sample of noise undergoing a simple polynomial baseline correction

The spectrum is separated into a number of samples containing only points further than twice the autocorrelation distance from each other. Assuming these points are not correlated, the standard deviation of each of these samples can then be estimated using the method above. The mean of these values is then taken as the true noise standard deviation. Figure 16 illustrates how not sampling the spectrum in this way leads to an underestimate of the true noise standard deviation and how this modified method leads to a more accurate estimate.

Once the standard deviation of the noise has been accurately estimated, a point is classified as signal if the standard deviation measured around it is larger than 1.1 times the noise standard deviation. [7] Signal points are grouped together if they are less than the autocorrelation distance apart.

The baseline is then calculated as a smooth univariate spline fitted to the regions of the spectrum classified as noise, the baseline in signal regions is estimated as a linear interpolation between the noise points at the edges of the region. The baseline is then subtracted from the spectrum to give the final corrected spectrum as illustrated in. Figure 17

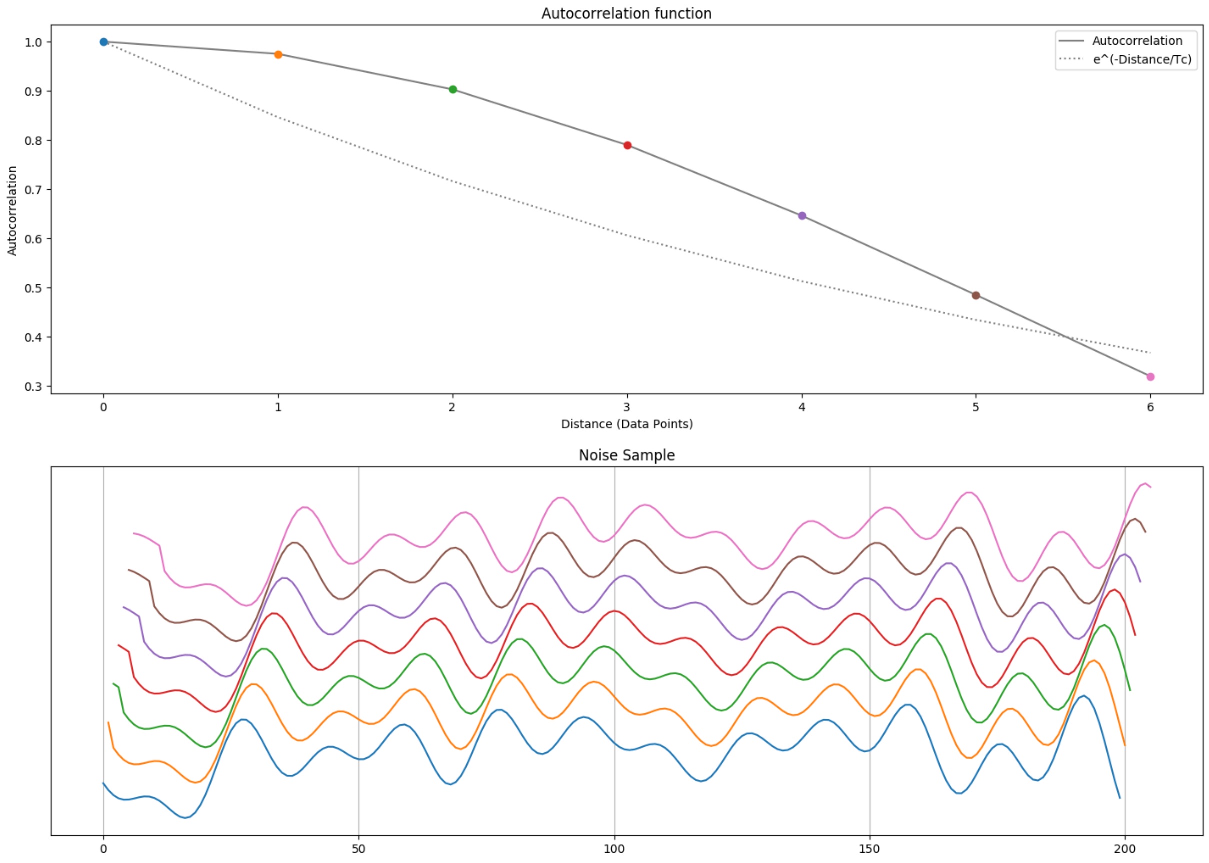

Figure 15: Figure illustrating the estimation of the autocorrelation distance of a 1D signal. Top: The value of $\sum_{x=0}^{N} y(t)y(t+\delta t)$ at different values of δt. The dotted line represents $e^{-\frac{\delta t}{\tau_c}}$.
Bottom: (1 blue) the 1d signal $y(t)$, (2 orange) $y(t-1)$, (3 green) $y(t-2)$ etc. The integral of the product of the blue signal with each shifted coloured signal it gives the corresponding coloured point in the plot above.

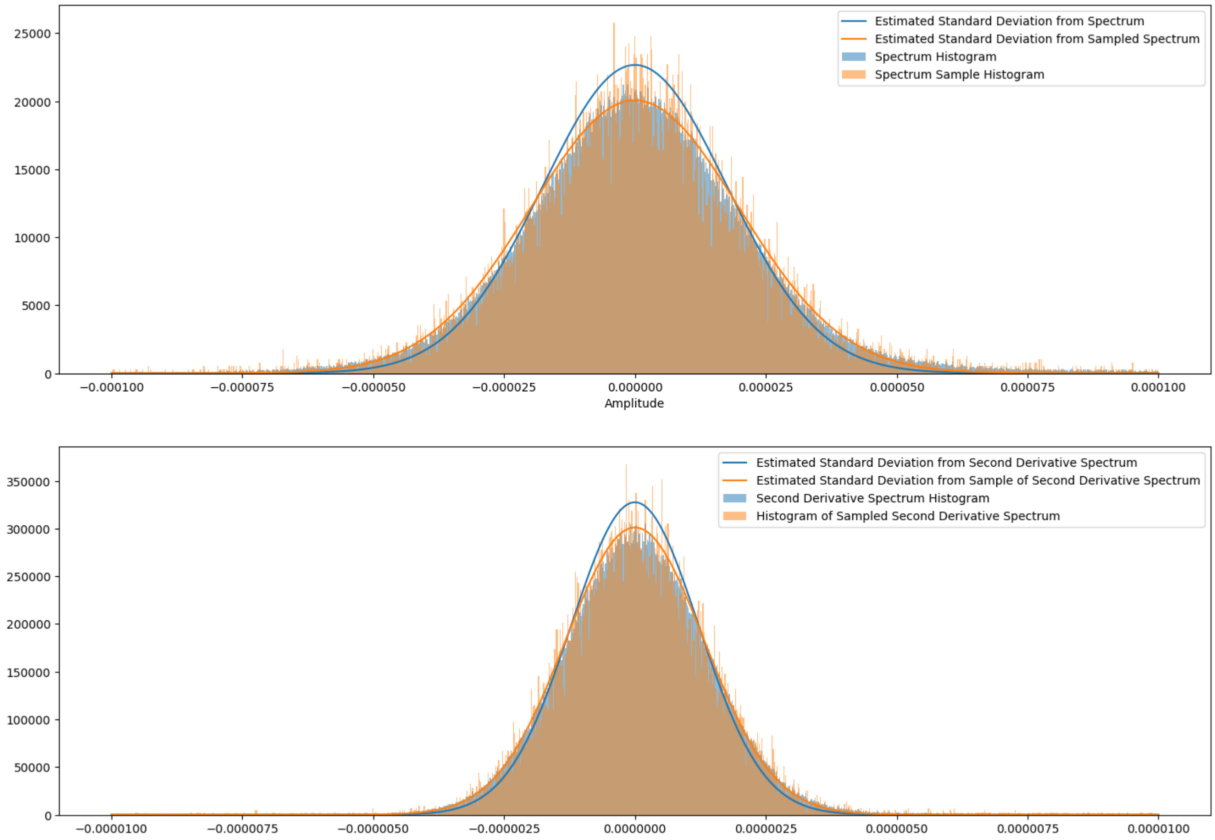

Figure 16: Figure illustrating when estimating the standard deviation of the noise in the spectrum, correlation must be taken into account. Top: the blue histogram represents all the points in the spectrum. The blue curve is a normal distribution with standard deviation estimated from the blue histogram, notice the standard deviation is smaller that of the blue histogram. The orange histogram represents points of the spectrum sampled at intervals larger than the autocorrelation distance, notice the standard deviation is the same as that of the blue histogram. In contrast, the orange curve a normal distribution with standard deviation estimated from the orange histogram is now the correct width. Bottom: This plot is the same as above but represents the second derivative spectrum.

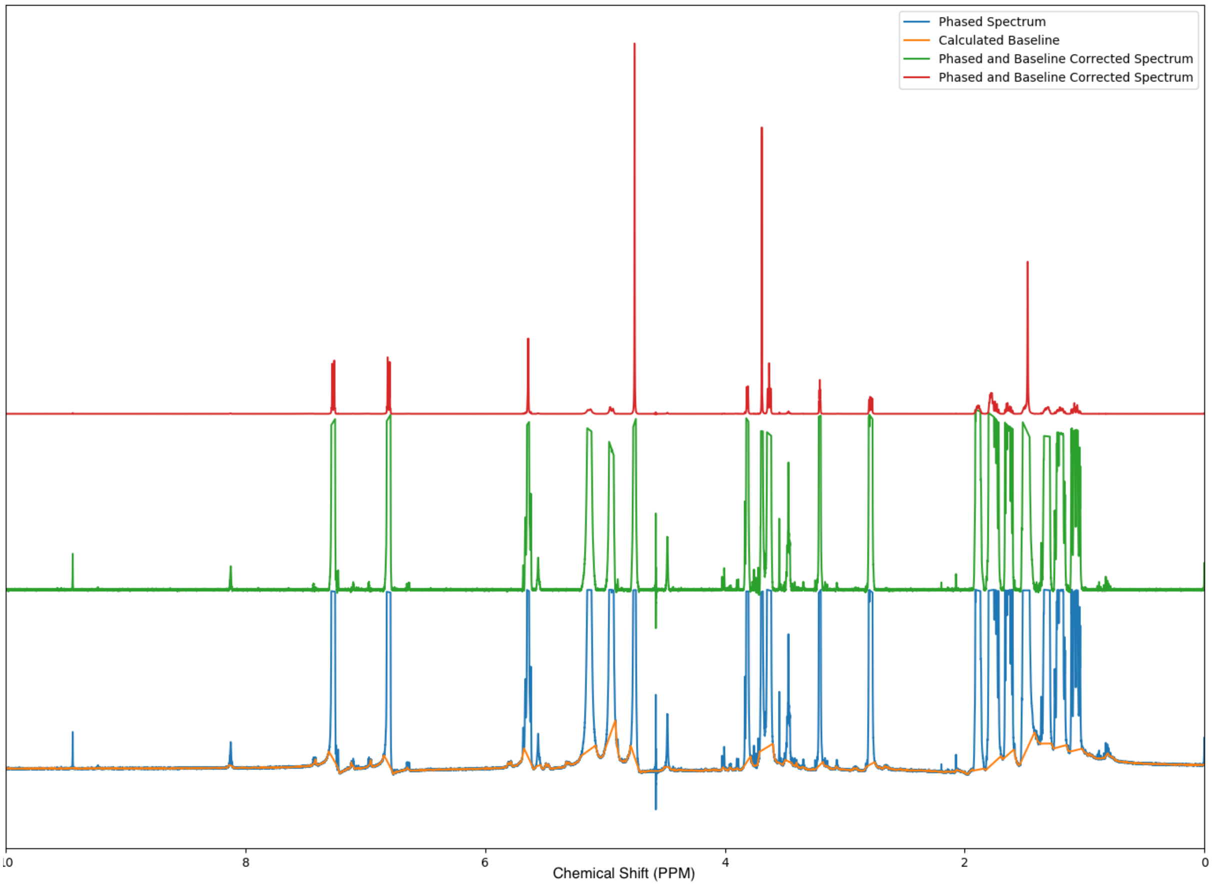

Figure 17: Figure illustrating how a calculated baseline is used to correct a spectrum. From bottom to top: (1 blue) the uncorrected spectrum, (2 orange) the calculated baseline, (3 green) the corrected baseline, (4 red) the final spectrum.

2.1.5 Gradient Peak Picking

Sections 2.1.5 to 2.1.8 describe the peak picking and modelling process employed for ^{1}H spectra. A simplified version of this process employed for ^{13}C spectra.

The signal identification method utilised in the baseline correction algorithm was adapted to estimate the standard deviation of the second derivative of the noise. Minima in the second derivative are picked as signal if their value is less than twice the standard deviation of the second derivative spectrum. The picked minima in the second derivative must also be maxima in the spectrum greater than five times the noise standard deviation to be classified as peaks. In using two adaptive threshold values, both values may be kept low avoiding missing signals whilst also screening out noise. This process is summarised in Figure 18.

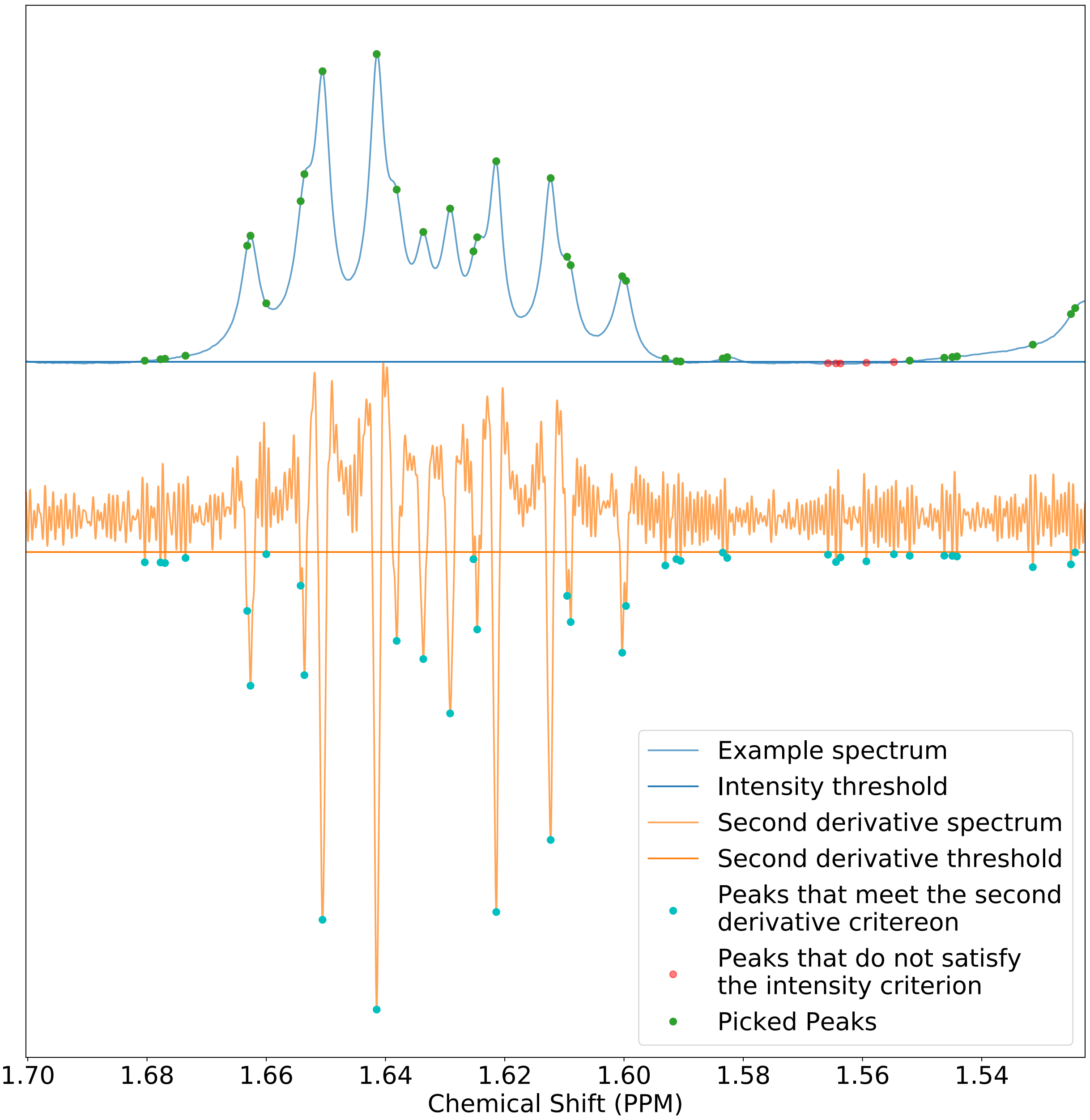

Figure 18: Figure illustrating the gradient peak picking process on a section of a ^{1}H NMR spectrum. In order to be classified as signals peaks must meet two criteria. The peaks must be minima in the second derivative spectrum (shown in orange) below a certain threshold (also shown in orange) and a maxima in the spectrum (shown in blue) above a second threshold (also shown in blue). The final picked peaks are highlighted in green

2.1.6 Spectral Modelling

In ^{1}H spectra signal peaks must be grouped together to establish where the multiplet centres are located. The maximum coupling constant expected to be seen between protons in ^{1}H spectra is around 18 Hz. Any peaks < 18 Hz apart can be grouped together as multiplets. As the peak picking signal to noise ratio is deliberately set very low, this increases the probability of noise peaks being mistaken for signal peaks. These noise peaks can bridge gaps between adjacent multiplets, leaving only a single peak centre to describe a number of multiplets. The differences between the NMR spectra of diastereomers are very subtle and hence to perform a reliable DP4 calculation the multiplet centres must be known as accurately as possible.

The solution to this problem is to construct a model describing each signal containing region of the spectrum with minimal set of parameters.[8 9] It is almost always possible to reduce the residual error of an empirical model by increasing the number of parameters used to describe it. Eventually as more and more parameters are added to the model, the information gained by the reduction in residual error does not compensate for the information required to define the additional parameters. The Bayesian information criterion (BIC) defined in equation 8 is a statistical parameter that can be used to objectively compare two models, taking into account their number of parameters and their residual error.[10 9] The model with the lowest BIC provides the optimal balance of parameter number and residual error. If it is assumed that parameters describing noise peaks do not contribute to the information content of the model, the required model is the one where no parameters can be added or removed to lower the BIC.

The modelling procedure is carried out as follows. Picked peaks separated by at less that 18Hz are grouped together to define signal containing regions. For each region a model is constructed with enough parameters to describe an individual line shape function for each peak in the region (see section 2.1.7). These parameters are then fitted to the spectrum to produce the initial model (see section 2.1.8)

The groups of parameters describing each peak are then tested. A new model is constructed without each group in turn, if the BIC is lower than that of the previous model by more than a threshold value (here set to 15[9]), these parameters (and the peak they describe) are deleted from the model and the testing continued.

Once all of the noise peaks have been removed from the model, the signals can be regrouped into multiplets. In addition to removing noise, decomposing the spectrum in this way is also very useful in later parts of the program and also will be useful in extension of the current program.[11] Examples of this modelling process are displayed in Figures 19 and 20.

$$\mathrm{BIC} = N \ln(\frac{\chi^2}{N}) + \ln(N) N_{\mathrm{parameters}} \tag{8}$$

Equation for the Bayesian information criterion. Where χ^2 is defined in equation 9, N is the number of data points and $N_{\mathrm{parameters}}$ is the number of parameters.

$$\chi^2 = \sum_i^N r_i^2 \tag{9}$$

The chi squared equation. Where N is the total number of data points and r_i^2 is the residual error at point i

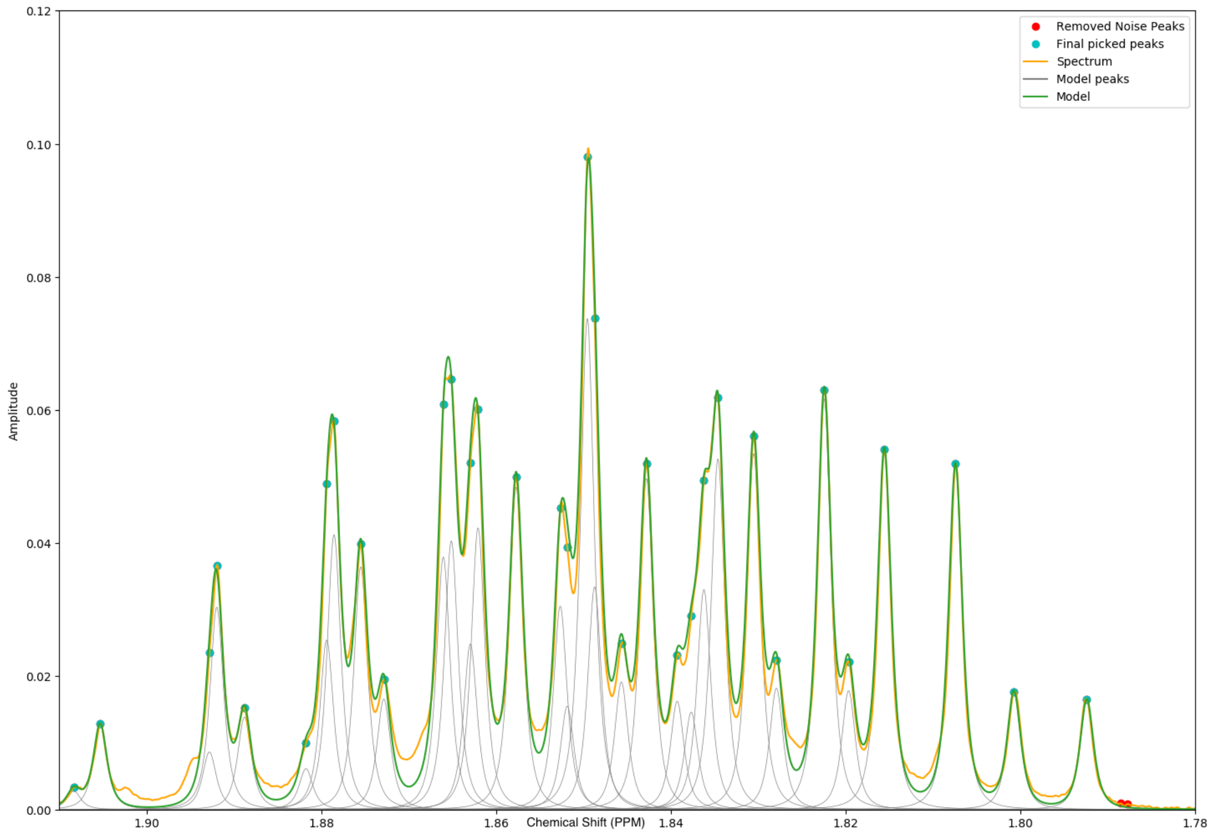

Figure 19: An example multiplet (yellow) and deconvolved model (green). Note how the spectrum has been deconvolved into the series of grey curves that make up the model.

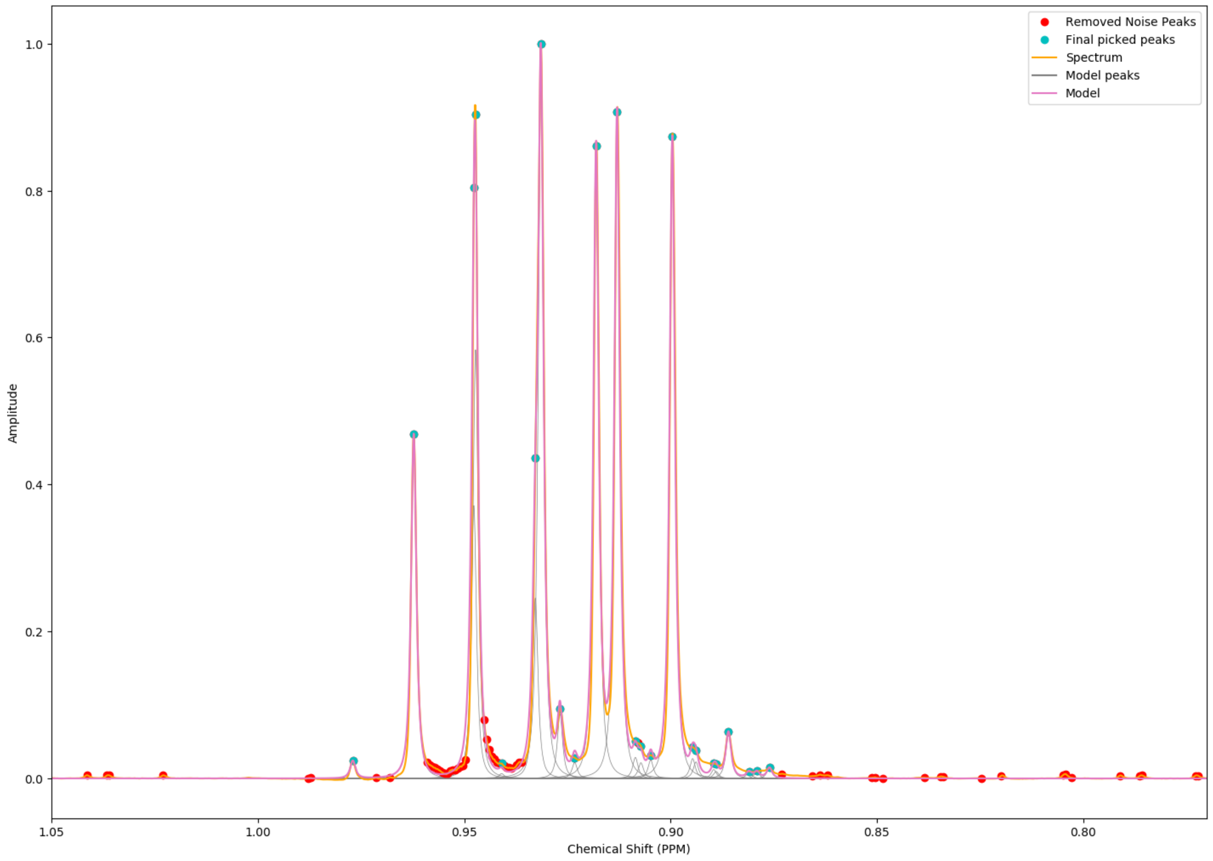

Figure 20: An example multiplet (yellow) and deconvolved model (pink). Note how the noise peaks highlighted in red have been removed from the model leaving only the cyan signal peaks.

2.1.7 Spectral Line Shape

In order to deconvolve the spectrum into a set of parameters, a function that can accurately describe the line shapes of individual peaks must be chosen. An ideal NMR spectrum will display perfectly Lorentzian line shapes. It was discovered that this line shape is seldom found in reality. This variation is caused by many factors such as, the shimming when taking the spectrum, the phasing and baseline correction used and amount of zero filling. Consequently, a more general function with enough parameters to allow for variation in line shapes seen in different spectra was sought.

A number of different functions were tested. These functions were initially screened by fitting them to an isolated singlet and measuring both the absolute sum and standard deviation of the residual errors. The result of this analysis is shown in Figure 21.

From this study it was concluded that the generalised Lorentzian (GL),[12] Pearson VII and Voigt distributions were the most promising line shapes, these functions all have an additional kurtosis parameter allowing for additional variation. As shown in Figure 21 a Lorentzian function and a Voigt distribution with an additional phase parameter were also tested. These would potentially allow for slight phasing errors to improve the fit. However, it was found that these functions typically fit less reliably.

The highest scoring distributions, Lorentzian, Pearson VII, Voigt and GL distributions were then tested further. Each function was used in the fitting process (see section 2.1.8) to model all of the spectra in the dataset. Figure 22 plots the resulting normalised residuals as defined by equation 10.

It was found that the efficacy of many fitting methods tested (see section 2.1.8) was highly dependent on the line shape function used. In contrast, figure 22 illustrates how the final fitting method produced similar residual errors with all the line shape functions tested. This reflects the robust nature of the fitting process. Following this result, the GL distribution was chosen as the optimum function as it gives low residual errors and also due to ease of integration, see section 2.1.10.

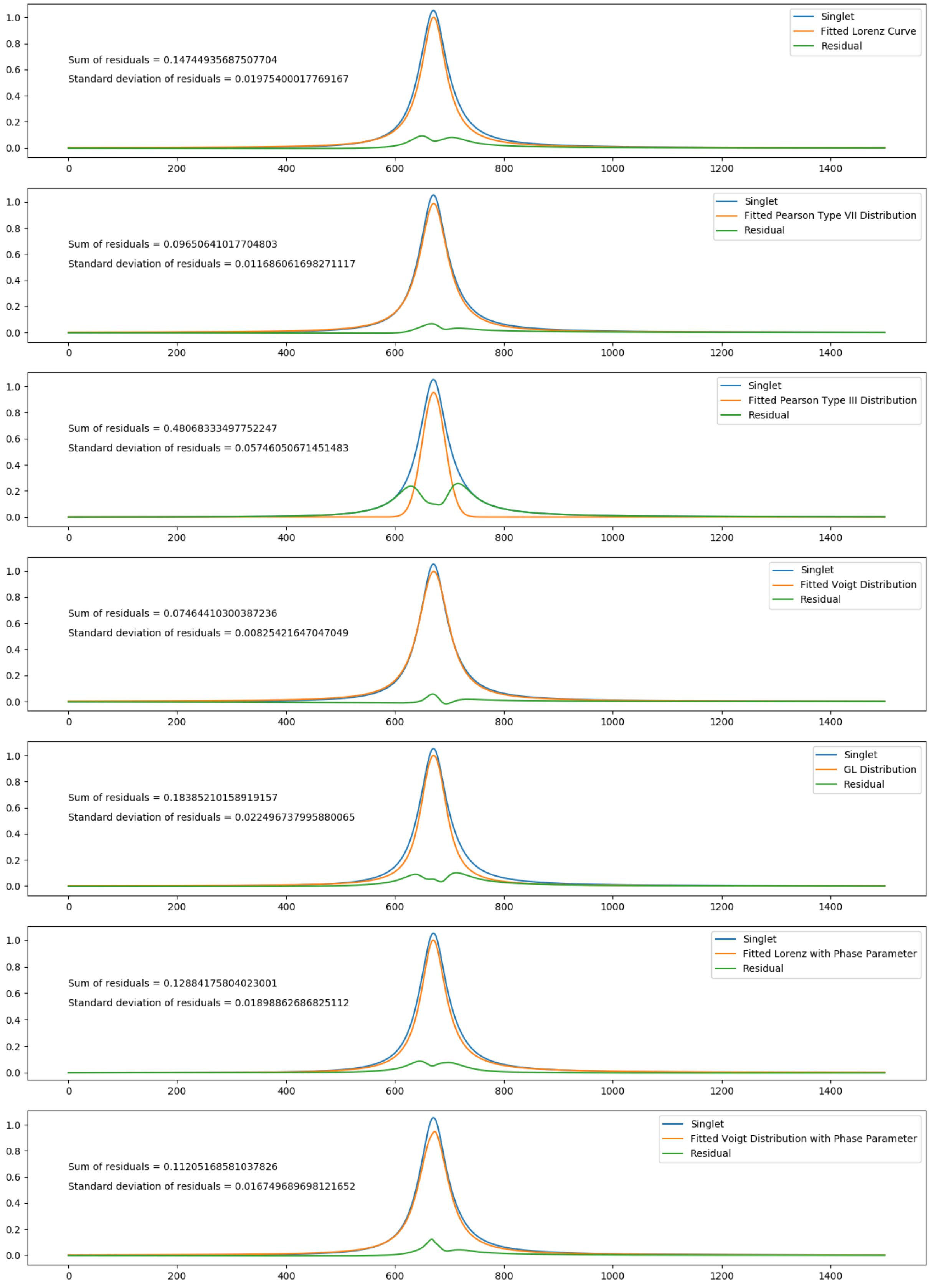

Figure 21: Figure displaying the results of fitting different line shapes to an isolated singlet. The functions used from top to bottom: (1) Lorentzian, (2) Pearson VII, (3) Pearson III, (4) Voigt, (5) generalised Lorentzian,[12] (6) Lorentzian with phase parameter, (7) Voigt with phase parameter

$$\text{normalised residual} = \sum_{j}^{R} \frac{\sum_i^N |f(x_{ij}) - y(x_{ij})|}{\sum_i^N y(x_{ij})} \tag{10}$$

Equation for the normalised residual. Where R is the number of peak containing regions, N is the total number of points in the spectrum, $f(x)$ is the fitted function and $y(x)$ is the Intensity data

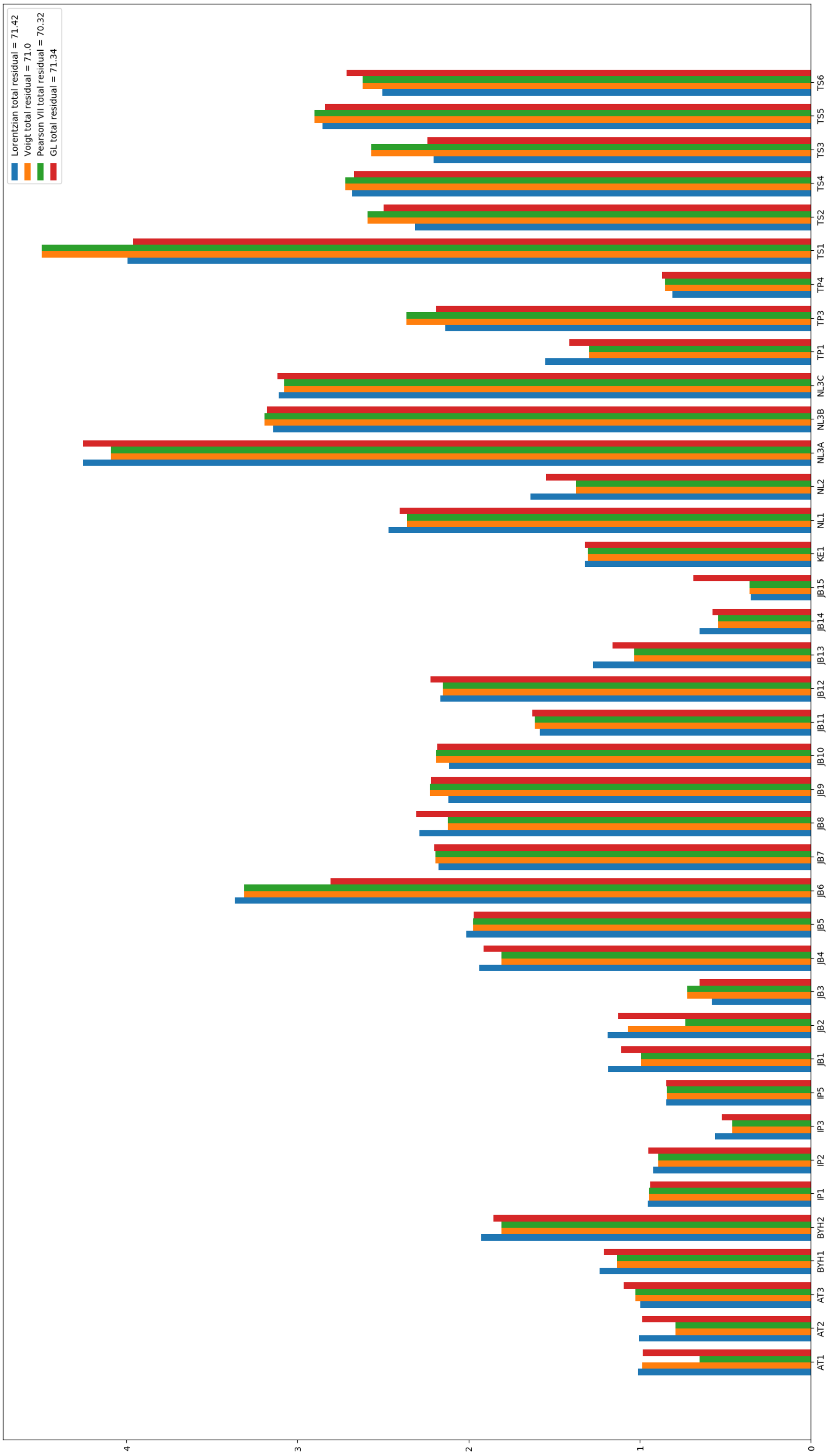

Figure 22: Figure displaying the normalised residuals (10) from fitting different line shapes to a database of proton NMR spectra. (blue) Lorentzian, (orange) Voigt, (green) Pearson VII, (red) generalised Lorentzian.

2.1.8 Fitting Process

Achieving efficient optimisation of multidimensional functions with limited computing power, is a problem that permeates throughout many areas of modern science and mathematics.

A full model of a peak containing region of the spectrum with a GL(position, width, amplitude, kurtosis) distribution for all M peaks will contain 3M + 1 parameters (as the kurtosis has been taken as a regionwise parameter). For example, if a region has 30 peaks, 91 parameters will need to be optimised in order to produce the full model. Each spectrum may have around 10-20 regions and therefore the number of parameters to be optimised quickly becomes very large. Optimising all these parameters at once leads to long computational times which also scale poorly with the number of peaks. Therefore, an approximate method for fitting these parameters was developed.

A large number of different methods were investigated and tested. These methods all contained a curve fitting routine at their core, implemented using the LMfit Python package.[13] These methods used different combinations of, sequentially fitting individual peaks, optimising groups of parameters whilst fixing others in turn, using different residual functions, using different fitting routines and constraining the parameters to different ranges and initial conditions. All of these methods suffered the same problem, by constraining the fitting process, the residual error was never as low as when all the parameters were optimised together. In addition, the increase in error was not compensated for by the decrease in computational time.

It was found that approximate values for the amplitude and width parameters for each peak could be found using a simple procedure. The parameters describing all of the peaks are initially added to the model. The widths are set to the same value of 0.5Hz. The amplitude parameters are set to the amplitude of the corresponding peak in the spectrum, producing the initial model of the region. There are three steps in the fitting process, first the integral of the model and the corresponding region of the spectrum are calculated. The widths of all the peaks are adjusted by equation 11. Following this, the amplitudes are adjusted by equation 12. This process is repeated until the integral of the model is within 1% of that of the spectrum. This process is illustrated using a simulated peak in Figure 23. After the model has converged, the kurtosis and position parameters are optimised by least-squares minimisation.

$$\text{width}^i_{n+1} = \text{width}^i_n \frac{\int y(x)dx}{\int m_n(x)dx} \tag{11}$$

Equation for the width adjustment step. Where $m(x)$ is the model intensity data, $y(x)$ is the real intensity data and width^i_n is the width of peak i at step n

$$\text{amplitude}^i_{n+1} = \text{amplitude}^i_{n'} \frac{y(x^i)}{m_{n'}(x^i)} \tag{12}$$

Equation for the amplitude adjustment step. where amplitude^i_n is the amplitude of peak i at step n, $y(x^i)$ is the value of the real intensity data at the location of peak i and $m_{n'}(x^i)$ is the value of the width adjusted model at the location of peak i

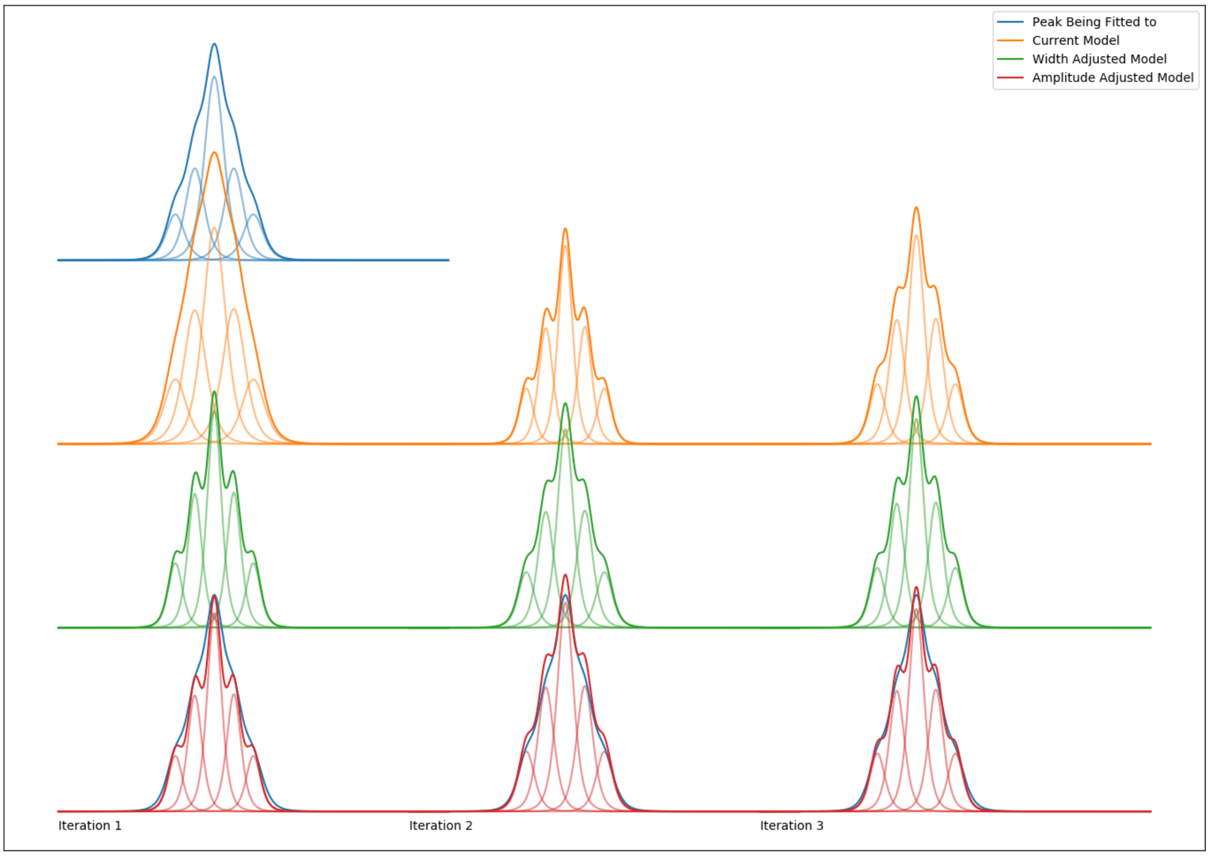

Figure 23: Figure displaying three iterations of the fitting process on a simulated peak (blue). This figure is read from top to bottom, left to right. The initial model is displayed in the leftmost curve on the second row (orange), the width adjustment is applied to give the curve below it (green), followed by the amplitude adjustment to give the left most curve on the bottom row (red). This curve then becomes the initial model in the second column and so on.

2.1.9 Solvent Peak Identification and Spectrum Referencing

Deuterated solvents often produce multiplets with distinct line shapes that are very well characterised, this can be exploited in their identification. Once the solvent peak has been identified, it must be removed from the list of assignable peaks and the spectrum must be referenced to minimise systematic errors in the peak centre values. The method used to perform this task must be able to identify many known NMR solvents including those that produce multiple peaks. It must also be able to cope with situations where the solvent is present in different stoichometry to the sample. The method developed works as follows.

The NMR solvent used when taking the spectrum is known as this was parsed to PyDP4 when defining conditions for DFT calculations. The program contains a database of, common solvents, their chemical shifts and J values for each peak they are expected to produce. New NMR solvents can also be easily added to this database.

Initially the region of the spectrum closest to the location of the first expected solvent multiplet is determined and a testing process is performed on each peak in this region. A simulated solvent multiplet is calculated using the known properties of the solvent and centred around the test peak with its amplitude scaled to match as shown in Figure 24. Three parameters are then calculated whilst the simulated solvent multiplet is centred around each peak. The first is an amplitude residual parameter, calculated as the sum of the absolute differences in amplitude between each component peak of the simulated solvent multiplet and the corresponding closest peak in the spectrum as shown in Figure 25. The amplitude of each peak is taken from the model spectrum (see section 2.1.6), preventing any overlapping multiplets affecting this process. The second parameter is a distance residual calculated in the same way, also shown in Figure 25. The third parameter simply decays as the peak being tested gets further from the expected location of the solvent peak. The overall score for each peak is given by the geometric mean of these parameters as shown in Figure 26.

The peak that gives the lowest score is selected as the centre of the solvent multiplet, the peaks that make up the solvent multiplet are then removed from the peak list and removed from the model spectrum. The spectrum is then referenced. This process is repeated for all of the multiplets the solvent is expected to produce.

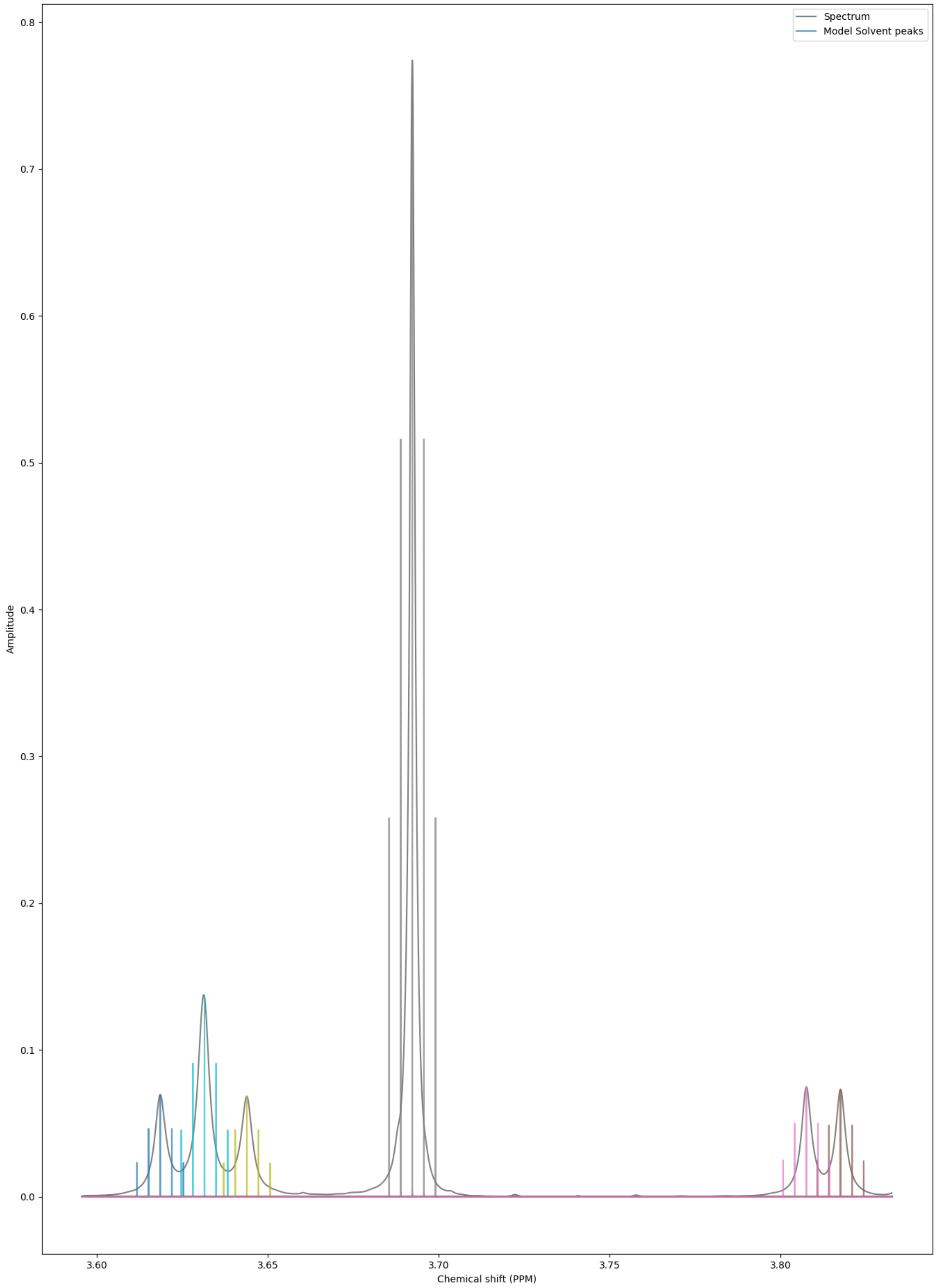

Figure 24: Figure illustrating how a model methanol multiplet is simulated centred around each picked peak in the solvent containing region of the spectrum. The simulated solvent multiplets have their amplitudes matched to the peak they are centred around.

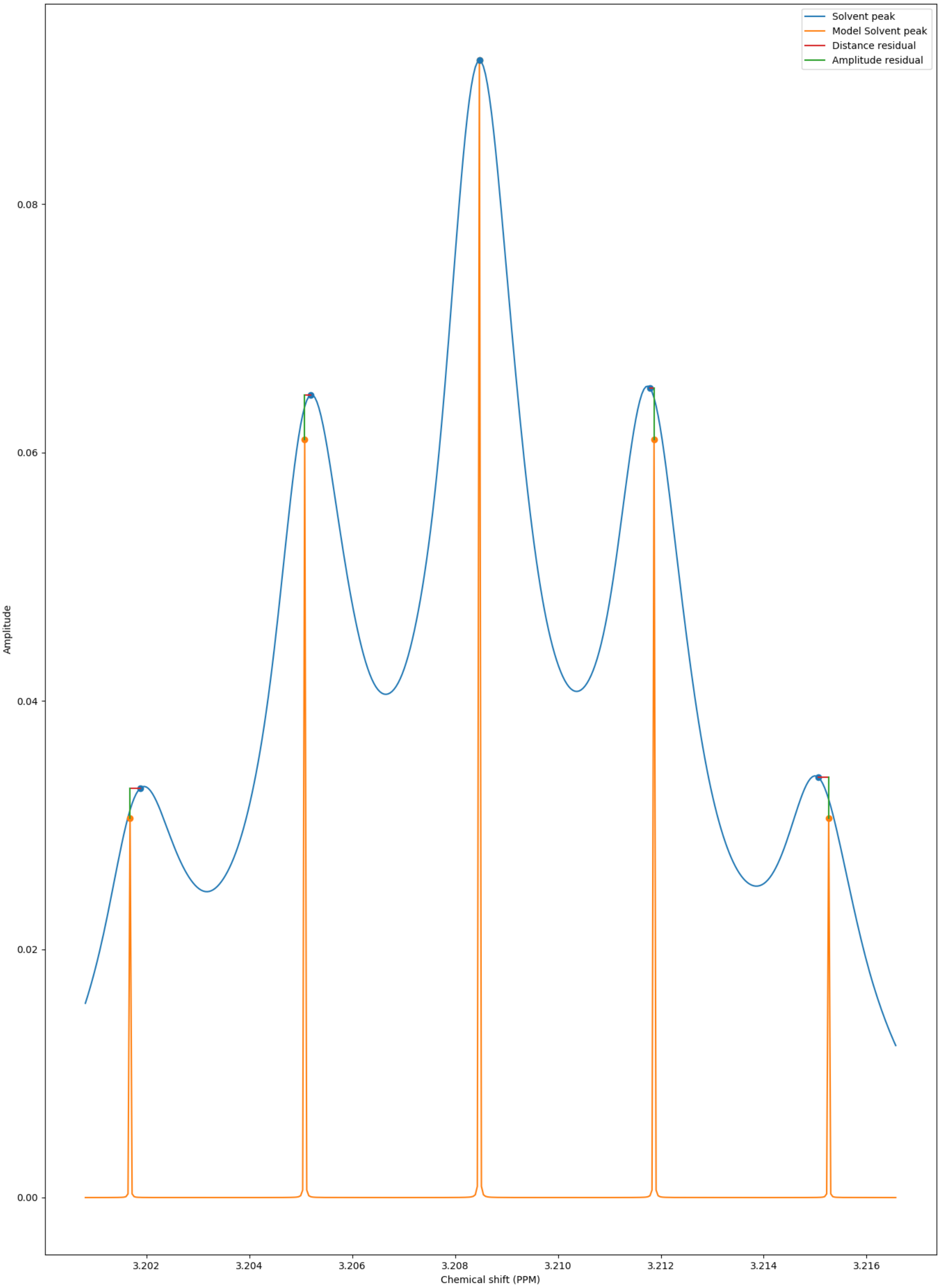

Figure 25: Figure illustrating how the amplitude and distance parameters are calculated for each picked peak in the solvent region. In this example the peak being tested is the middle of the highlighted area, the centre of the solvent multiplet has been placed on top of this peak with its amplitude to match. The amplitude residual is calculated as the sum of the green (vertical) lines and similarly the distance residual is taken as the sum of the red (horizontal lines).

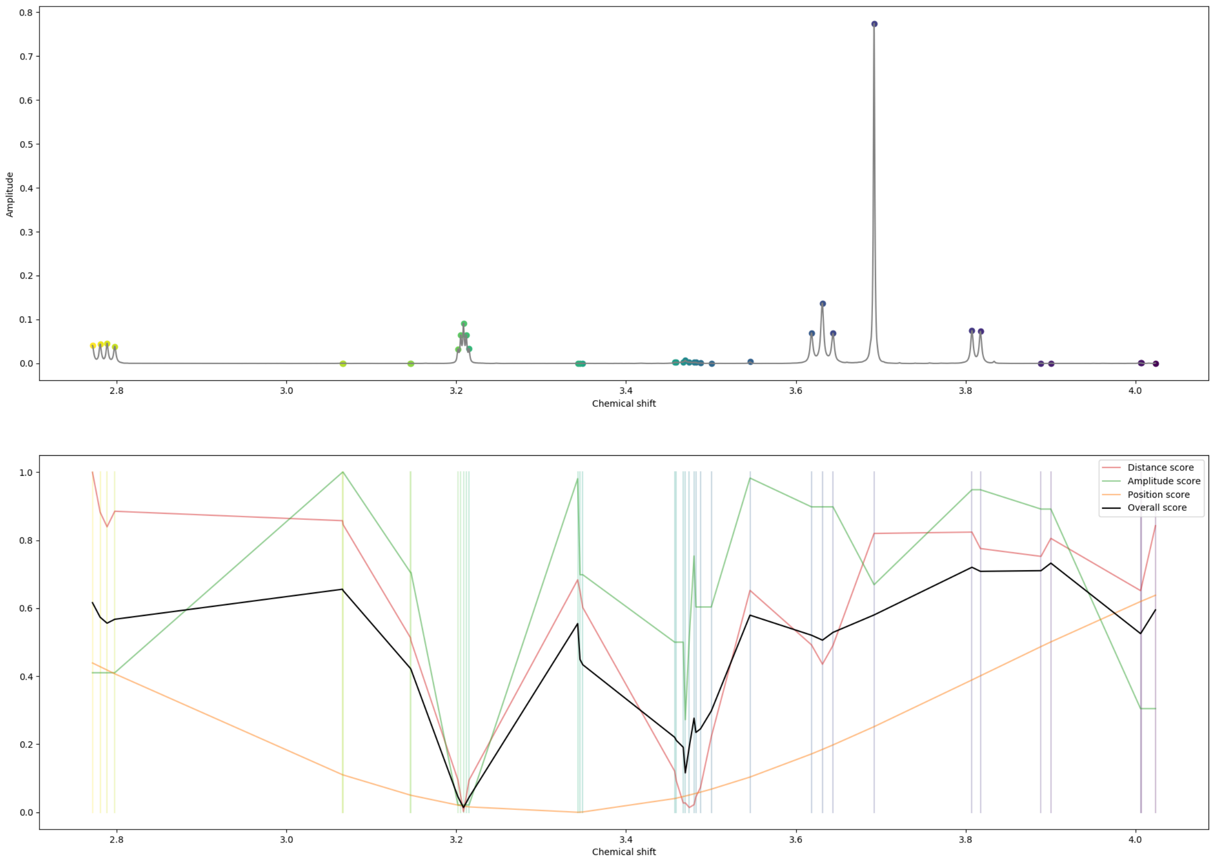

Figure 26: Figure displaying how scores are calculated for each peak in the solvent region. Top: the region of an example spectrum expected to contain a methanol solvent peak, the picked peaks tested are highlighted by the coloured markers.
Bottom: graph of the, distance, amplitude, position and overall scores for the peaks in this region. The minimum (best) score is achieved when the model curve is centred over the correct solvent peak around 3.2 ppm.

2.1.10 Integration

The algorithm for estimating k the number of protons in the spectrum has been developed from previous work in this area.[11 14] The premise of this algorithm is to iterate this constant k from the minimum possible number of protons in the spectrum to a maximum value and calculate a score based on the corresponding set of integrals. The value of k producing the highest score is taken as the constant of proportionality, and is used to normalise the integrals.

As the labile protons are the only protons that may not be observed, the minimum number of protons expected to be seen in the spectrum can be calculated by equation 13.

$$N_{\text{min}} = N_{\text{t}} - N_{\text{l}} \tag{13}$$

The minimum number of protons expected to be observed in a proton NMR spectrum. Where N_{t} is the total number of protons in the structure provided by the user and N_{l} is the number of labile protons in the structure.

The integrals are normalised for each value of k. Any multiplets with integrals < 0.5 are assumed to be impurities and removed. The remaining integrals are then used to calculate the score. This score is based on two parameters that vary from zero to one. The first decreases the further the value of k from the number of protons in the structure, as the spectra are assumed to not contain a significant number of impurities. The second score is calculated by taking the difference between each integral and its closest integer value. These differences vary from zero to one half and are combined using equation 18. The product of these parameters is taken as the score for each value of k.

The line shape used to model peaks in the spectrum is given by equation 15, this function was used as it can be easily integrated analytically to give the exact integral of the model spectrum. This value is then used to estimate the true multiplet integrals by equation 20. This method initially was developed in the field of qNMR to avoid errors produced by the truncation of peak tails and to give reliable error estimates for the integrals.[12 15]

$$GL(x(p); k, w, p0) = (1 - k)L(x) + kG(x) \tag{14}$$

$$G(x) = \frac{1 + \frac{x^2}{2}}{1 + x^2 + x^4} \tag{15}$$

$$L(x) = \frac{1}{1 + x^2} \tag{16}$$

$$x(p) = \frac{p - p0}{w} \tag{17}$$

$GL(x(p); k, w, p0)$ the generalised Lorentzian function used to construct the model spectrum. Where p denotes position in the spectrum, $p0$ is the shift value of the peak w is the half width at half maximum and k is the kurtosis parameter.

$$\text{Integer like score} = \sqrt[N]{\int_0^{d_n} \mathrm{N}(x|\mu = 0,\, \sigma = \frac{1}{16})dx_n^N} \tag{18}$$

The integral integer like score is given above. Where N is the number of peaks, $\mathrm{N}(x, \mu, \sigma)$ is a normal distribution and $\boldsymbol{d}$ is given by equation 19

$$\boldsymbol{d} = \boldsymbol{I}\%\frac{1}{2} \tag{19}$$

Figure 27: The vector I contains the integrals of the peaks in the spectrum and the vector $\boldsymbol{d}$ contains the integrals distances from integer values.

$$I_{\text{multiplet}} = k\frac{\int_0^n \boldsymbol{GL}(x(p); \boldsymbol{k}, \boldsymbol{w}, \boldsymbol{p0})dx}{\int_0^n y(x)dx} \int_b^a y(x)dx \tag{20}$$

Equation the integral of a multiplet utilising both the model and real spectrum. Where $\int_0^n \boldsymbol{GL}(x(p); \boldsymbol{k}, \boldsymbol{w}, \boldsymbol{p0})dx$ is the integral of the model spectrum, $\int_0^n y(x)dx$ is the integral of the spectrum, $\int_b^a y(x)dx$ is the integral of the real spectrum over the range of the multiplet and n is the number of points in the spectrum.

2.1.11 Peak Centre Determination

Finally the centres for each multiplet must be determined. This centre is calculated as the average chemical shift of the multiplet weighted by the intensity value at each point. This ensures that if a multiplet has been grouped together with noise around its tails, the peak centre will not be significantly affected.

2.2 Assignment Algorithm

The final part of the program assigns the atoms in each diastereomer of the molecule to observed peaks in the spectra. This assignment is made using the GIAO predicted shifts. The framework of the AA is very similar for both ^{13}C and ^{1}H spectra, this is summarised in Figures 29 and 30. The AA performs two assignments in an iterative fashion where the second assignment uses information obtained from the first assignment, the reasons for this are explained sections 2.2.1 and 2.2.2.

The core of the AA calculates $\boldsymbol{M}$ the assignment probability matrix. The elements of this matrix M_{ij} give the probability of calculated shift i corresponding to experimental peak j, if there are I calculated shifts and J experimental peaks this matrix will have dimensions $I * J$. The matrix $\boldsymbol{M}$ is then used to produce assignments.

It is necessary to define the required assignment rigorously; the most mathematically sensible definition is the assignment with highest probability as given by the elements of $\boldsymbol{M}$. It must be noted that the most chemically relevant assignment may not yield the maximum probability value, in these cases the AA may not be expected to give a correct DP4 probability. The necessity of this choice can be illustrated by considering a modest 20x20 assignment probability matrix (i.e. 20 atoms and 20 peaks). There are 20! or 2.43×10^{18} possible assignments assuming no multiple assignments. Hence, testing all the assignments is impossible. However, if the required assignment is defined as above, this problem can easily be recast into the form of a well studied problem in mathematics which describes choosing the minimum possible sum of elements in a matrix using each row and column only once. An algorithmic solution to this problem referred to as the Hungarian method was published in 1955 based on the earlier work of two Hungarian mathematicians.[16] This algorithm is available within the Scipy Python package and has been implemented in the AA to find the most probable assignment given by the matrix $\boldsymbol{M}$. This process is summarised pictorially in Figure 28.

The matrix $\boldsymbol{M}$ is given by equation 21. The terms in this equation are described in sections 2.2.1 to 2.2.3.

$$M_{ij} = (P_{ij}(D_{ij}) * A_j)^{\frac{1}{2}} \tag{21}$$

The overall equation for assignment matrix $\boldsymbol{M}$ used in the carbon AA. If there are I calculated shifts and J experimental peaks this matrix will have dimensions $I * J$. The rows correspond to calculated shifts and the columns to experimental peaks. This matrix is calculated by multiplying each row of the position probability matrix $\boldsymbol{P}$ by the corresponding amplitude weight A_j raised to the power of the corresponding element of the bias vector B_i and taking the square root

$$M_{ij} = P_{ij}(D_{ij}) \tag{22}$$

Overall equation for assignment matrix $\boldsymbol{M}$ used in the proton AA.

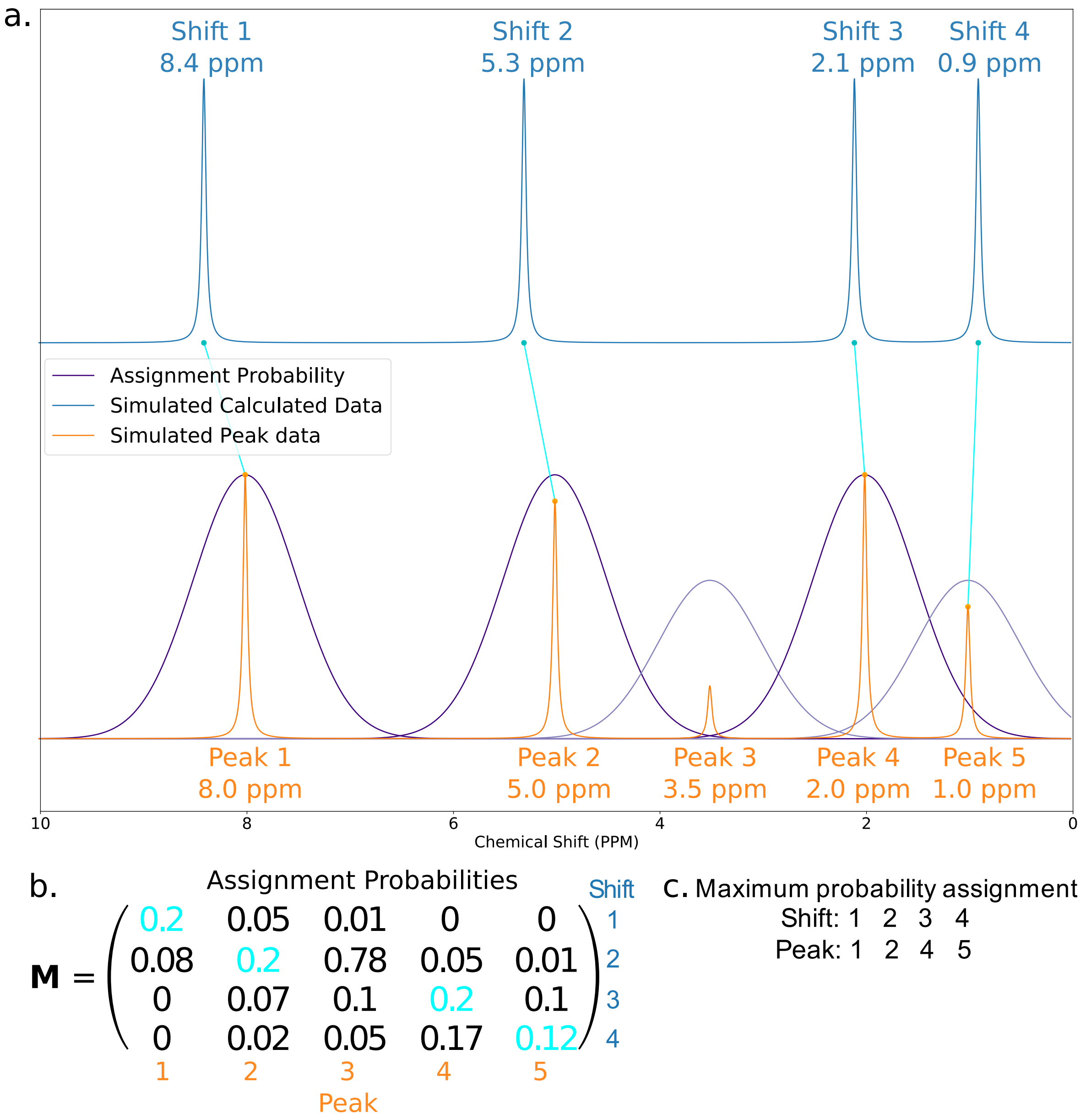

Figure 28: Figure illustrating using simulated data how calculated shifts can be assigned to experimental peaks using the assignment probability matrix $\boldsymbol{M}$ a) The peaks in the simulated calculated spectrum (blue) are assigned to those in the experimental spectrum (orange). The assignment probability (purple) varies with the amplitude of the experimental peaks and their relative distances from the peaks in the calculated spectrum. b) The matrix $\boldsymbol{M}$ is calculated and the final assignment (highlighted in cyan) found using the Hungarian Algorithm. The columns of the matrix $\boldsymbol{M}$ correspond to the experimental peaks and the rows to the calculated shifts. Hence, $M_i j$ corresponds to the probability of calculated shift i corresponding to experimental peak j. c) The final assignment found in this example.

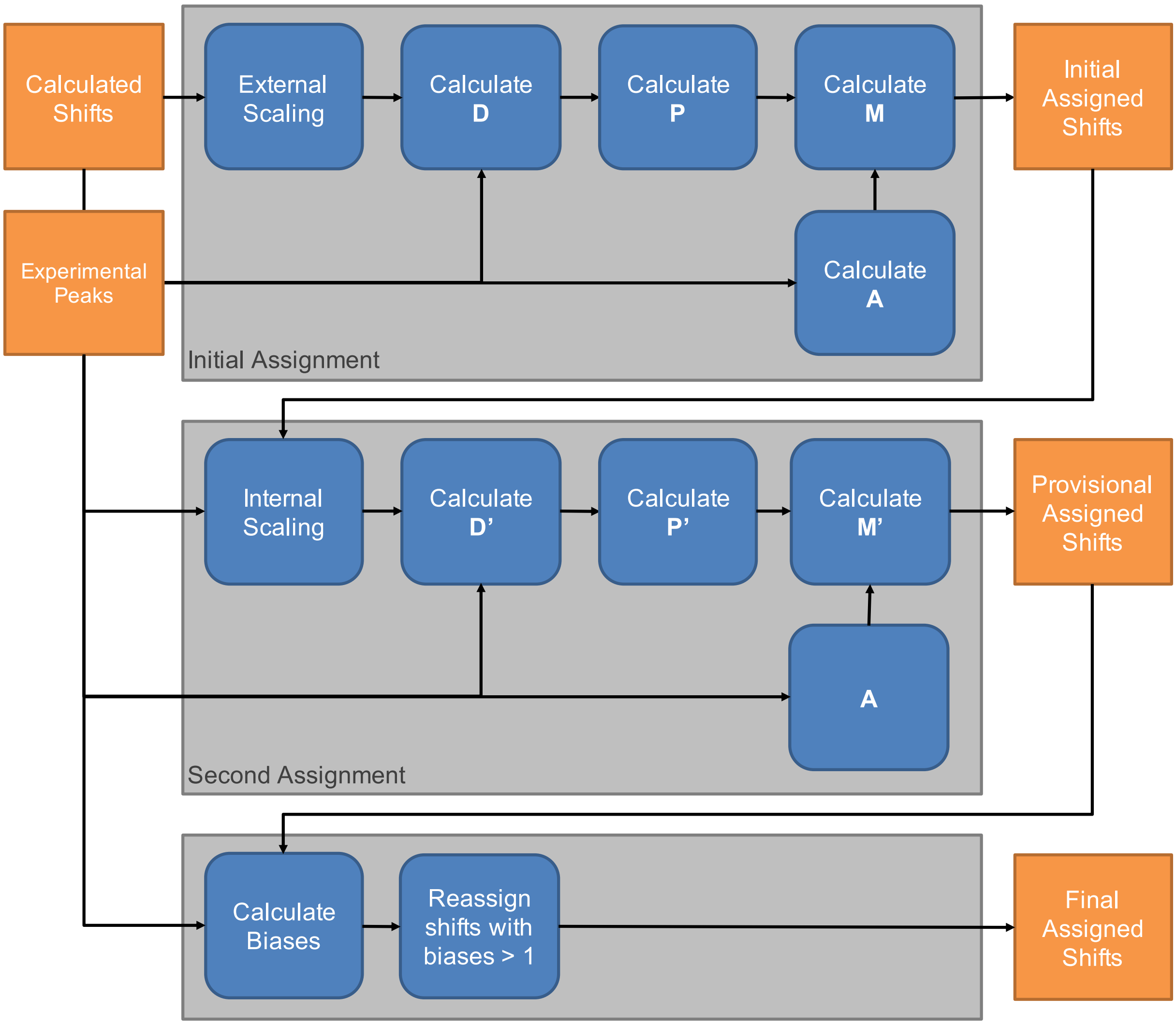

Figure 29: Figure illustrating the framework of the ^{13}C assignment algorithm. The AA takes in calculated shifts and experimental peak locations. Initially the calculated shifts are scaled using external scaling factors prior to calculation of $\boldsymbol{M}$ and assignment. Once this initial assignment has been made, it is used to calculate internal scaling factors for rescaling of the calculated shifts and bias vector (section 2.2.2) prior to the final assignment.

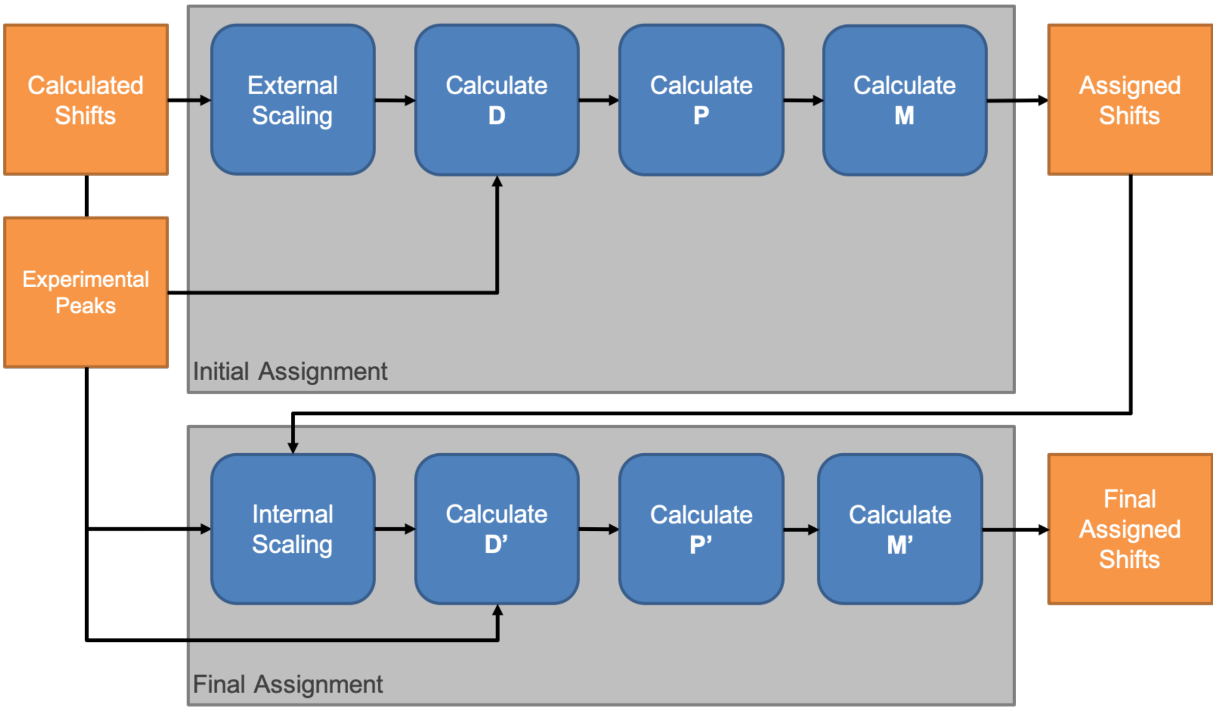

Figure 30: Figure illustrating the framework of the ^{1}H assignment algorithm. The AA takes in calculated shifts and experimental peak locations. Initially the calculated shifts are scaled using external scaling factors prior to calculation of $\boldsymbol{M}$ and assignment. Once this initial assignment has been made, this is used to calculate internal scaling factors for rescaling of the calculated shifts prior to the final assignment.

2.2.1 Prediction Error Statistical Models

The equations 21 and 22 for $\boldsymbol{M}$ contain the term $\boldsymbol{P}(\boldsymbol{D})$, the prediction error probability matrix. $\boldsymbol{D}$ is the difference matrix, its elements D_{ij} describe the difference between the calculated shift for atom i and shift of experimental peak j. The elements of $\boldsymbol{P}(\boldsymbol{D})$, P_{ij}, describe the probability of observing an error of D_{ij} between the corresponding calculated and experimental values.

To calculate $\boldsymbol{P}(\boldsymbol{D})$ a model describing the distribution of prediction errors is required. A number of different models describing the external prediction error distribution were tested. A simple single Gaussian model was found to perform well and has been incorporated into the AA. The mean and standard deviation of this single Gaussian model were estimated by fitting these parameters to an empirical probability density function describing a dataset of known prediction errors produced in a previous DP4 study.[17]

The prediction error probabilities cannot be found using the calculated shift values directly. It has been previously noted that GIAO shift predictions are subject to systematic errors that vary depending on position within the spectrum.[18][17] Plotting experimental peaks against calculated shifts for the mPW1PW91 functional, a clear linear trend is observed as shown in Figure 31. However, the gradient and intercept of this line are not one and zero respectively.

To mitigate this issue, as shown in Figures 29 and 30, the assignment process is performed in two stages. In the first stage of assignment, an external linear scaling method is employed using parameters estimated from the dataset of known prediction errors mention above. For example, carbon GIAO predicted shifts calculated using the mPW1PW91 functional and 6-311g(d) basis set are scaled using equation 23. Once the calculated shifts have been scaled, $\boldsymbol{D}$ is calculated by equation 24 and $\boldsymbol{P}$ by equation 25.

Once the initial assignment has been completed the assigned shifts and peaks are used to calculate internal linear scaling factors in a similar fashion to DP4. The calculated shifts are then rescaled and the assignment process repeated.

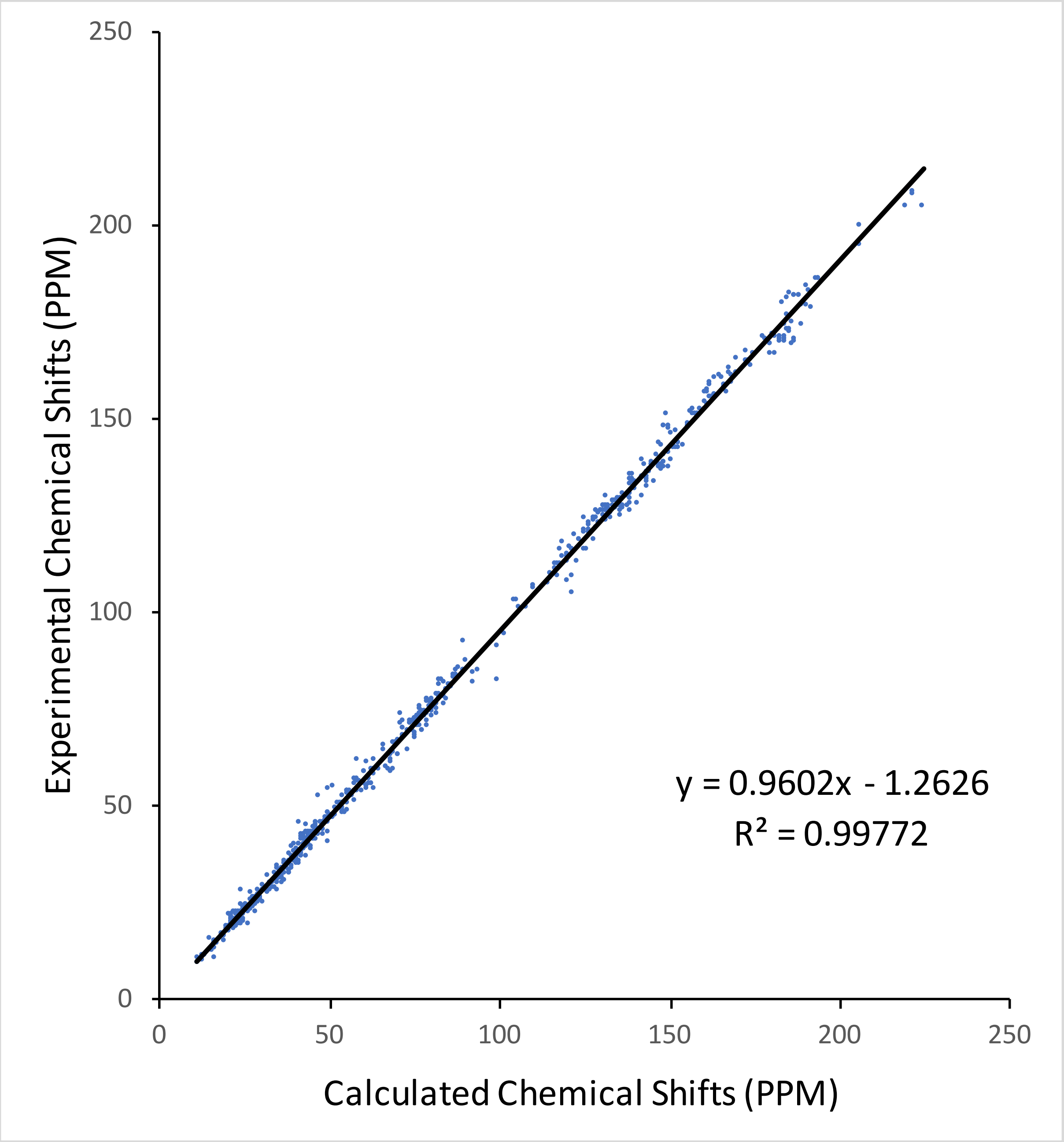

Figure 31: A graph to show the variation in experimental proton chemical shift values with their corresponding GIAO predicted values using the mPW1PW91 functional, 6-311g(d) basis set and PCM solvent model.

$$\boldsymbol{\sigma}_{\textbf{scaled}} = 0.9602\boldsymbol{\sigma}_{\textbf{calculated}} - 1.2626 \tag{23}$$

GIAO carbon shifts calculated using the mPW1PW91 functional and 6-311g(d) basis set are scaled using the above equation.

$$D_{ij} = |\sigma_{\text{scaled calc},i} - \sigma_{\exp,j}| \tag{24}$$

The difference matrix $\boldsymbol{D}$ is calculated by the above equation. Where $\sigma_{\text{scaled calc},i}$ is the scaled calculated shift i and $\sigma_{\exp,j}$ is experimental peak j.

$$P_{ij} = N(D_{ij} \,|\, \sigma,\, \mu) \tag{25}$$

The position probability matrix $\boldsymbol{P}$ is calculated by the above equation. The element P_{ij} is calculated from D_{ij} using a normal probability density function with standard deviation σ and mean μ which change depending on the type of spectrum and computational conditions used.

2.2.2 Amplitude Statistical Model

As shown in Figure 29 the ^{13}C AA has an additional amplitude weighting stage. This is also shown in equation 21 by the term A_j. This has been incorporated for a number of reasons.

The first reason for including this stage is to prioritise the assignment of intense peaks over lower intensity peaks that are more likely to be noise. A number of different amplitude weighting functions such as, logistic and sigmoidal functions were investigated. The amplitude weighting system incorporated into the final program is described below.

A kernel density estimation[19] (KDE) is performed to estimate the probability distribution function of the experimental peak amplitudes, as shown in Figure 32. The peaks are then divided into groups, the group boundaries are set as the minima in the second derivative of the probability distribution function.

The cumulative sum of peaks above each groups lower boundary is calculated, the weight assigned to each group is the number of carbon atoms in the structure divided by this value. The weights are then normalised to fix the largest weight to one. This is summarised in Figure 32.

Figure 29 illustrates how the ^{13}C AA performs the assignment in three stages. The overall probability of an experimental peak calculated shift pair is given by combining the position based probabilities and the amplitude based probabilities as shown in equation 21. However, this does not take into account how each piece of information should be weighted, this is illustrated in Figure 33. This weighting should consider the distribution of peak intensities and positions in the local environment around each calculated shift, the third round of assignment addresses this problem.

After the provisional assignment has been made, the unassigned peaks within 10ppm of the experimental peak assigned to each calculated shift are analysed (including potential multiple assignments of already assigned peaks). The bias for calculated shift i is given by equation 26. Any calculated shifts with biases above one are reassigned. This reassignment is performed by simply sorting these shifts in descending order of bias and assigning them in the order to available peaks within 10 ppm in descending order of amplitude. The cases where this function is useful is described in figure 33. Finally a simple function is utilised to remove any cross assignments that have been made by the AA, this helps prevent the system from assigning calculated shifts that fit the experimental data poorly to very distant experimental peaks.

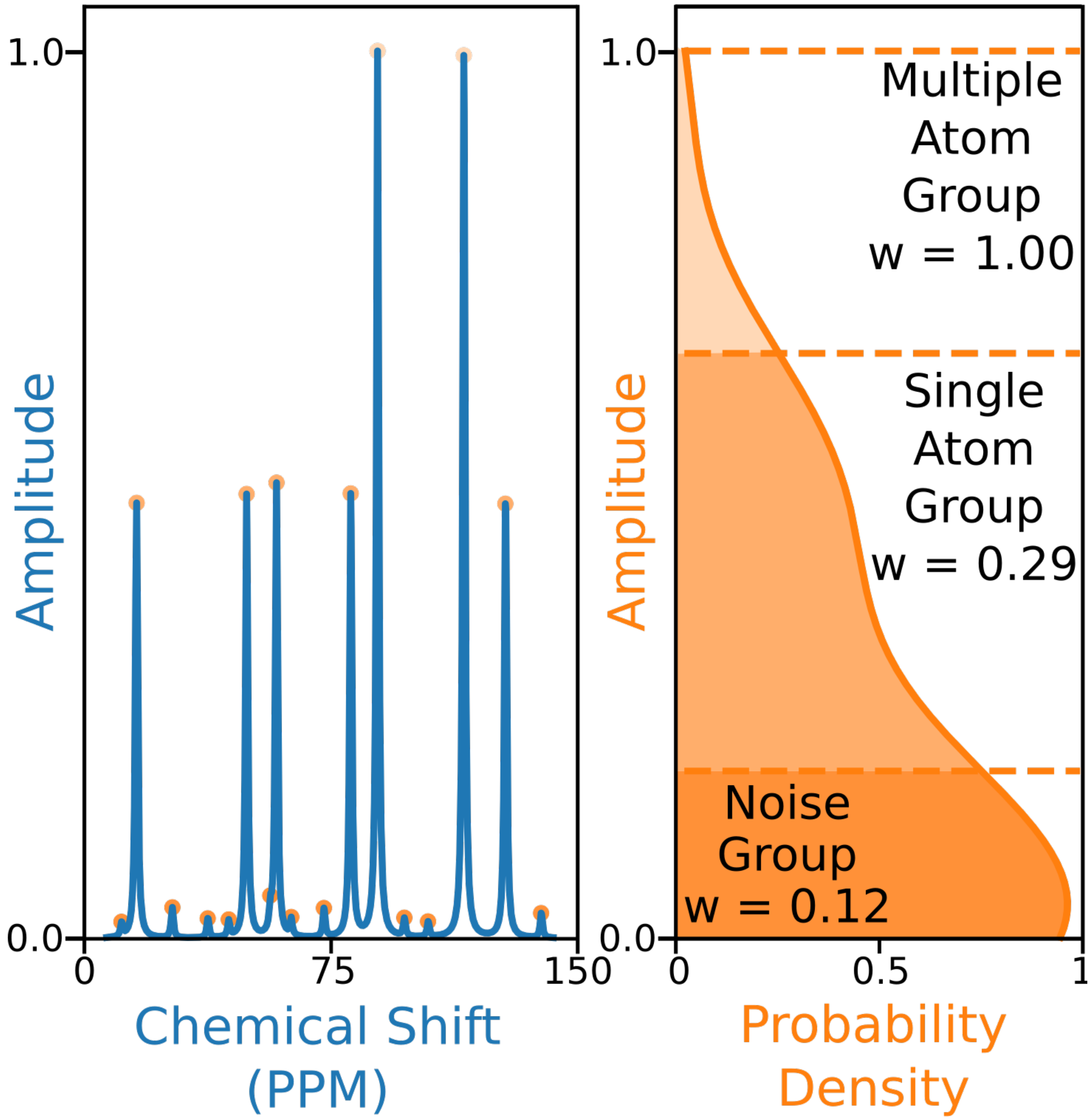

Figure 32: Peaks (left) are grouped by amplitude, depending on the minima in the second derivative of the amplitude probability density func-tion (right) they fall between (dashed lines). In this simulated example, the number of carbon atoms in the structure is nine. The cumulative sum of peaks above each groups lower boundary is calculated, the weight assigned to each group is the number of carbon atoms in the structure divided by this value. The weights are then normalized to fix the largest weight to one.

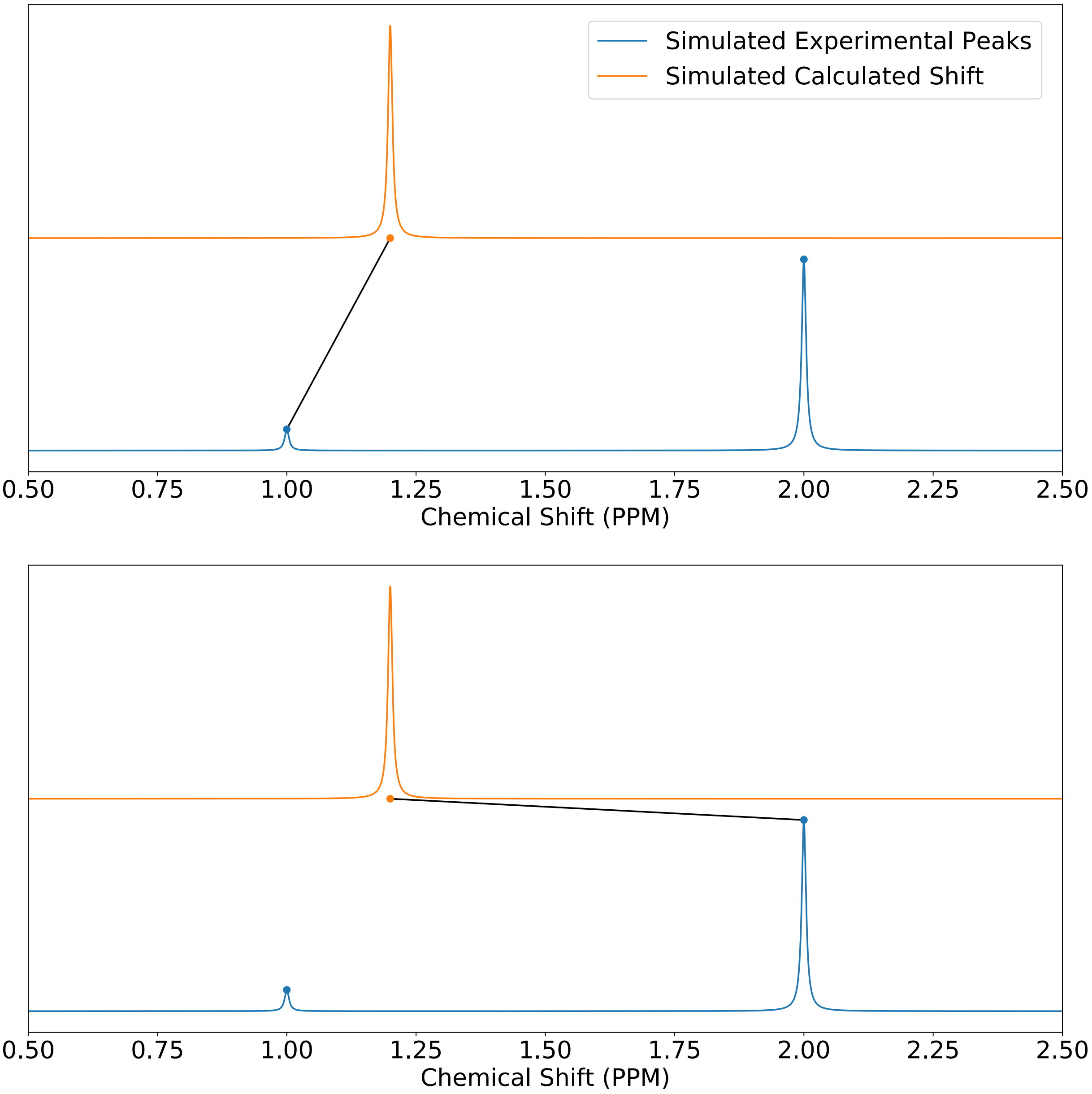

Figure 33: Figure illustrating how an assignment may change depending on the bias towards amplitude or position probabilities. On the left the assignment has been made with a bias towards position. Whilst on the right, the assignment has been made with a bias towards amplitude. The assignment that is preferred by this system depends on the distribution of peaks around each calculated shift.

$$B_i = \begin{cases} \frac{\max(\boldsymbol{A'^i})}{A^i_{\text{assigned}}}, & \text{if } > 1 \\ 1, & \text{otherwise} \end{cases} \tag{26}$$

The bias for shift i is given above. Where $\boldsymbol{A'^i}$ is a vector containing the amplitude weights of all unassigned peaks within 10ppm of the peak assigned to calculated shift i and A^i_{assigned} is the amplitude weight of the peak assigned to calculated shift i

2.2.3 Multiple Assignment Penalty

In ^{13}C spectra it is not always the case that there are as many experimental peaks as carbon atoms in the molecule, due to both equivalent carbons and non equivalent carbons in very similar environments. Similarly, the GIAO shift predictions may not reflect the degeneracy seen in the spectrum. It was found that allowing the AA additional flexibility to assign peaks in the ^{13}C spectrum multiple times, using a multiple assignment penalty system was beneficial.

The multiple assignment penalty used is given by equation 27, this equation illustrates how the multiple assignment penalty becomes more severe as a peak is assigned more and more times. In addition, this equation also priorities the multiple assignment of peaks determined by the KDE grouping (section 2.2.2) that likely correspond to multiple carbon atoms.

$$\text{penalty}_i = \frac{1}{8}^{(k_i)(t_i)} \tag{27}$$

The multiple assignment penalty for peak i is given by the above equation. The value of k_i depends on the KDE group peak i is in. A value of $k = 1$ is given to the group containing the most intense peaks, then $k = 2$ to the group with the second most intense peaks and so on. The value of t represents the number of times the peak has already been assigned.

3 Results and Discussion

3.1 Results

In order to evaluate the performance of NMR-AI a test set of 47 molecules with a diverse range of NP like carbon skeletons with between 2 and 32 diastereomers was constructed, shown in figure 34. This test set includes a number of molecules with challenging properties for both the AA and DP4 including, spectra with very low signal to noise ratios, impure spectra, molecules with multiple rotamers, very flexible molecules and molecules containing heavy elements such as iodine. These spectra have been taken in a range of solvents with a range of dipole moments providing an additional challenge. Using this test set will produce a very realistic and reliable evaluation of NMR-AI performance when compared to a more realisitic test set containing many small rigid molecules with densely packed stereocentres.

To determine the efficacy of DP4-AI, the performance of the program was compared with the previous minimum user input option in PyDP4. This benchmark is referred to here as the pairwise AA. When using PyDP4, the user must provide a description of either the molecules ^{13}C NMR spectrum, ^{1}H NMR spectrum or both. A full assignment of the molecule does not have to be provided, only the locations of the peaks and their corresponding integrals which the user must obtain by processing and analysing the NMR spectrum manually. With this information, PyDP4 assigns the atoms in the molecule to experimental peaks by pairing the calculated shifts to experimental peaks in the NMR description in order of chemical shift. All of the spectra in the test set were processed and peak picked manually to allow for DP4 probabilities to be calculated using the pairwise AA.

Both NMR-AI and the pairwise AA were tested with DFT calculations being performed at three levels of theory, these are summarised in figure 35

A statistical model describing the DFT NMR shift prediction error probabilities is also required to perform a DP4 calculation. There are a large number of potential forms this statistical model can take, these have been investigated in a previous study.[17] In this study four statistical models were tested at each level of theory, these included, a single region single gaussian model fitted to an external dataset of prediction errors (external models), three single region models with, one two and three gaussian functions (respectively) fitted to the prediction error distribution observed in this test set (internal models). In order to ensure no overfitting was being performed, a cross validation statistical model was also tested for the two highest levels of theory. In this model, molecules with similar frameworks were divided into groups (illustrated in 34 by the initials) for each of these groups a separate 3 gaussian internal model was fitted to the prediction error distribution of the test set excluding the molecules in the group (leave one out style).

The a break down of the results from analysis are presented in figures 36, 37, 38 and 39. These results are discussed in the next section.

3.2 Discussion

Figure 38 displays some interesting trends to be analysed. As the level of theory is increased in the DFT calculations is increased there is a corresponding increase in the correct prediction rate of both DP4-AI and and pairwise AA. This trend can be rationalised as the level of theory is increased, the NMR shift predictions become more accurate and thus the DP4 probabilities become more representative. Moreover, the DP4-AI results show a greater increase in correct prediction rate with increasing level of theory, this occurs as both the accuracy of the assignment and the DP4 calculation are dependent on the accuracy of the NMR shift calculations.

There is also a slight increase in performance when increasing the number of parameters in the statistical model used. This is also the expected trend, as the number of parameters increases the statistical model used will more closely fit the empirical prediction error distribution, hence the calculated probabilities will become more accurate.

As DP4-AI has been designed to replicate the pairwise assignment as closely as possible, DP4-AI can only perform as well as its pairwise counterpart. Hence the pairwise AA represents the upper limit of DP4-AIs performance. In the limit of perfect NMR shift calculations DP4-AI and the pairwise AA would be expected to give the same result.

The only situations where it is possible for DP4-AI to outperform the pairwise AA are when mistakes have been incorporated into the NMR description provided by the user such as, including an impurity peak or incorrectly estimating the integral of a peak. The NMR descriptions have been written to minimise these cases as far as possible.

In contrast there are a number of situations where the pairwise AA may be expected to outperform DP4-AI. These situations occur when additional intuition is required when writing the NMR description that has not been explicitly given the DP4-AI. These include spectra with large number of impurities, low signal to noise ratios and spectra containing mixtures of compounds/diastereomers/rotamers. These situations may be tackled in the future by adding explicit treatment for mixtures and impurities in DP4-AI.

Therefore the final correct prediction rate of 160% above that of chance (for the highest level of theory combined with the cross validation 3 gaussian statistical model) is an excellent result, indicating DP4-AI has hit the current upper limit of its performance. The probability of correctly assigning the relative stereochemistry of at least as many molecules in this dataset as DP4-AI by chance is $2.1 * 10^{-8} \pm 1 * 10^{-9}$. This shows DP4-AI is very reliably performing better than chance (see figure 40).

The highest performance of the pairwise AA, represents a correct prediction rate170% above that of chance. This is a very high performance given the very challenging nature of the evaluation test set of molecules. This dataset clearly indicates there are a number of ways in which this performance can be improved.

Conformational selection is very important in calculation of DP4. In order to calculate the chemical shifts for a diastereomer of a molecule, first a conformational search must be performed. This conformational search will identify all low energy conformers within a specific energy window, it is assumed that these conformers will accurately represent the ensemble of conformations that the molecule will adopt in solution. Chemical shift values and single point energies are calculated for each conformer, the chemical shift values are then Boltzmann weighted using the single point energies to produce overall chemical shifts for the diastereomer. The way in which the conformers are selected is very important for two reasons. The first is that the conformers selected must very accurately represent the ensemble of conformers adopted in solution in order to produce accurate chemical shift values. The second reason is the set of conformers chosen to represent the ensemble of conformations must be as small as possible in order to minimise the computational cost/time of performing a DP4 calculation. By developing new conformational searching, clustering and pruning techniques, the chemical shift values may be improved.

The difficulties of conformational searching and selection are particularly apparent for flexible molecules as these can adopt a wide range of conformations in solution. It may be beneficial in the future to develop new methods specifically for the treatment of flexible regions of molecules in order to improve the accuracy of shift prediction.

Another related way in which the chemical shift prediction may be improved is better treatment of the solvent in the system. PyDP4 uses a PCM implicit solvent model in its calculation. Whilst implicit solvent models are useful as they are more computationally efficient than explicit solvent methods, the later provide a more accurate representation of the system being analysed. This is true for a number of reasons, using explicit solvent models may give a more accurate representation of intramolecular forces in the molecule such as intramolecular hydrogen bonds. In addition, using explicit solvent may produce a more realistic set of conformers to represent the ensemble of conformers the molecule adopts. There are a number of difficulties in using explicit solvent that must be overcome. For example, the computational time in DFT calculations becomes much larger when dealing with solvent molecules explicitly as the number of electrons in the system increases. Moreover, the conformational flexibility of the system also dramatically increases. The solvent molecules can orient in many ways with respect to the molecule, leading to a large number of conformers with very different solvent placements but very similar molecular conformations. This may lead to additional challenges in conformational searching and also conformational selection.

A number of the spectra display mixtures of compounds, an interesting example of this can been seen in the spectra of molecule IP2. The amide bond in this molecule leads to two sets of rotamers clearly being present in the spectra (see section 4, figure 83). As a result the calculated chemical shift values are not expected to be accurate as the conformers no longer follow a Boltzmann distribution. Despite this, DP4-AI still produces an accurate assignment of the molecule, assigning only the peaks for the major

rotamers in the spectrum. This displays DP4-AI in some cases can already assign spectra of mixtures, this functionality will be developed further in future releases of DP4-AI.

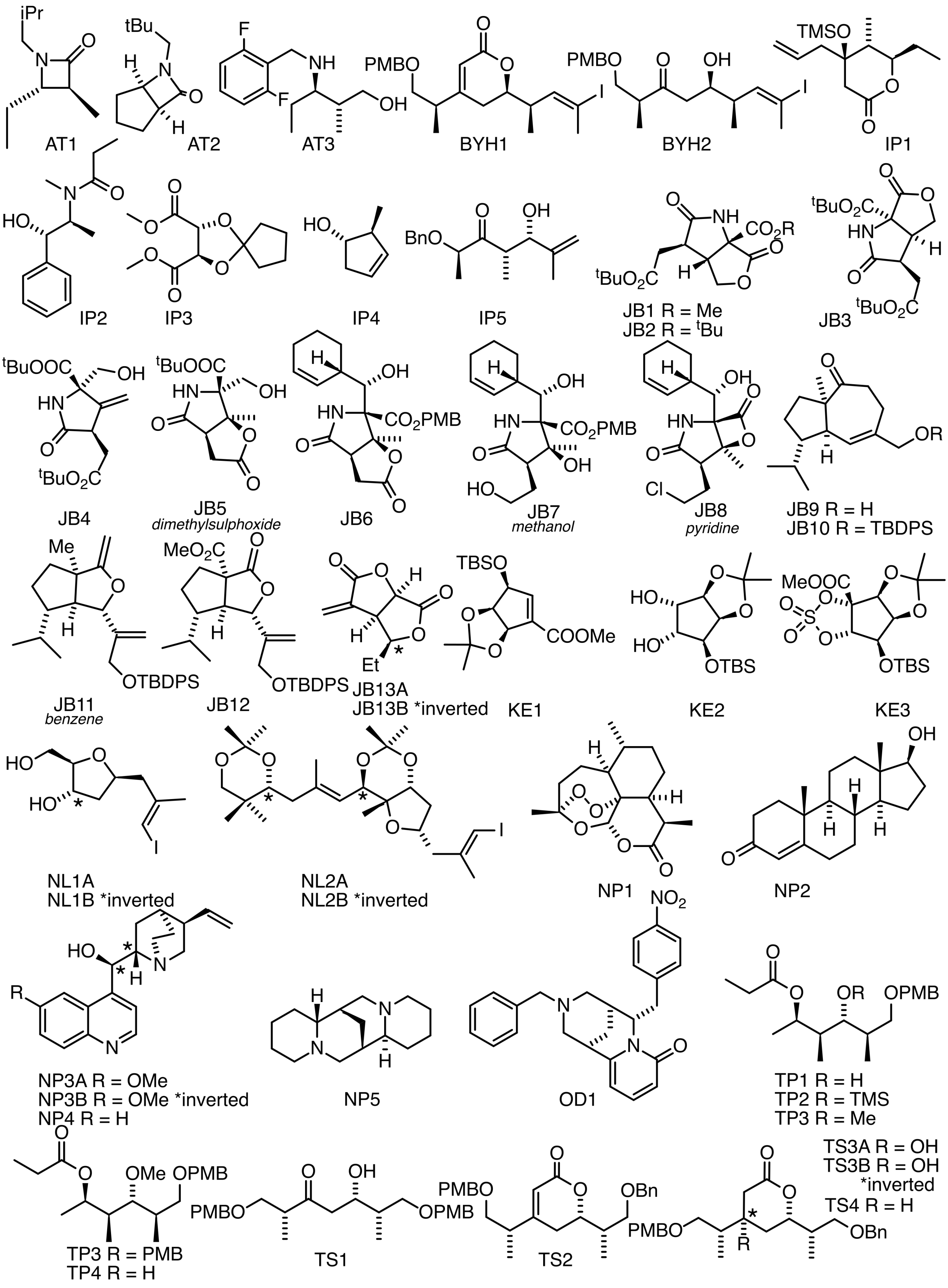

Figure 34: Figure illustrating the 47 molecules utilised to evaluate the performance of the AA. Molecules, AT3, TS3A, TS4 and NL1A only have corresponding 1H NMR data, all other molecules have both 1H and 13C NMR data. The spectra for molecules JB7, JB11, JB5 and JB8 were taken in solvents methanol, benzene, DMSO and methanol respectively, whilst all others were taken in $CDCl_3$. Sources for the spectral data: AT1-3,[20,21]BYH1-2,[22] JB1-13B,[23,24]KE1-3 (personal correspondence),NL1A-2B,[25] TP1-4 (personal correspondence), TS1-4(personal correspondence), data for all other molecules has been collected specifically for this study.

Conformer Geometries	DFT NMR Calculation	Single point energy calculation
MM	mPW1PW91/6-311gd **	
DFT optimized B3LYP/6-31gd	mPW1PW91/6-311gd **	
DFT optimized B3LYP/6-31gd	mPW1PW91/6-311gd **	M062x/Def2TZVP

Figure 35: Table describing the three levels of theory NMR-AI and the pairwise AA were evaluated at. **Def2SVP was used for molecules containing iodine

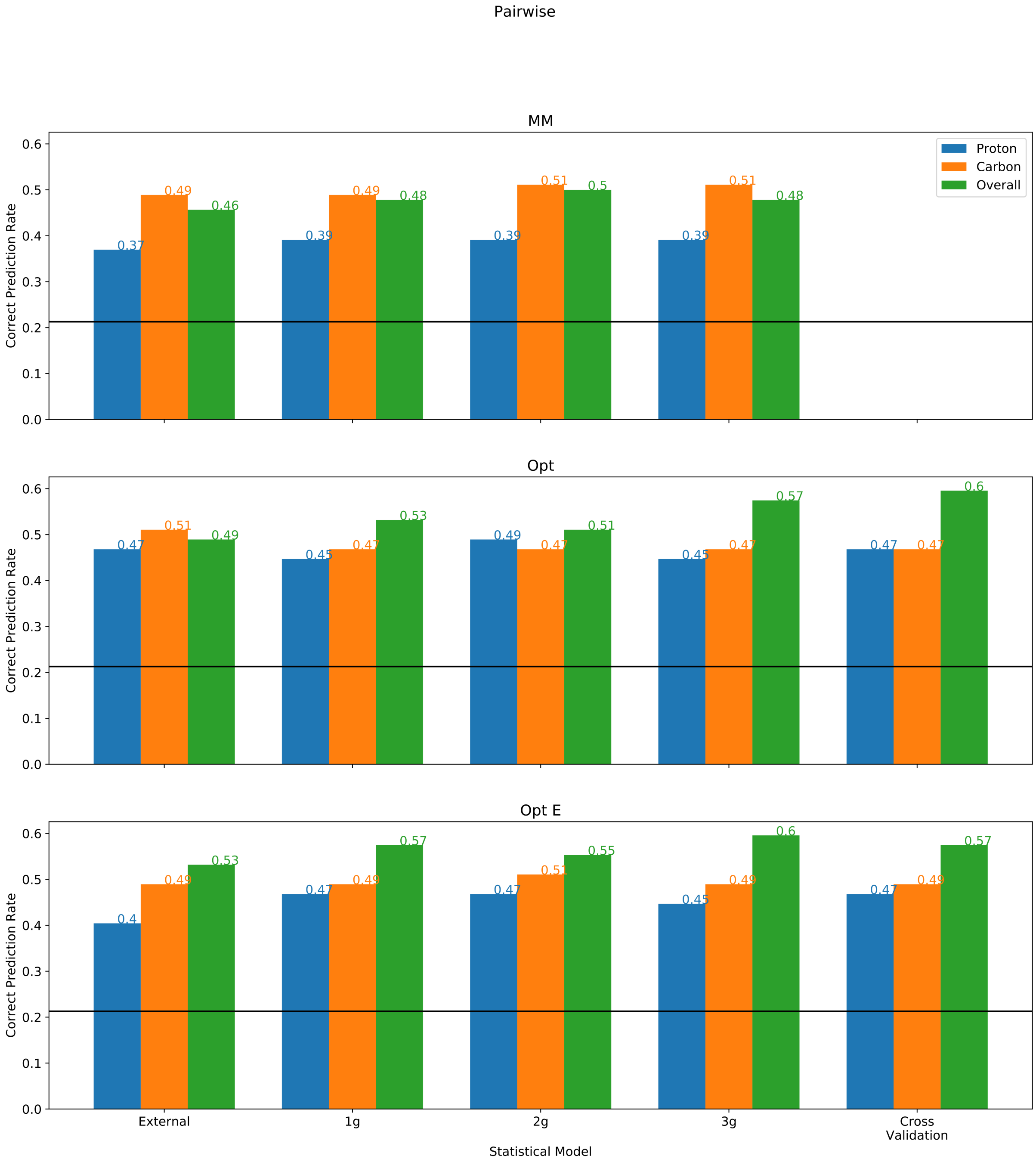

Figure 36: The correct stereochemistry prediction rates for the pairwise AA for each of the 14 combinations of level of theory and statistical model tested. The Correct prediction rates are expressed as the percentage of the molecules in the dataset the algortihm has correctly assigned. Correct prediction rates are quoted for DP4 calculations made utilising ^{13}C data only (orange), ^{1}H data only (blue) and combined $^{13}C + {}^{1}H$ data.

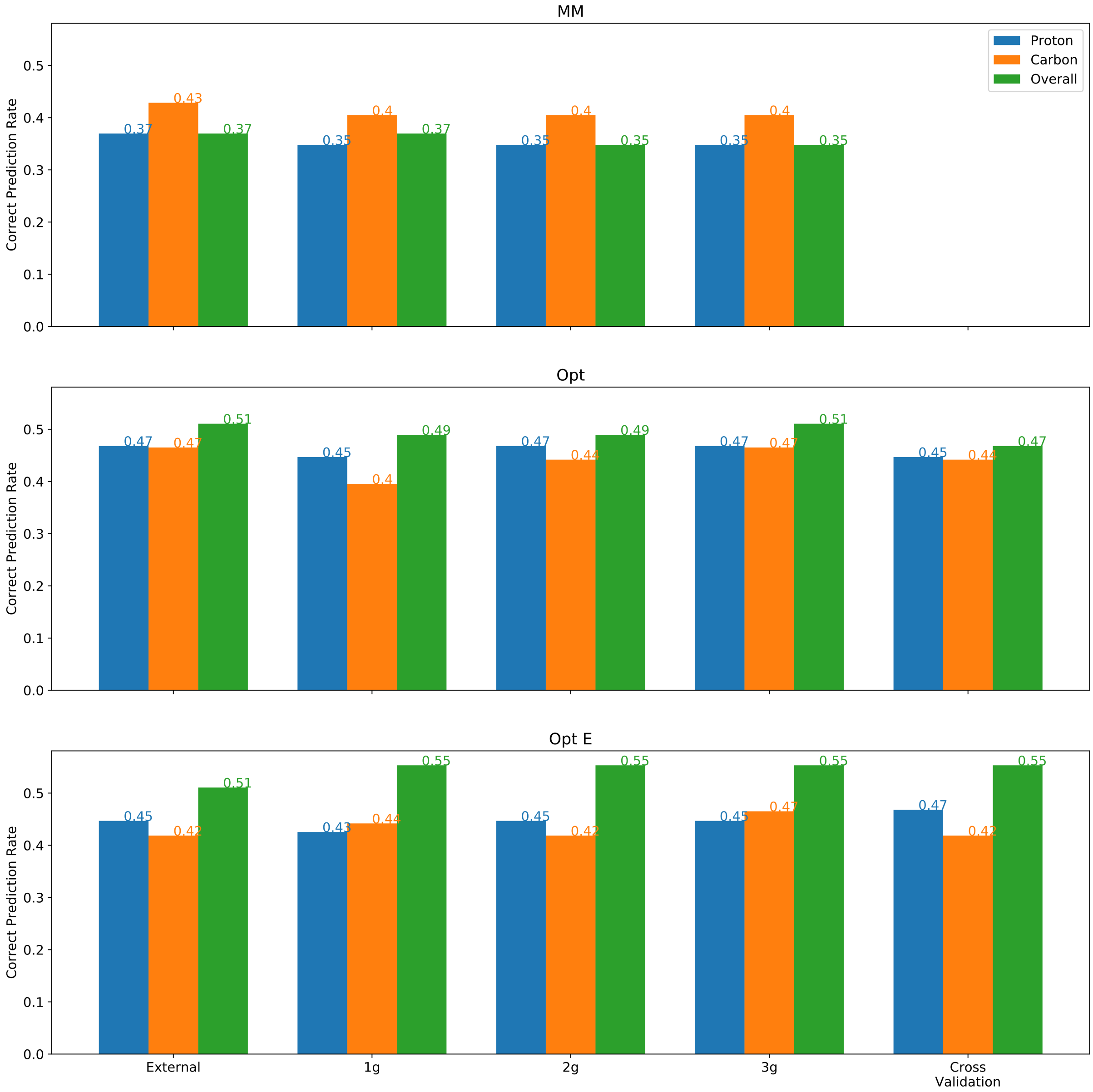

Figure 37: The correct stereochemistry prediction rates for the pairwise AA for each of the 14 combinations of level of theory and statistical model tested. The Correct prediction rates are expressed as the percentage of the molecules in the dataset the algortihm has correctly assigned. Correct prediction rates are quoted for DP4 calculations made utilising ^{13}C data only (orange), ^{1}H data only (blue) and combined ^{13}C + ^{1}H data.

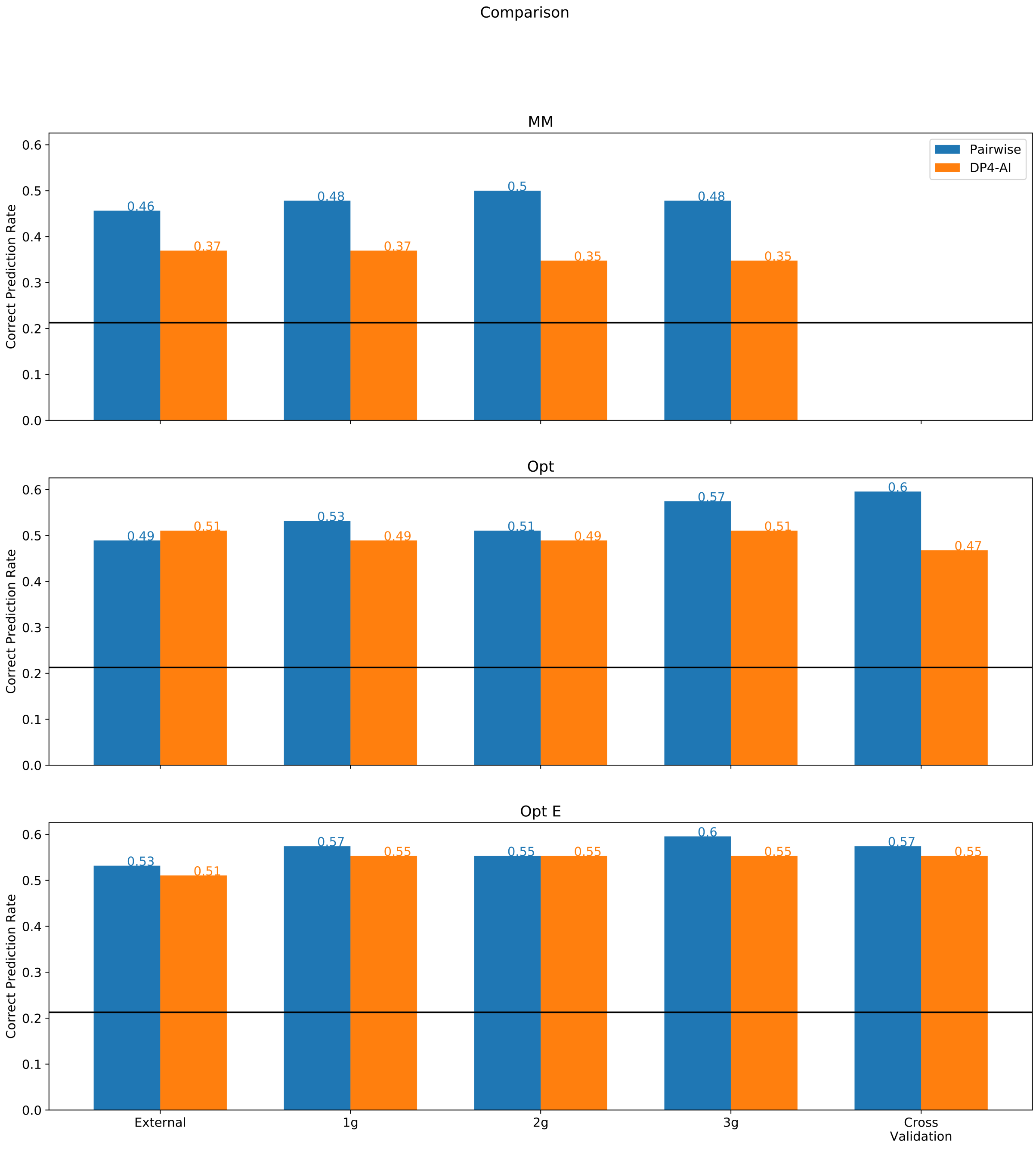

Figure 38: A side by side comparison of the correct stereochemistry prediction rates for DP4-AI and the pairwise AA for each of the 14 combinations of level of theory and model tested.

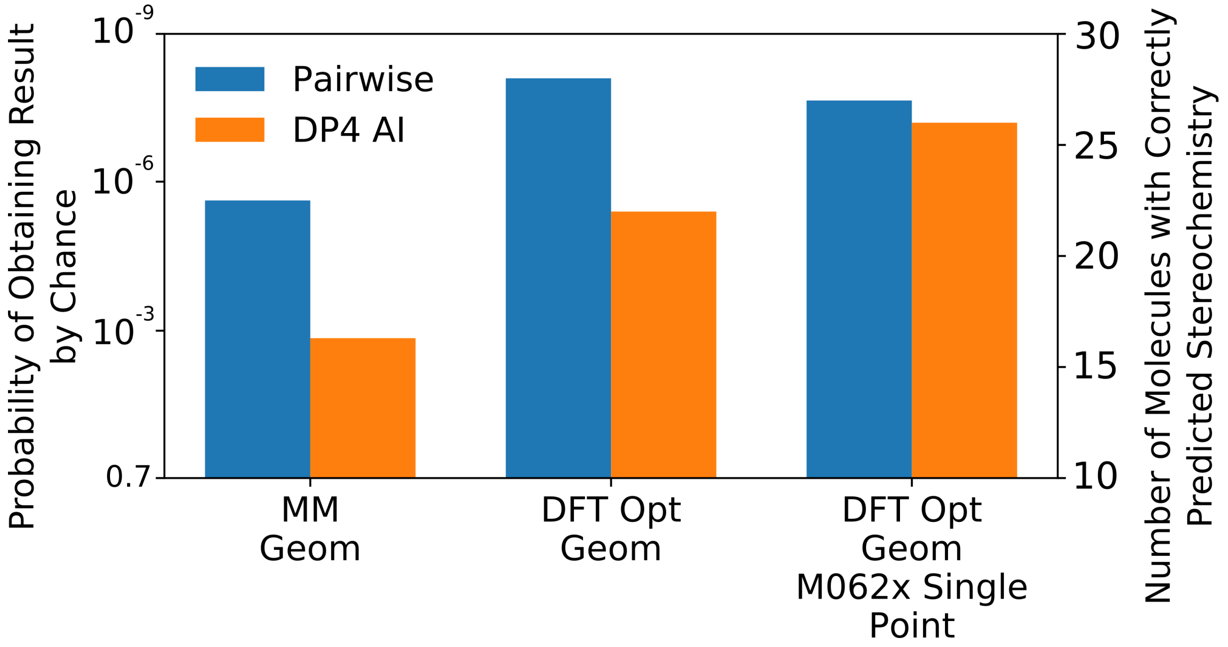

Figure 39: A side by side comparison of the correct stereochemistry prediction rates for DP4-AI and the pairwise AA at the highest level of theory tested and the 3 gaussian cross validation statistical model. The correct prediction rates are expressed as a percentage of the correct prediction rate expected for random predictions.

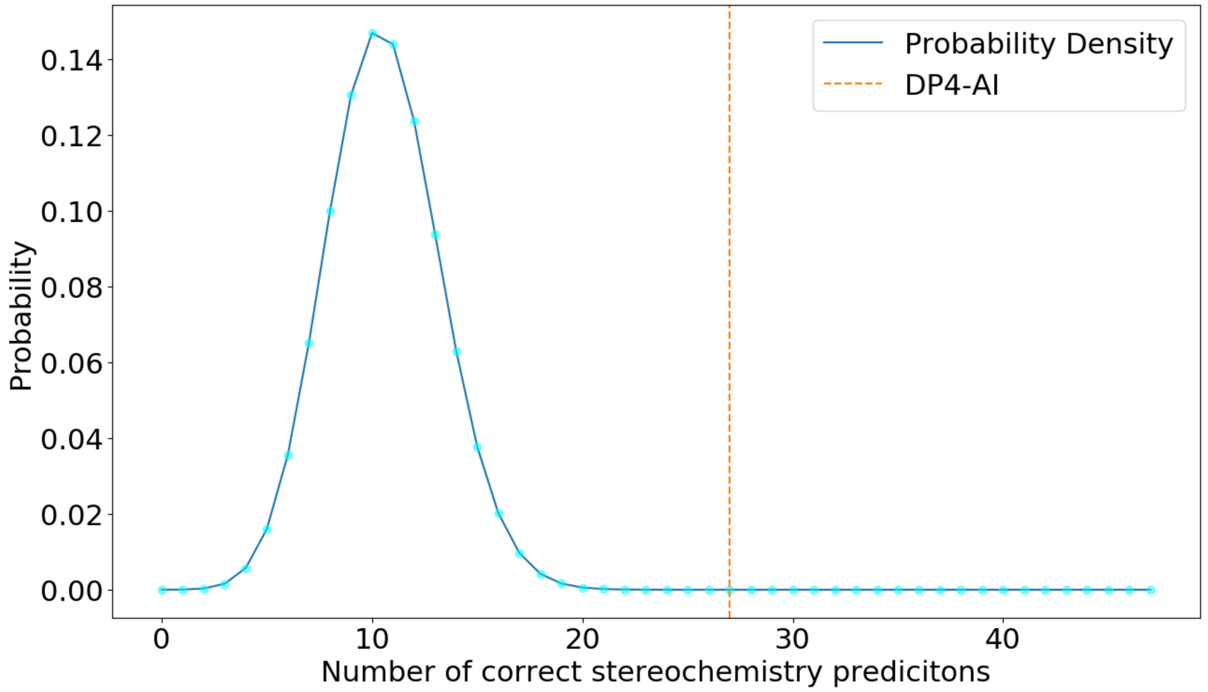

Figure 40: Figure illustrating the probability of correctly assigning the stereochemistry of different numbers molecules in this dataset by random guessing. The expected number of molecules correctly assigned by chance is 10, and the probability of assigning as many molecules correctly as DP4-AI being $2.1 * 10^{-8} \pm 1 * 10^{-9}$. These values are based on $1 * 10^{9}$ randomly simulated results.

3.3 Full Results

Below is a table summarising the DP4 probabilities (expressed as percentages) assigned to the correct diastereomers of the molecules in the dataset by DP4-AI and the pairwise AA (respectively). This table contains the results at the three levels of theory tested with their corresponding most relevant statistical model.

Molecule	Number of Diastereomers	MM 3g	Opt cross validation	Opt E crossvalidation
AT1	2	98.9	79.6	73.7
AT2	2	100.0	100.0	100.0
AT3	2	96.1	98.6	99.9
BYH1	4	15.1	0.0	0.0
BYH2	4	99.5	70.4	0.0
IP1	4	5.3	13.6	48.4
IP2	2	0.0	2.6	0.0
IP3	2	99.2	33.3	54.2
IP4	2	99.3	19.9	99.4
IP5	4	45.7	94.0	74.6
JB10	4	42.7	85.8	97.7
JB11	8	0.3	0.0	0.0
JB12	8	0.9	99.8	0.3
JB13A	4	99.8	100.0	100.0
JB13B	4	0.1	49.6	22.8
JB1	4	0.0	0.0	3.6
JB2	4	0.1	0.0	0.0
JB3	4	0.4	96.4	91.5
JB4	2	100.0	100.0	100.0
JB5	4	100.0	97.7	97.9
JB6	16	99.9	83.3	97.5
JB7	16	46.9	0.0	1.6
JB8	8	5.5	64.8	95.8
JB9	4	63.2	92.8	87.9
KE1	4	93.7	100.0	100.0
KE2	16	20.7	20.7	42.6
KE3	16	15.9	99.7	99.5
NL1A	4	54.7	54.0	97.3
NL1B	4	42.8	98.3	93.6
NL2A	16	0.0	0.0	0.0
NL2B	16	0.0	0.0	0.0
NP1	32	57.1	88.6	87.7
NP2	32	99.9	99.9	99.8
NP3A	8	0.9	1.3	0.8
NP3B	8	0.6	0.4	0.9
NP4	8	0.0	16.8	53.5
NP5	2	100.0	100.0	100.0
OD1	4	5.0	0.0	17.9
TP1	8	2.6	7.3	1.2
TP2	8	19.2	0.9	34.3
TP3	8	0.9	43.2	52.8
TP4	8	0.2	0.3	1.3
TS1	4	0.1	59.5	0.0
TS2	4	0.0	0.0	0.0
TS3A	8	7.9	0.0	0.0
TS3B	8	0.0	30.7	99.6
TS4	8	0.0	0.0	0.0

Figure 41: Table displaying the DP4 probabilities (as percentages) assigned to the correct diastereomer of each molecule by DP4-AI at the three different levels of theory tested

Molecule	Number of Diastereomers	MM 3g	Opt cross validation	Opt E crossvalidation
AT1	2	99.1	83.2	82.9
AT2	2	100.0	100.0	100.0
AT3	2	96.7	98.1	99.7
BYH1	4	81.6	54.3	8.6
BYH2	4	99.2	35.6	0.3
IP1	4	6.2	14.9	49.5
IP2	2	36.2	99.7	99.6
IP3	2	63.6	30.4	51.9
IP4	2	99.9	19.8	99.9
IP5	4	19.5	88.5	74.5
JB10	4	79.1	2.3	5.8
JB11	8	99.7	80.7	0.0
JB12	8	0.2	8.0	91.7
JB13A	4	99.6	100.0	100.0
JB13B	4	91.9	99.9	0.1
JB1	4	3.4	45.4	100.0
JB2	4	5.0	0.0	0.0
JB3	4	100.0	100.0	100.0
JB4	2	100.0	100.0	100.0
JB5	4	100.0	100.0	100.0
JB6	16	97.7	99.8	100.0
JB7	16	0.0	0.0	0.0
JB8	8	4.2	35.9	26.3
JB9	4	36.8	69.6	72.4
KE1	4	66.6	100.0	100.0
KE2	16	2.0	32.5	27.0
KE3	16	22.0	99.6	99.3
NL1A	4	94.7	85.9	42.1
NL1B	4	92.1	98.7	99.9
NL2A	16	0.1	35.9	0.0
NL2B	16	67.6	79.5	0.0
NP1	32	95.0	97.1	94.9
NP2	32	99.5	99.3	98.9
NP3A	8	1.3	0.3	0.2
NP3B	8	6.8	2.4	5.4
NP4	8		99.3	99.2
NP5	2	100.0	100.0	100.0
OD1	4	4.2	1.0	96.3
TP1	8	2.8	8.5	1.5
TP2	8	1.4	0.1	48.6
TP3	8	0.6	49.1	56.6
TP4	8	2.6	0.4	1.0
TS1	4	0.0	0.0	33.5
TS2	4	0.0	0.0	0.0
TS3A	8	1.2	0.0	21.5
TS3B	8	24.0	92.5	100.0
TS4	8	25.1	2.3	100.0

Figure 42: Table displaying the DP4 probabilities (as percentages) assigned to the correct diastereomer of each molecule by the pairwise AA at the three different levels of theory tested

Presented below are the sets of parameters used for the statistical models tested in this study for each set of computational conditions.

Carbon						
	Mean (PPM)			Standard Deviation (PPM)		
Conditions	1g	2g	3g	1g	2g	3g
MM 1g	-0.063372			2.328593		
MM 2g	0.23908	-0.183497		3.215941	1.748467	
MM 3g	0.00281	-0.009213	-0.01008	1.679699	3.227146	2.39291
Opt 1g	-0.194465			1.960887		
Opt 2g	-0.245383	0.2279		1.500306	2.530502	
Opt 3g	-0.006017	-0.002113	-0.008755	2.708701	1.396216	1.909214
Opt E 1g	-0.15355			1.986855		
Opt E 2g	0.197469	-0.150954		2.652192	1.561153	
Opt E 3g	0.001598	-0.009914	-0.007496	1.766254	3.000603	1.559535

Proton						
	Mean (PPM)			Standard Deviation (PPM)		
Conditions	1g	2g	3g	1g	2g	3g
MM 1g	0.000847			0.176454		
MM 2g	0.001898	0.003405		0.255891	0.129655	
MM 3g	0.007965	-0.00702	-0.002382	0.257145	0.109309	0.217931
Opt 1g	-0.016458			0.169782		
Opt 2g	0.034137	-0.016918		0.259518	0.121696	
Opt 3g	-0.011162	-0.004944	0.004681	0.304799	0.119454	0.160343
Opt E 1g	-0.008667			0.165246		
Opt E 2g	0.005395	-0.005487		0.264481	0.116806	
Opt E 3g	0.004621	0.008918	-0.003164	0.160656	0.313006	0.110515

Figure 43

Presented below are the full DP4 results for each combination of, computational conditions, AA and statistical model. All the results are presented in the same manner, the blue bar always represents the DP4 probability assigned to the correct diastereomer. The orange bar is always the probability of the incorrect diasteroemer predicted with the highest probability, followed the a green bar for the second most probable incorrect diastereomer, then red, purple, brown etc. The black bars represent the probability of choosing the correct diastereomer by chance.

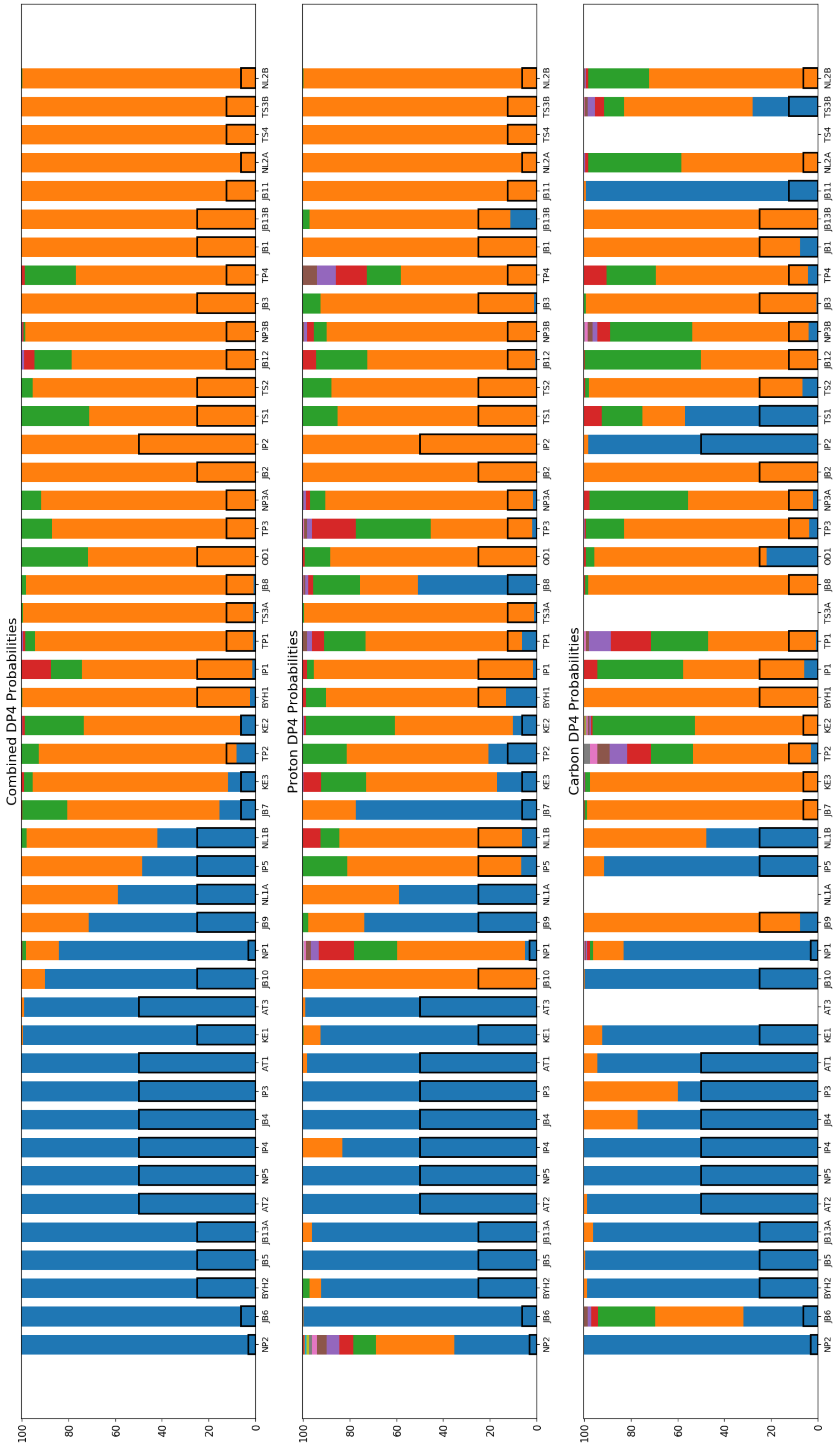

Figure 44: Assignment: DP4-AI, Conditions: Geometries = MM, NMR = mPW1PW91/6-311g(d), Single Point energy = mPW1PW91/6-311g(d), Statistical Model = External

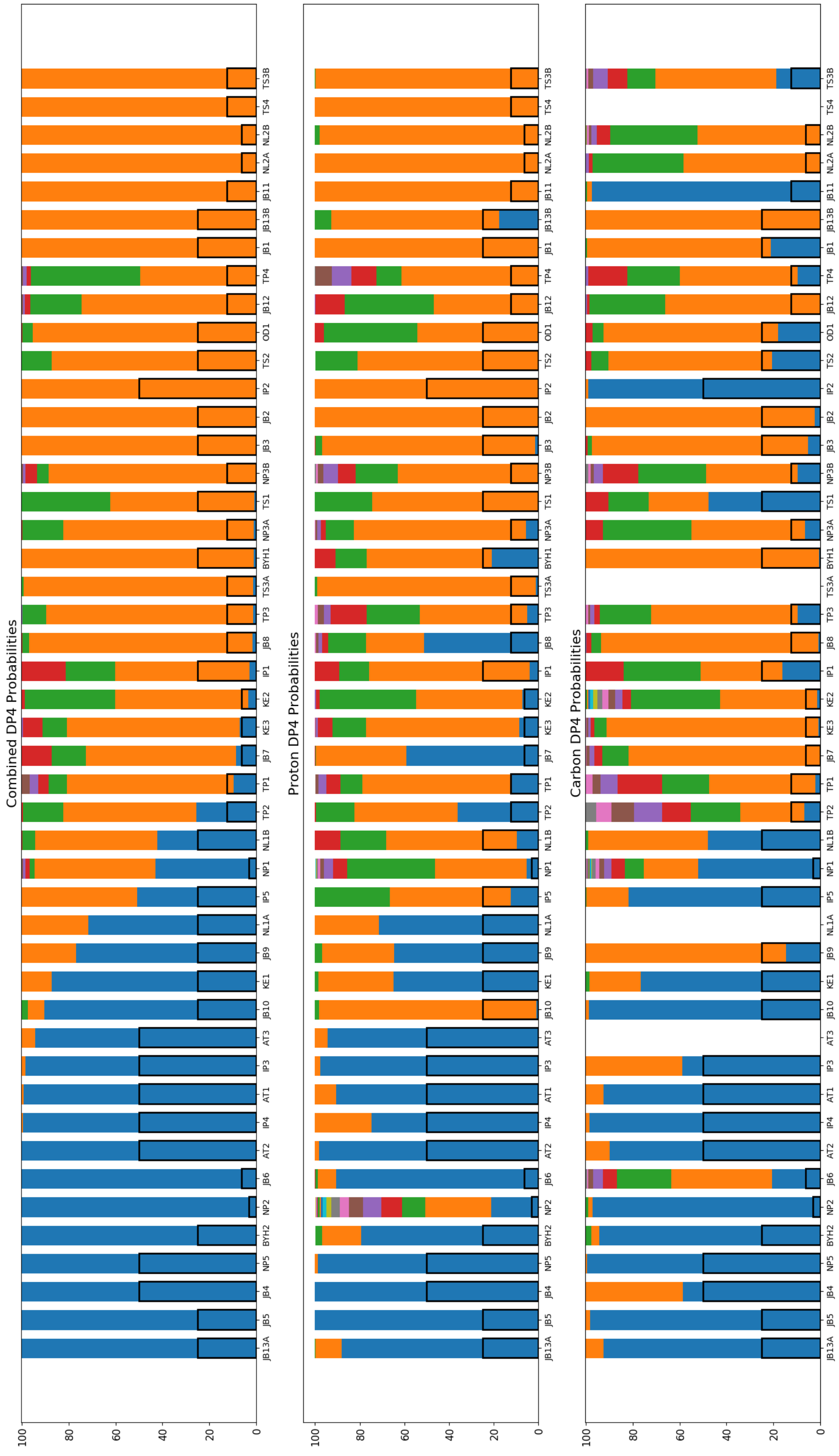

Figure 45: Assignment: DP4-AI, Conditions: Geometries = MM, NMR = mPW1PW91/6-311g(d), Single Point energy = mPW1PW91/6-311g(d), Statistical Model = 1 Gaussian

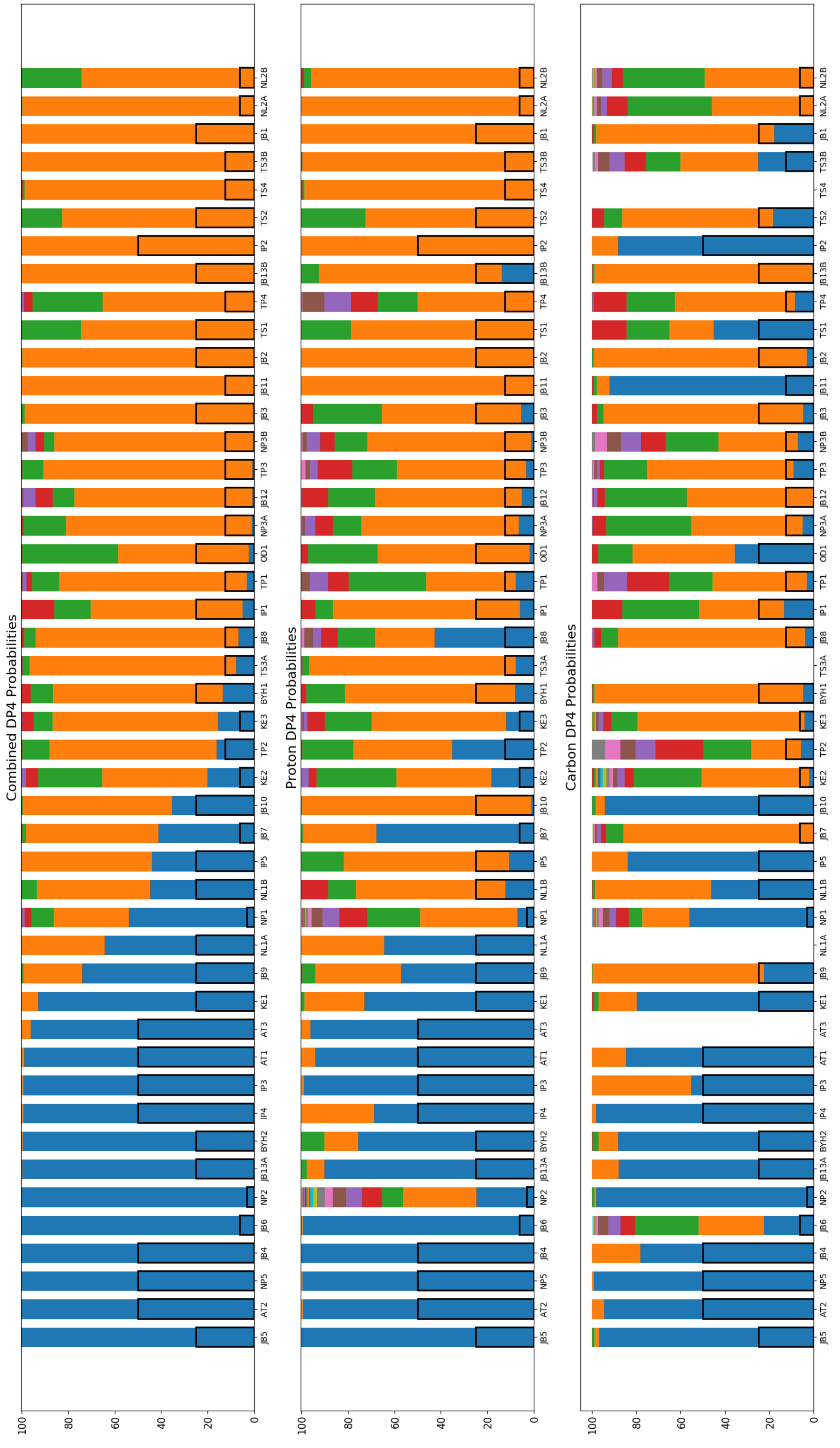

Figure 46: Assignment: DP4-AI, Conditions: Geometries = MM, NMR = mPW1PW91/6-311g(d), Single Point energy = mPW1PW91/6-311g(d), Statistical Model = 2 Gaussian

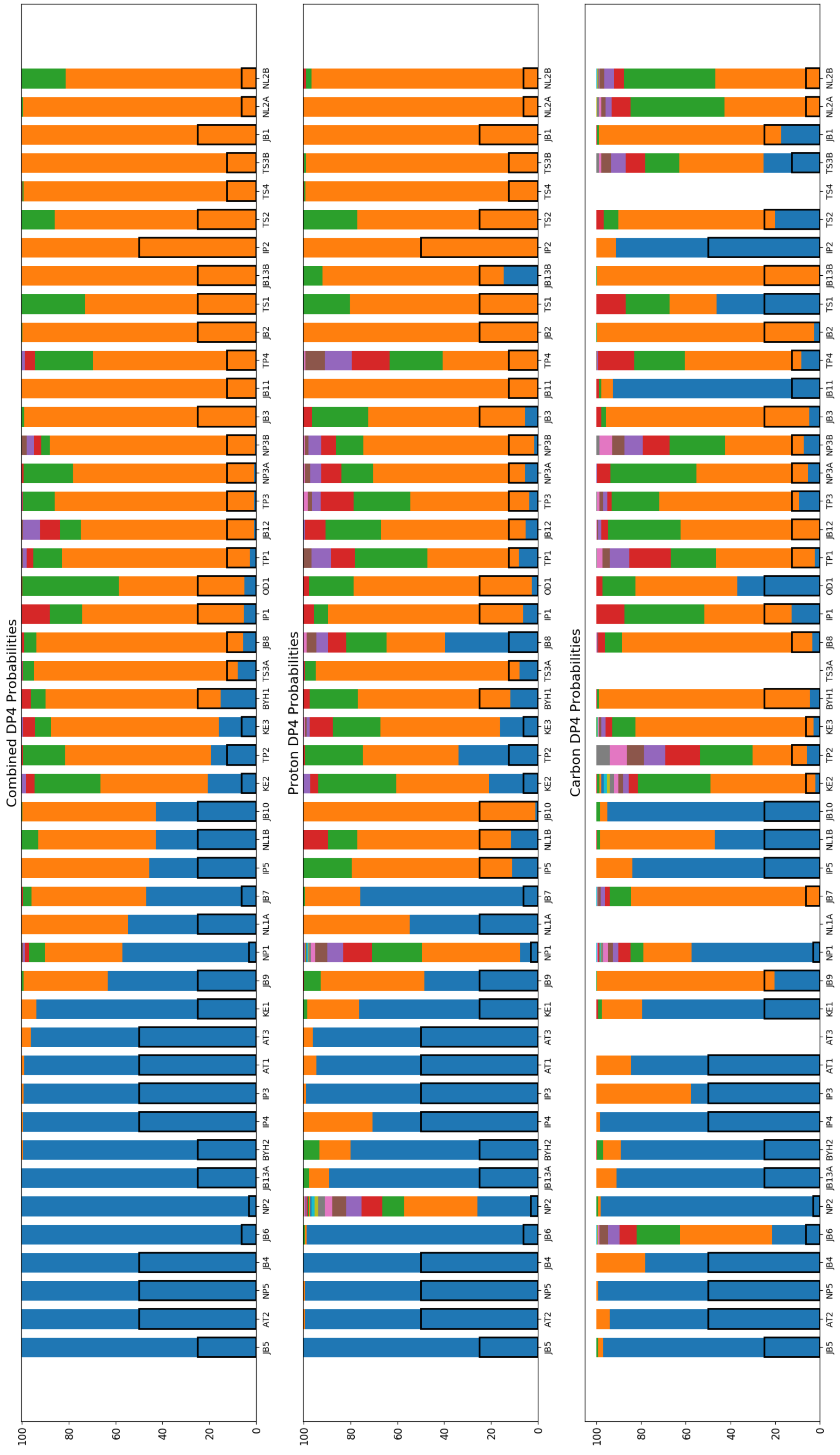

Figure 47: Assignment: DP4-AI, Conditions: Geometries = MM, NMR = mPW1PW91/6-311g(d), Single Point energy = mPW1PW91/6-311g(d), Statistical Model = 2 Gaussian

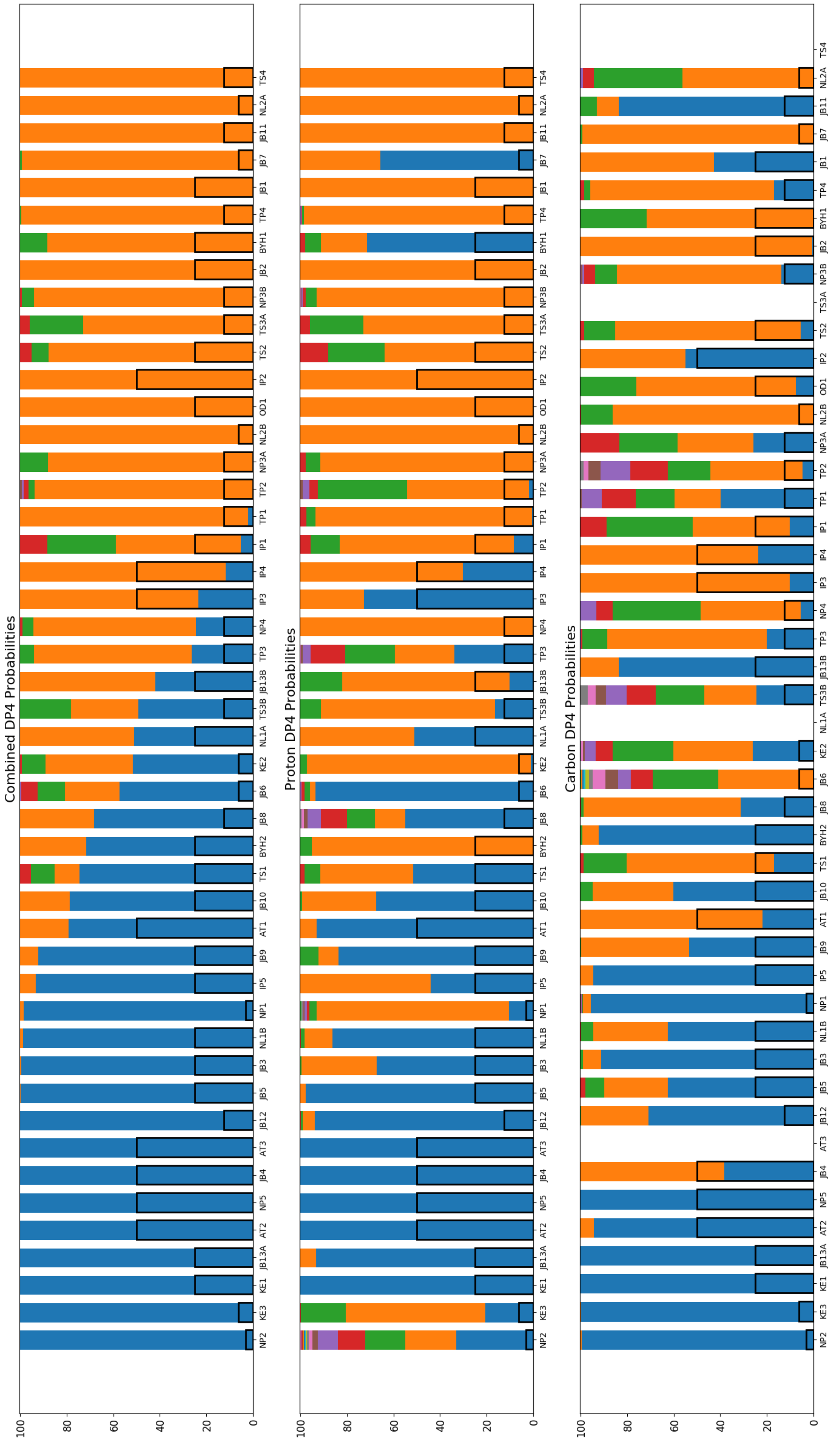

Figure 48: Assignment: DP4-AI, Conditions: Geometries = DFT Optimised B3LYP/6-31g(d), NMR = mPW1PW91/6-311g(d), Single Point energy = mPW1PW91/6-311g(d), Statistical Model = External

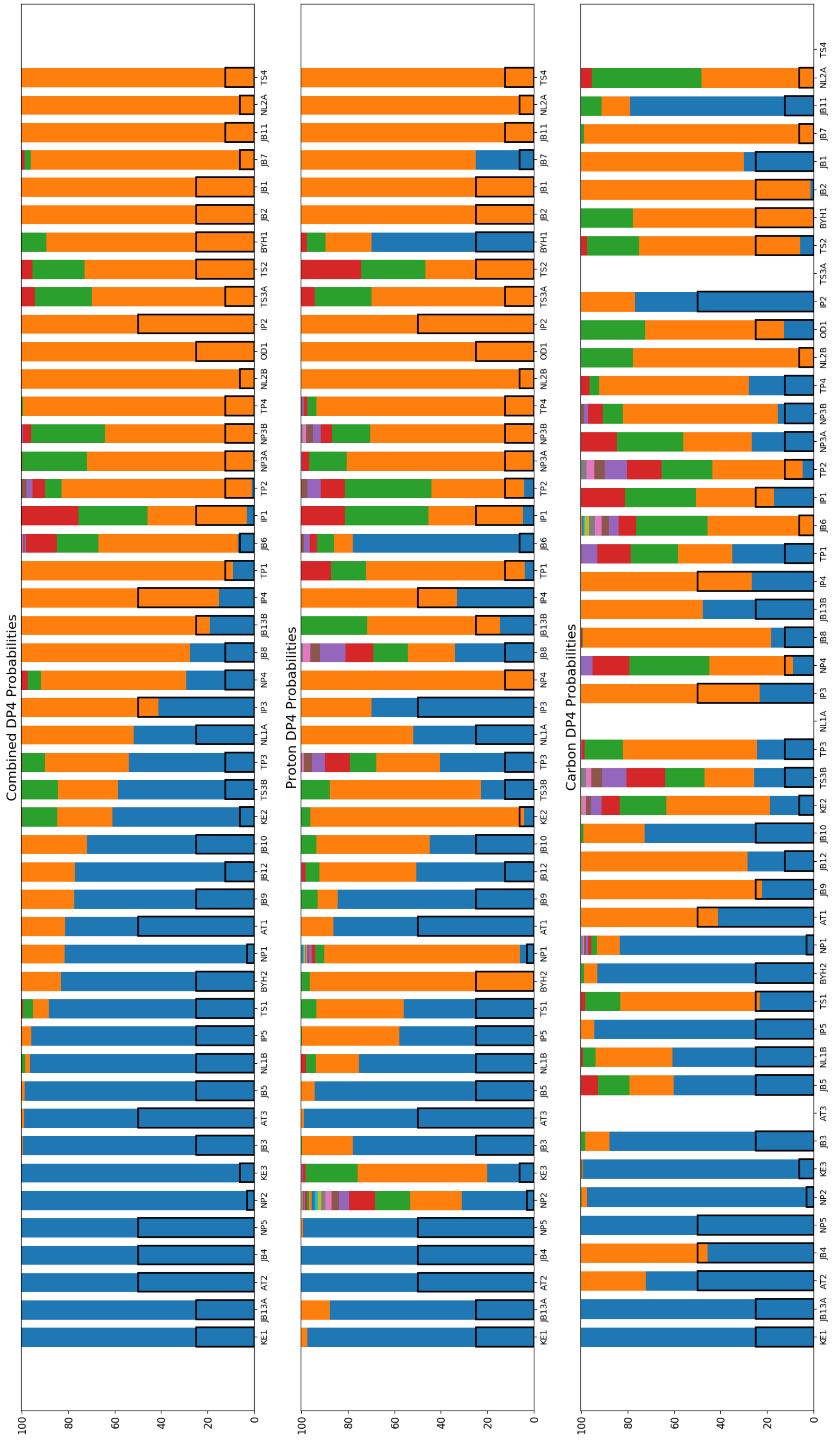

Figure 49: Assignment: DP4-AI, Conditions: Geometries = DFT Optimised B3LYP/6-31g(d), NMR = mPW1PW91/6-311g(d), Single Point energy = mPW1PW91/6-311g(d), Statistical Model = 1 Gaussian

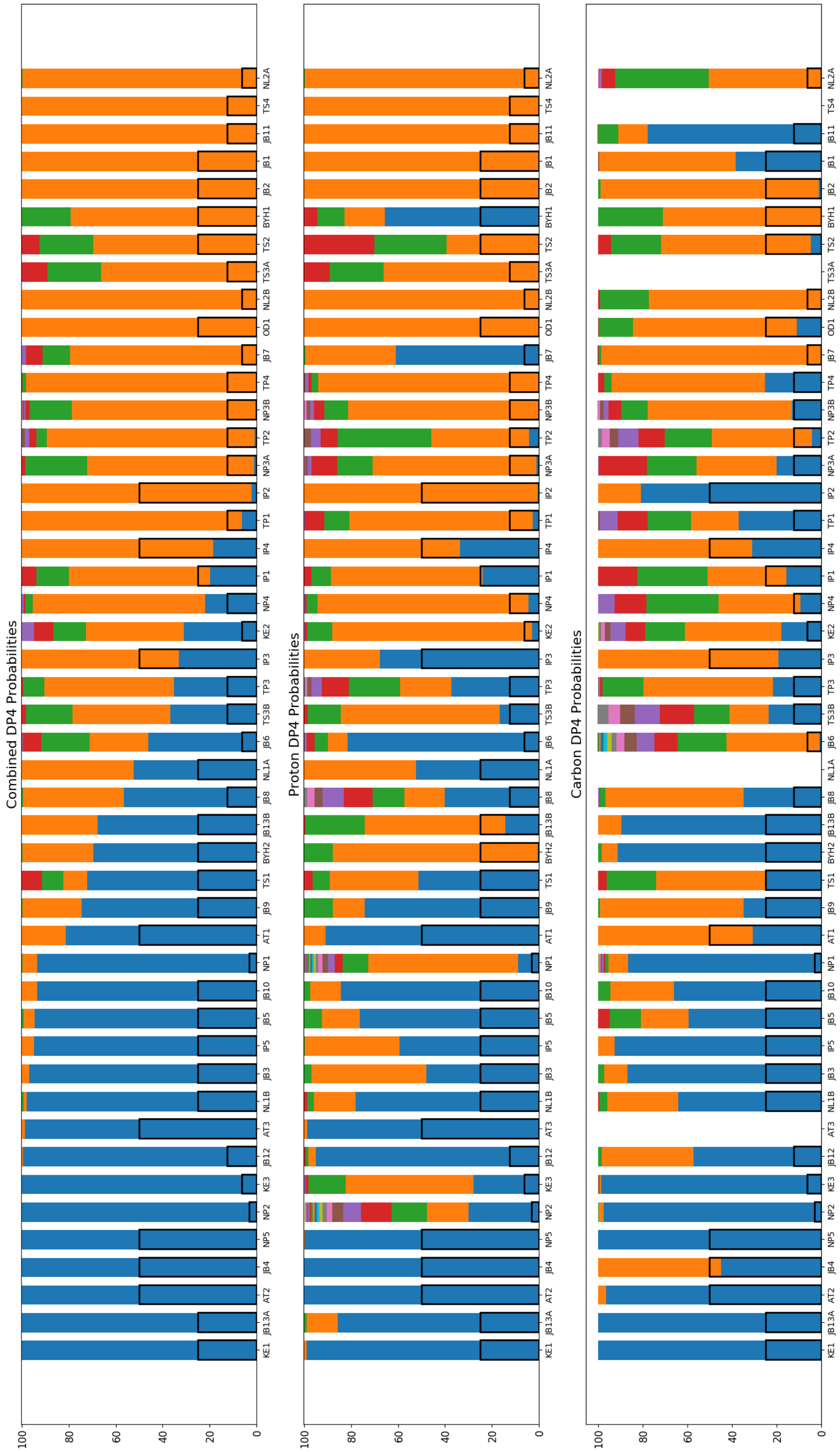

Figure 50: Assignment: DP4-AI, Conditions: Geometries = DFT Optimised B3LYP/6-31g(d), NMR = mPW1PW91/6-311g(d), Single Point energy = mPW1PW91/6-311g(d), Statistical Model = 2 Gaussian

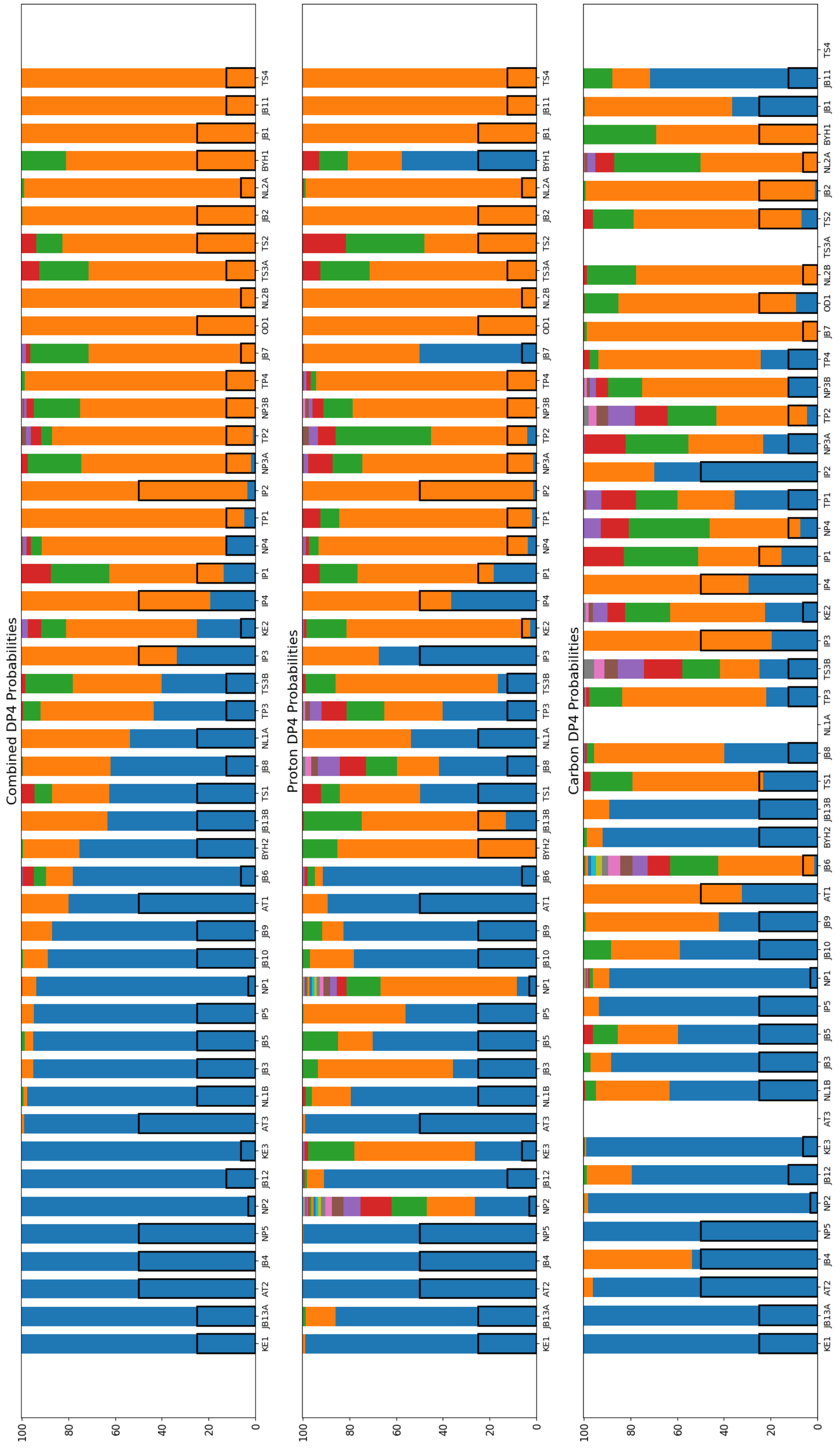

Figure 51: Assignment: DP4-AI, Conditions: Geometries = DFT Optimised B3LYP/6-31g(d), NMR = mPW1PW91/6-311g(d), Single Point energy = mPW1PW91/6-311g(d), Statistical Model = 3 Gaussian

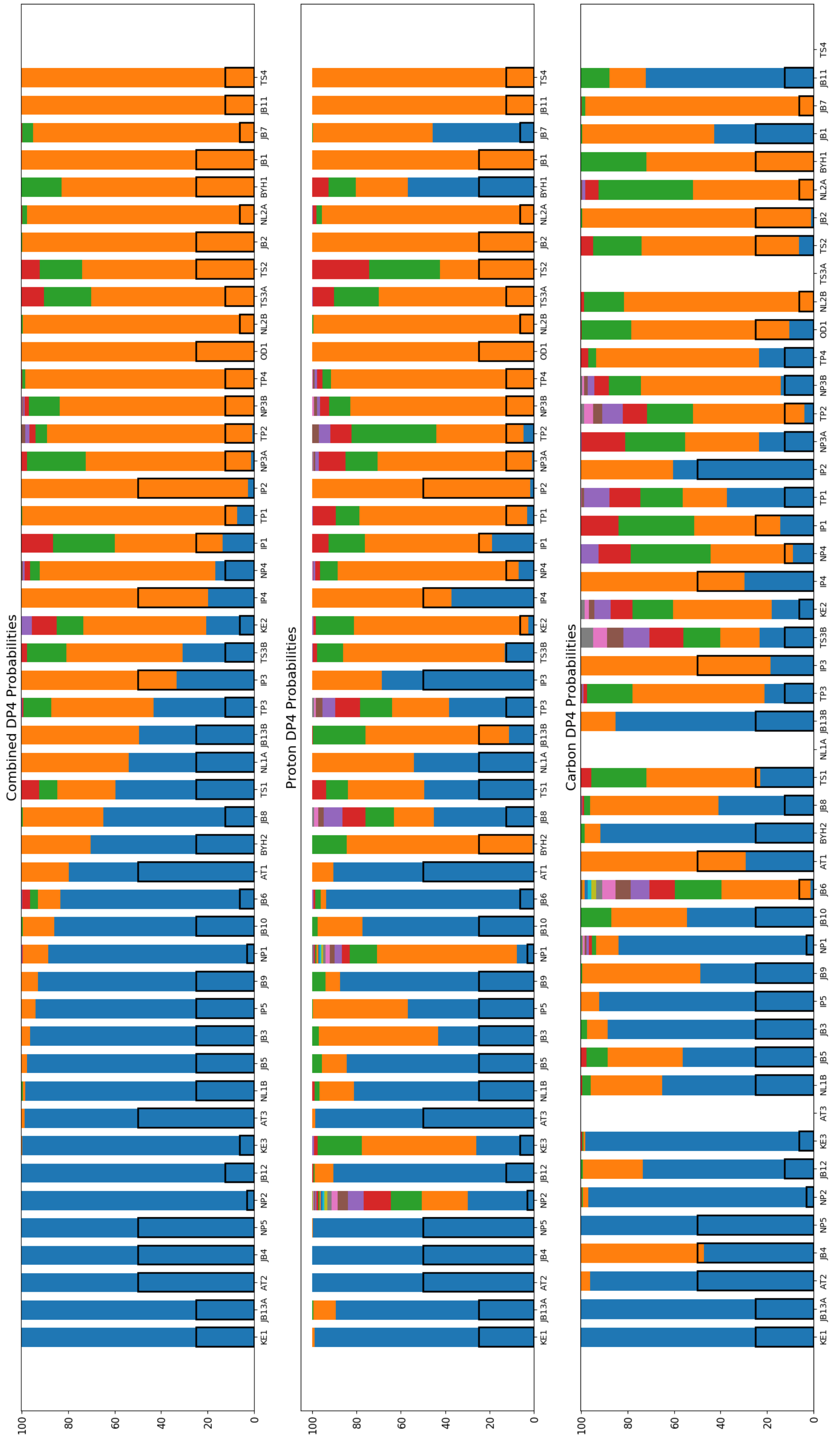

Figure 52: Assignment: DP4-AI, Conditions: Geometries = DFT Optimised B3LYP/6-31g(d), NMR = mPW1PW91/6-311g(d), Single Point energy = mPW1PW91/6-311g(d), Statistical Model = cross validation

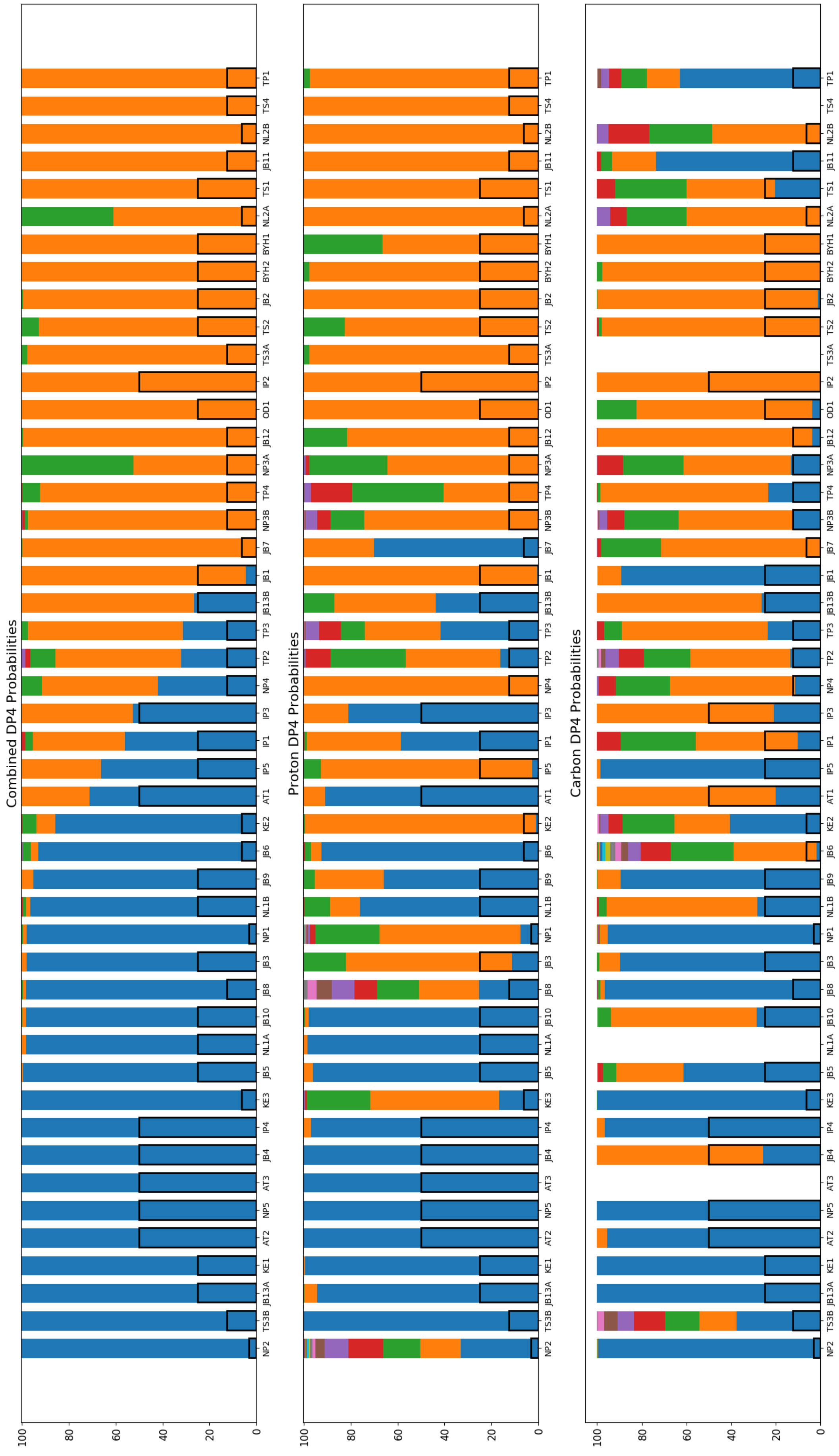

Figure 53: Assignment: DP4-AI, Conditions: Geometries = DFT Optimised B3LYP/6-31g(d), NMR = mPW1PW91/6-311g(d), Single Point energy = M062X/Def2TZVP, Statistical Model = External

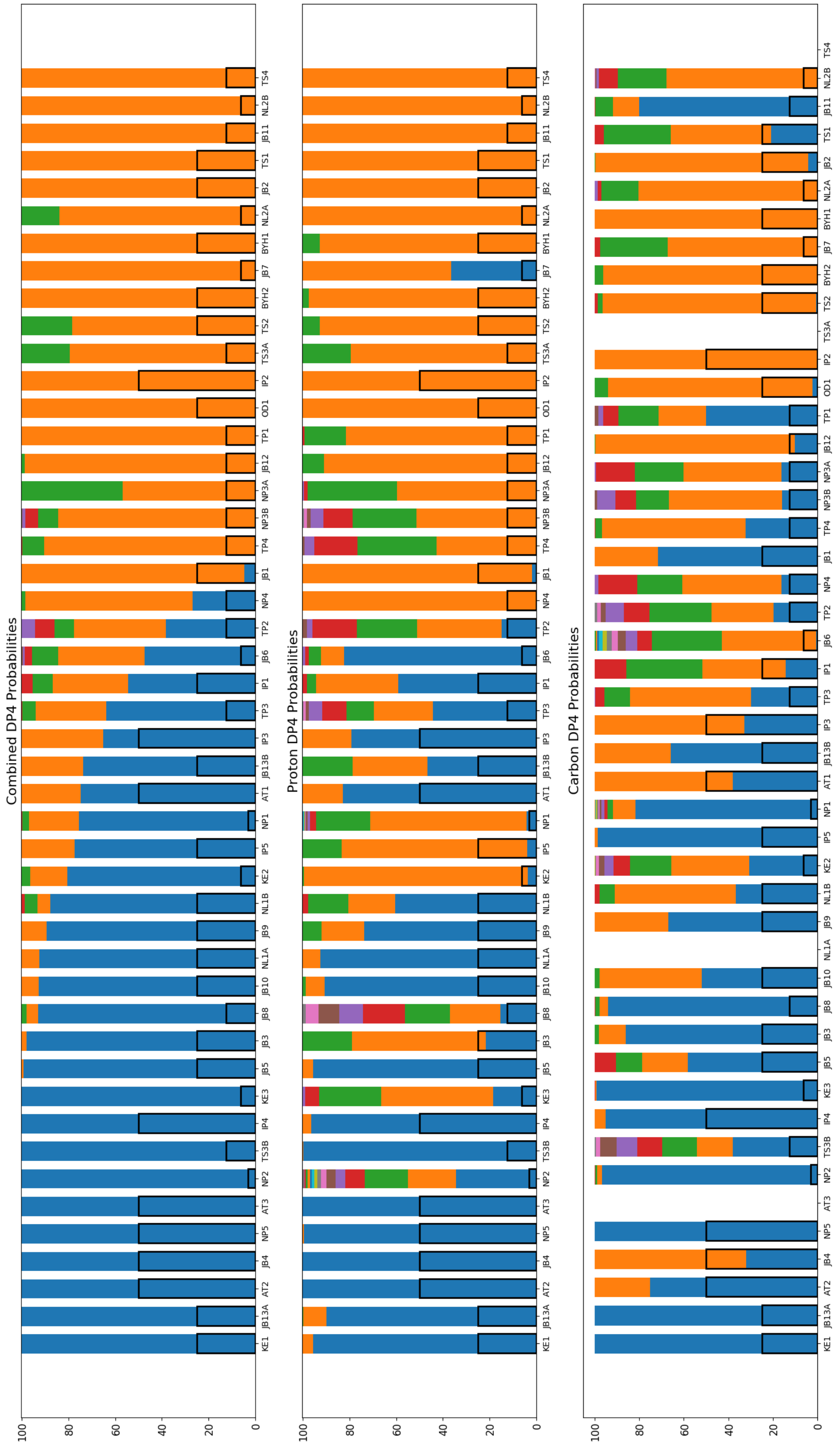

Figure 54: Assignment: DP4-AI, Conditions: Geometries = DFT Optimised B3LYP/6-31g(d), NMR = mPW1PW91/6-311g(d), Single Point energy = M062X/Def2TZVP, Statistical Model = 1 Gaussian

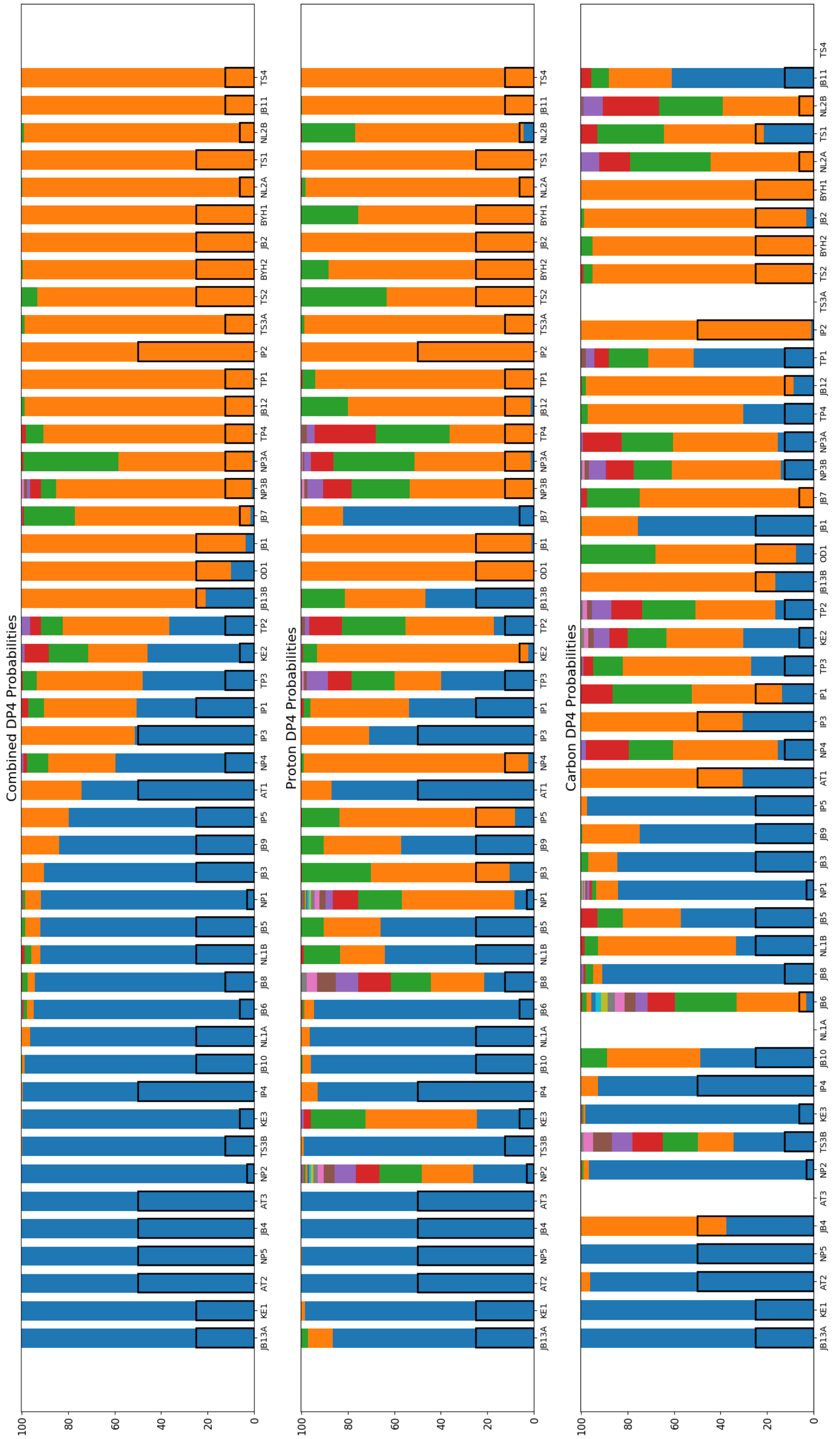

Figure 55: Assignment: DP4-AI, Conditions: Geometries = DFT Optimised B3LYP/6-31g(d), NMR = mPW1PW91/6-311g(d), Single Point energy = M062X/Def2TZVP, Statistical Model = 2 Gaussian

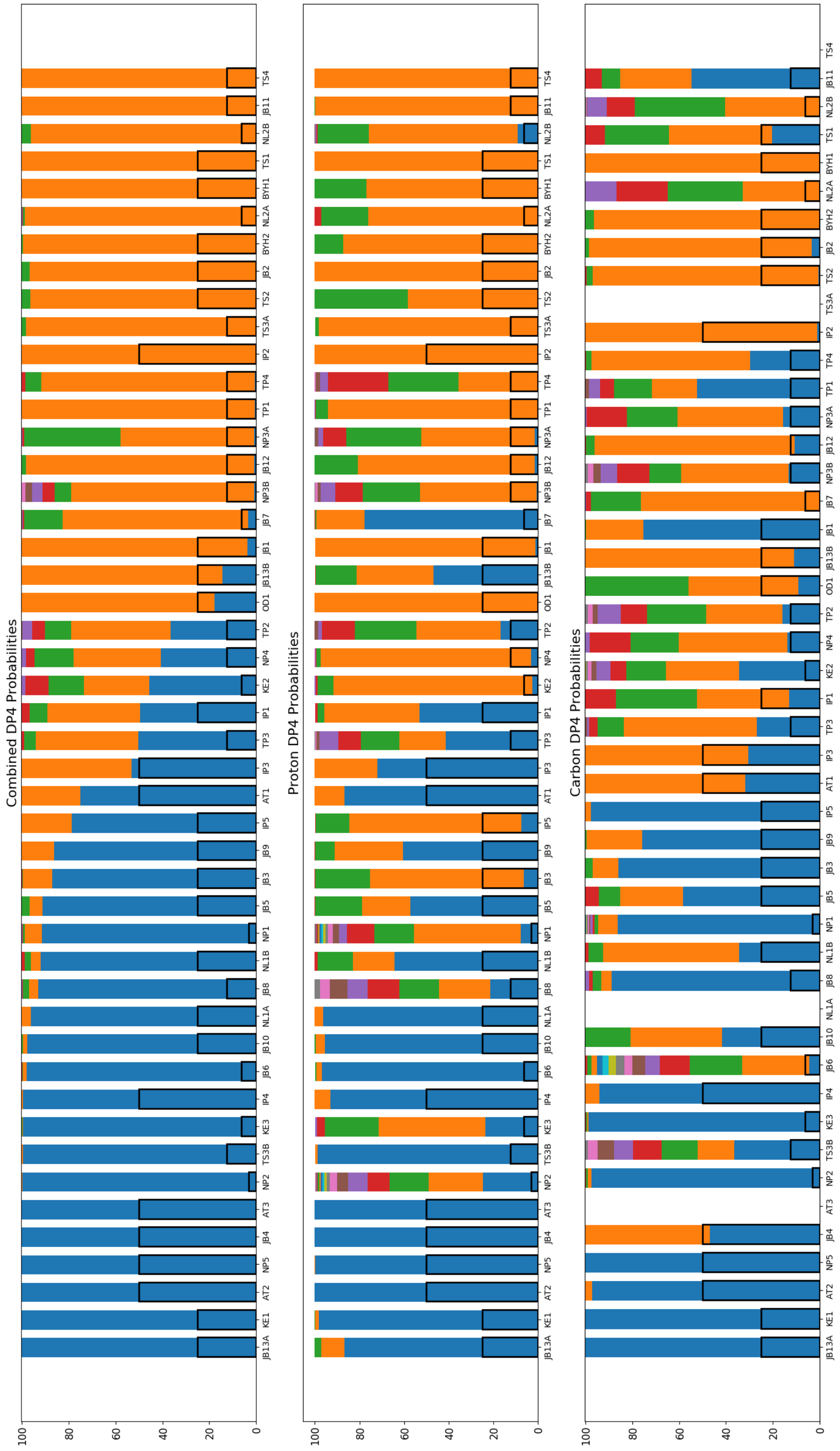

Figure 56: Assignment: DP4-AI, Conditions: Geometries = DFT Optimised B3LYP/6-31g(d), NMR = mPW1PW91/6-311g(d),Single Point energy = M062X/Def2TZVP, Statistical Model = 3 Gaussian

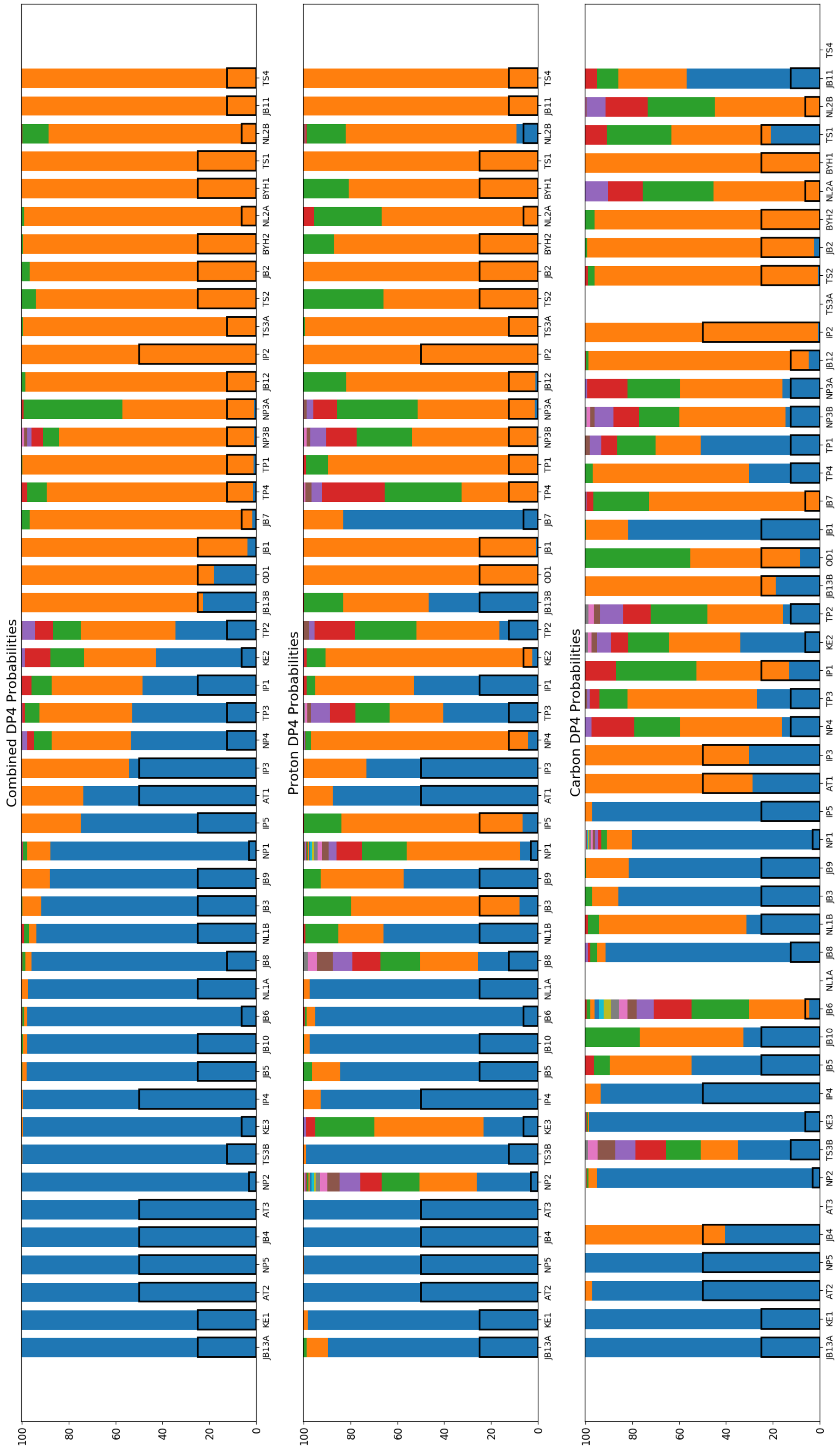

Figure 57: Assignment: DP4-AI, Conditions: Geometries = DFT Optimised B3LYP/6-31g(d), NMR = mPW1PW91/6-311g(d),Single Point energy = M062X/Def2TZVP, Statistical Model = cross validation

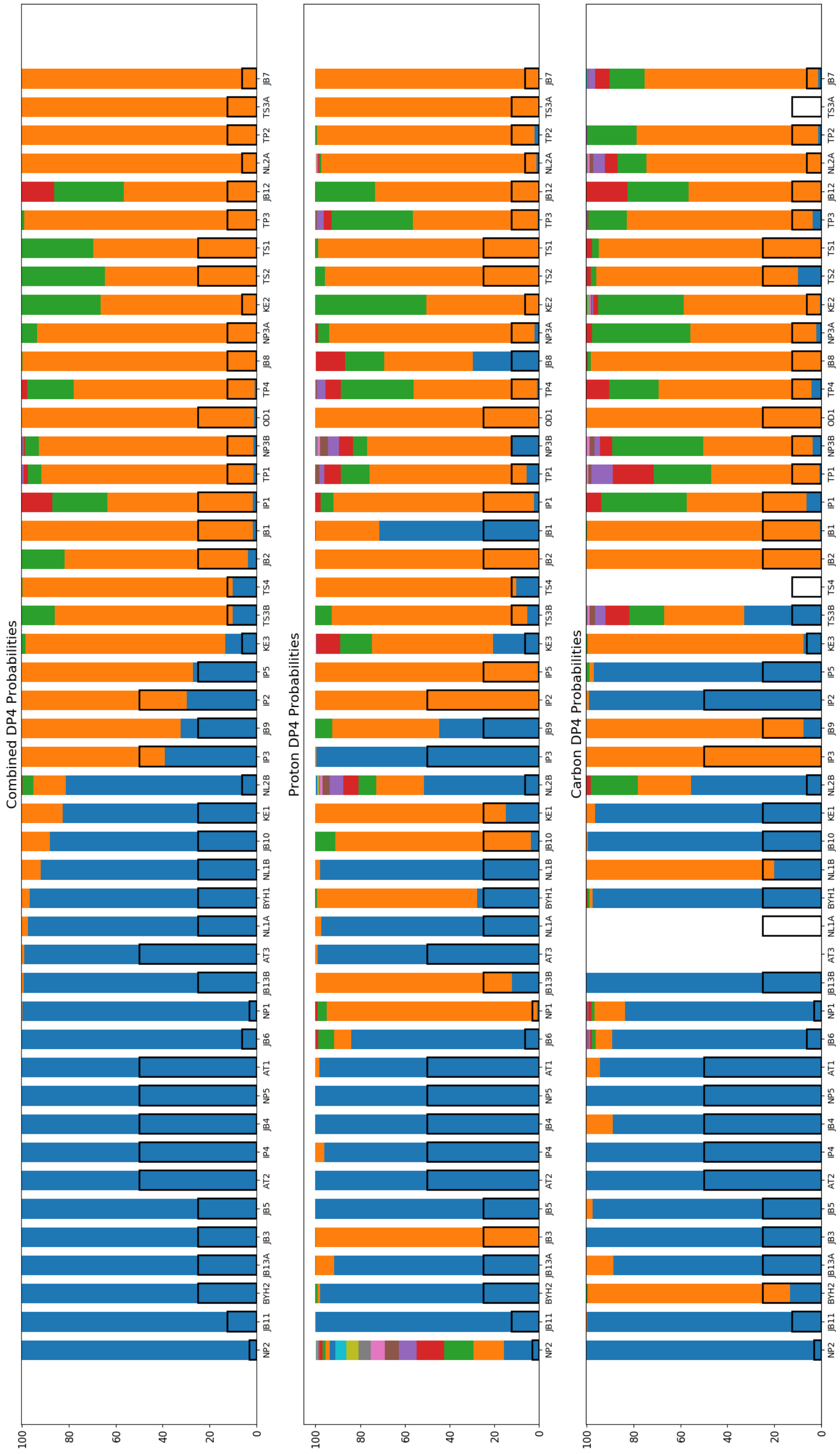

Figure 58: Assignment: DP4-AI, Conditions: Geometries = MM, NMR = mPW1PW91/6-311g(d), Single Point energy = mPW1PW91/6-311g(d), Statistical Model = External

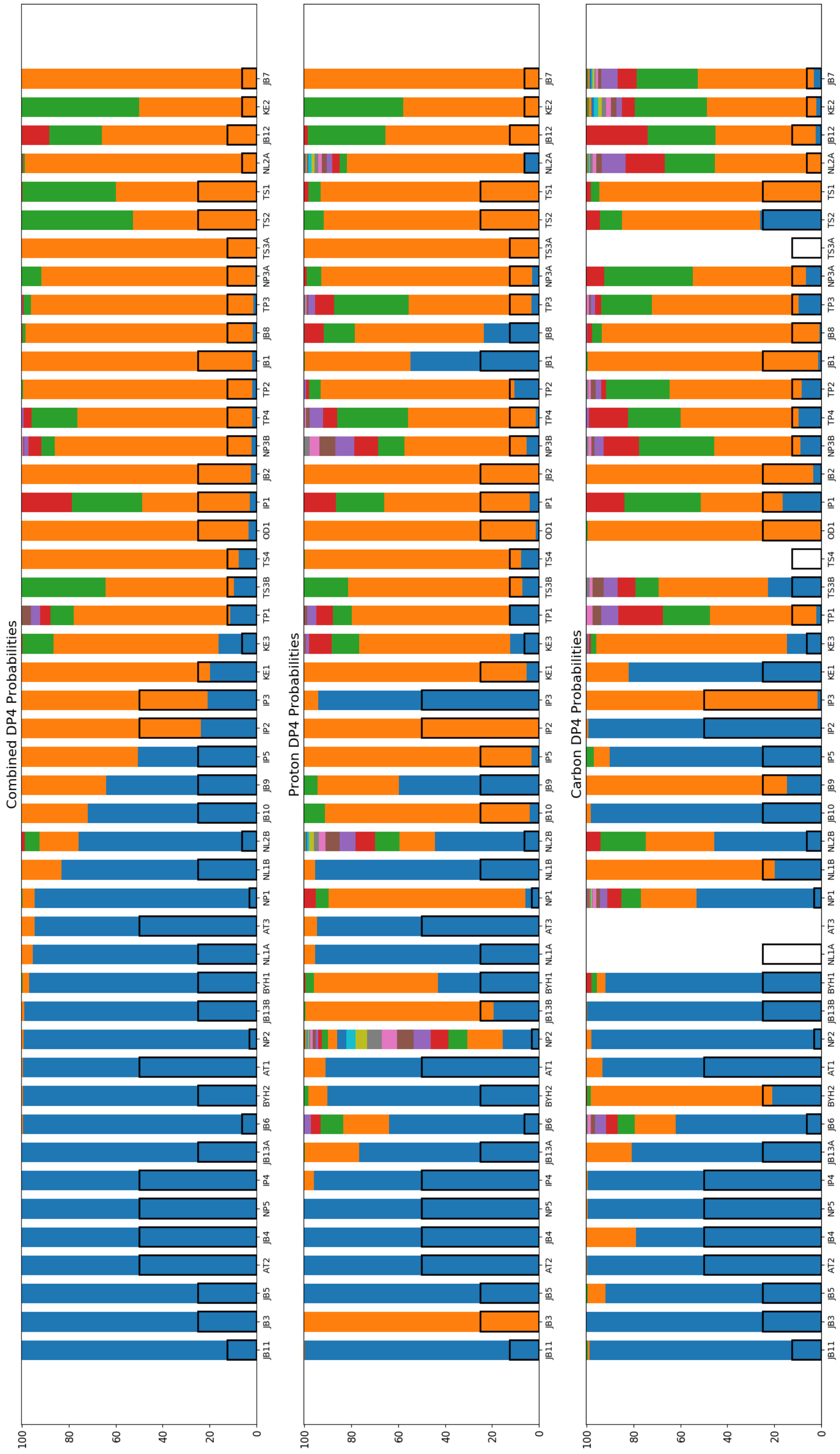

Figure 59: Assignment: DP4-AI, Conditions: Geometries = MM, NMR = mPW1PW91/6-311g(d), Single Point energy = mPW1PW91/6-311g(d), Statistical Model = 1 Gaussian

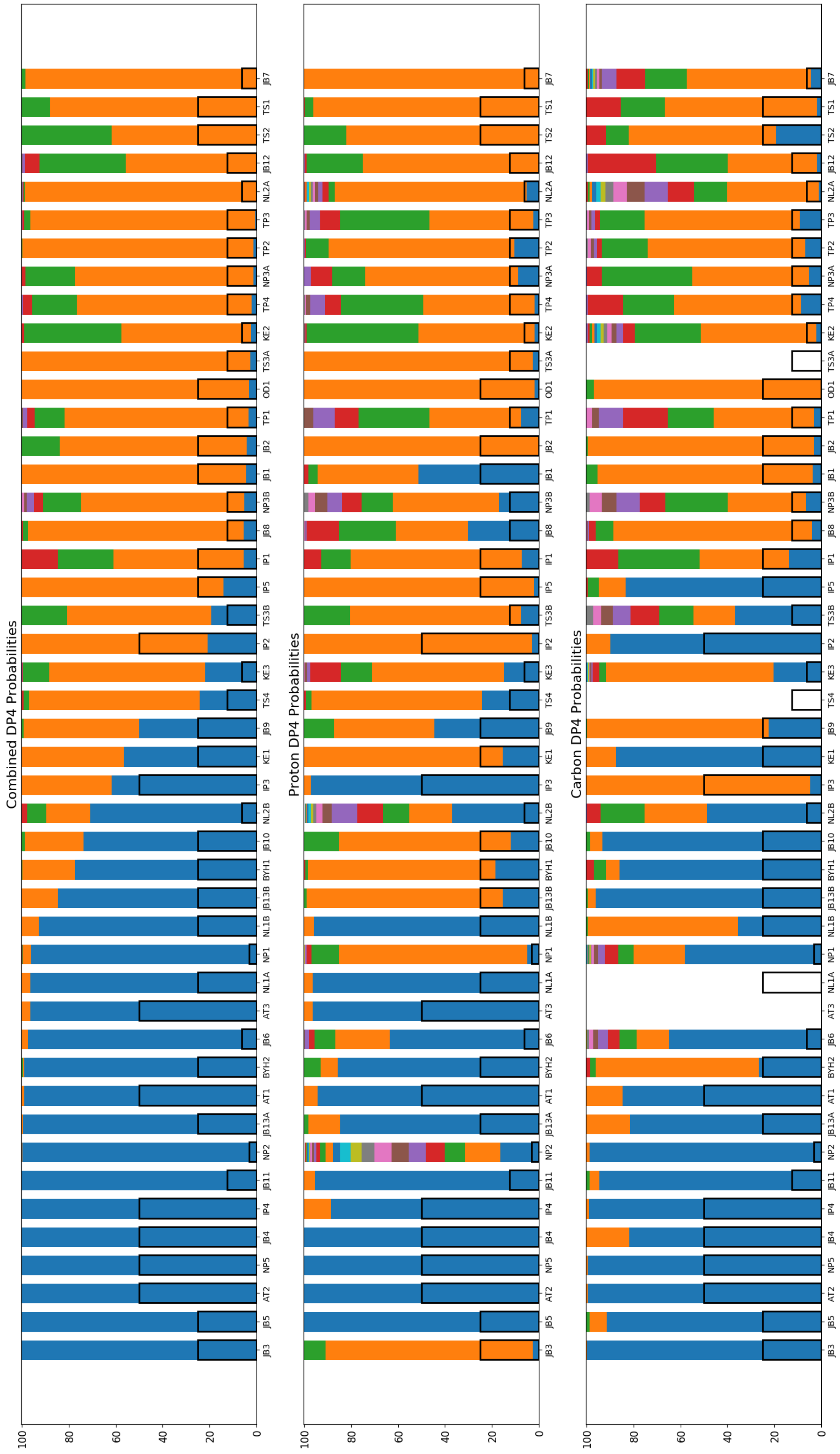

Figure 60: Assignment: DP4-AI, Conditions: Geometries = MM, NMR = mPW1PW91/6-311g(d), Single Point energy = mPW1PW91/6-311g(d), Statistical Model = 2 Gaussian

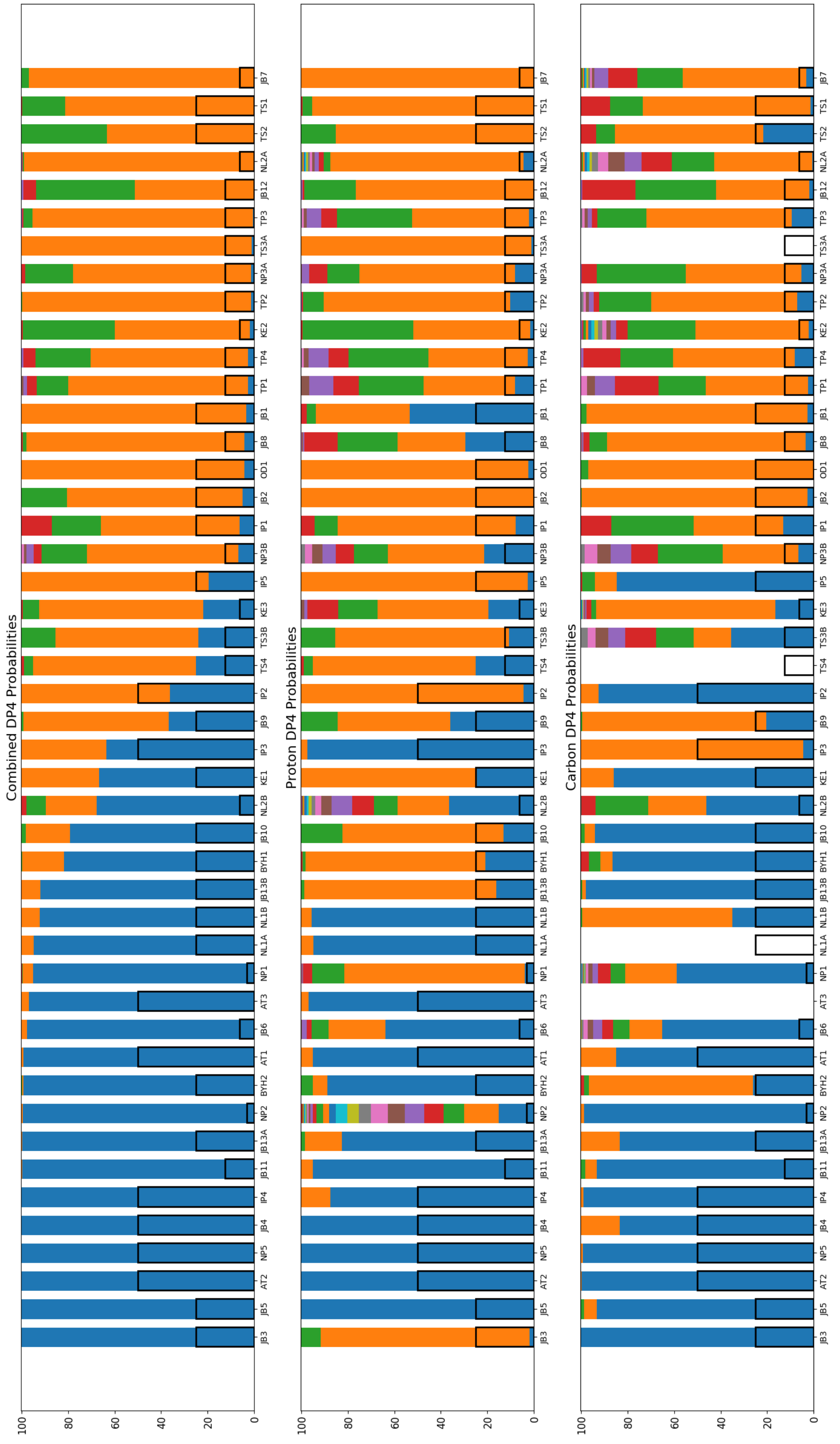

Figure 61: Assignment: DP4-AI, Conditions: Geometries = MM, NMR = mPW1PW91/6-311g(d), Single Point energy = mPW1PW91/6-311g(d), Statistical Model = 2 Gaussian

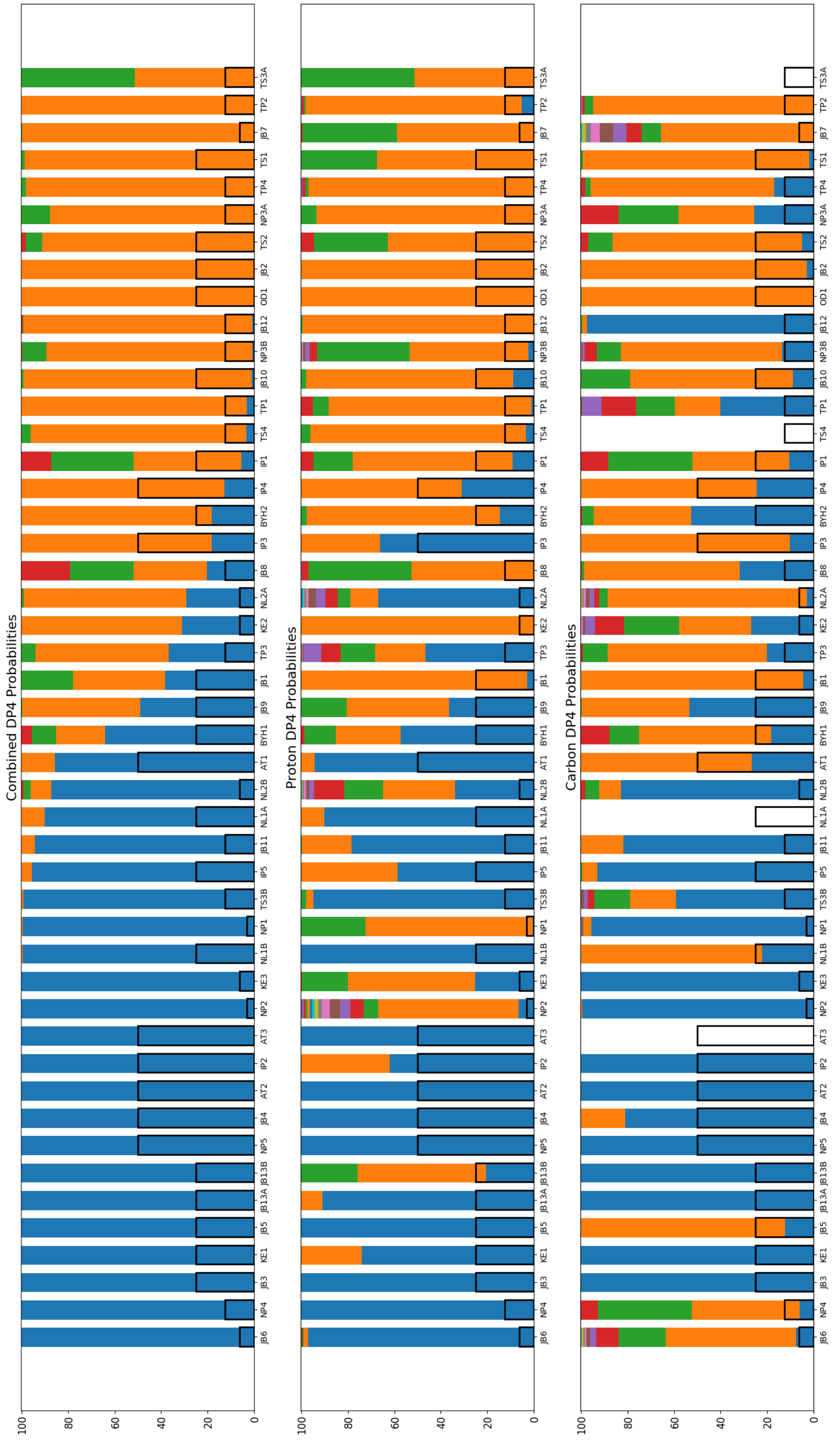

Figure 62: Assignment: DP4-AI, Conditions: Geometries = DFT Optimised B3LYP/6-31g(d), NMR = mPW1PW91/6-311g(d), Single Point energy = mPW1PW91/6-311g(d), Statistical Model = External

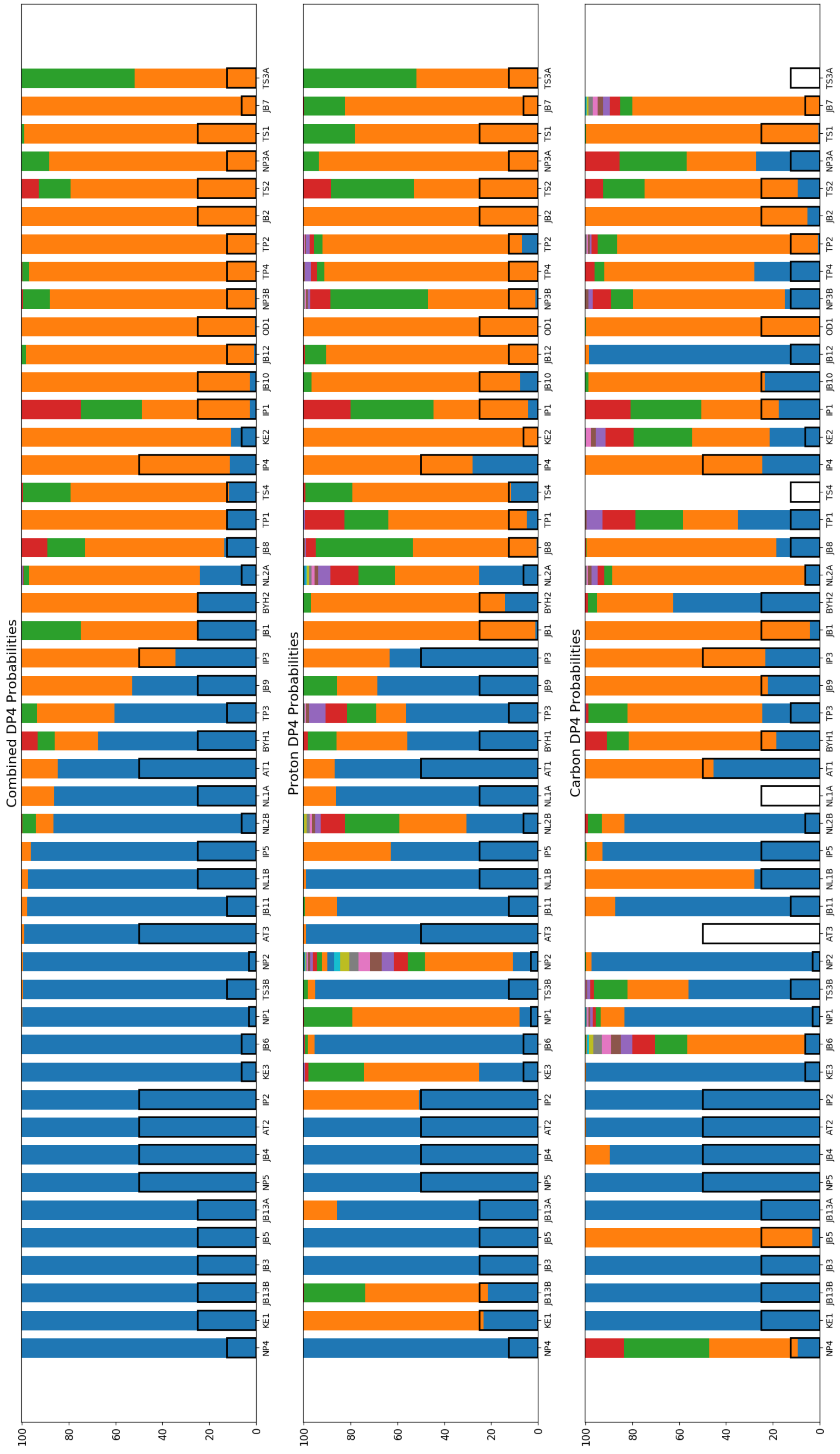

Figure 63: Assignment: DP4-AI, Conditions: Geometries = DFT Optimised B3LYP/6-31g(d), NMR = mPW1PW91/6-311g(d), Single Point energy = mPW1PW91/6-311g(d), Statistical Model = 1 Gaussian

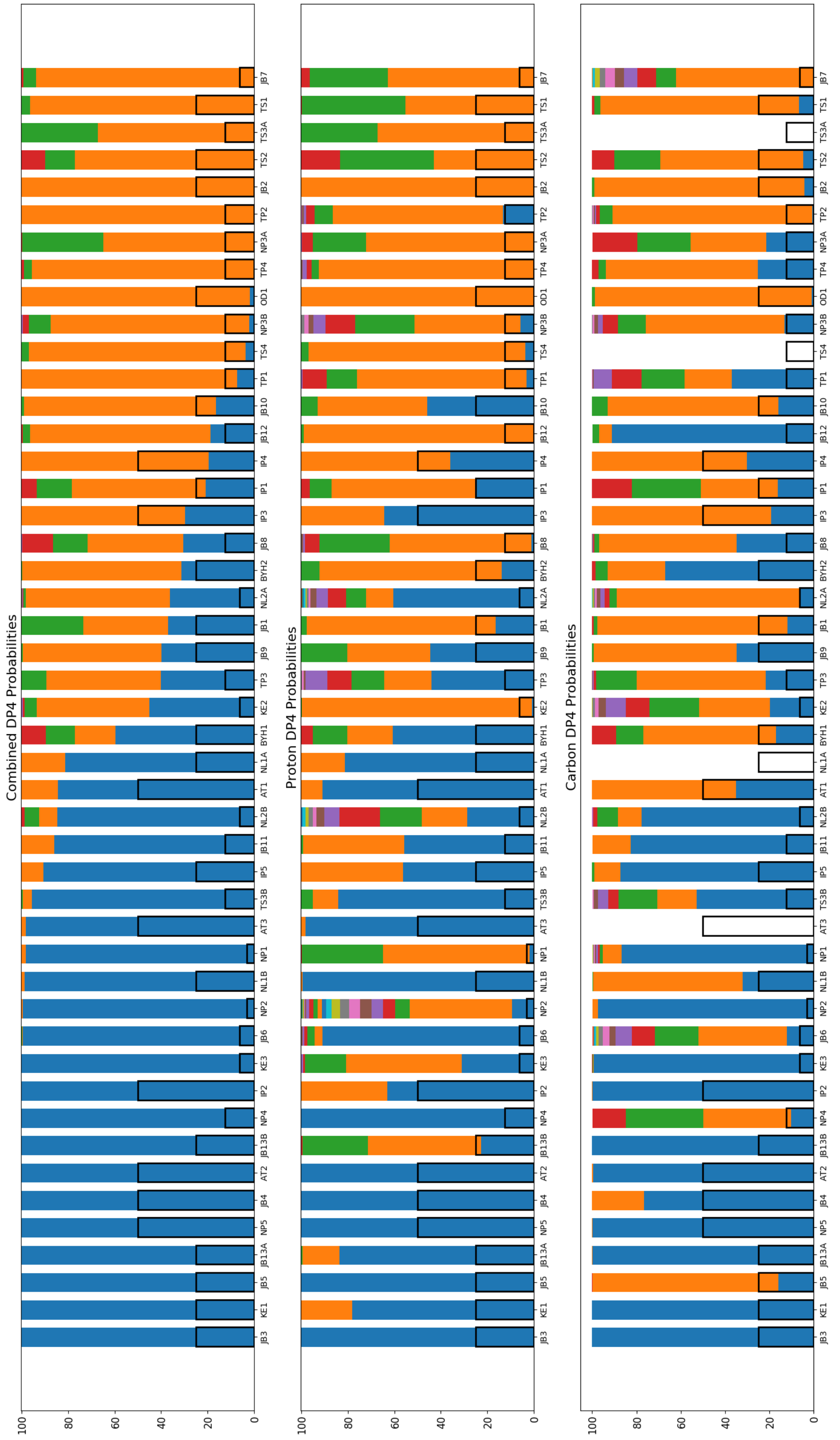

Figure 64: Assignment: DP4-AI, Conditions: Geometries = DFT Optimised B3LYP/6-31g(d), NMR = mPW1PW91/6-311g(d), Single Point energy = mPW1PW91/6-311g(d), Statistical Model = 2 Gaussian

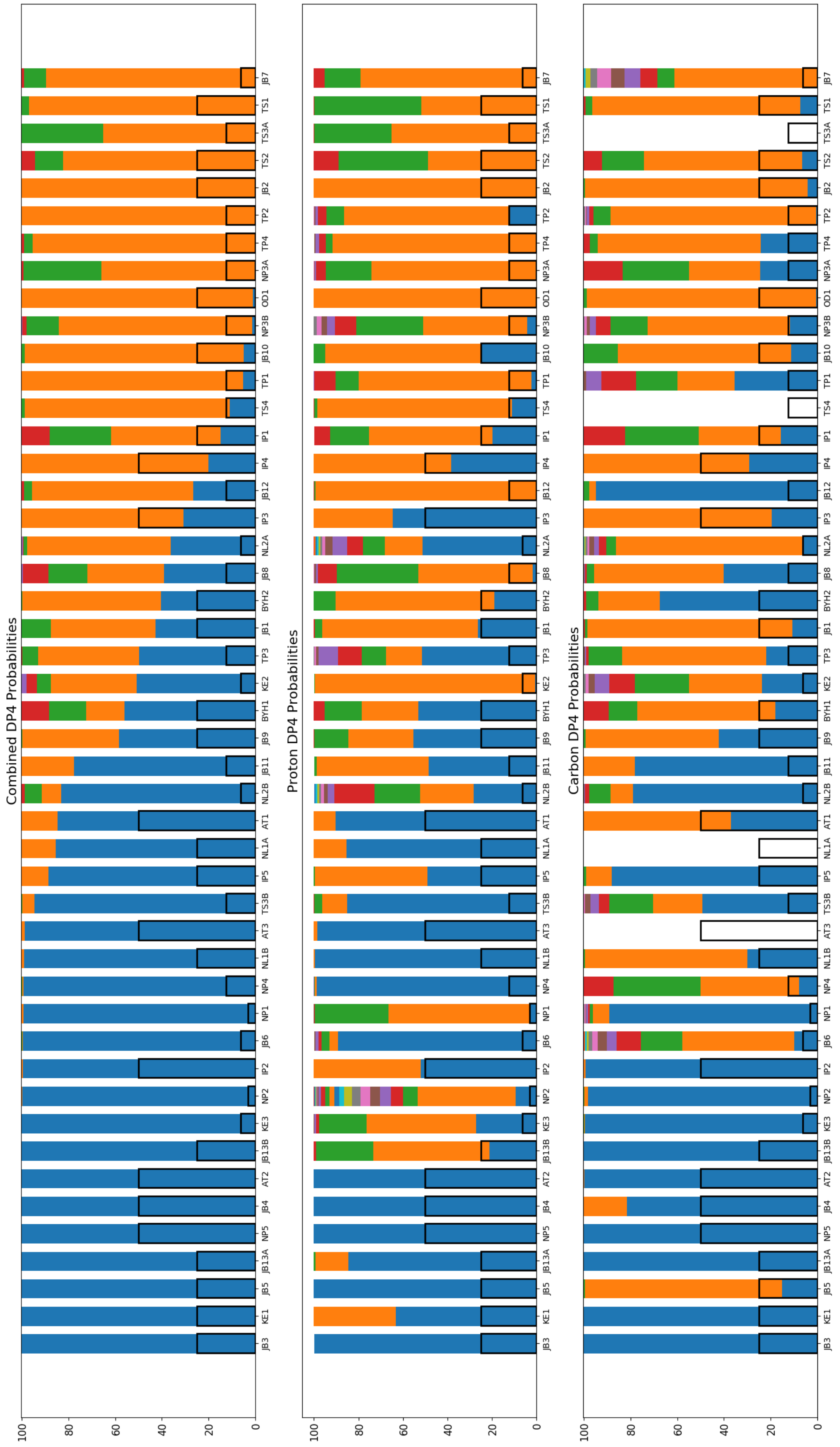

Figure 65: Assignment: DP4-AI, Conditions: Geometries = DFT Optimised B3LYP/6-31g(d), NMR = mPW1PW91/6-311g(d), Single Point energy = mPW1PW91/6-311g(d), Statistical Model = 3 Gaussian

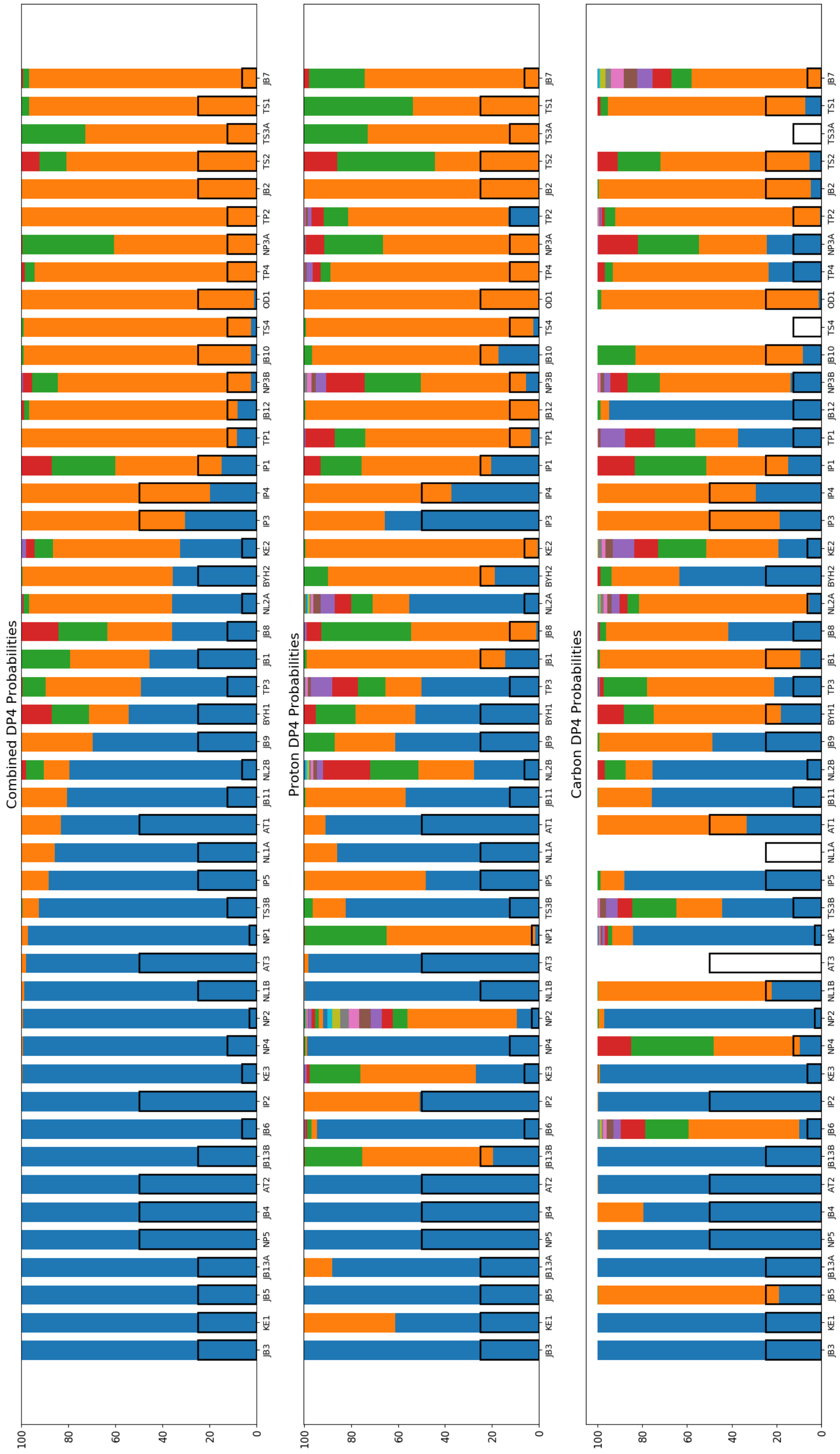

Figure 66: Assignment: DP4-AI, Conditions: Geometries = DFT Optimised B3LYP/6-31g(d), NMR = mPW1PW91/6-311g(d), Single Point energy = mPW1PW91/6-311g(d), Statistical Model = cross validation

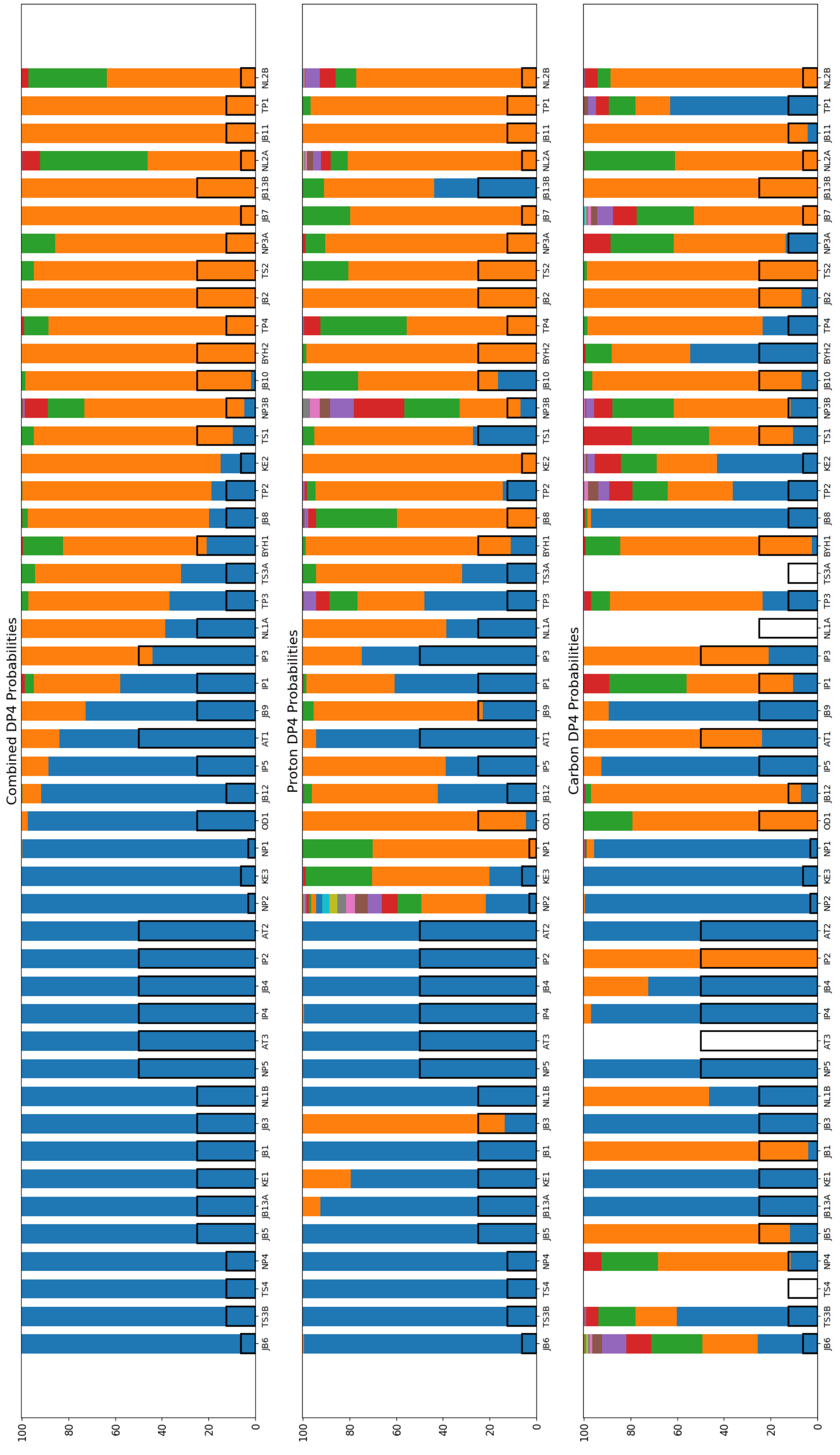

Figure 67: Assignment: DP4-AI, Conditions: Geometries = DFT Optimised B3LYP/6-31g(d), NMR = mPW1PW91/6-311g(d), Single Point energy = M062X/Def2TZVP, Statistical Model = External

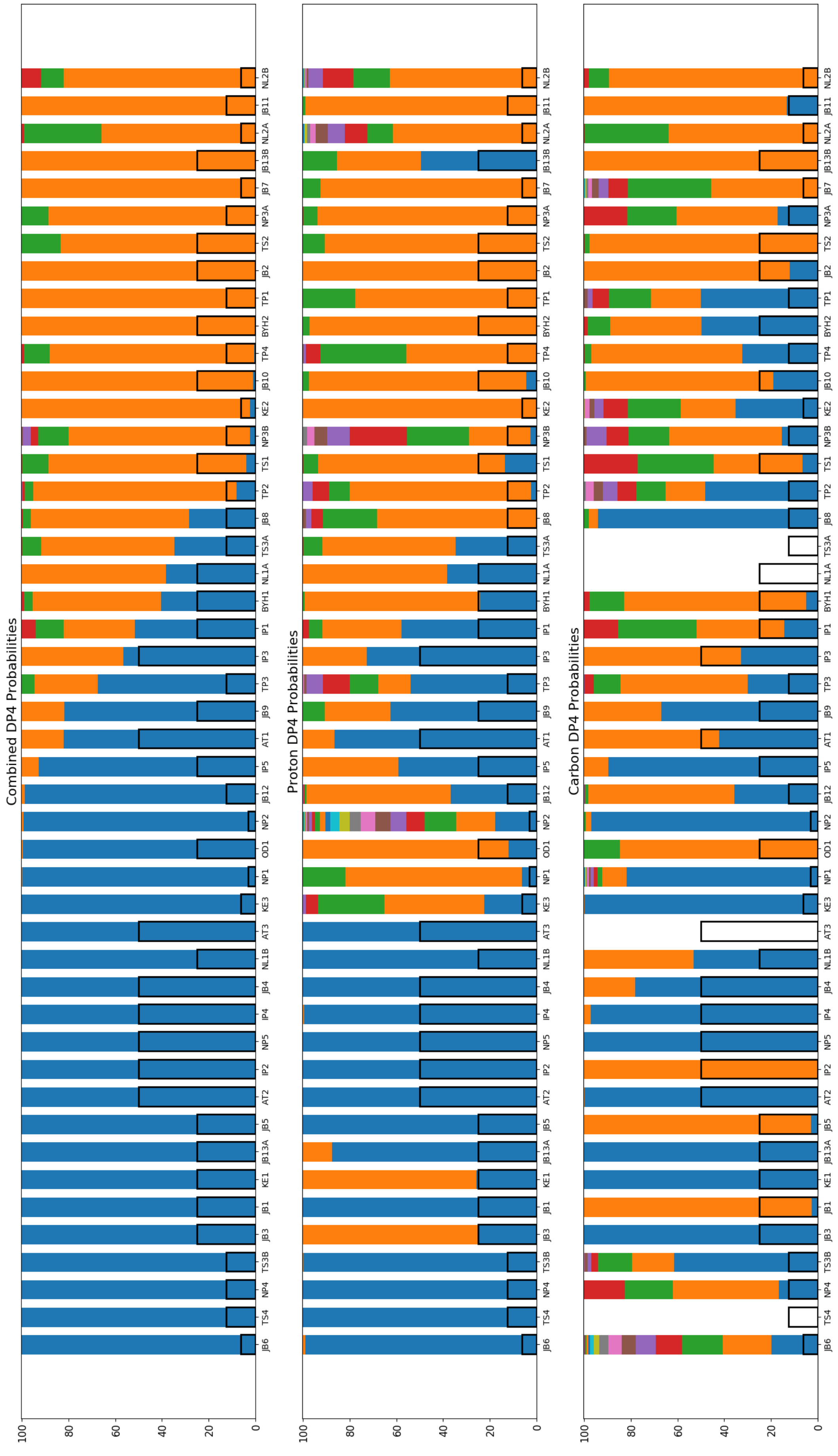

Figure 68: Assignment: DP4-AI, Conditions: Geometries = DFT Optimised B3LYP/6-31g(d), NMR = mPW1PW91/6-311g(d), Single Point energy = M062X/Def2TZVP, Statistical Model = 1 Gaussian

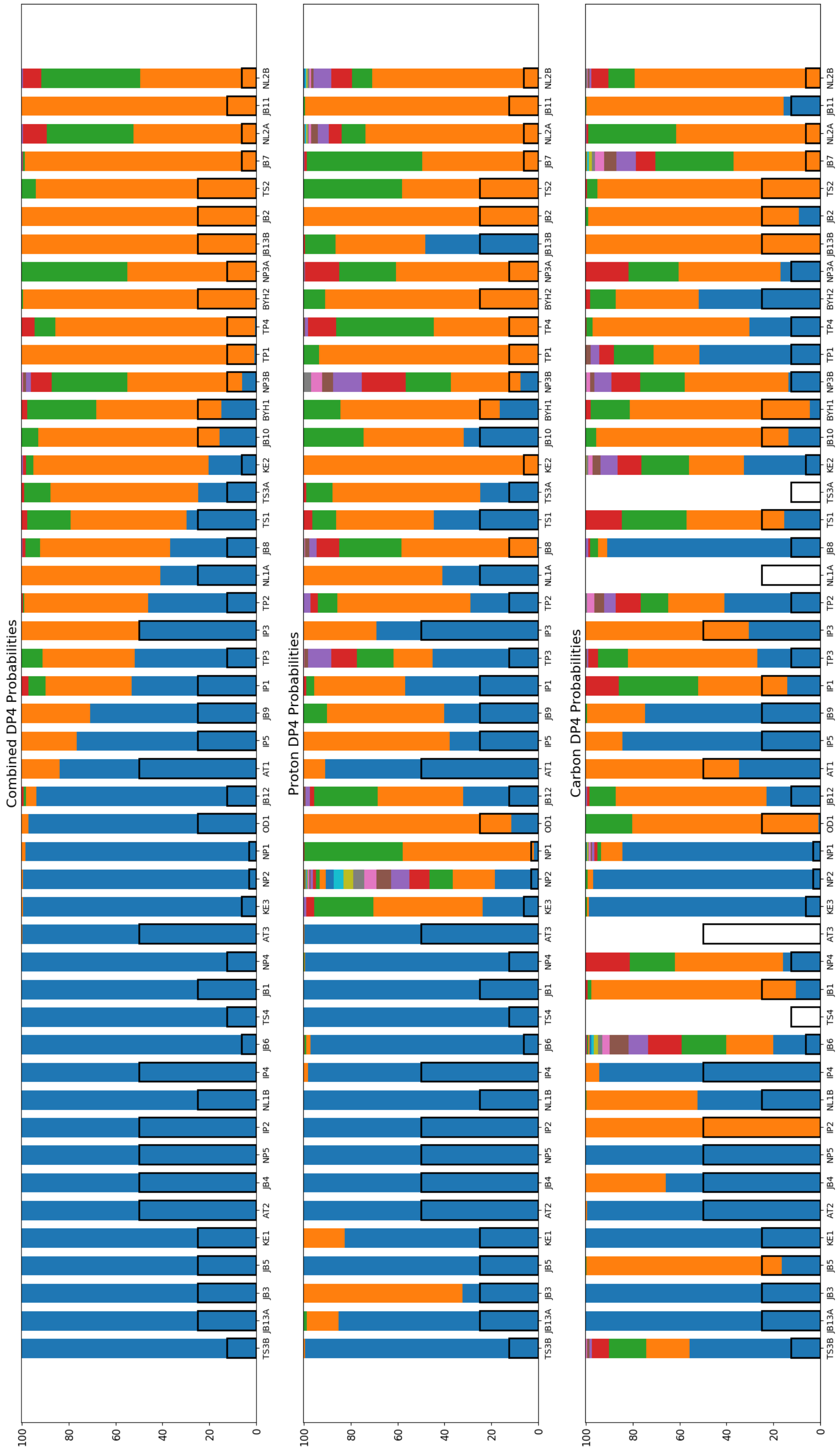

Figure 69: Assignment: DP4-AI, Conditions: Geometries = DFT Optimised B3LYP/6-31g(d), NMR = mPW1PW91/6-311g(d), Single Point energy = M062X/Def2TZVP, Statistical Model = 2 Gaussian

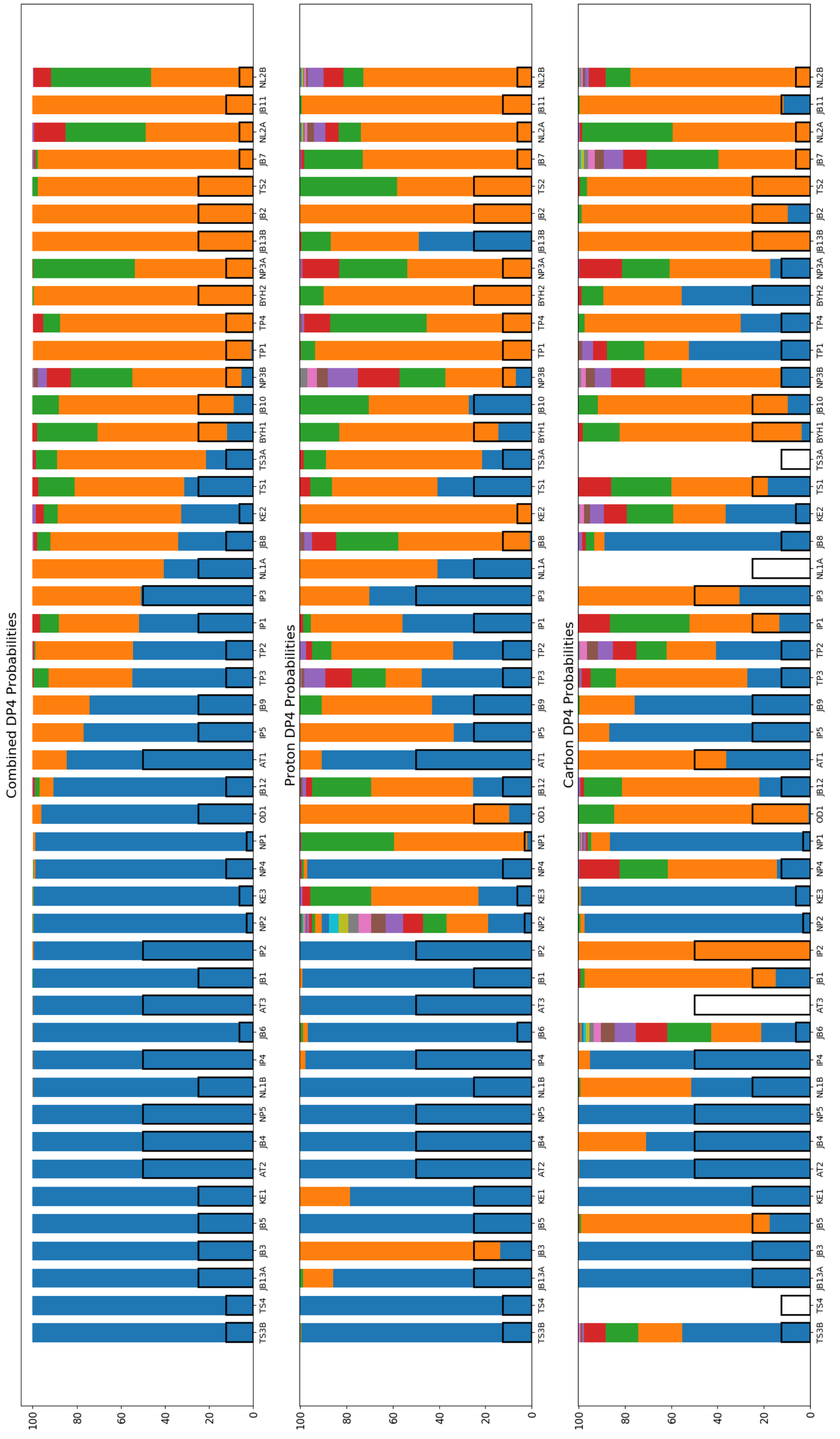

Figure 70: Assignment: DP4-AI, Conditions: Geometries = DFT Optimised B3LYP/6-31g(d), NMR = mPW1PW91/6-311g(d),Single Point energy = M062X/Def2TZVP, Statistical Model = 3 Gaussian

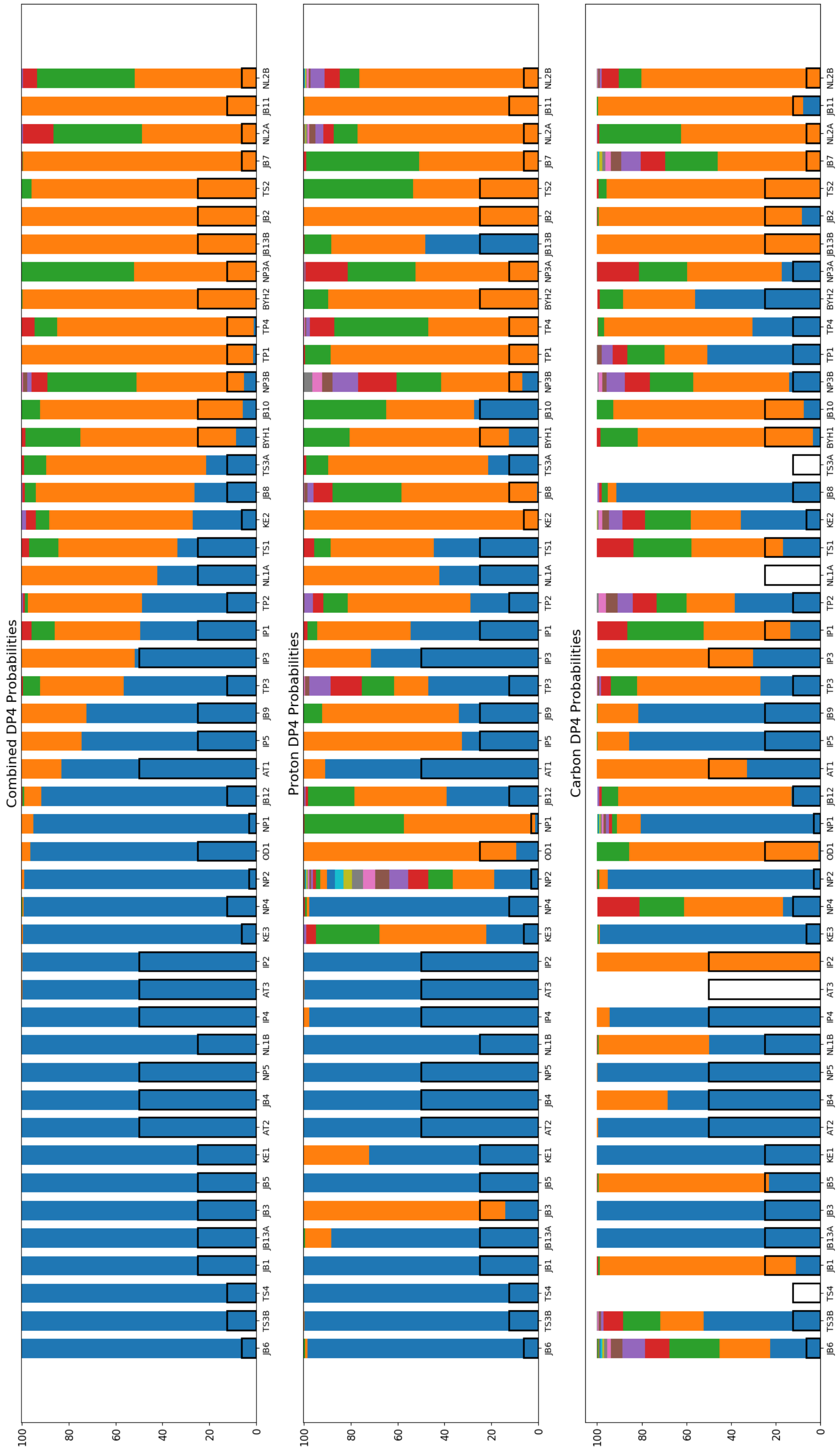

Figure 71: Assignment: DP4-AI, Conditions: Geometries = DFT Optimised B3LYP/6-31g(d), NMR = mPW1PW91/6-311g(d),Single Point energy = M062X/Def2TZVP, Statistical Model = cross validation

4 Assigned Spectra

Listed Below are the spectra processed and assigned by DP4-AI in this study. These assignments correspond to the highest level of theory tested and the cross validation statistical model

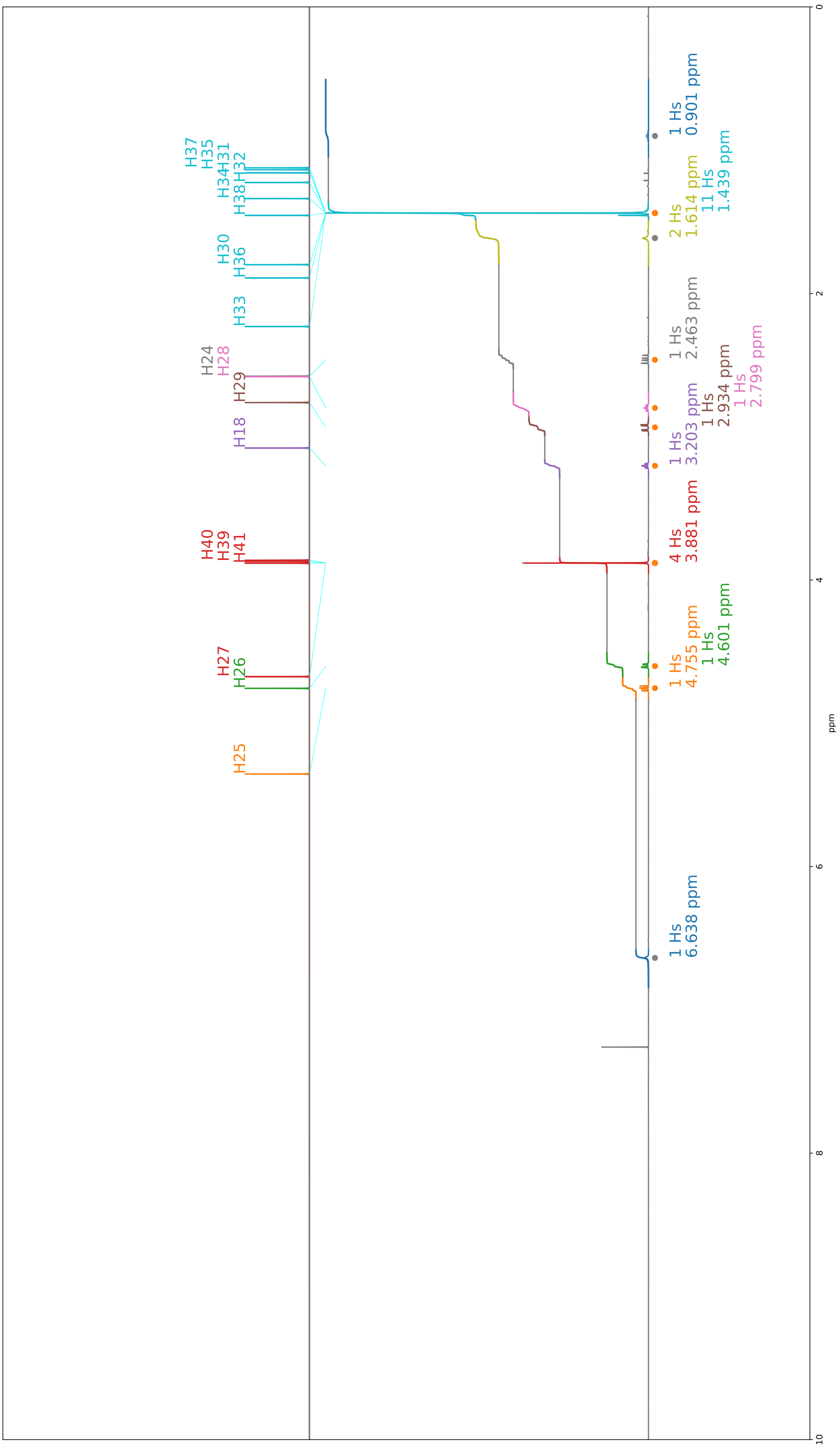

Figure 72: JB1 Proton Spectra

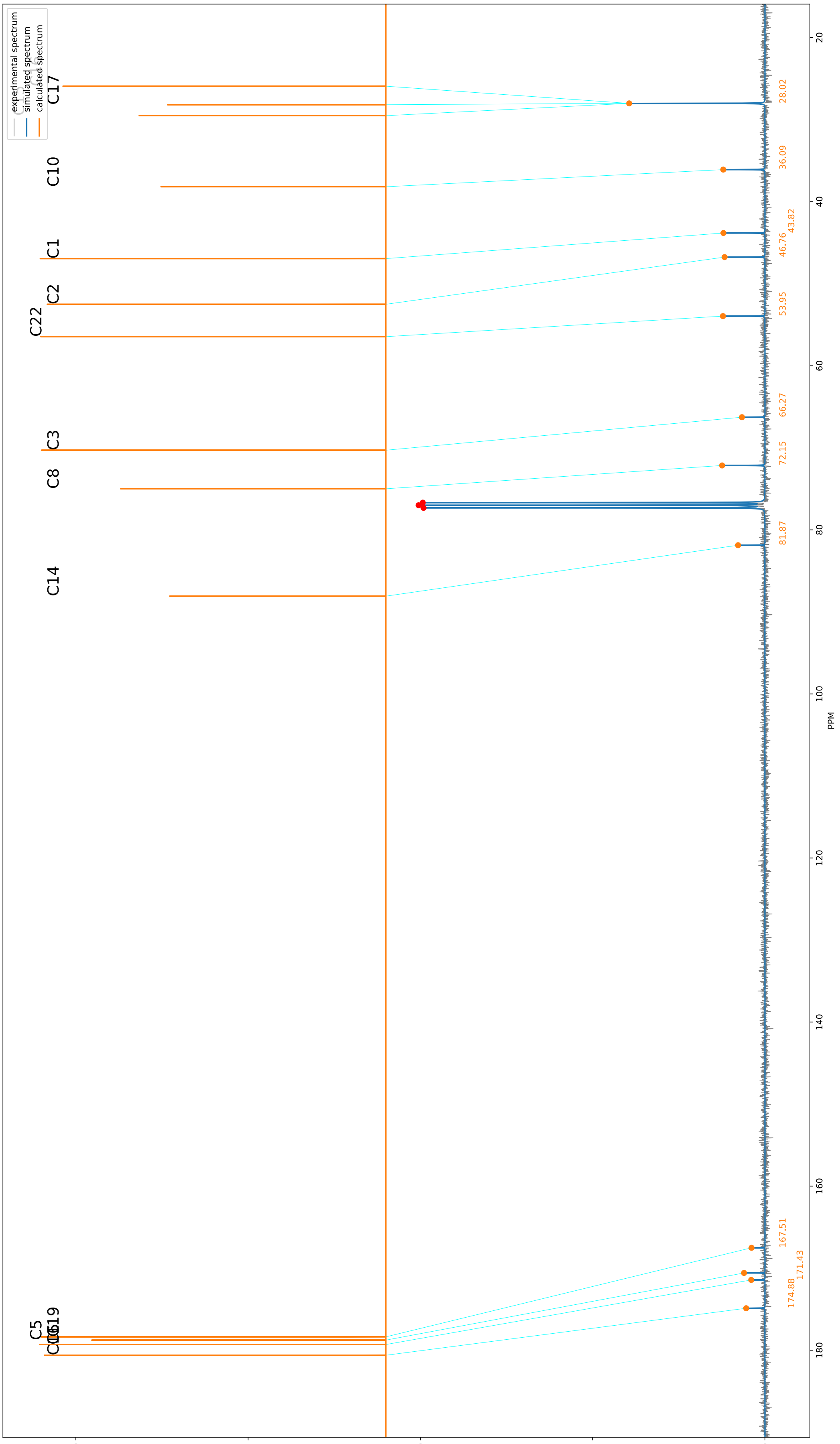

Figure 73: JB1 Carbon Spectra

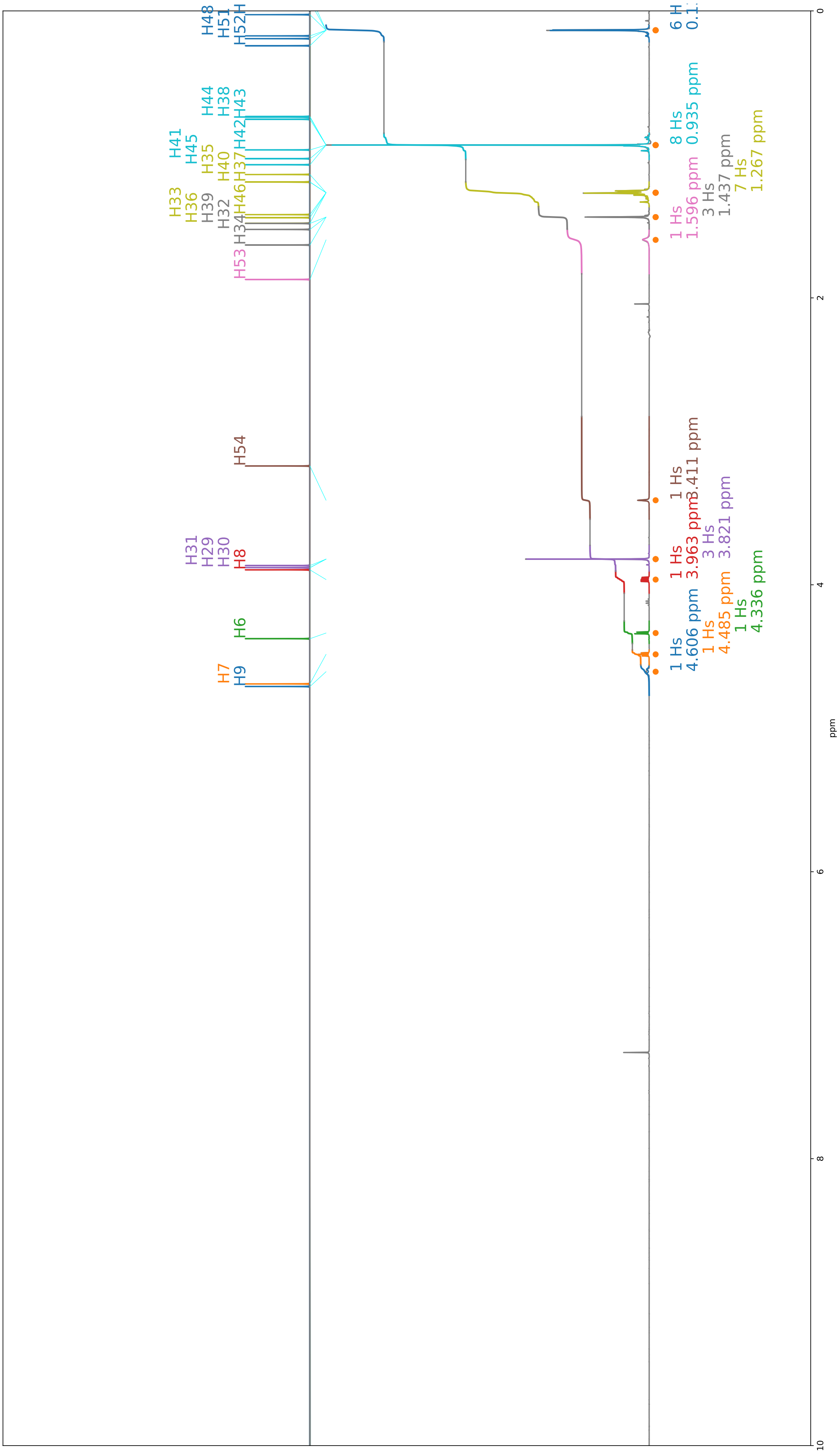

Figure 74: KE2 Proton Spectra

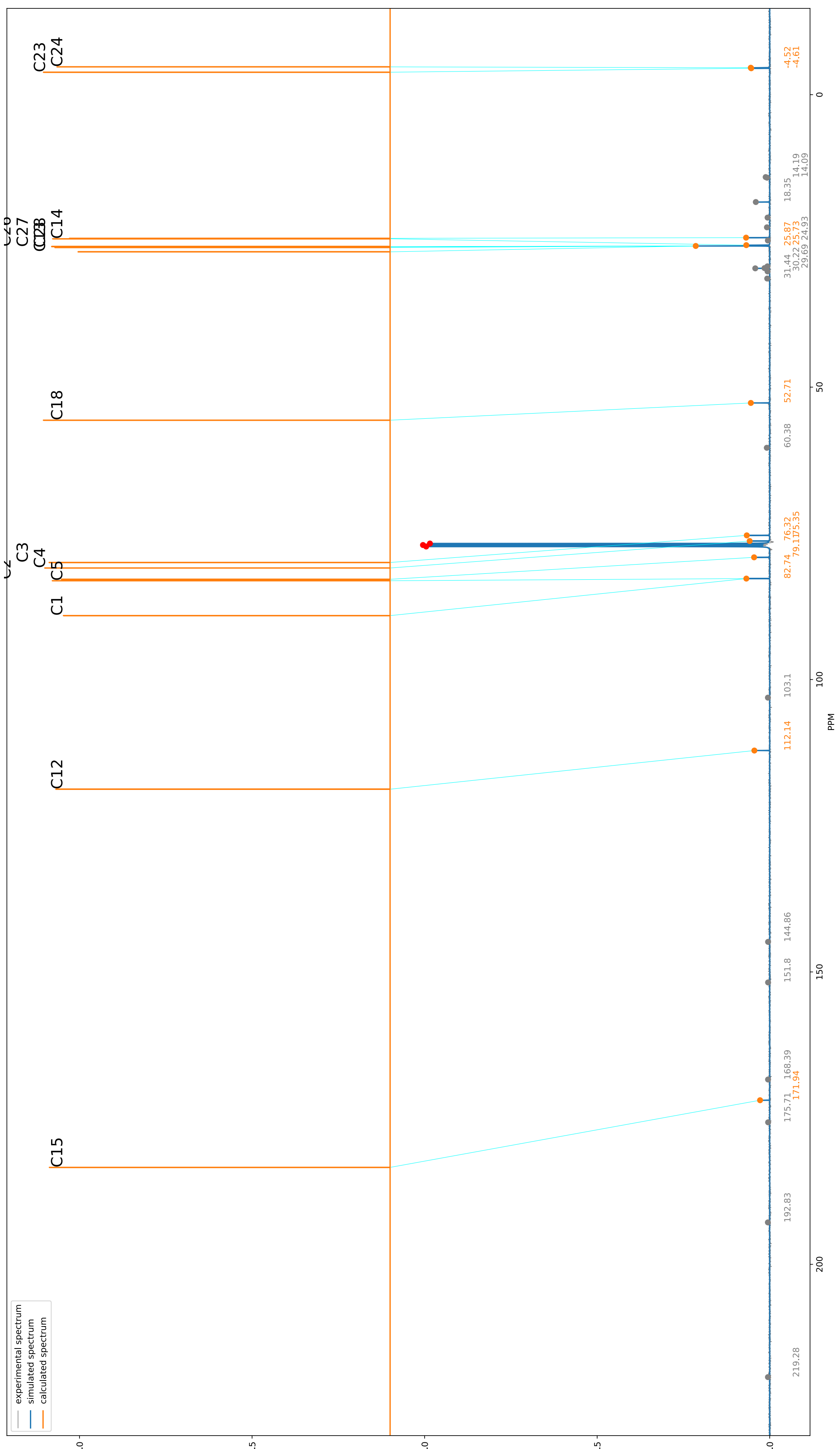

Figure 75: KE2 Carbon Spectra

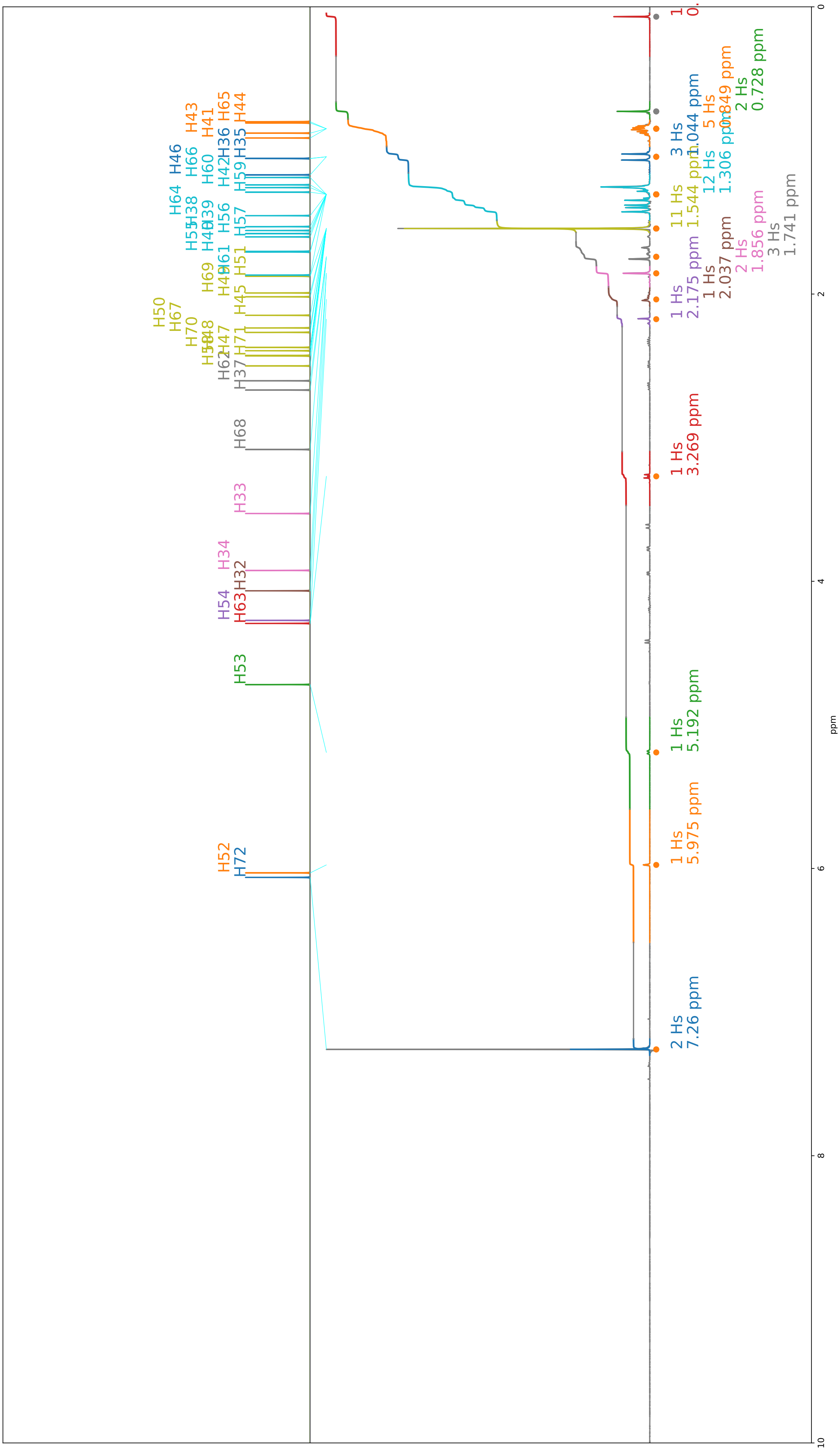

Figure 76: NL2B Proton Spectra

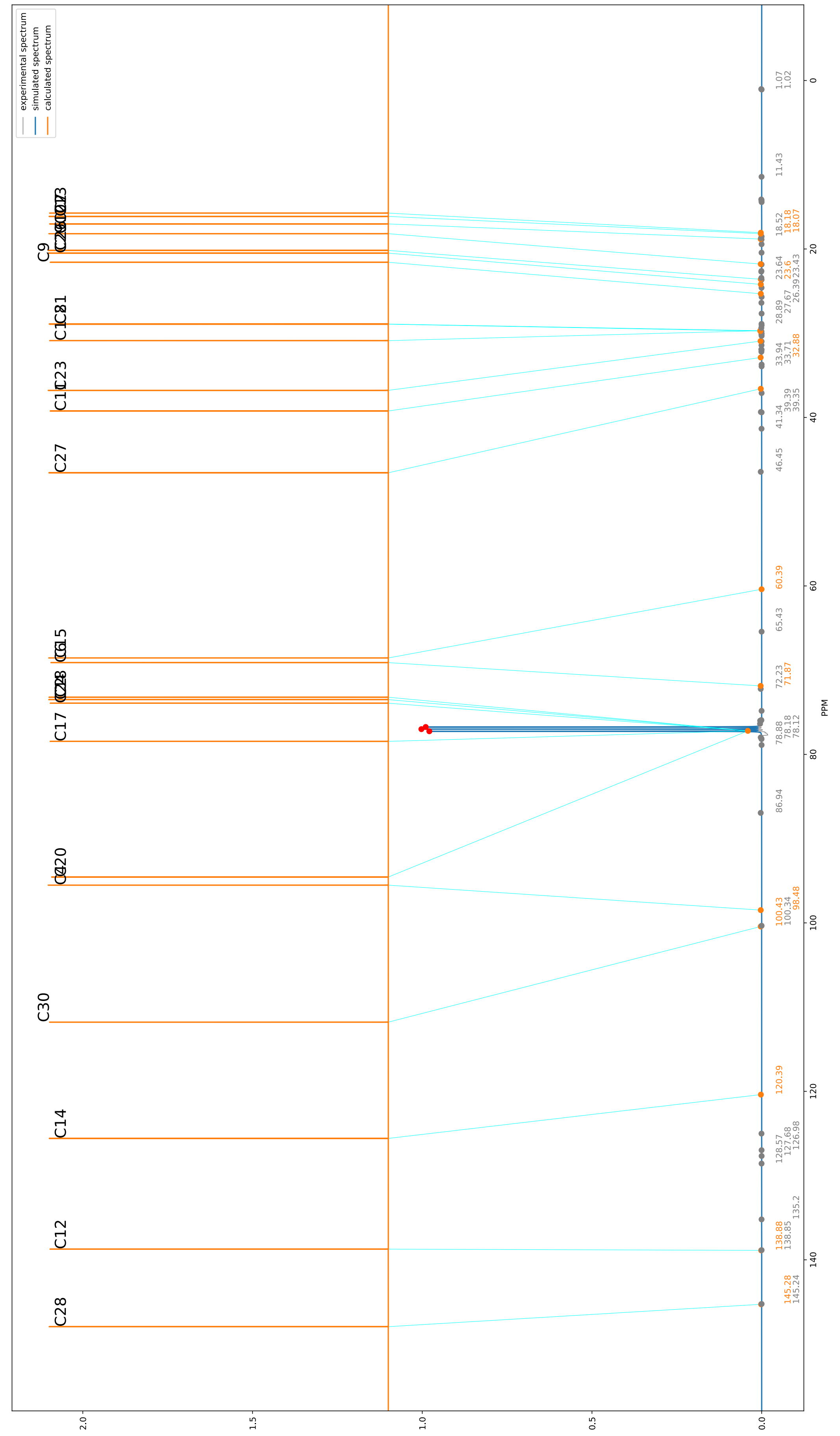

Figure 77: NL2B Carbon Spectra

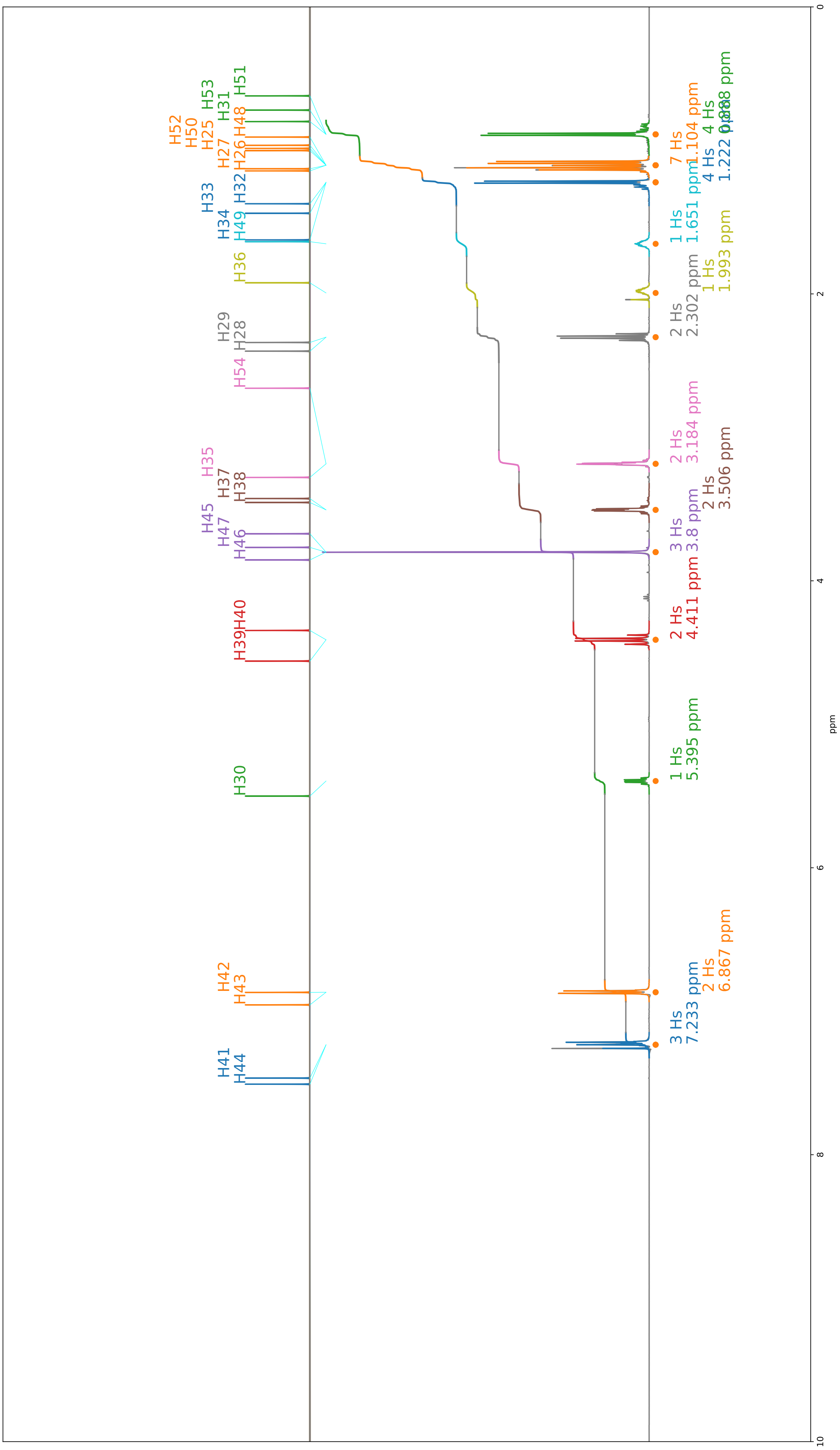

Figure 78: TP1 Proton Spectra

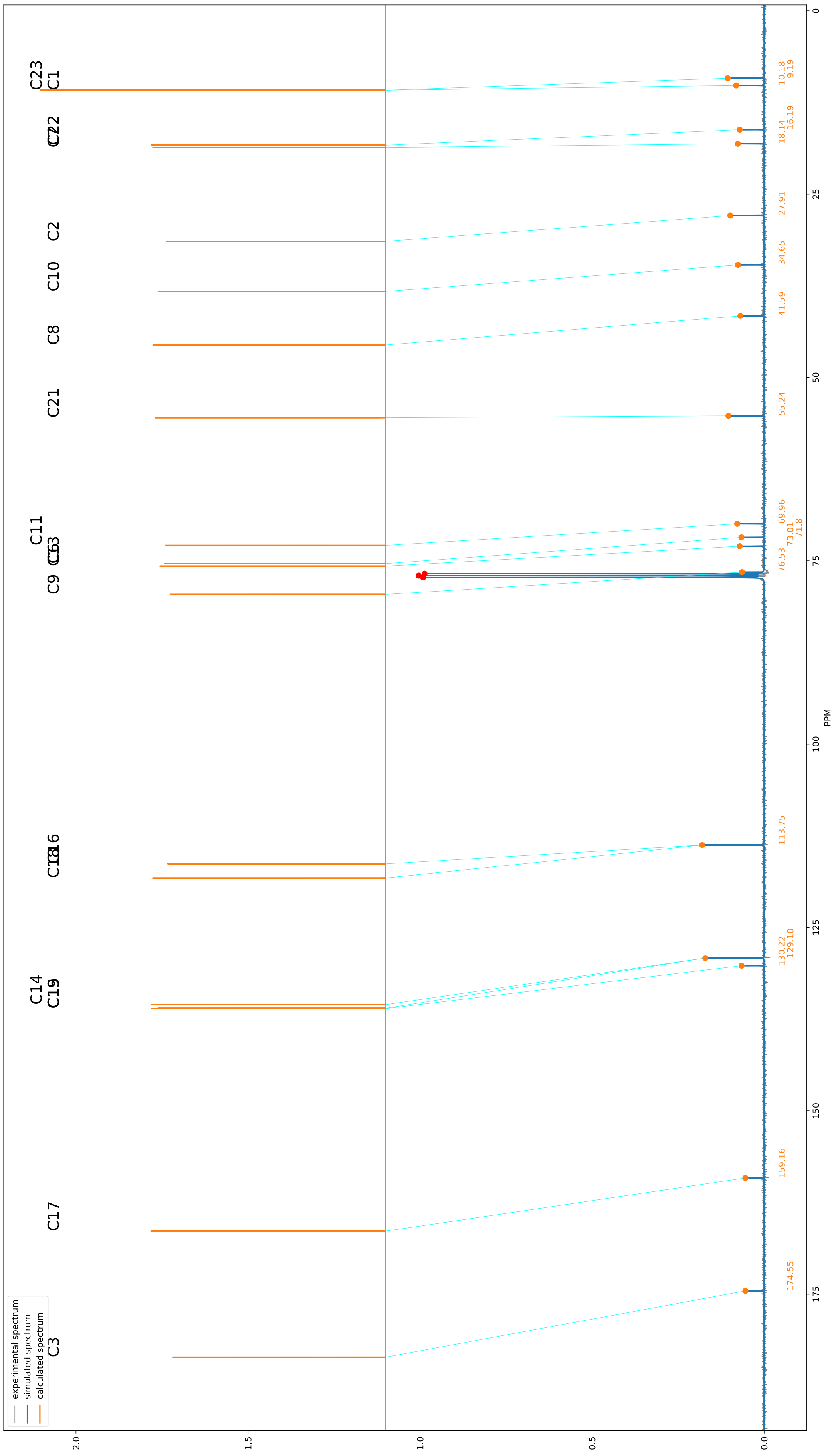

Figure 79: TP1 Carbon Spectra

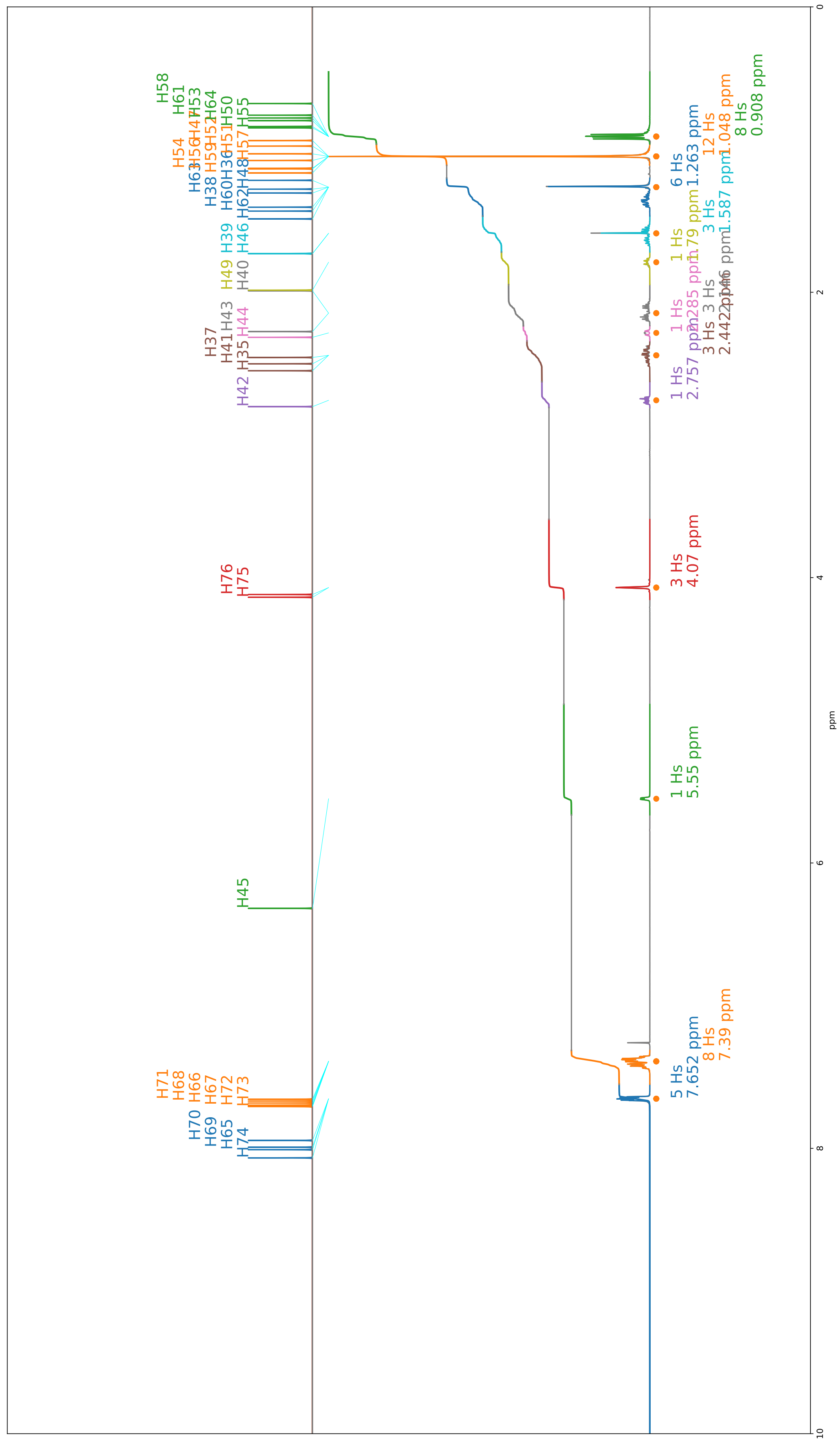

Figure 80: JB10 Proton Spectra

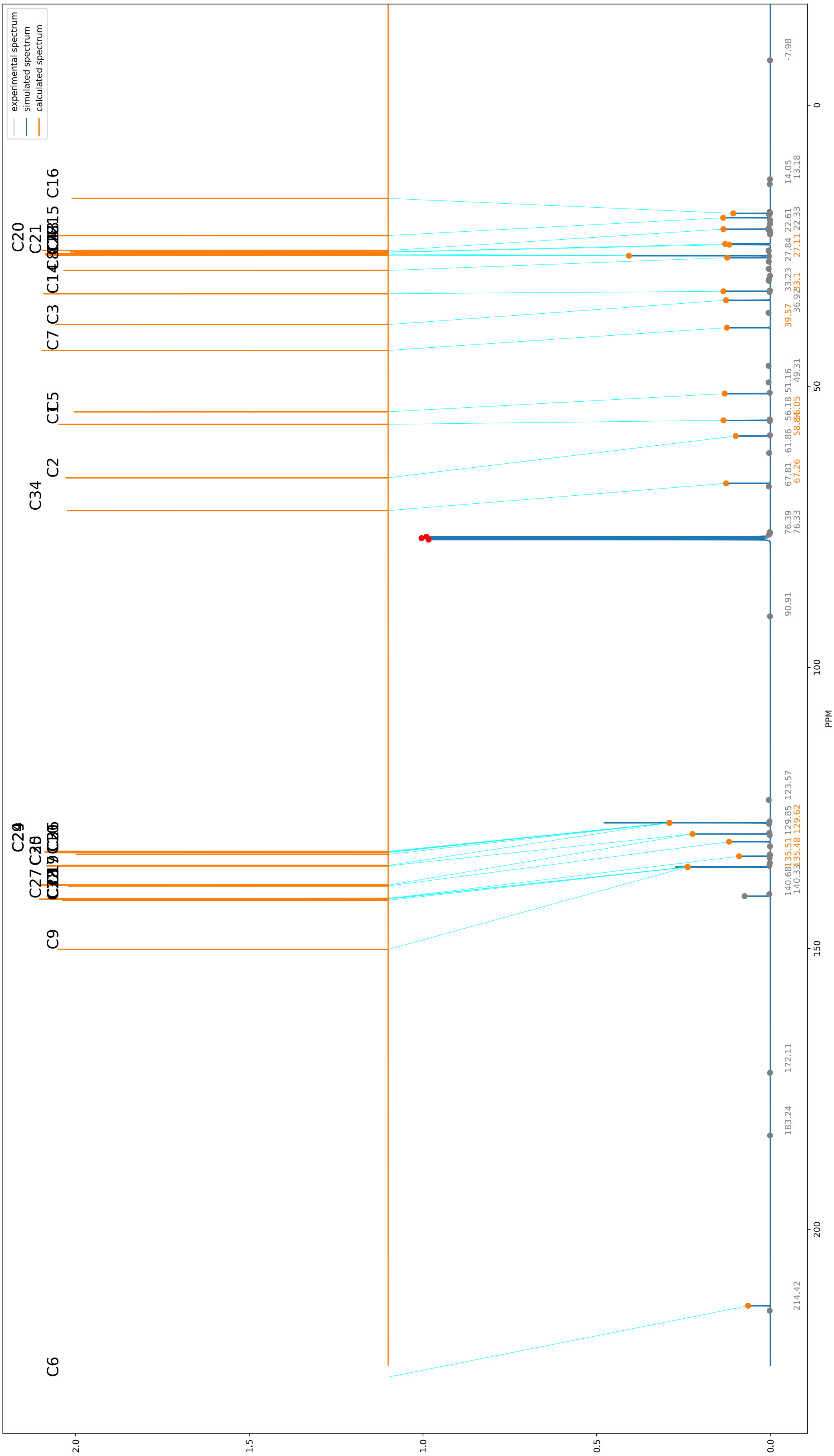

Figure 81: JB10 Carbon Spectra

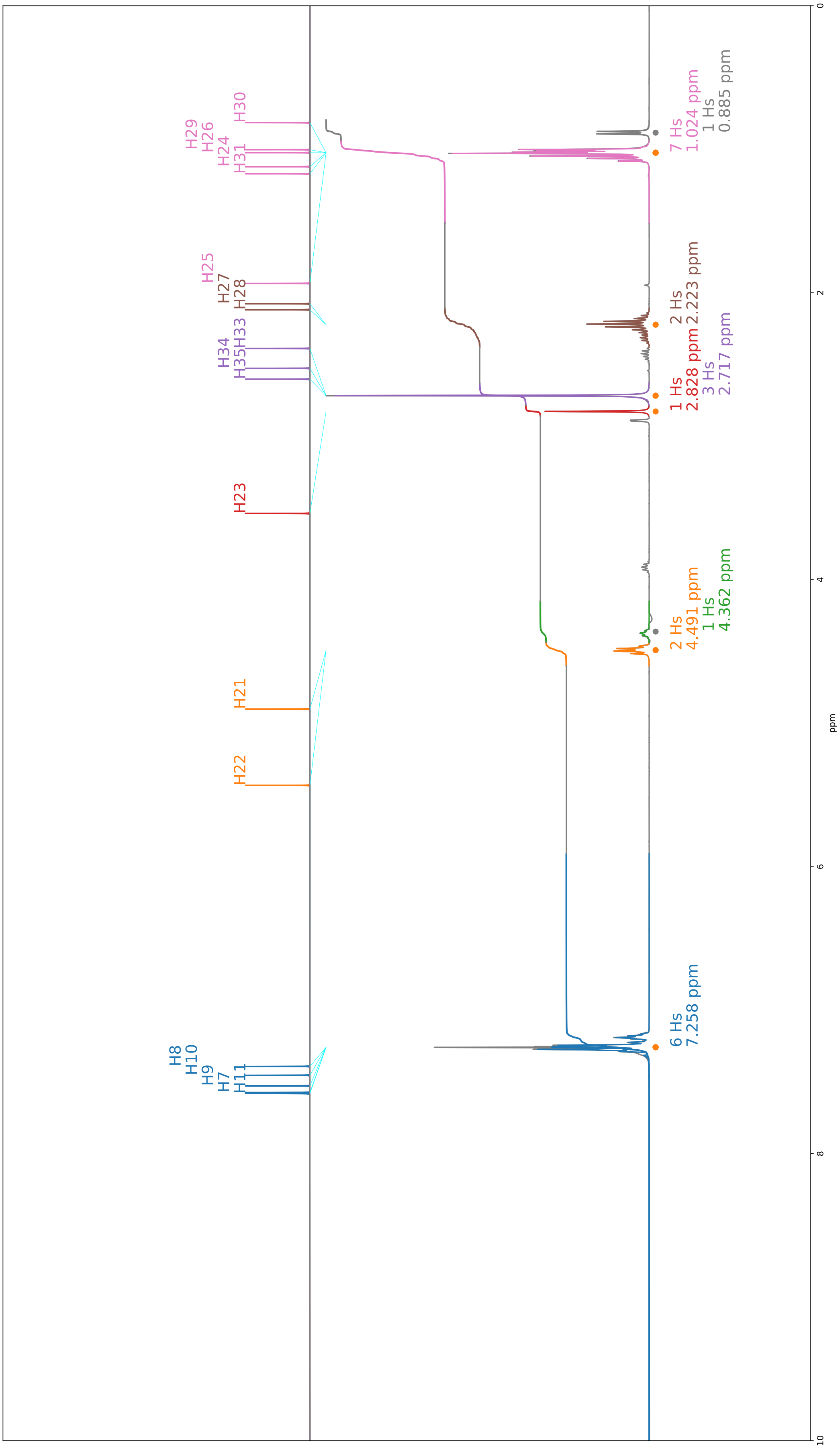

Figure 82: IP2 Proton Spectra

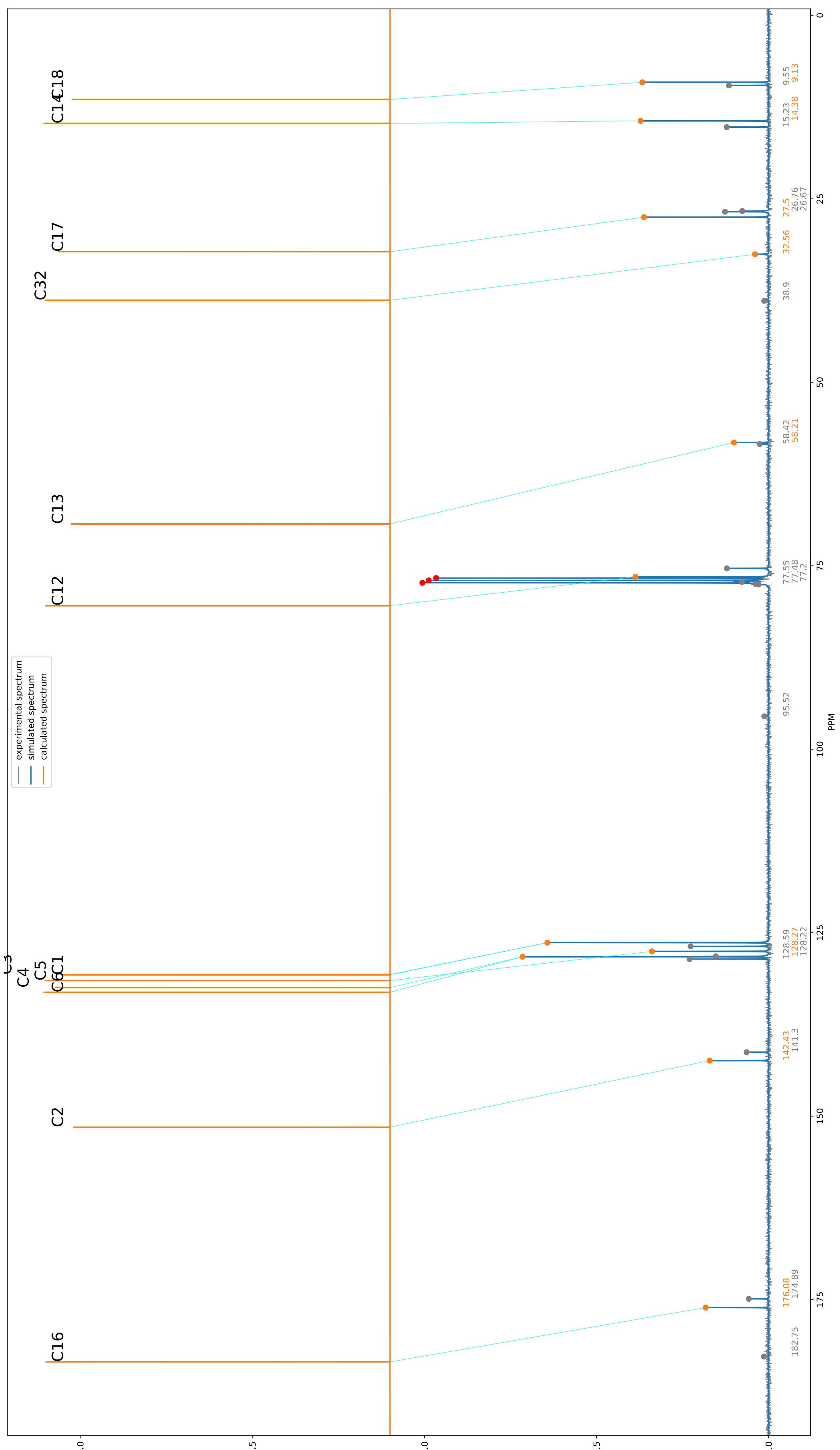

Figure 83: IP2 Carbon Spectra

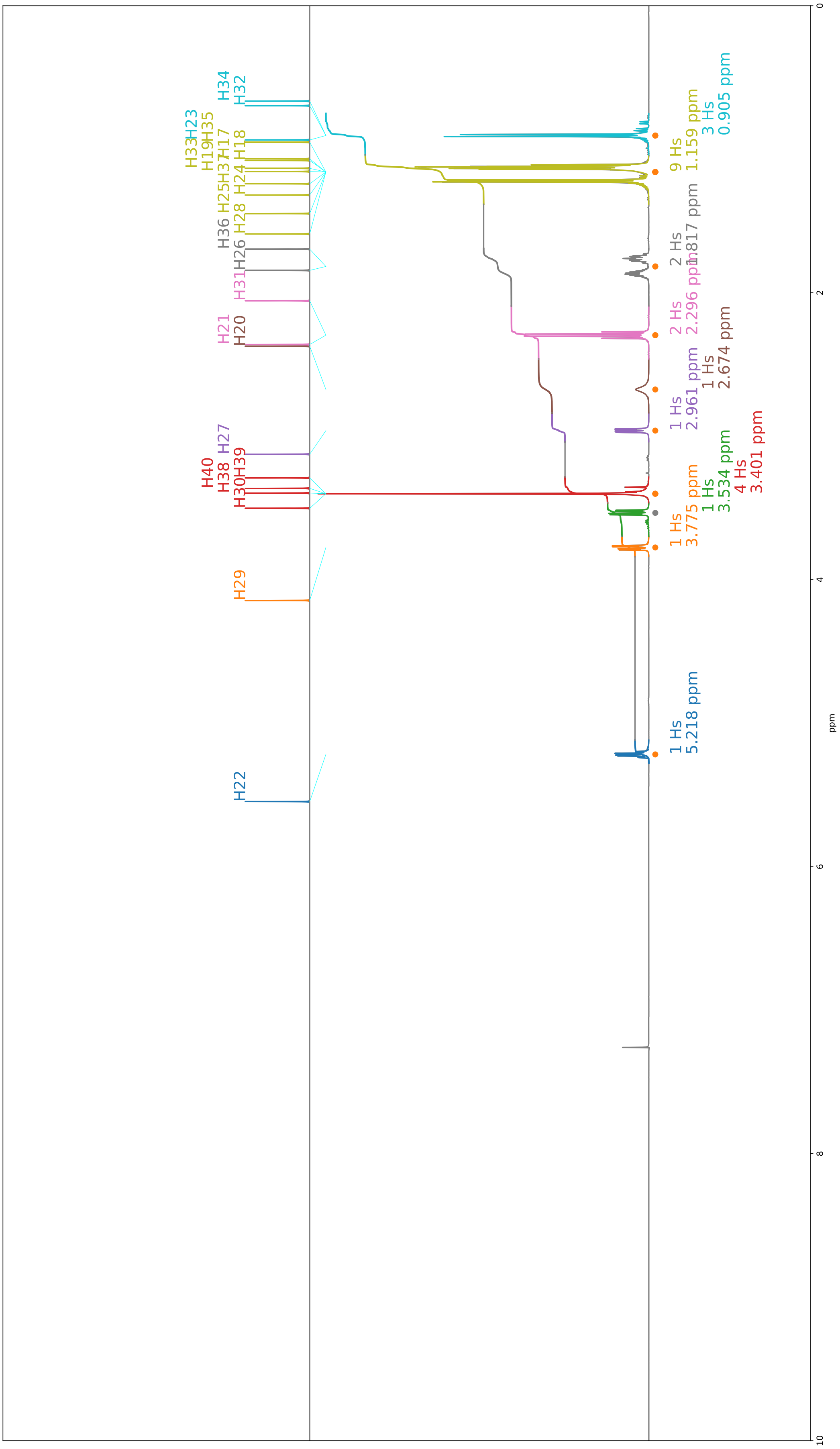

Figure 84: TP4 Proton Spectra

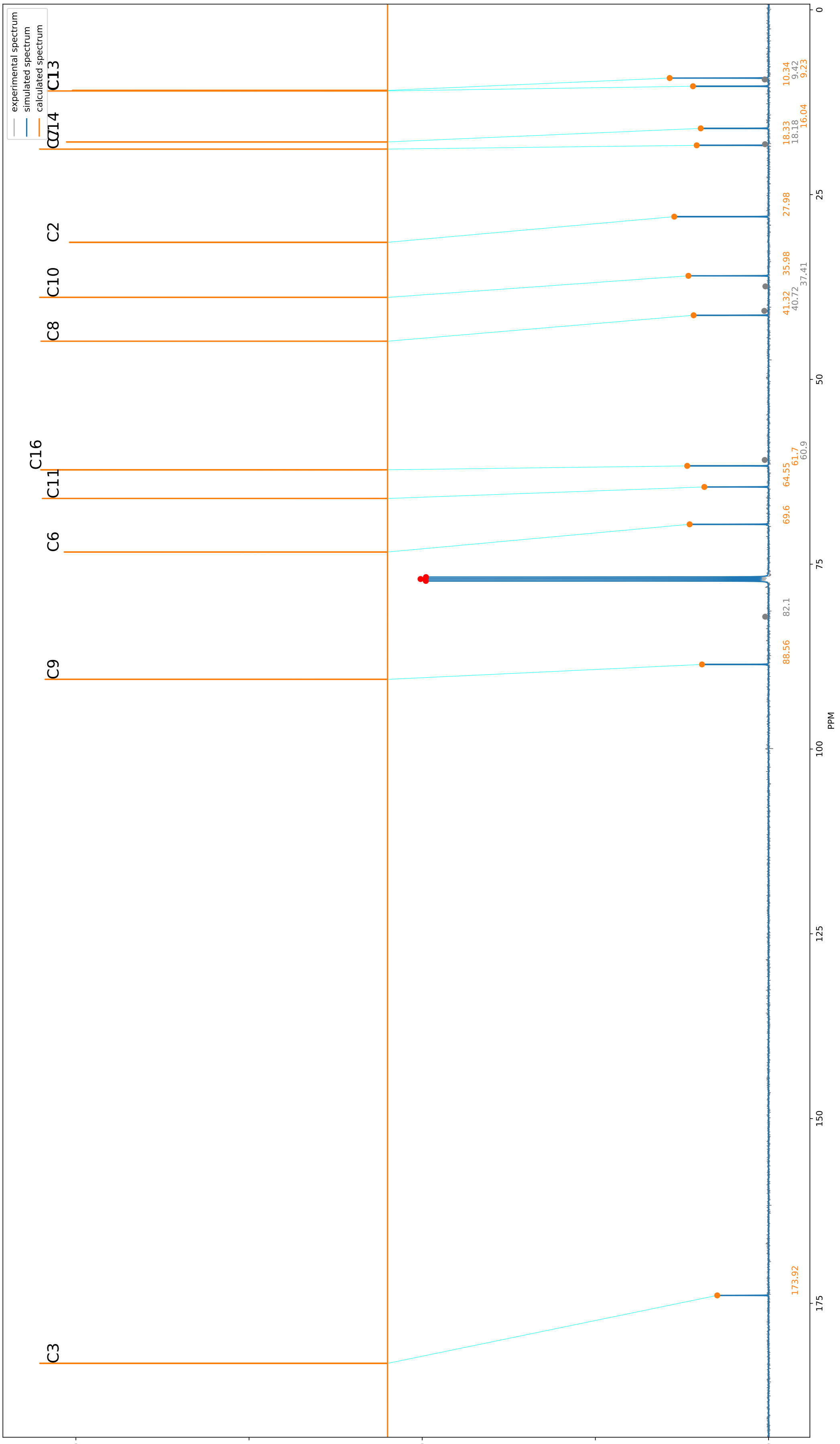

Figure 85: TP4 Carbon Spectra

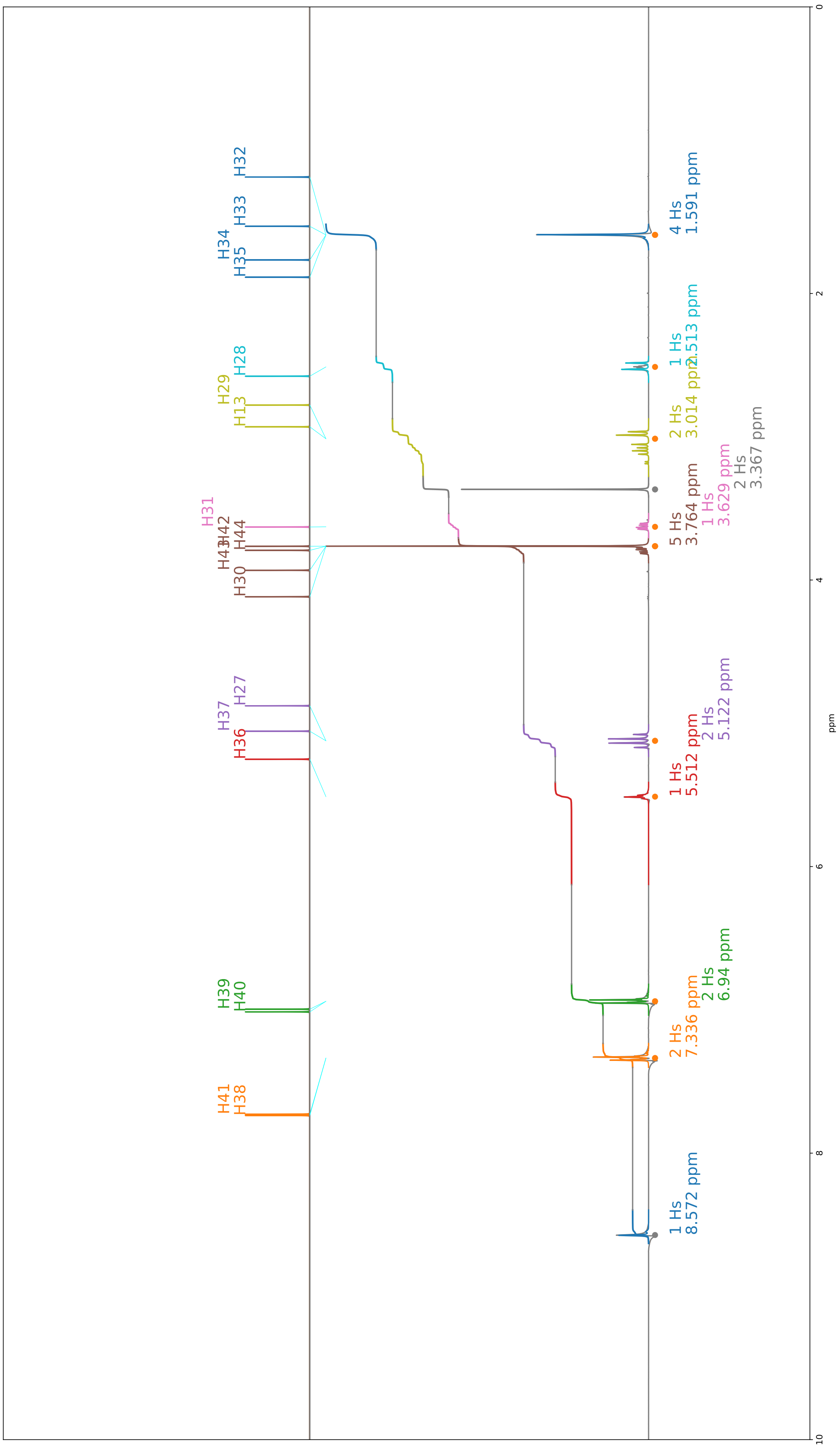

Figure 86: JB5 Proton Spectra

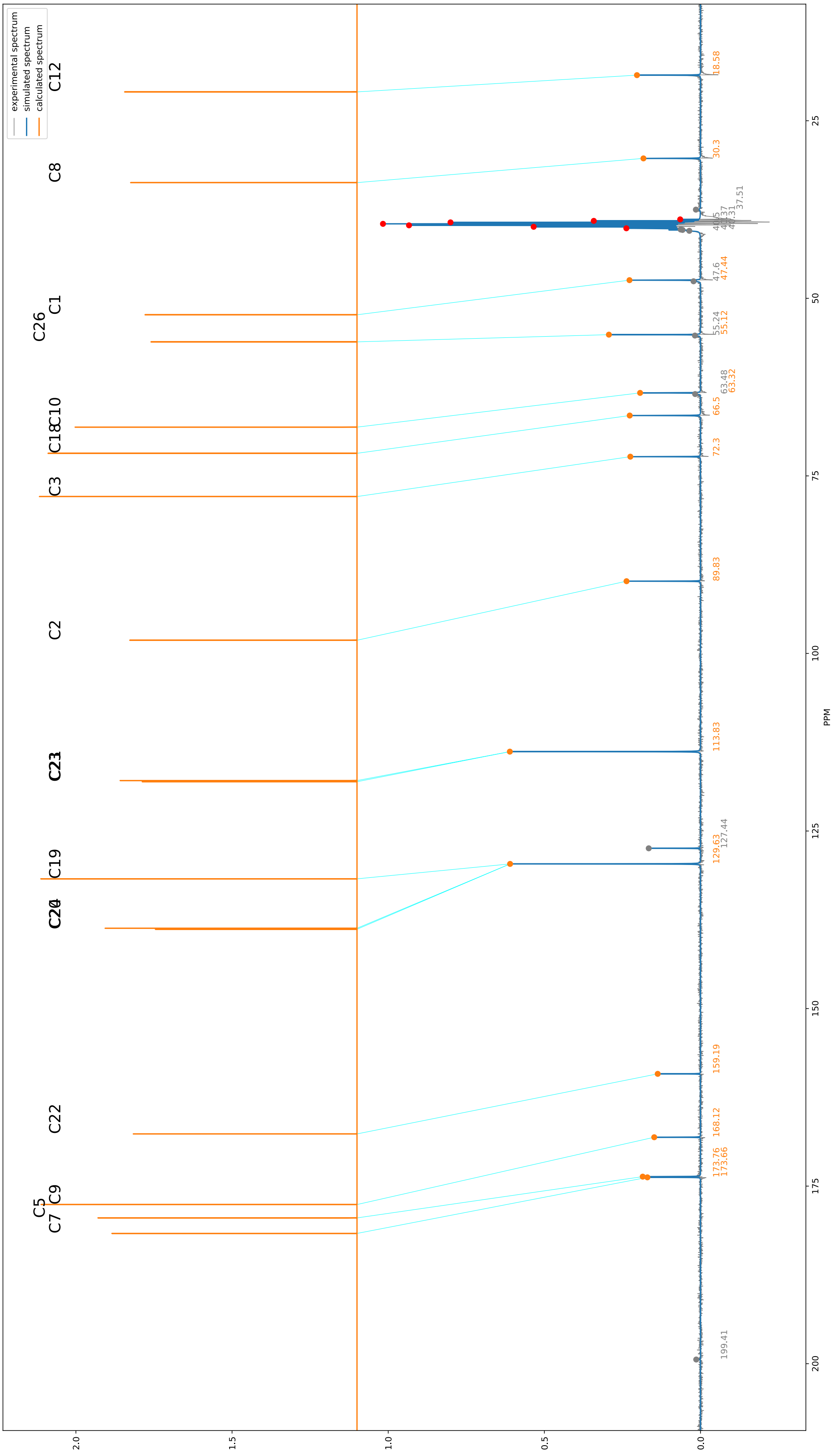

Figure 87: JB5 Carbon Spectra

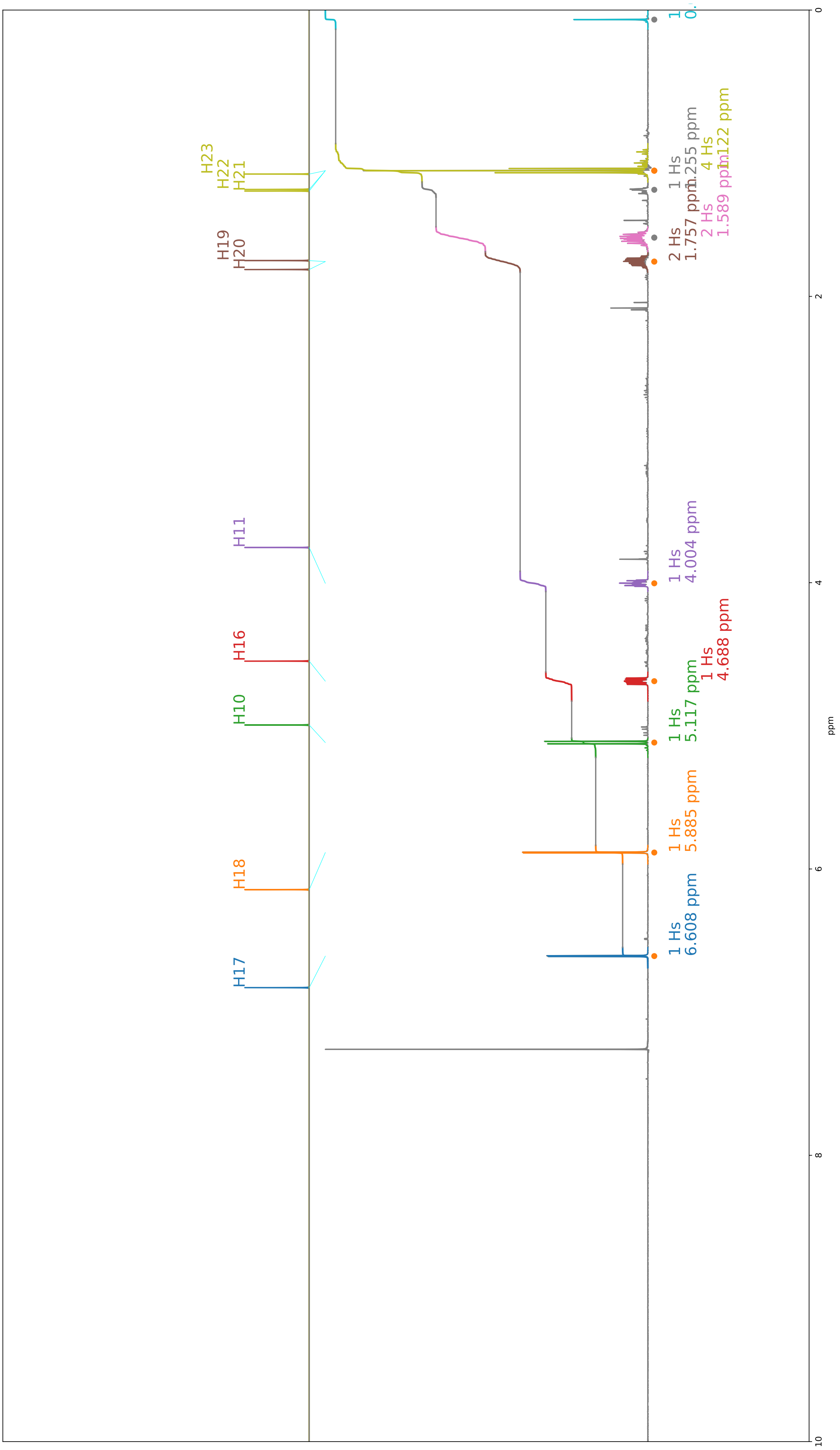

Figure 88: JB13A Proton Spectra

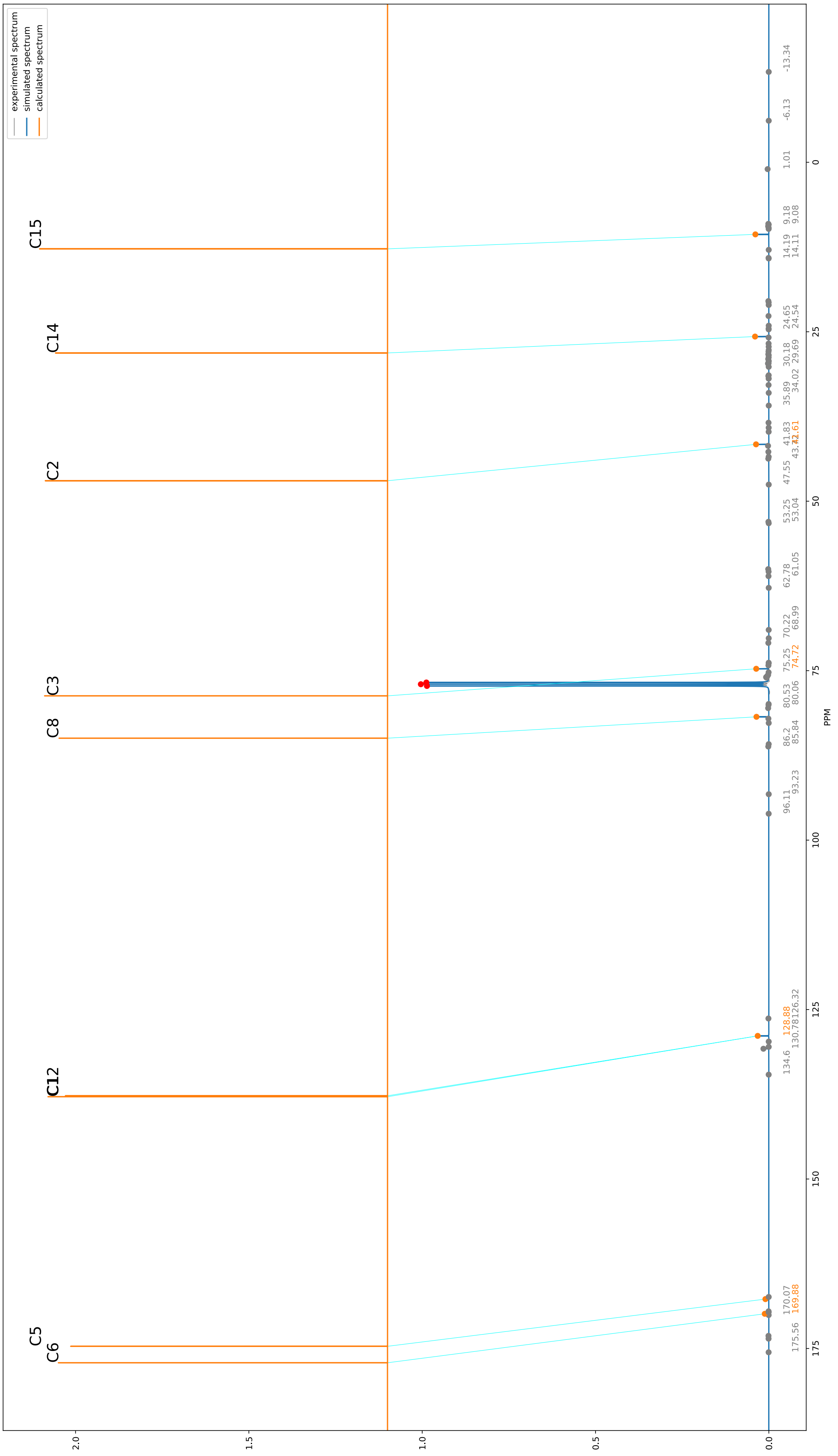

Figure 89: JB13A Carbon Spectra

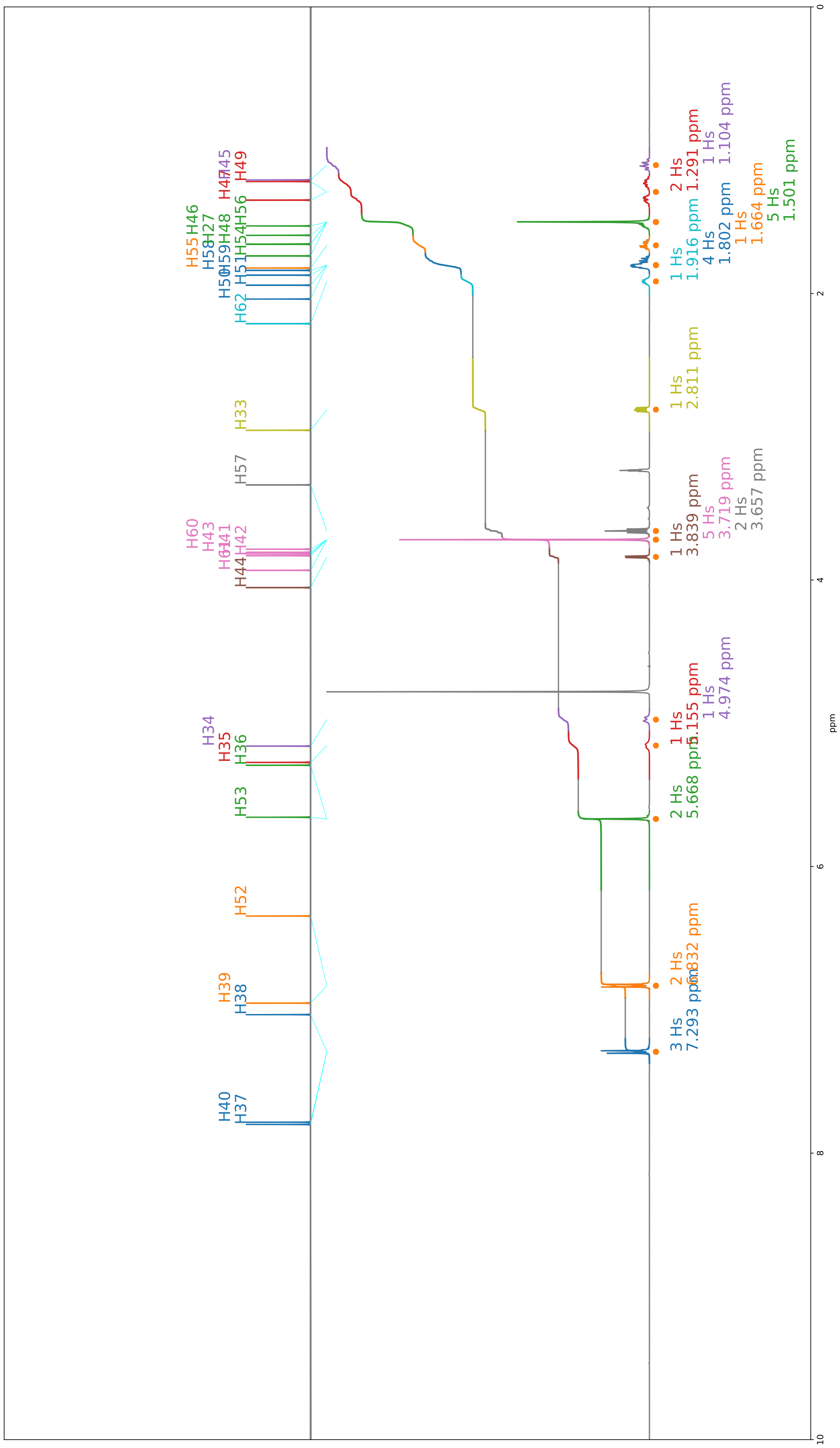

Figure 90: JB7 Proton Spectra

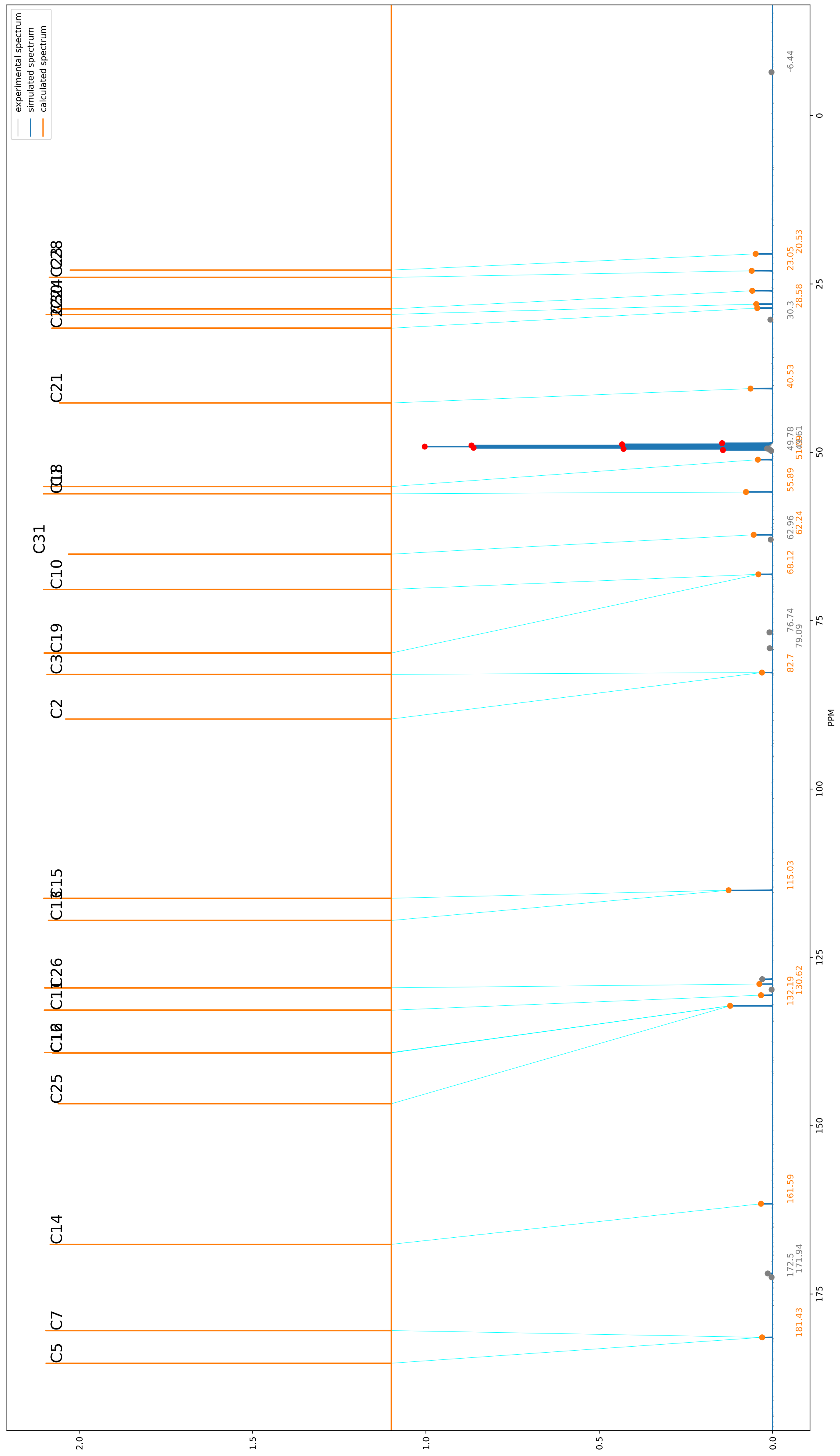

Figure 91: JB7 Carbon Spectra

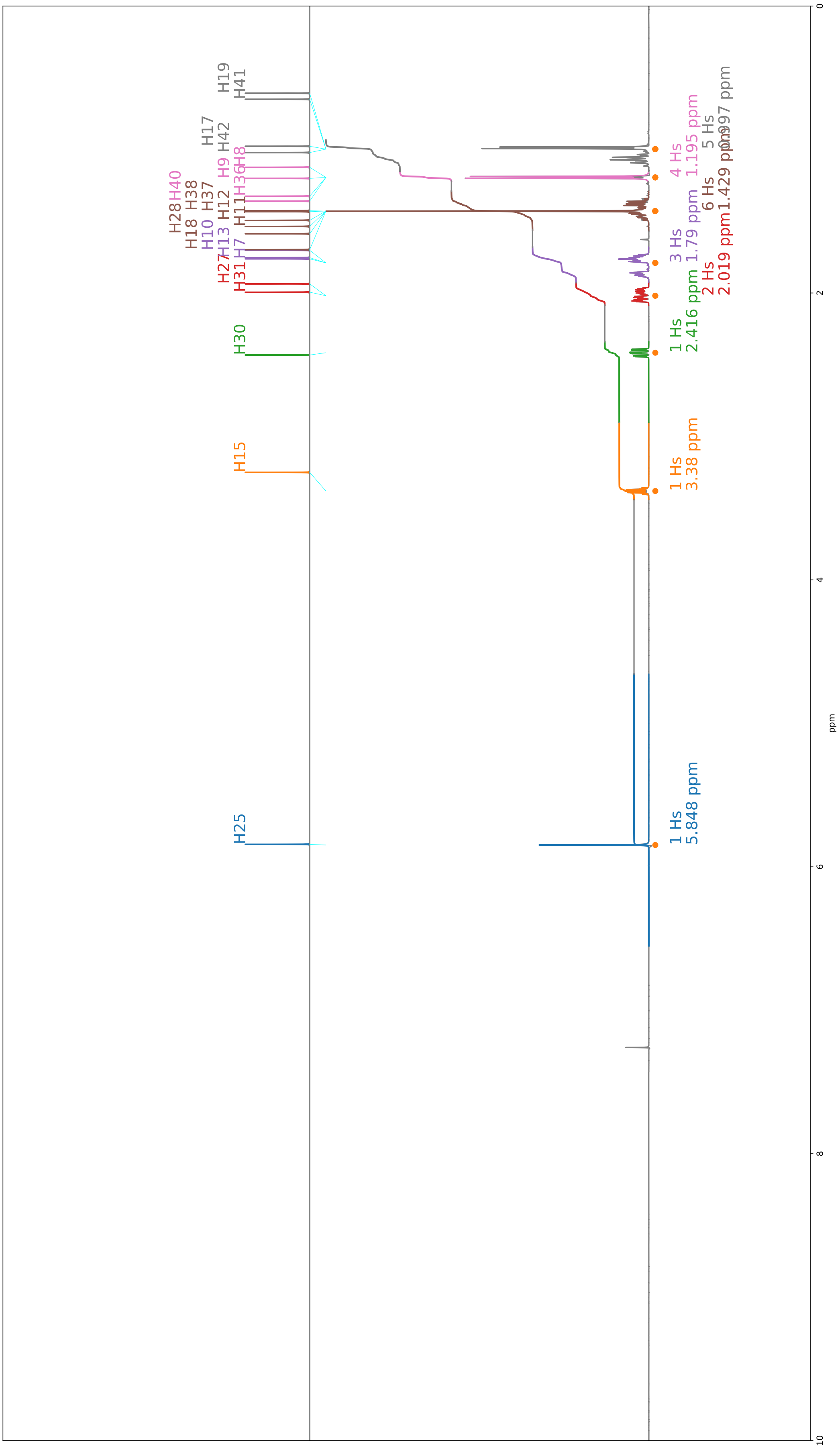

Figure 92: NP1 Proton Spectra

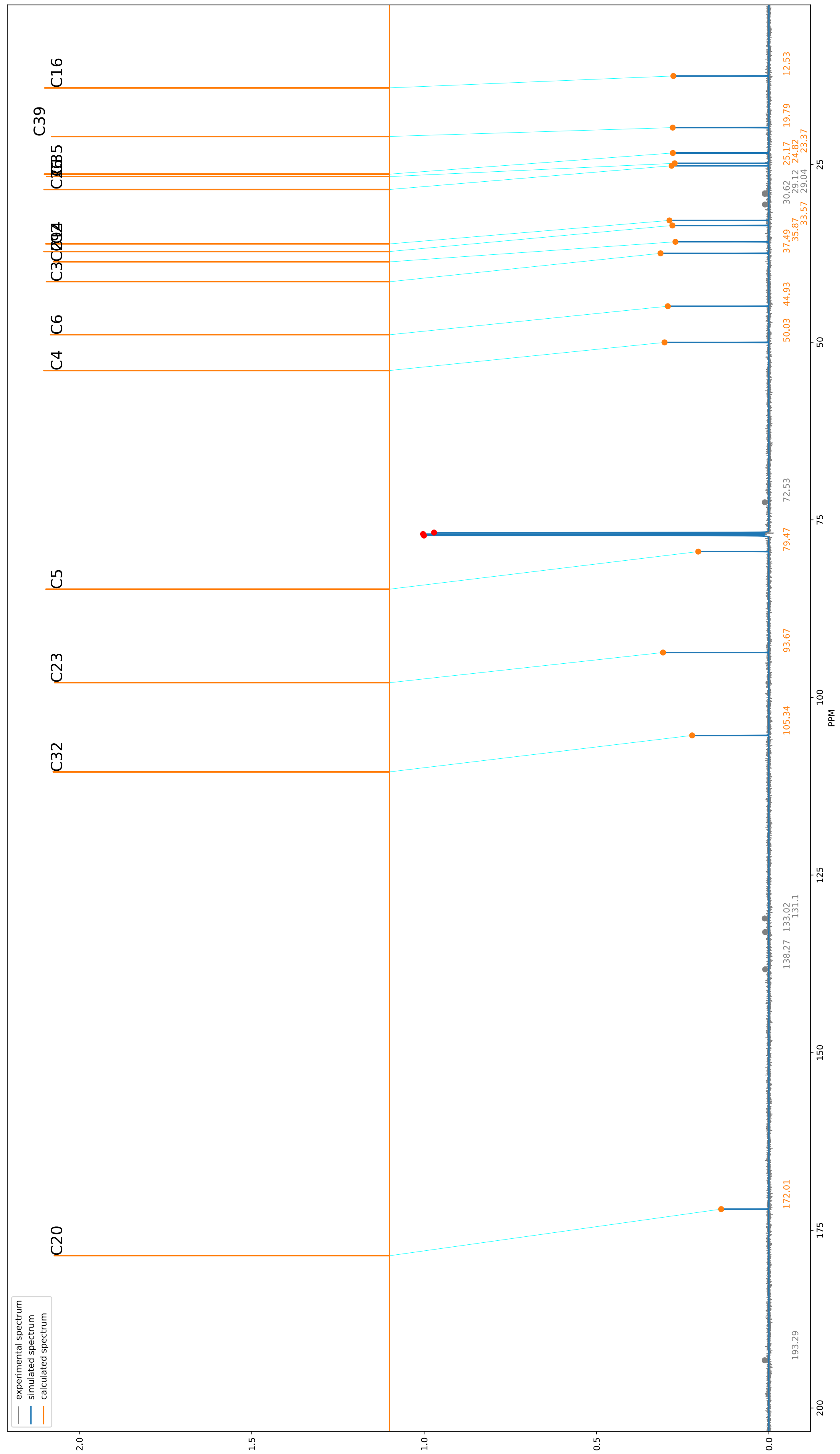

Figure 93: NP1 Carbon Spectra

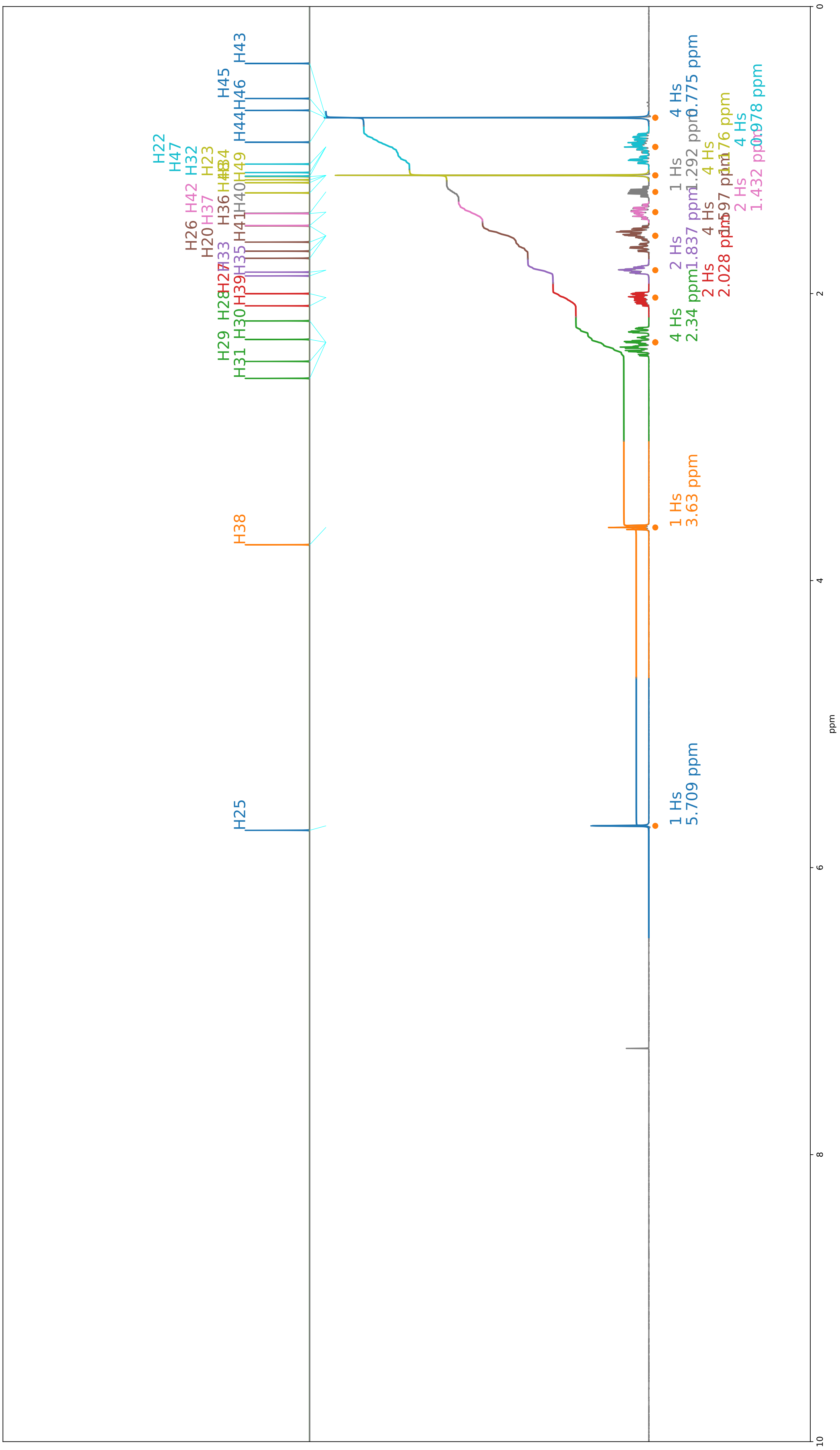

Figure 94: NP2 Proton Spectra

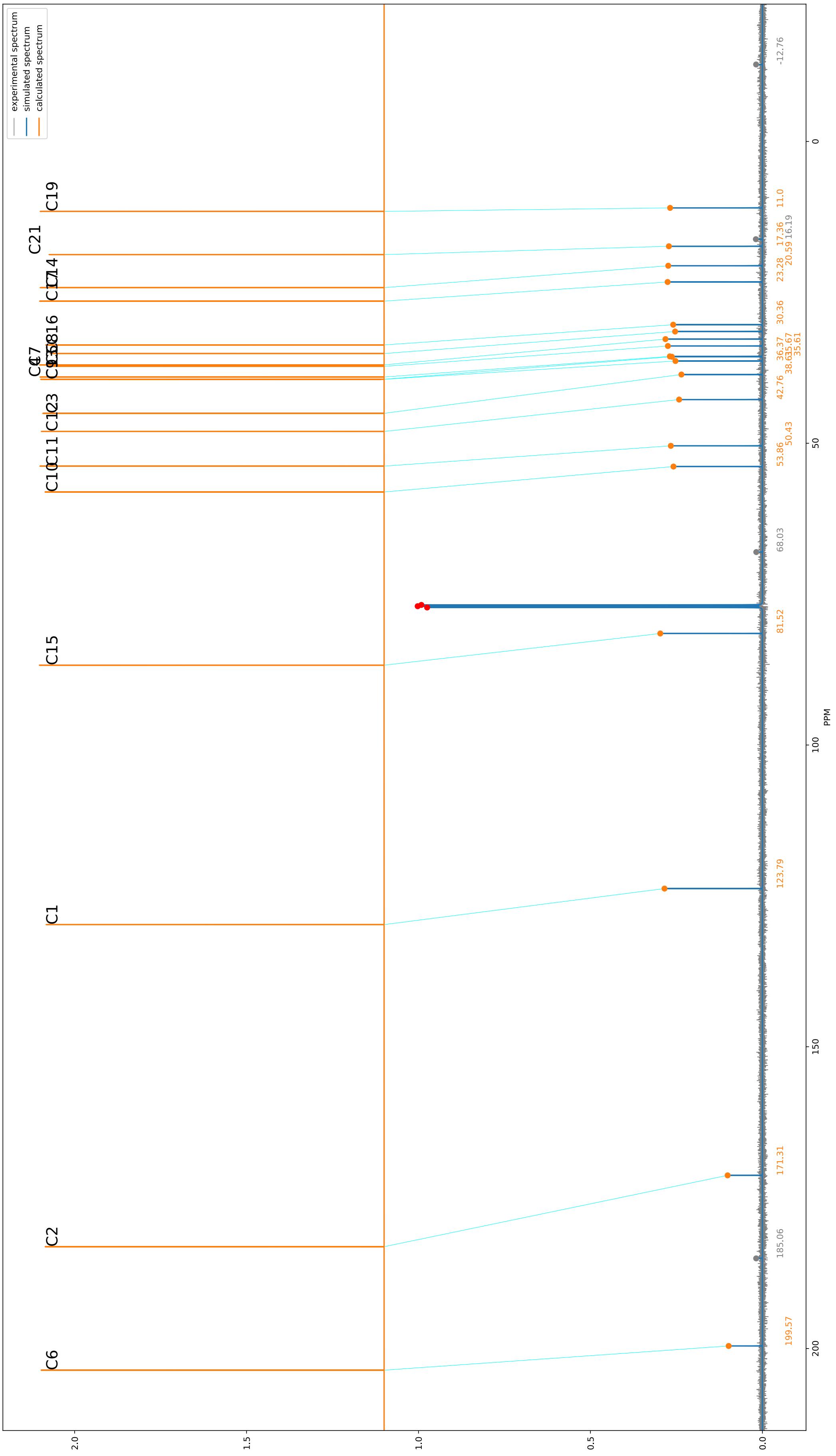

Figure 95: NP2 Carbon Spectra

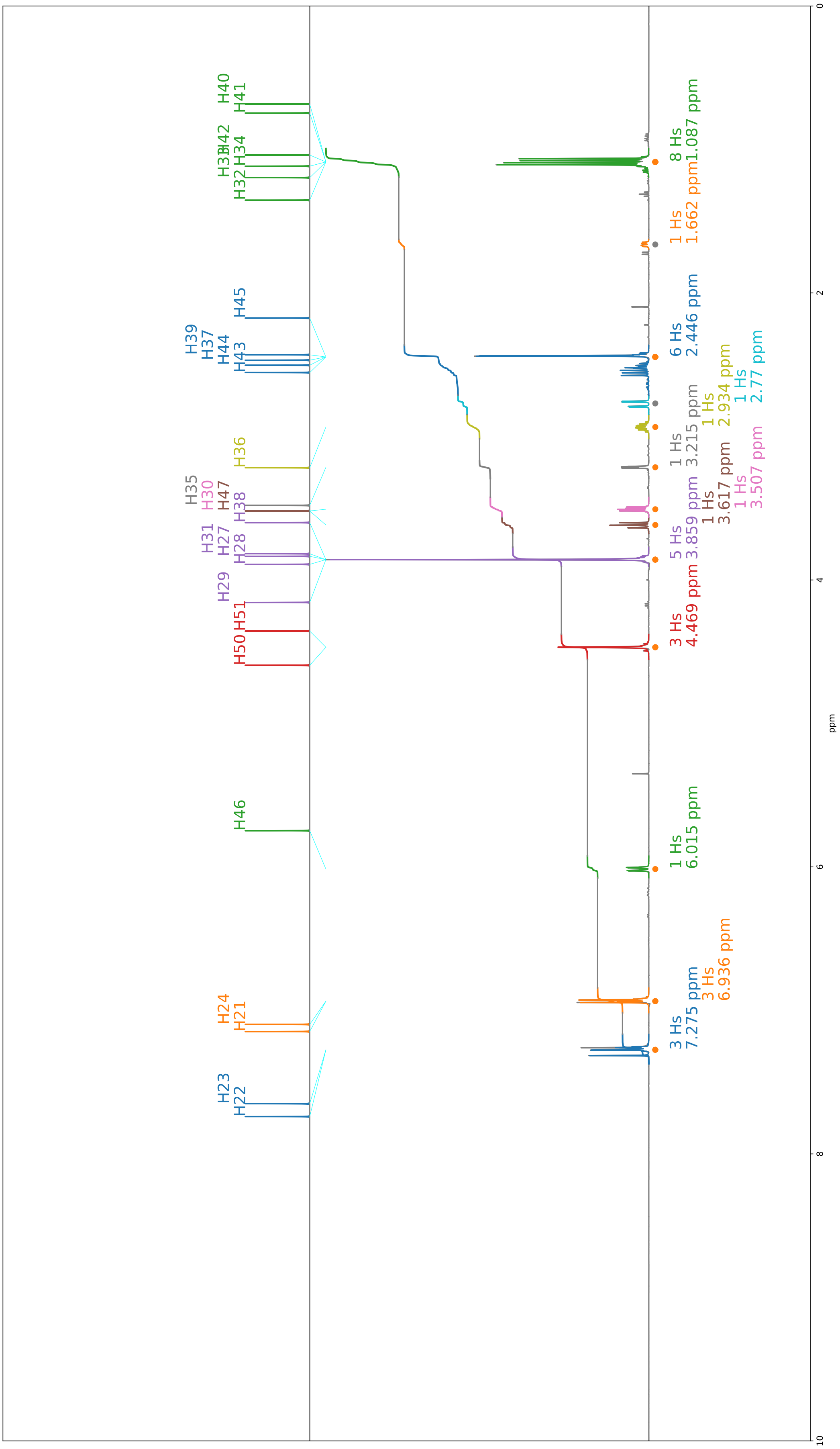

Figure 96: BYH2 Proton Spectra

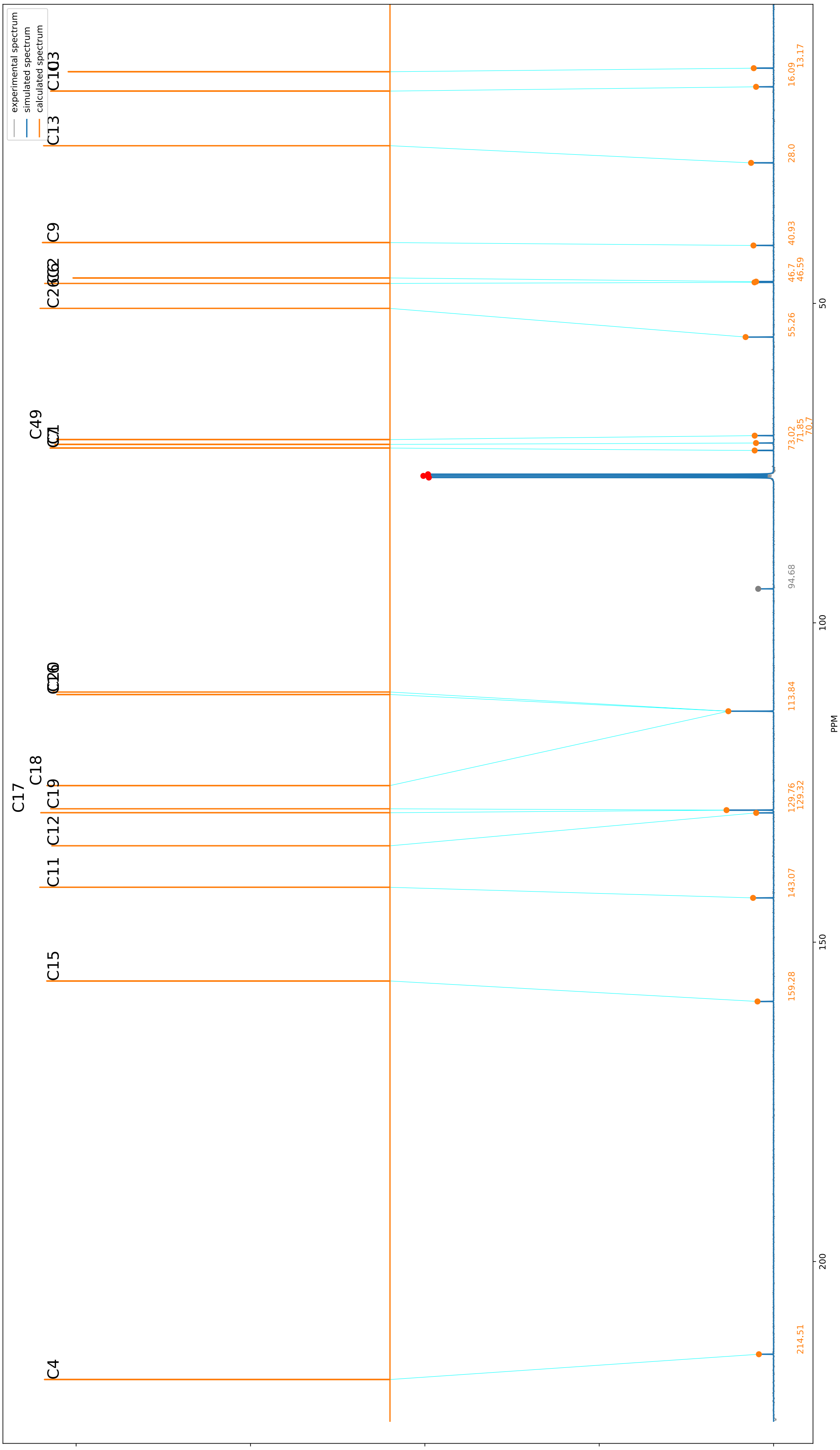

Figure 97: BYH2 Carbon Spectra

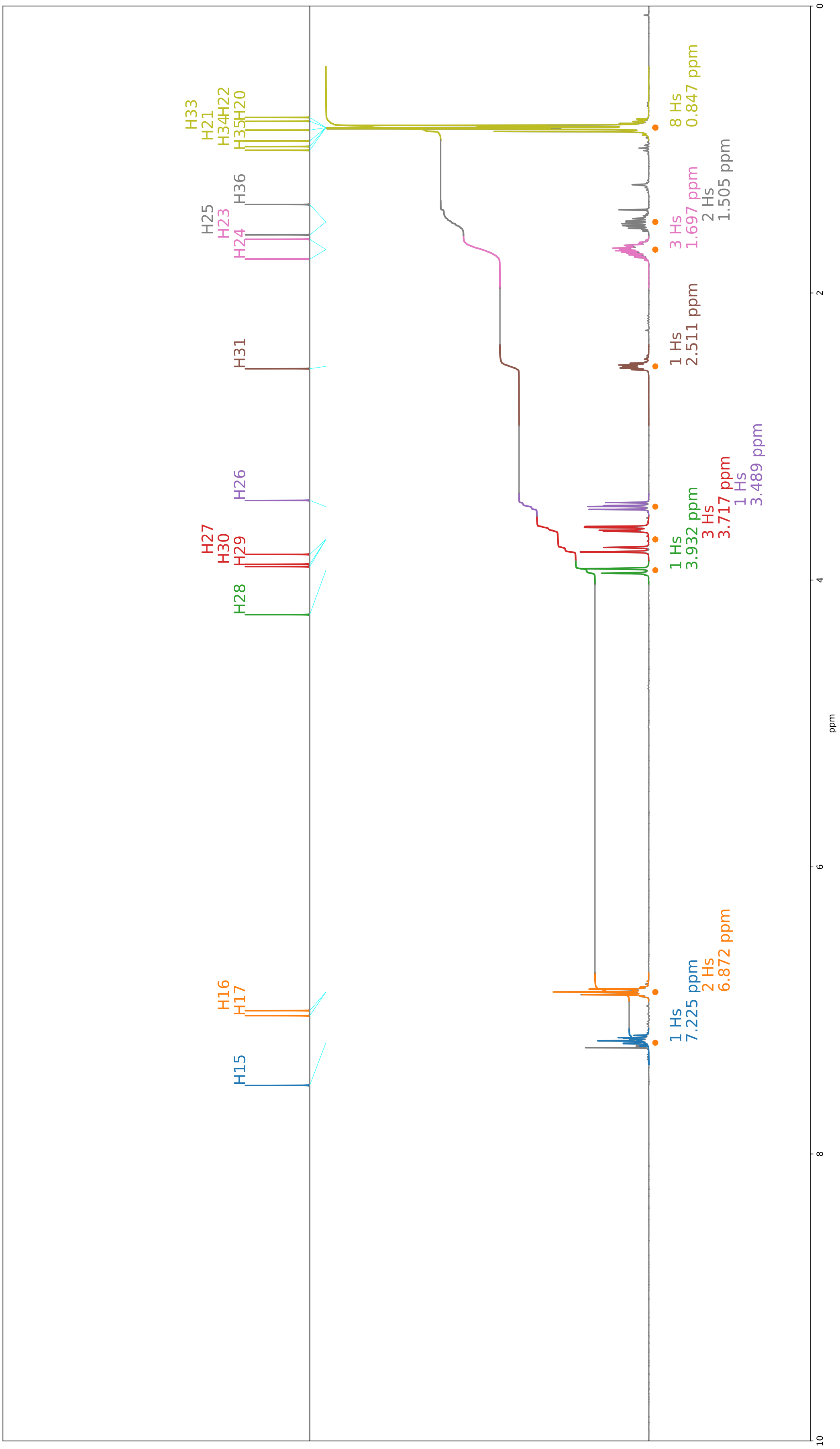

Figure 98: AT3 Proton Spectra

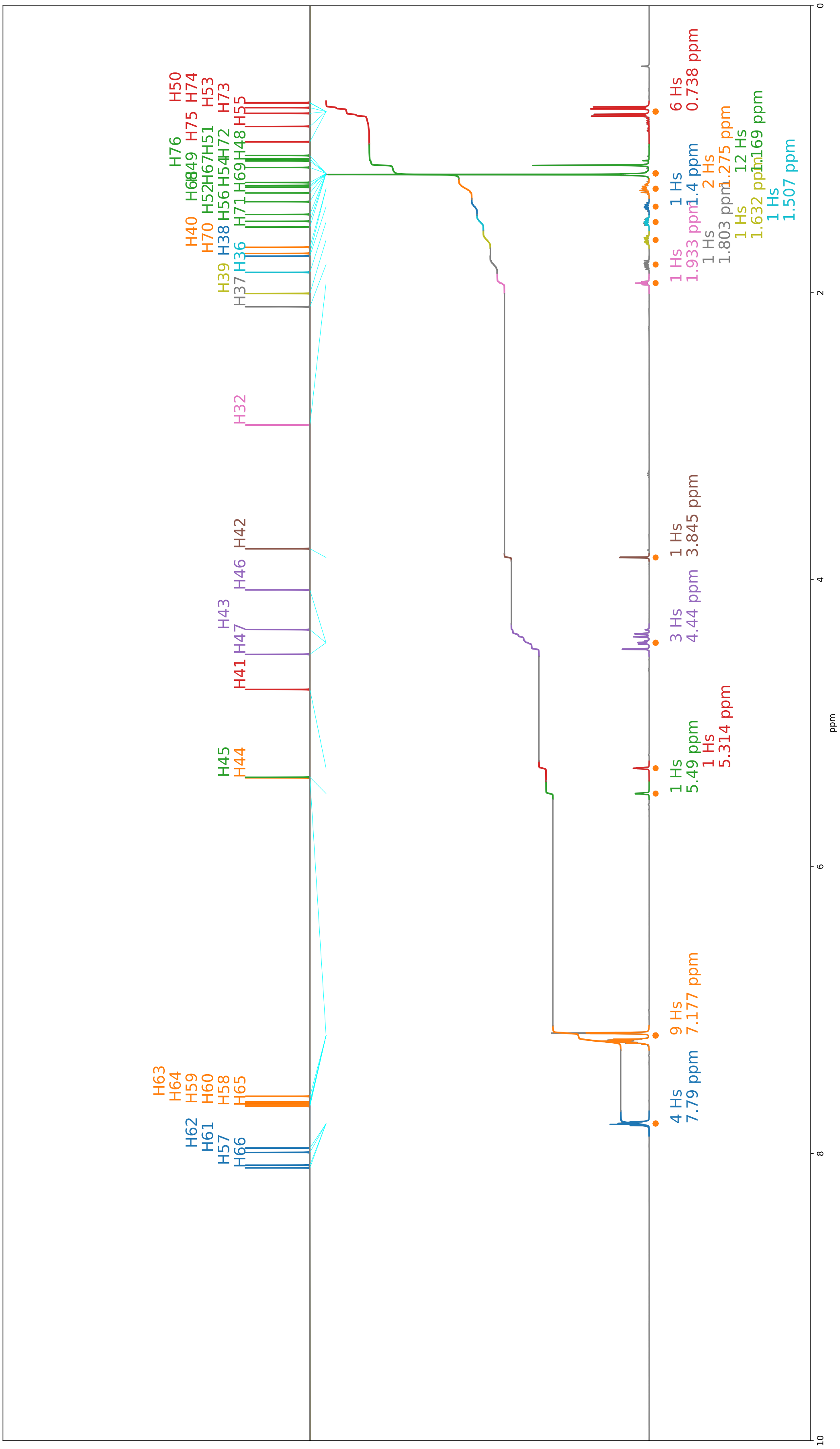

Figure 99: JB11 Proton Spectra

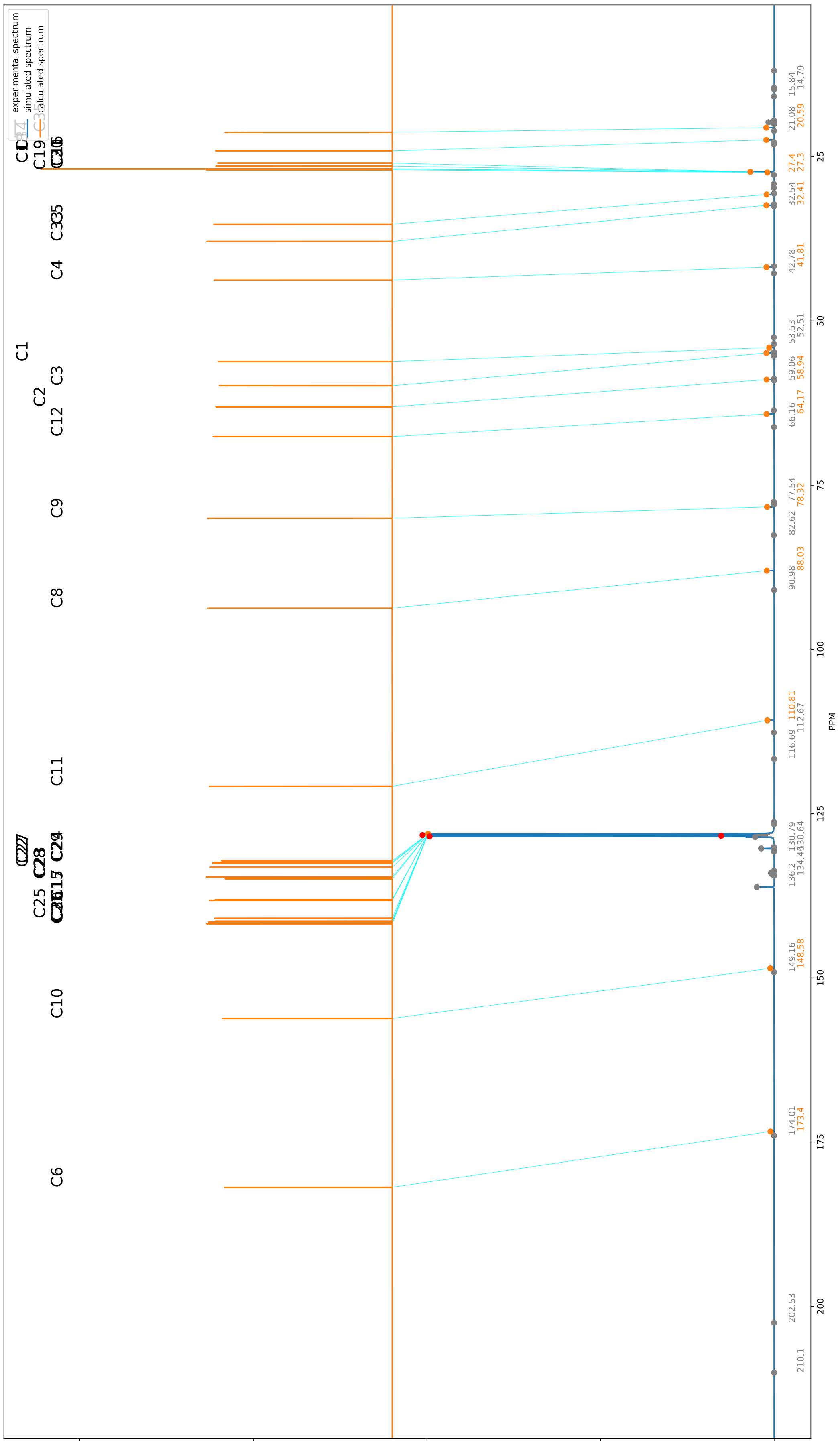

Figure 100: JB11 Carbon Spectra

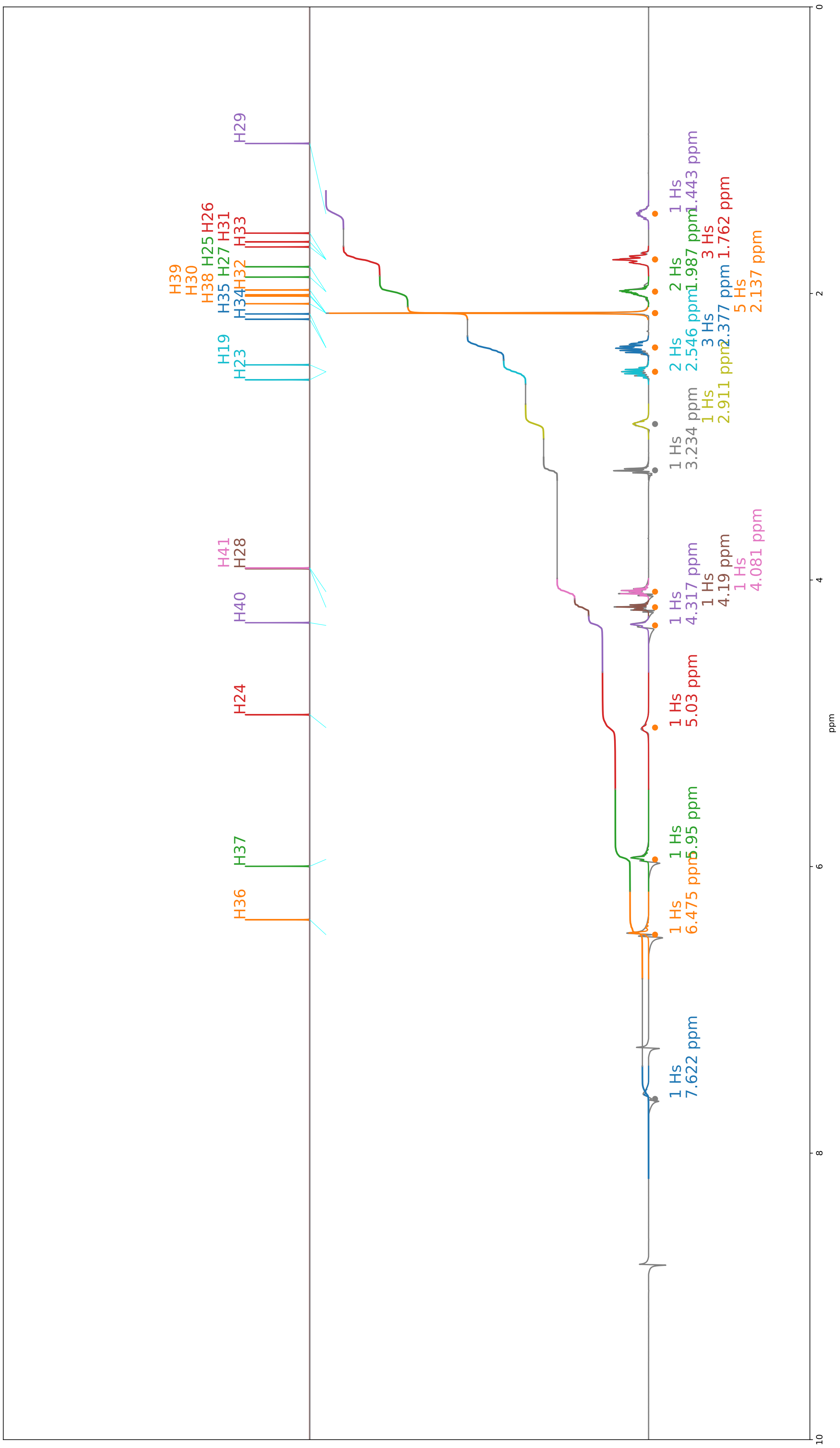

Figure 101: JB8 Proton Spectra

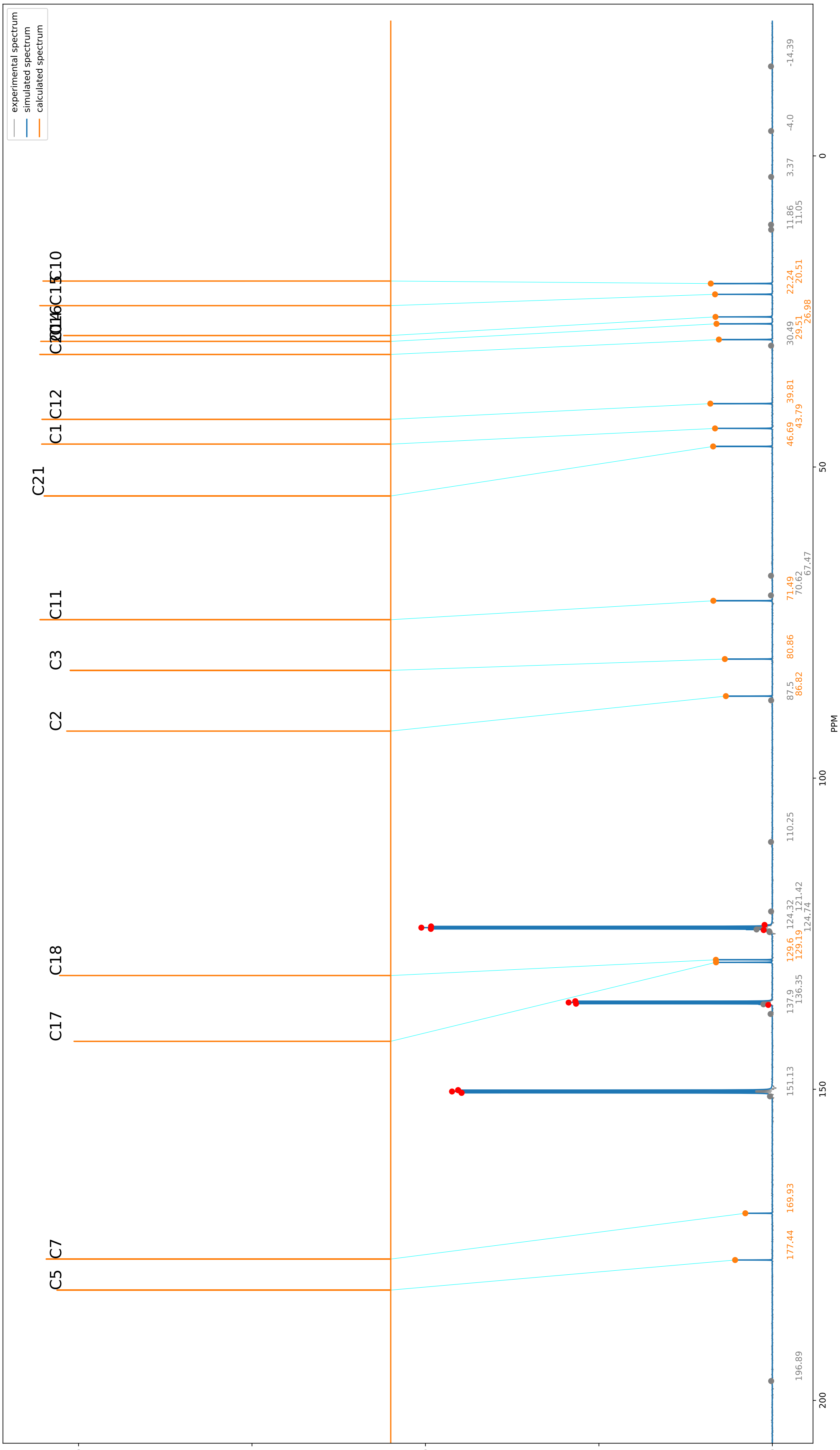

Figure 102: JB8 Carbon Spectra

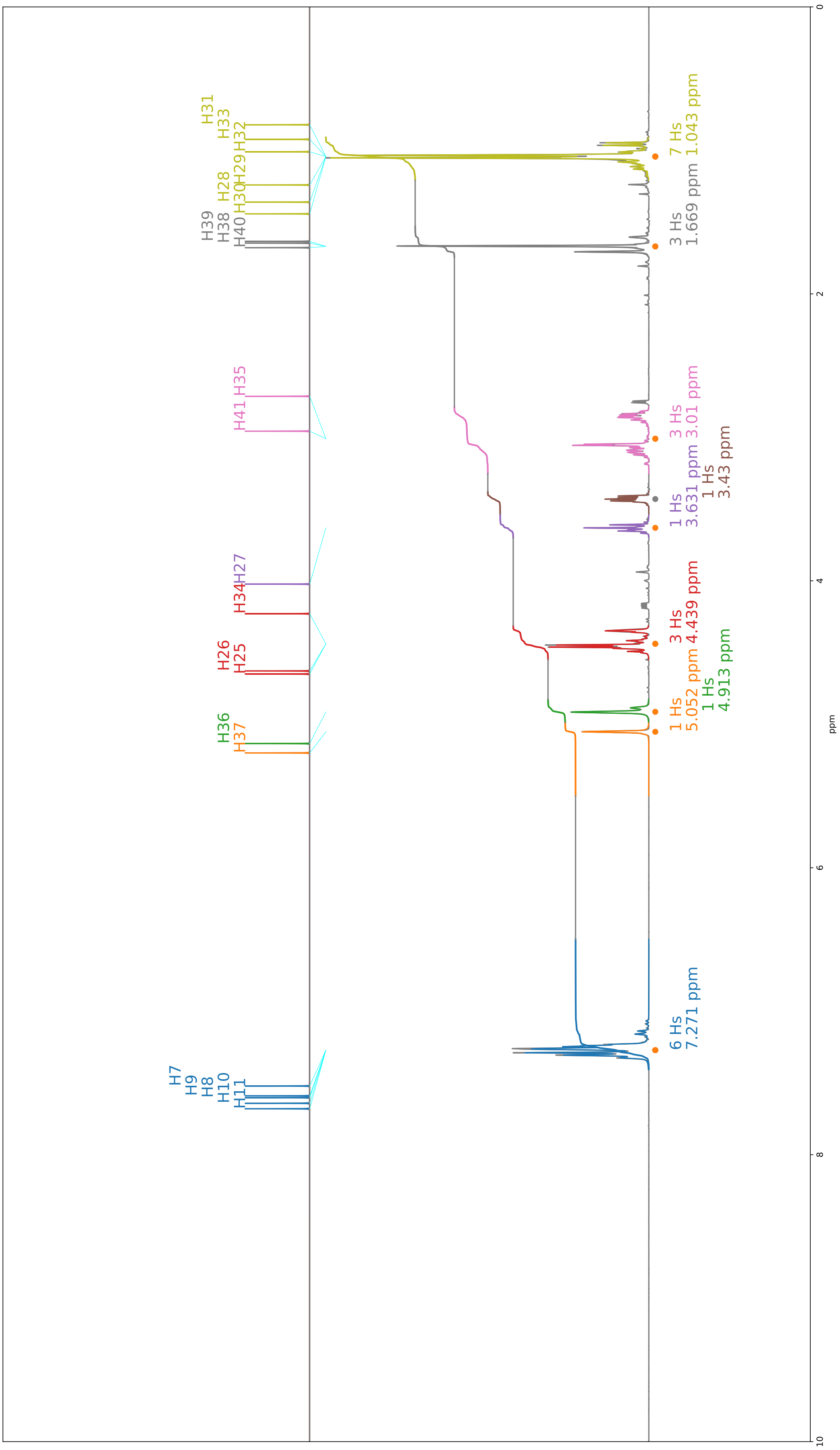

Figure 103: IP5 Proton Spectra

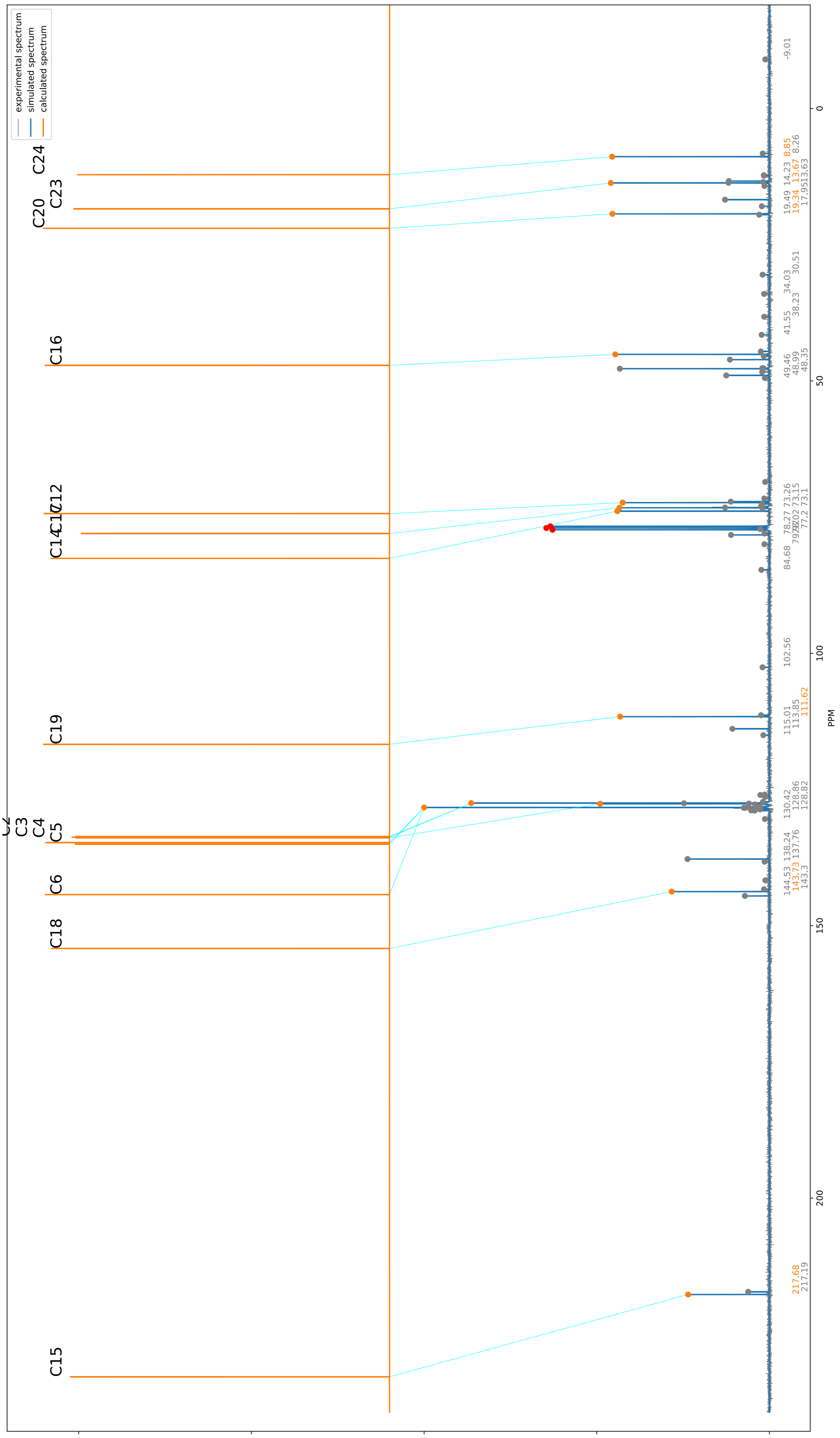

Figure 104: IP5 Carbon Spectra

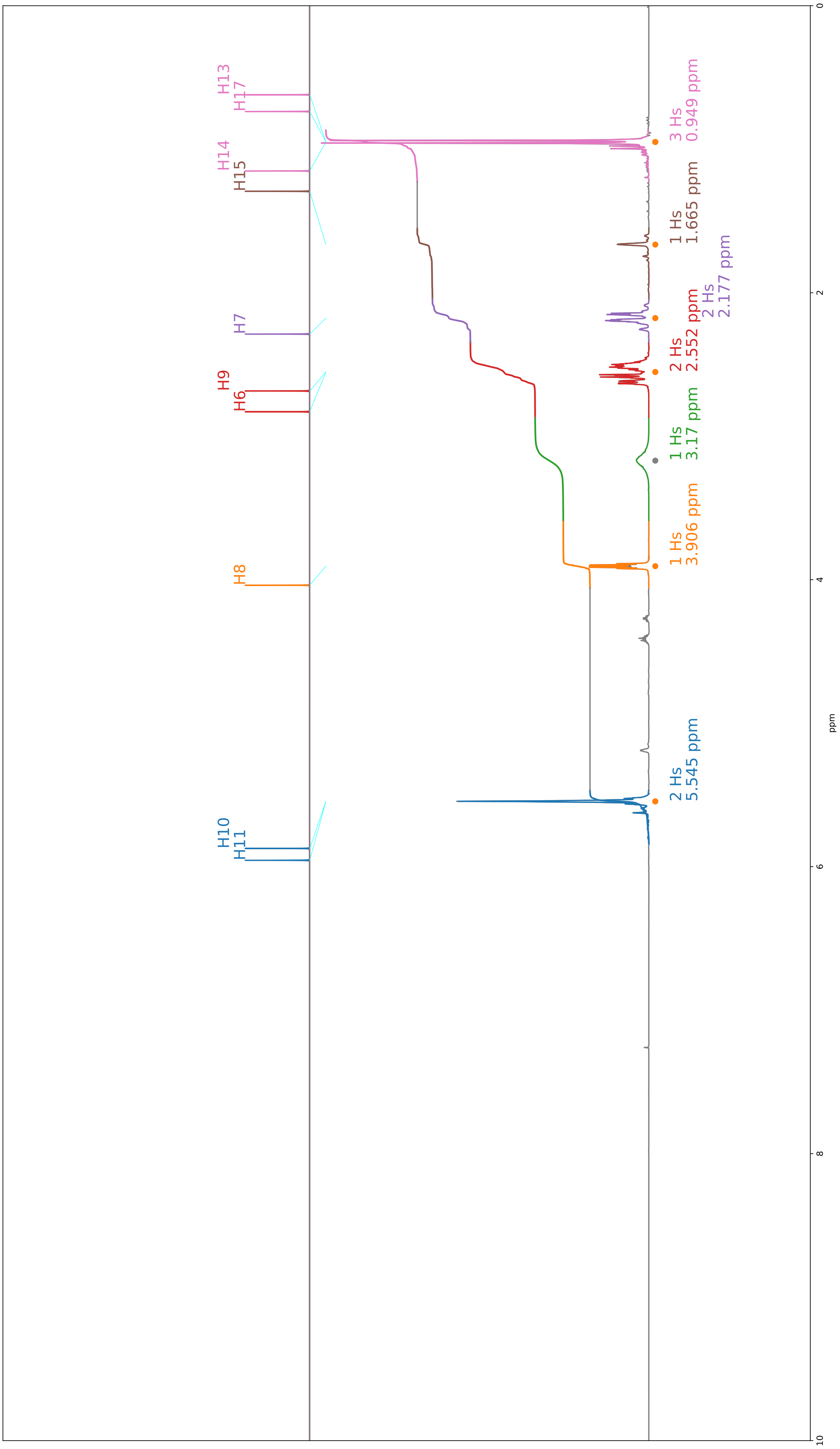

Figure 105: IP4 Proton Spectra

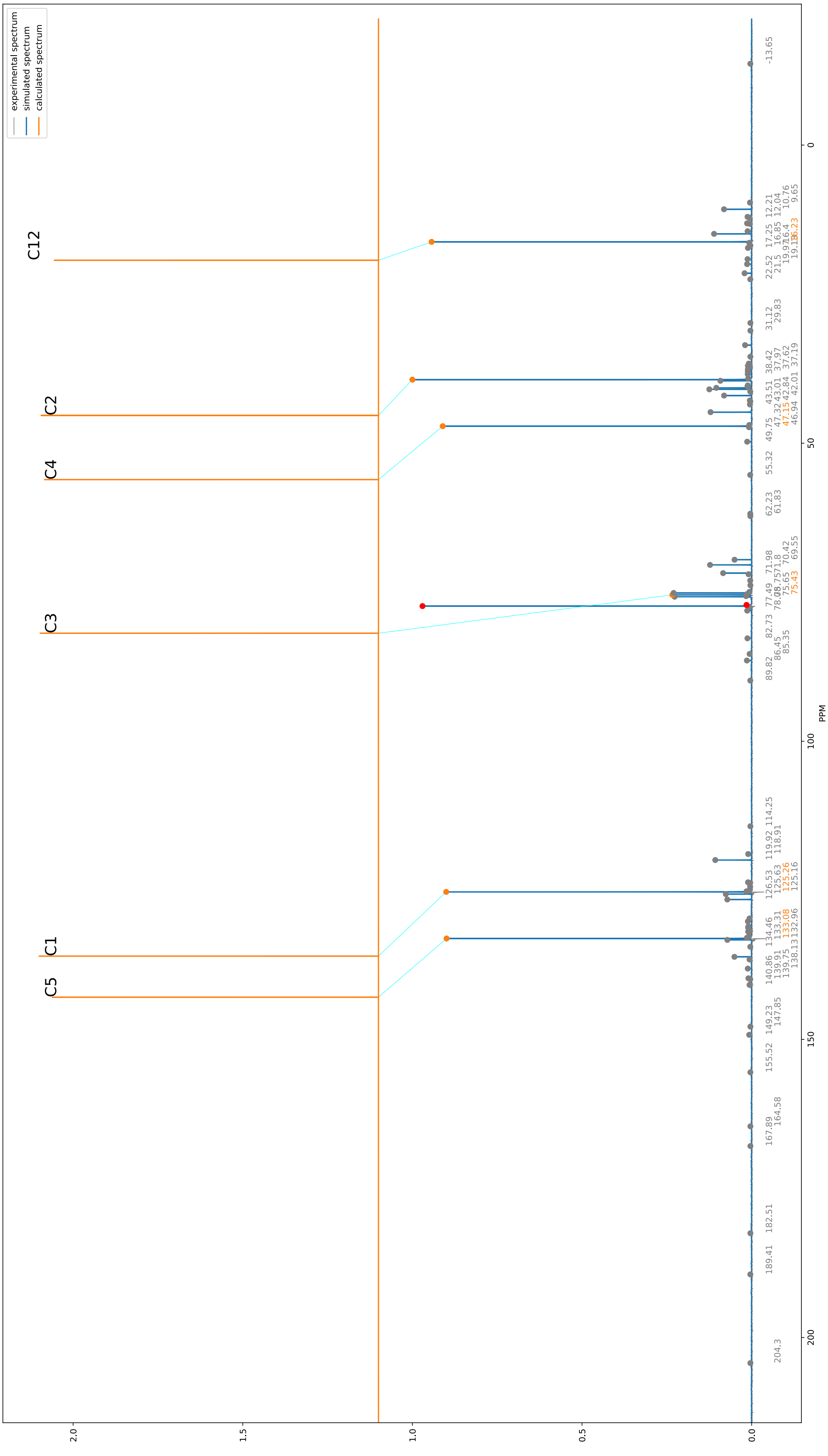

Figure 106: IP4 Carbon Spectra

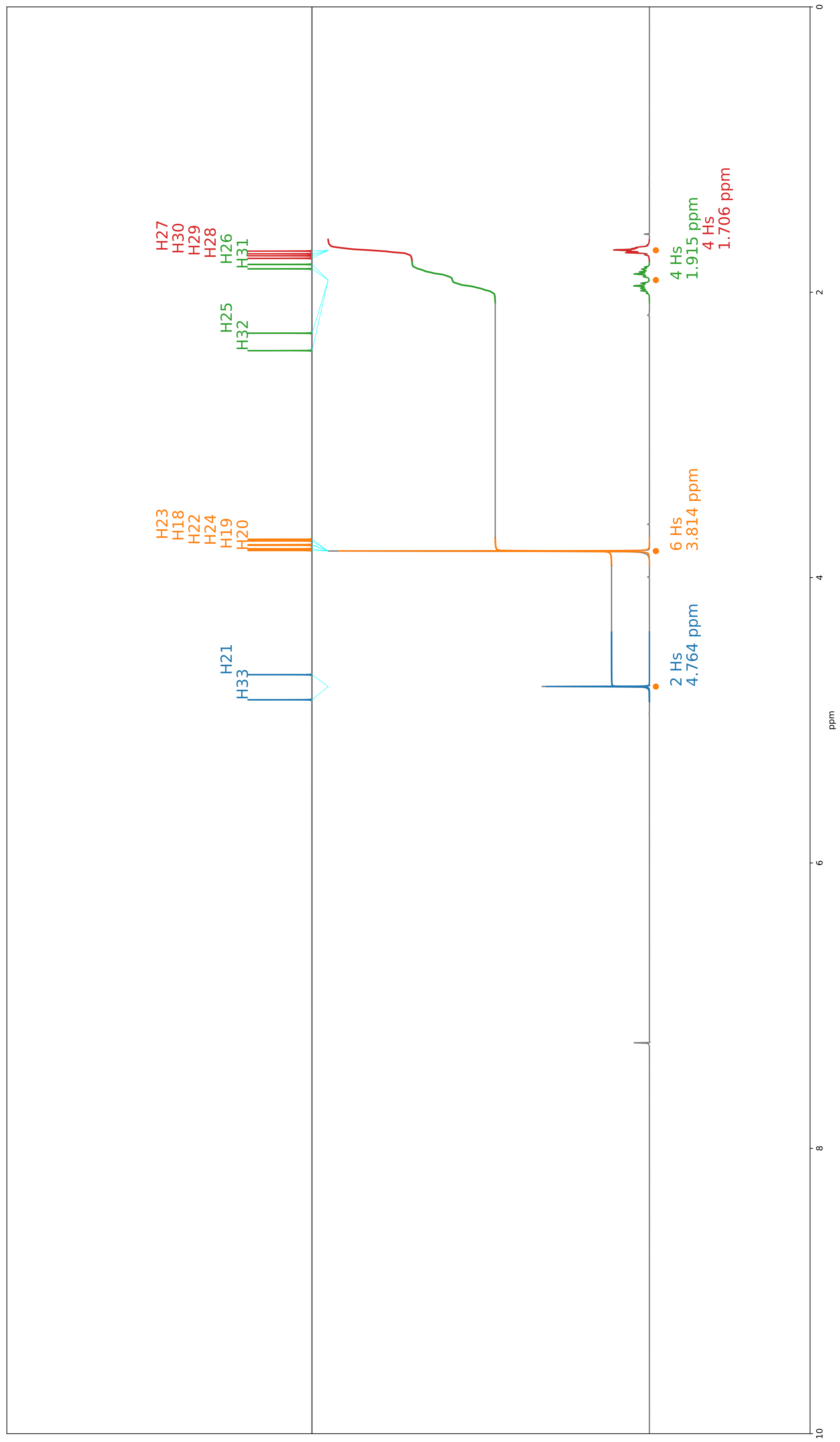

Figure 107: IP3 Proton Spectra

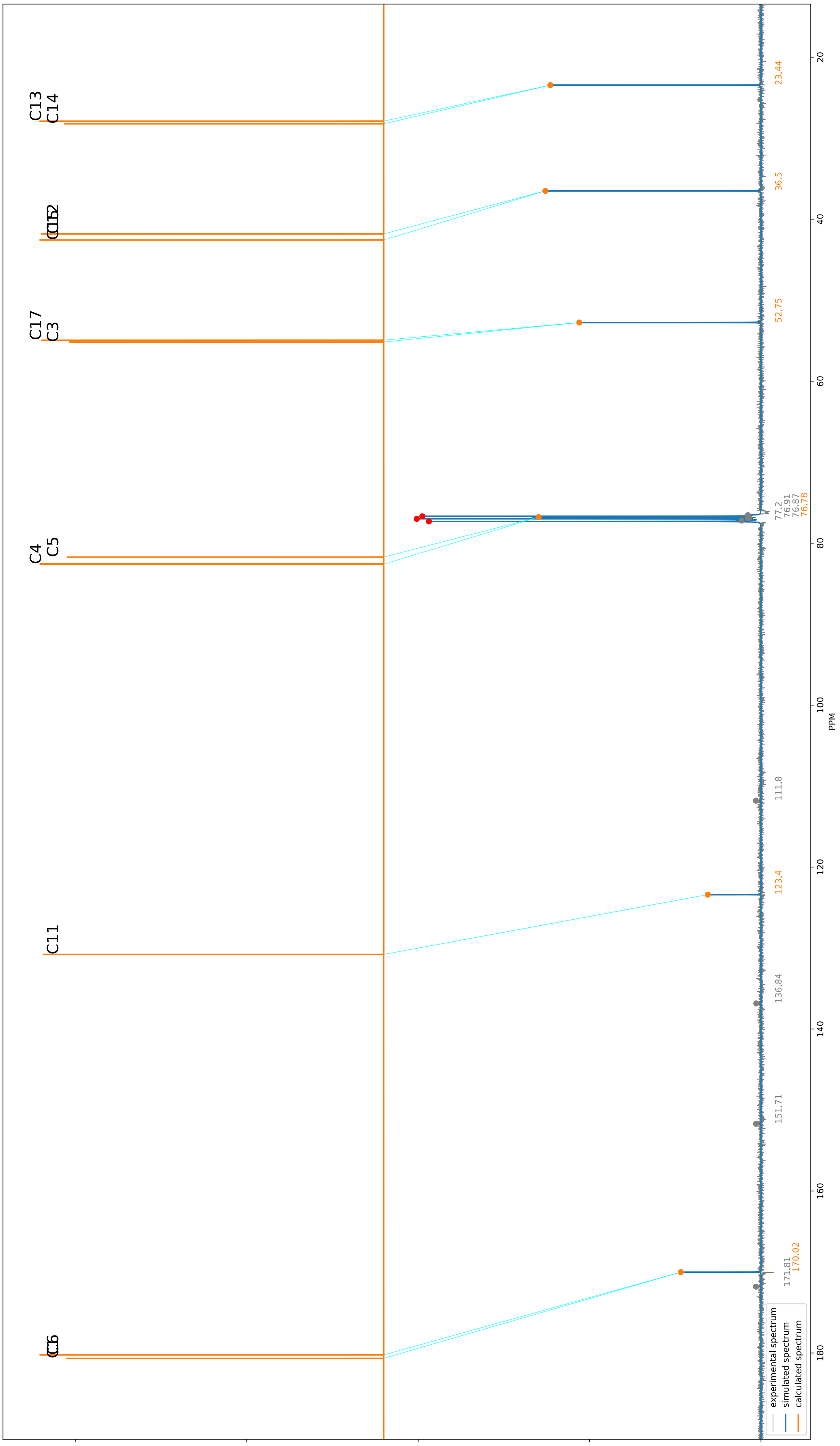

Figure 108: IP3 Carbon Spectra

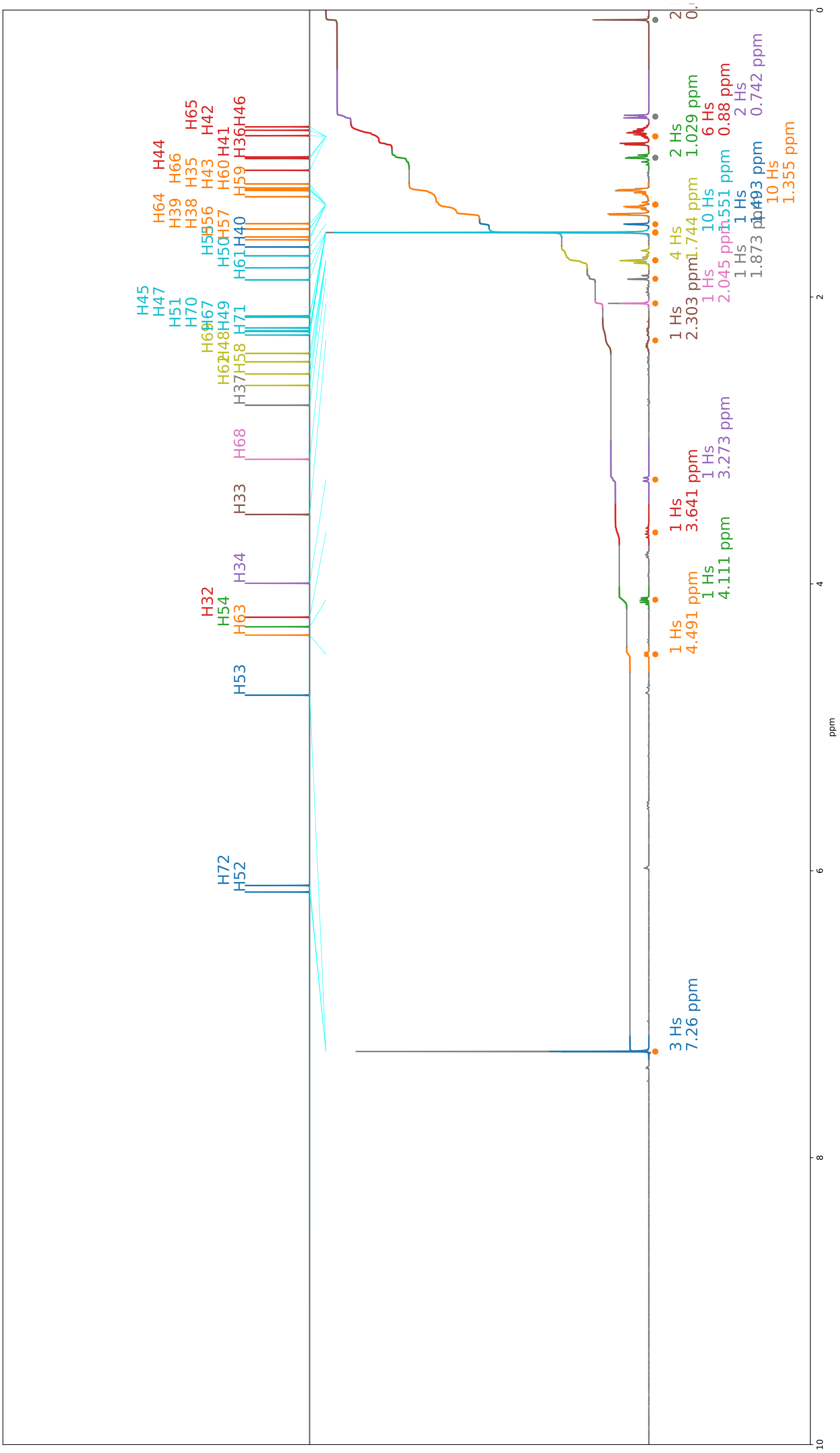

Figure 109: NL2A Proton Spectra

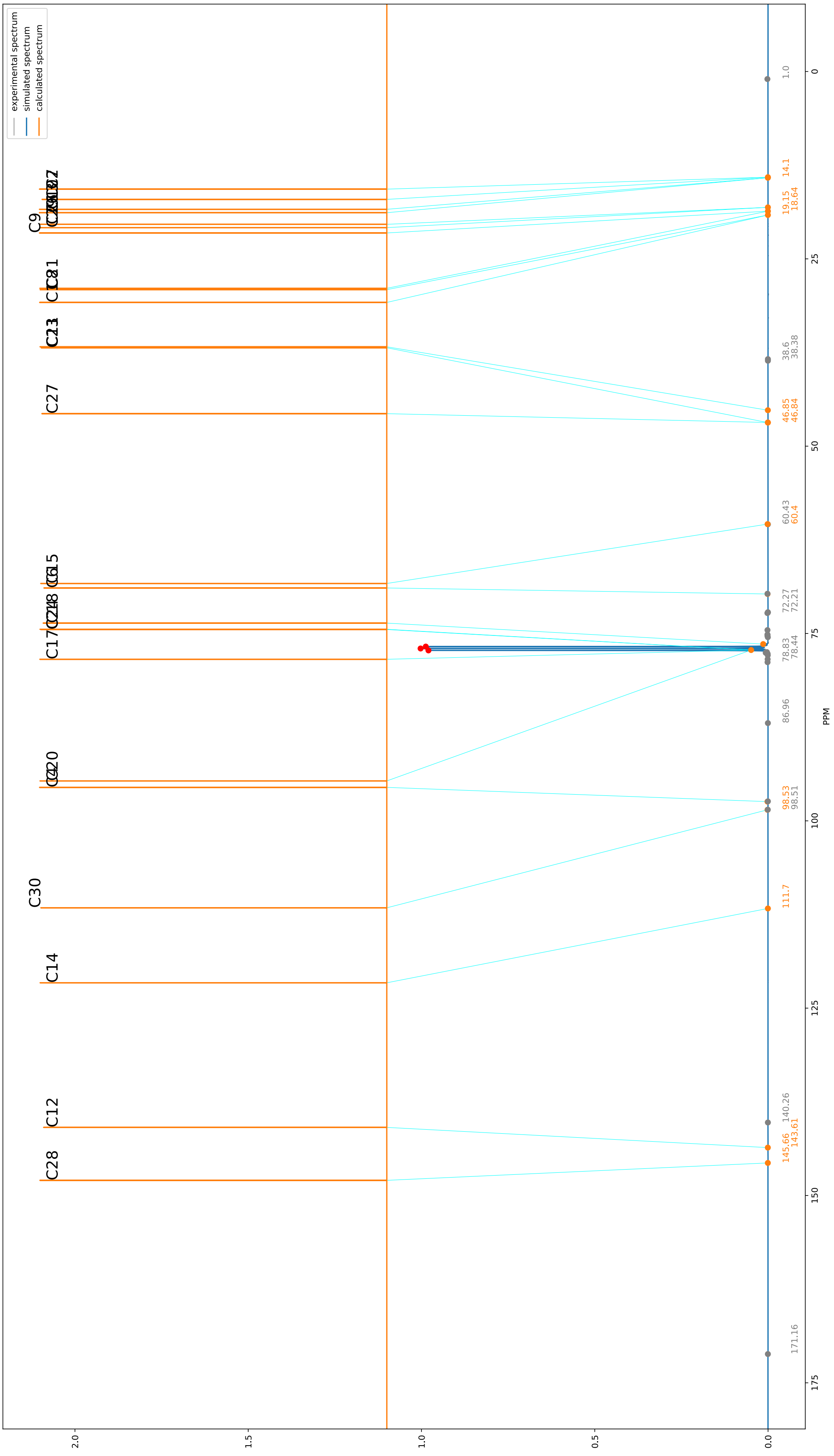

Figure 110: NL2A Carbon Spectra

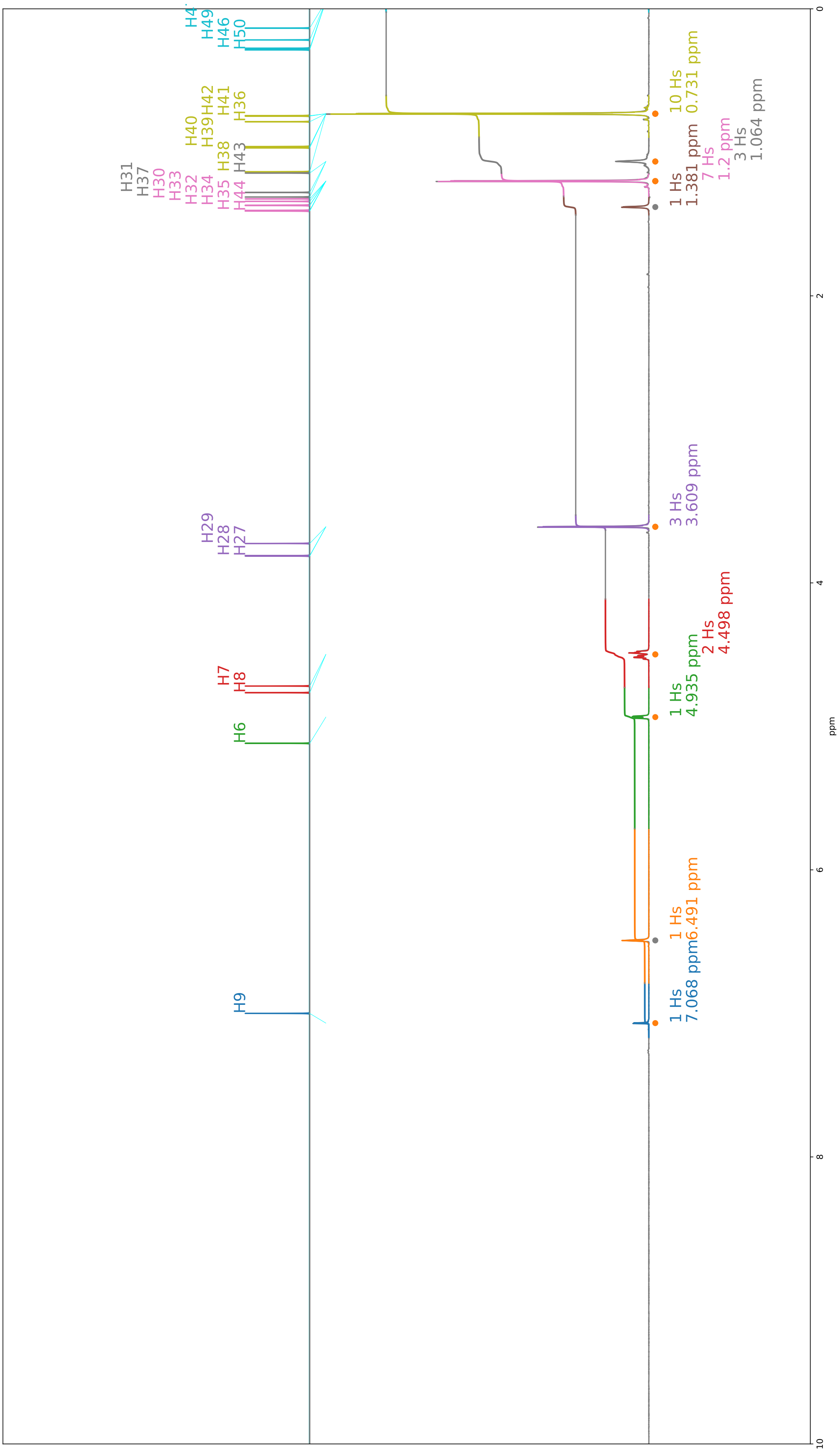

Figure 111: KE1 Proton Spectra

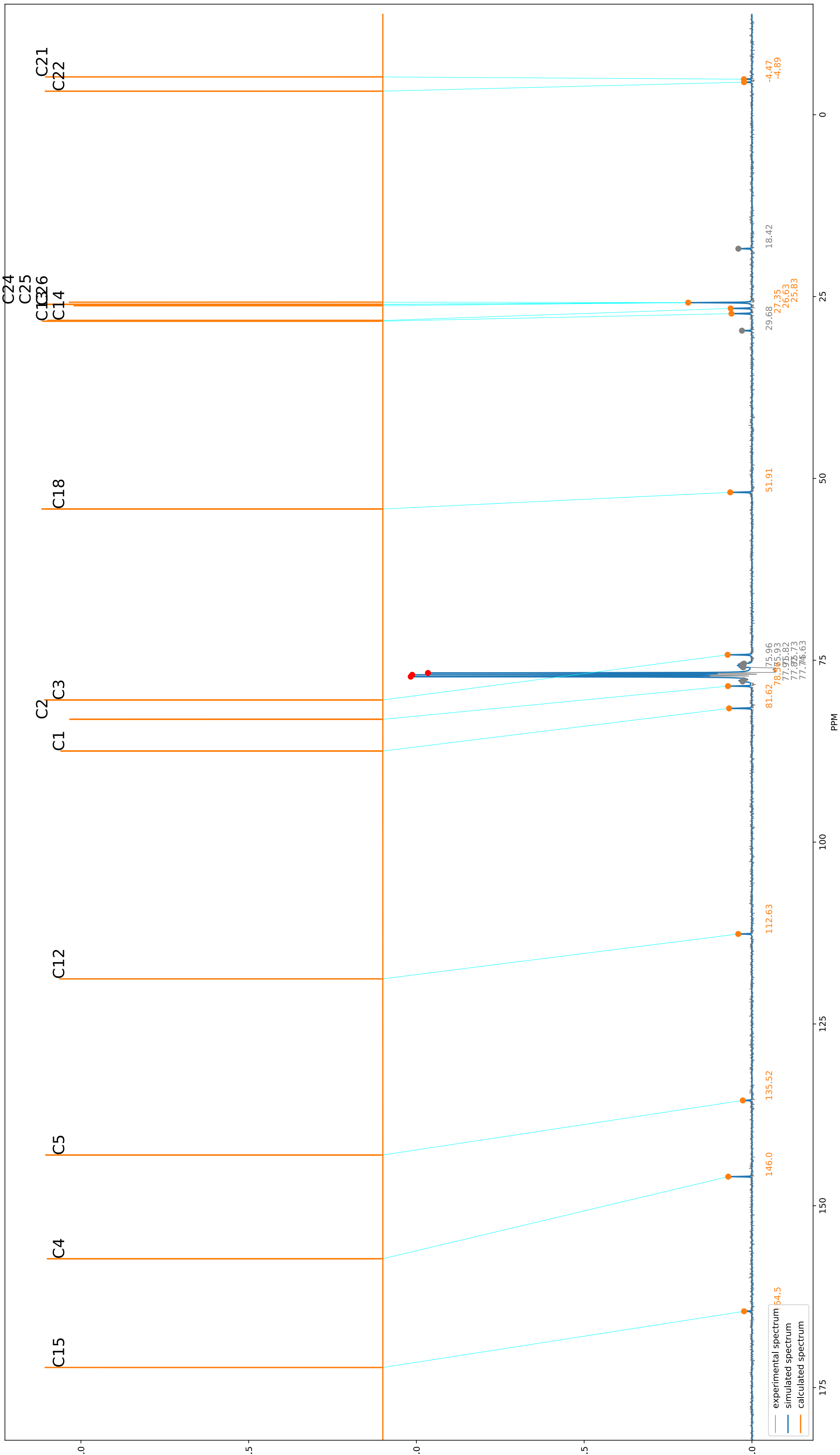

Figure 112: KE1 Carbon Spectra

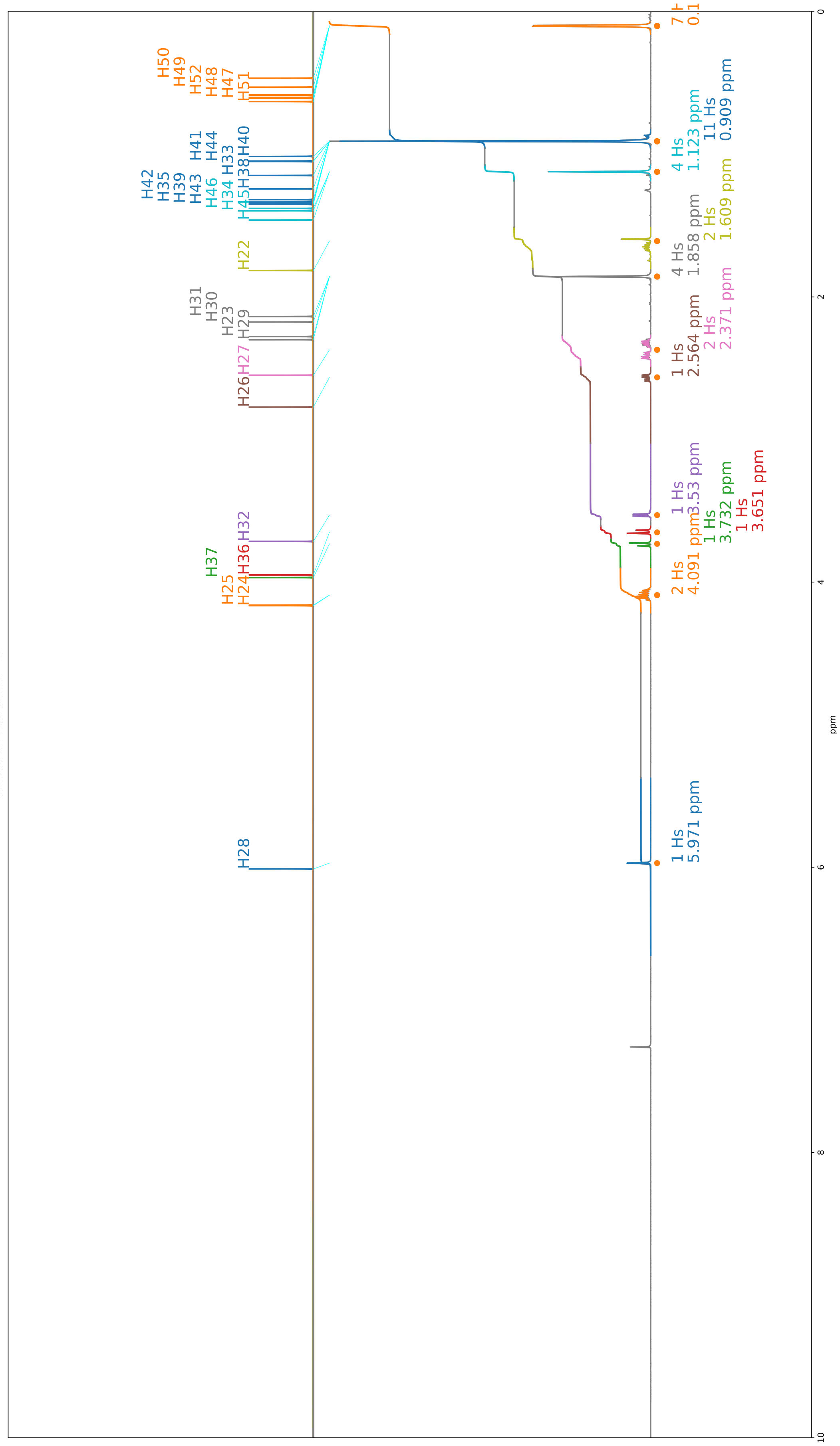

Figure 113: NL1B Proton Spectra

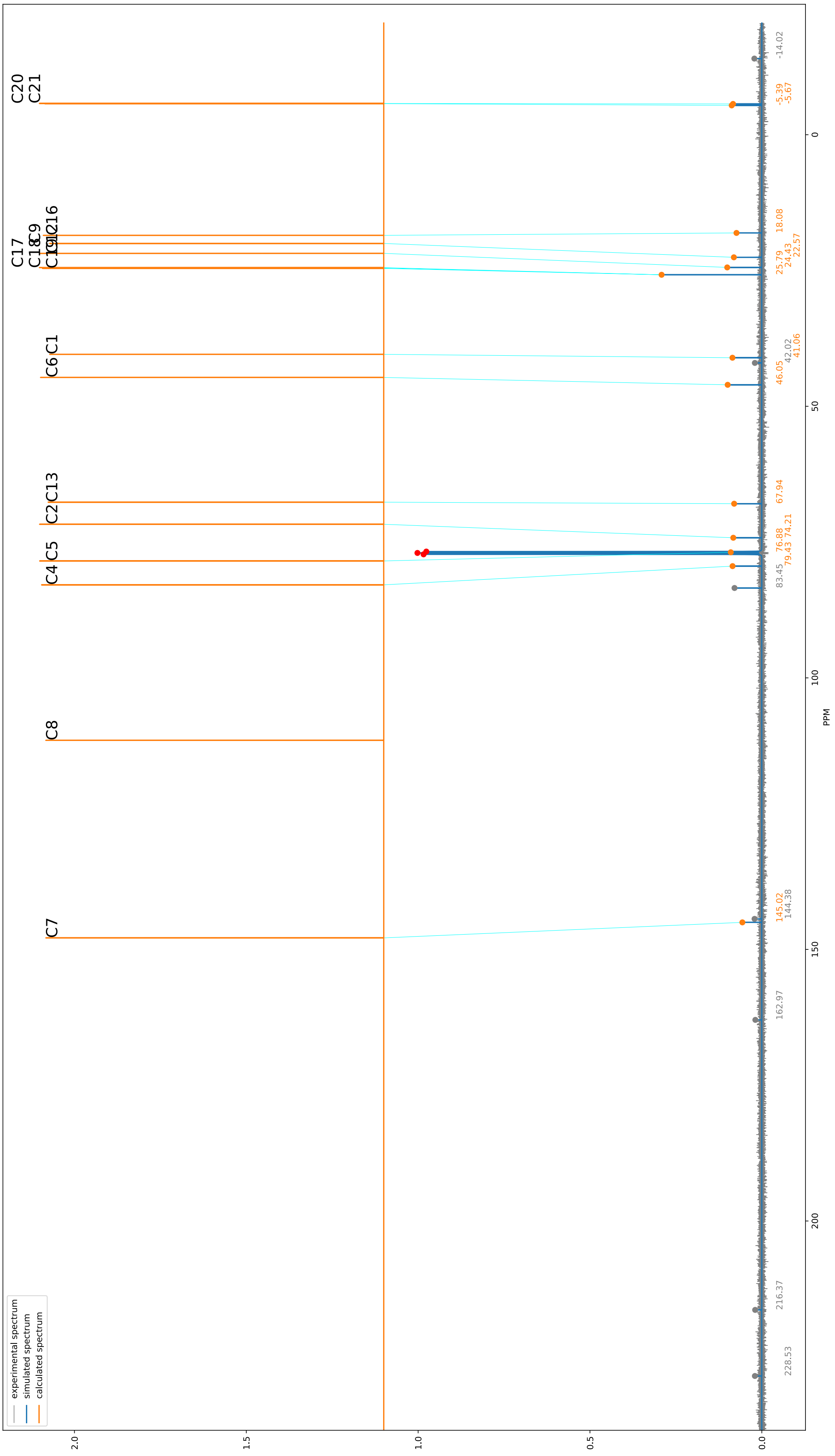

Figure 114: NL1B Carbon Spectra

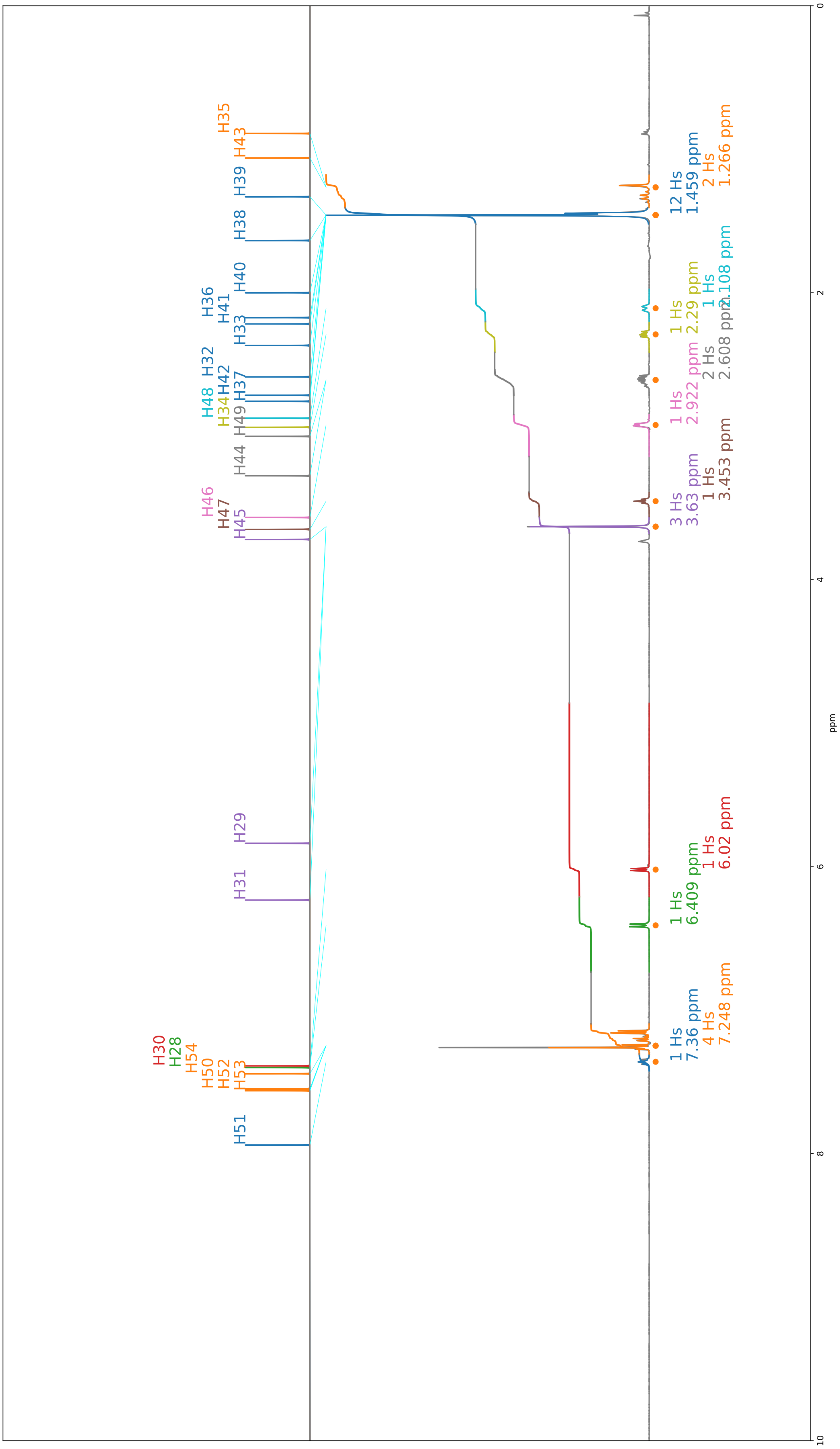

Figure 115: OD1 Proton Spectra

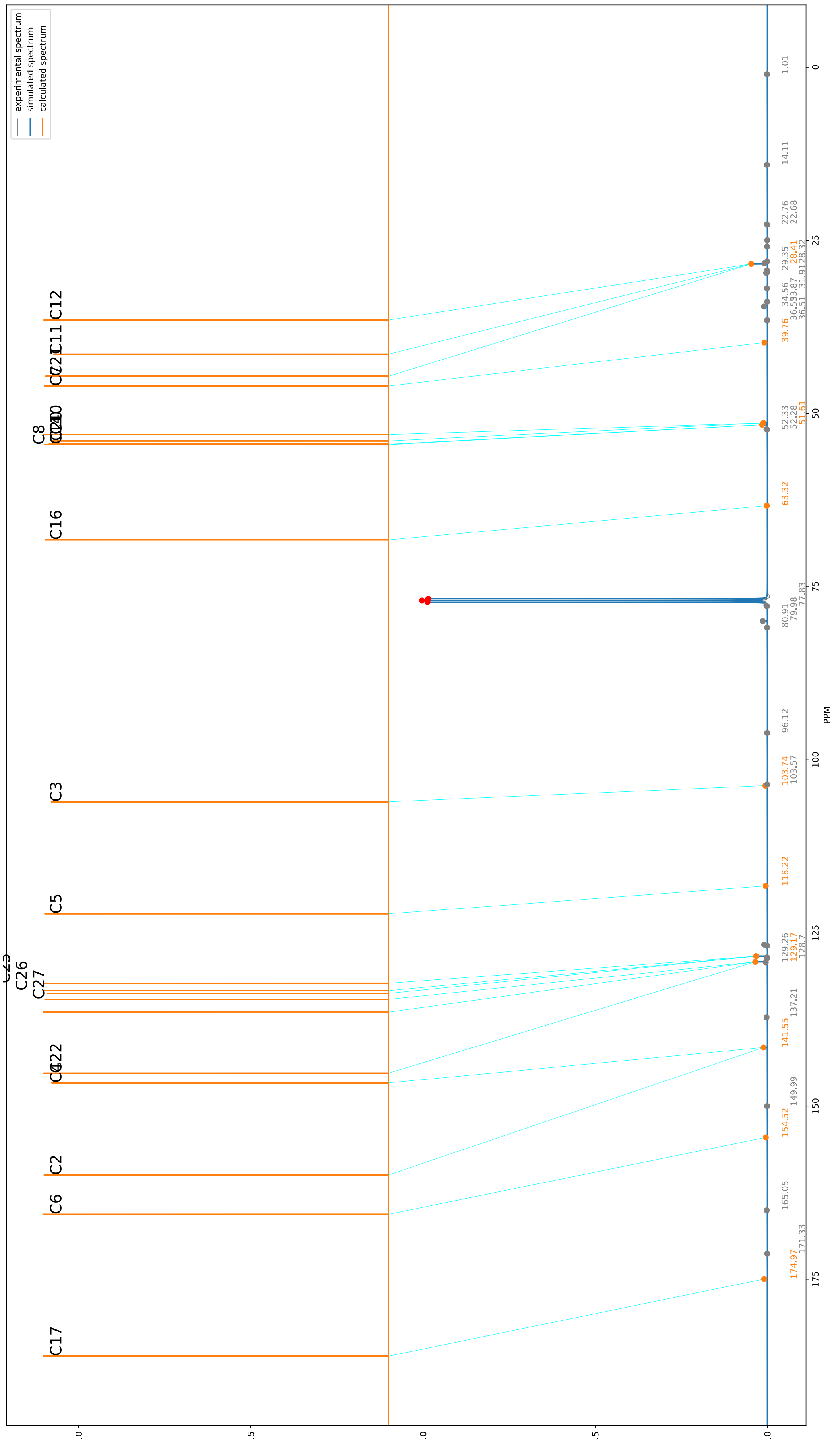

Figure 116: OD1 Carbon Spectra

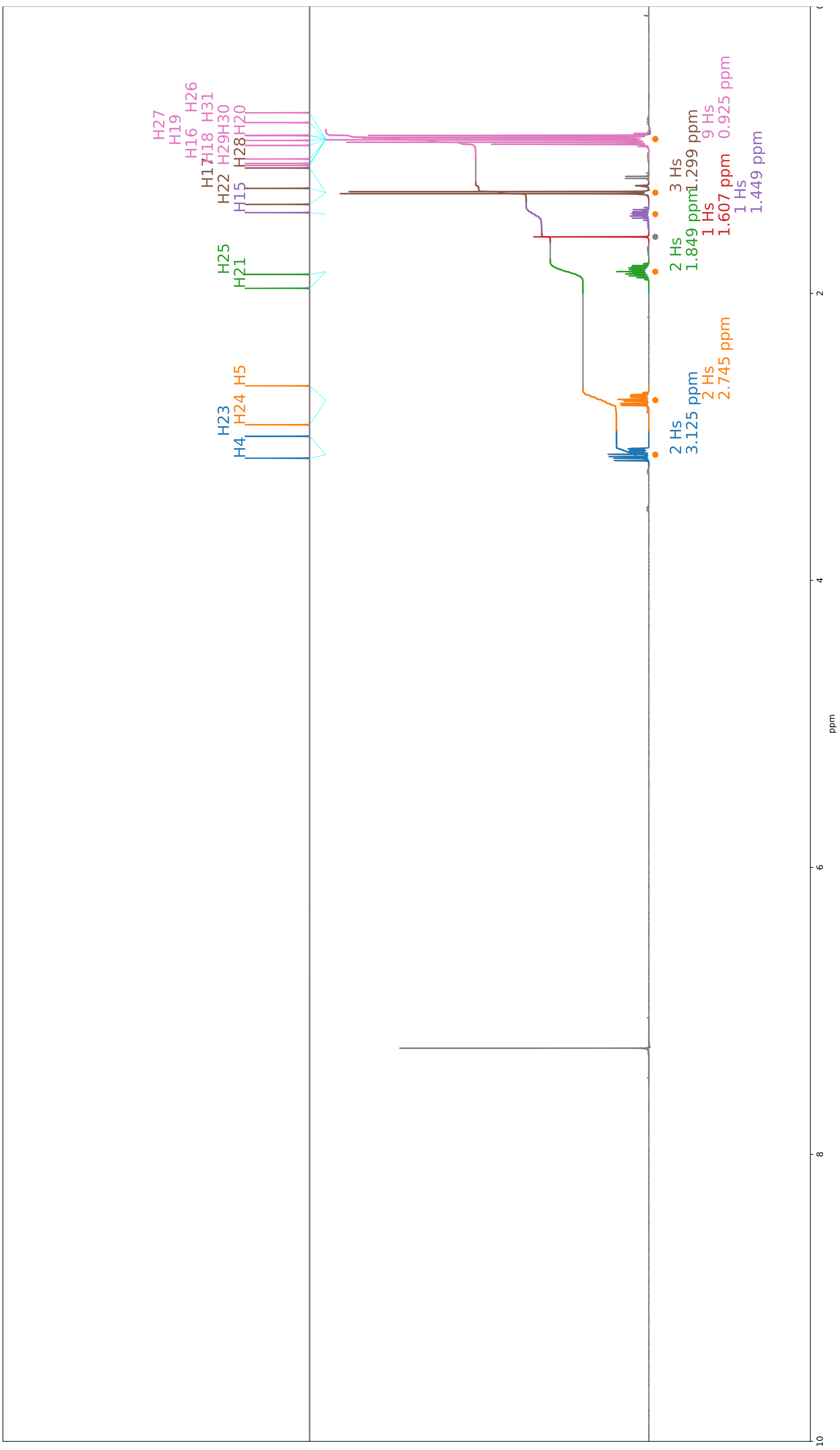

Figure 117: AT1 Proton Spectra

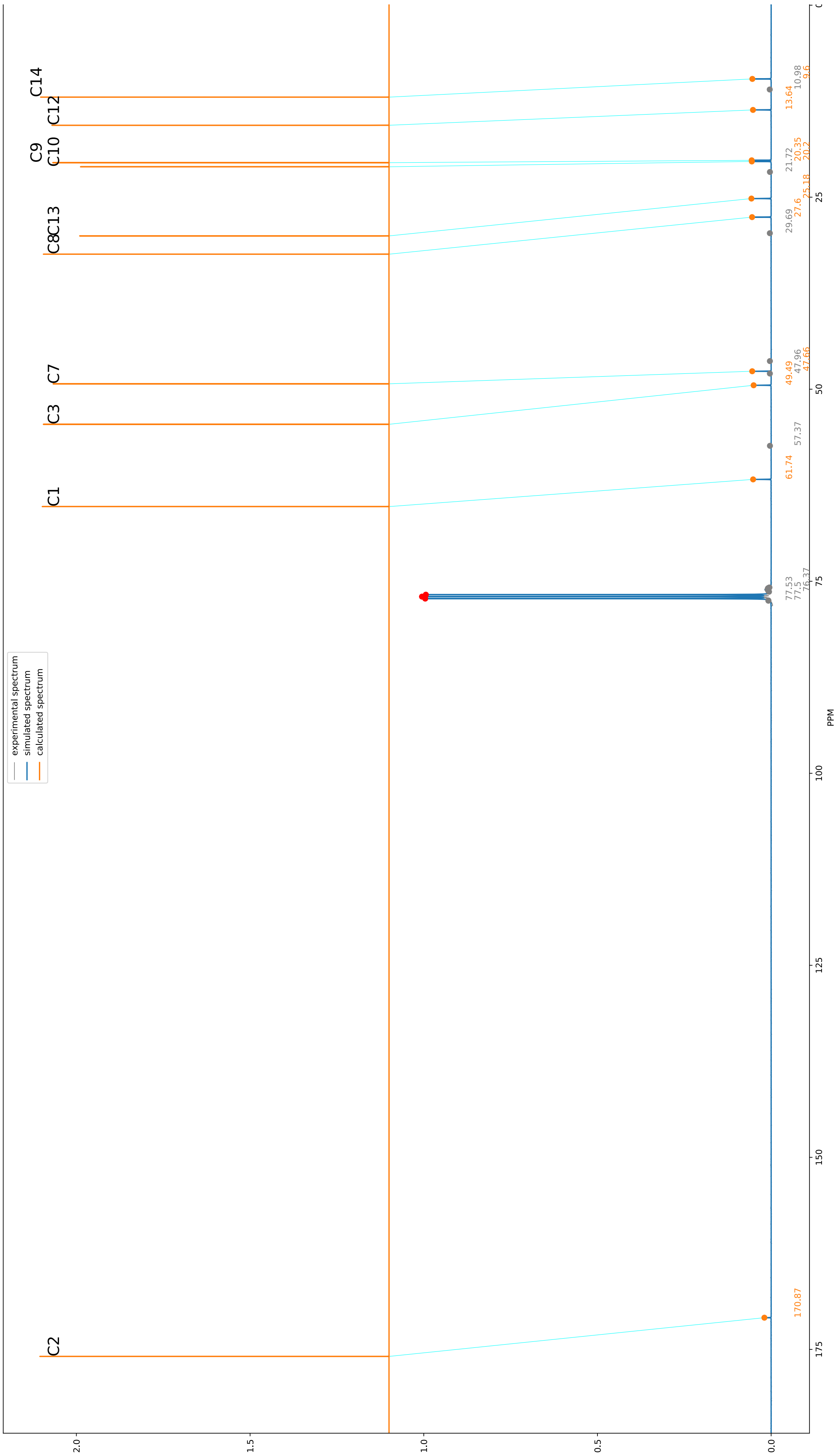

Figure 118: AT1 Carbon Spectra

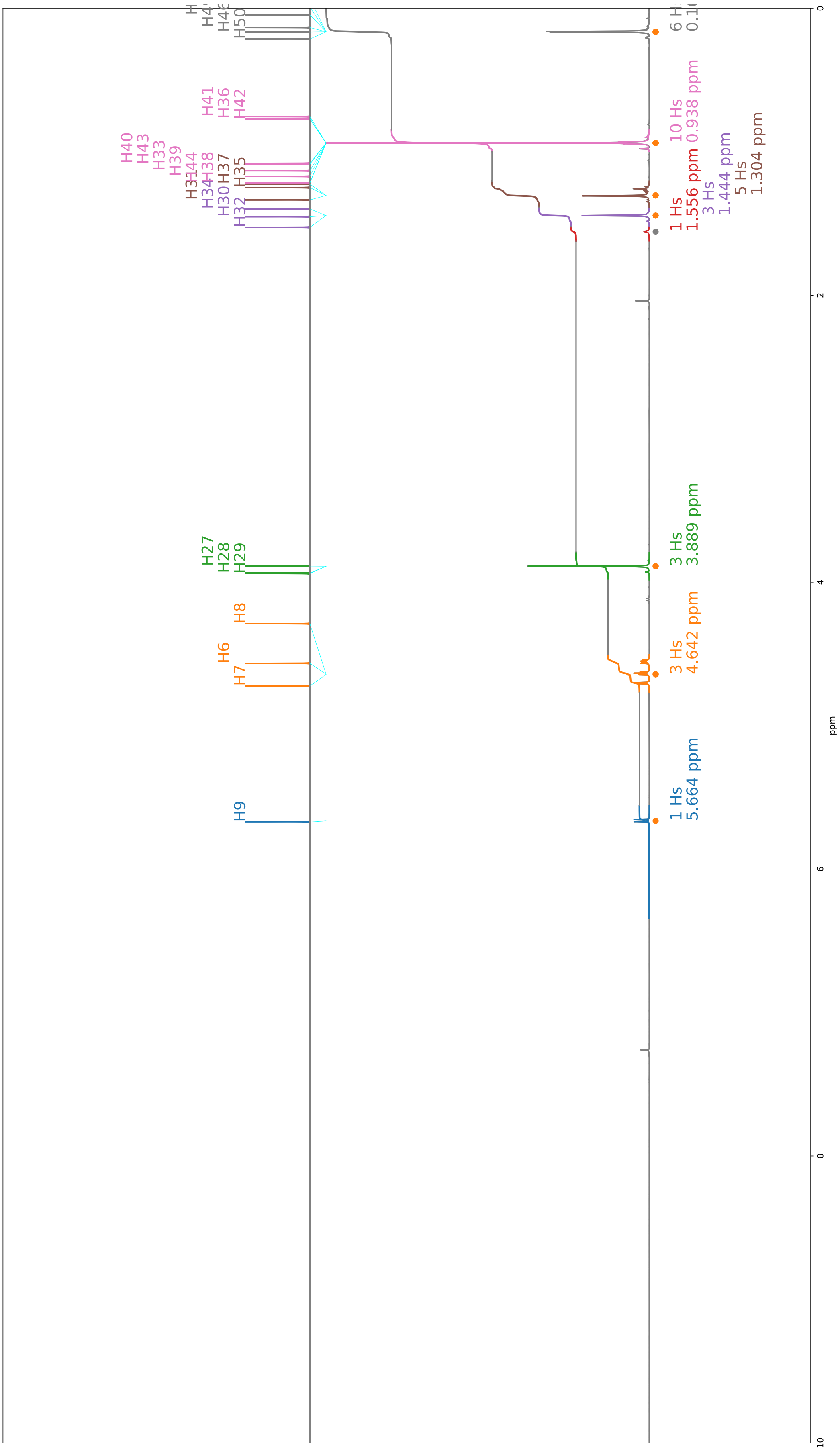

Figure 119: KE3 Proton Spectra

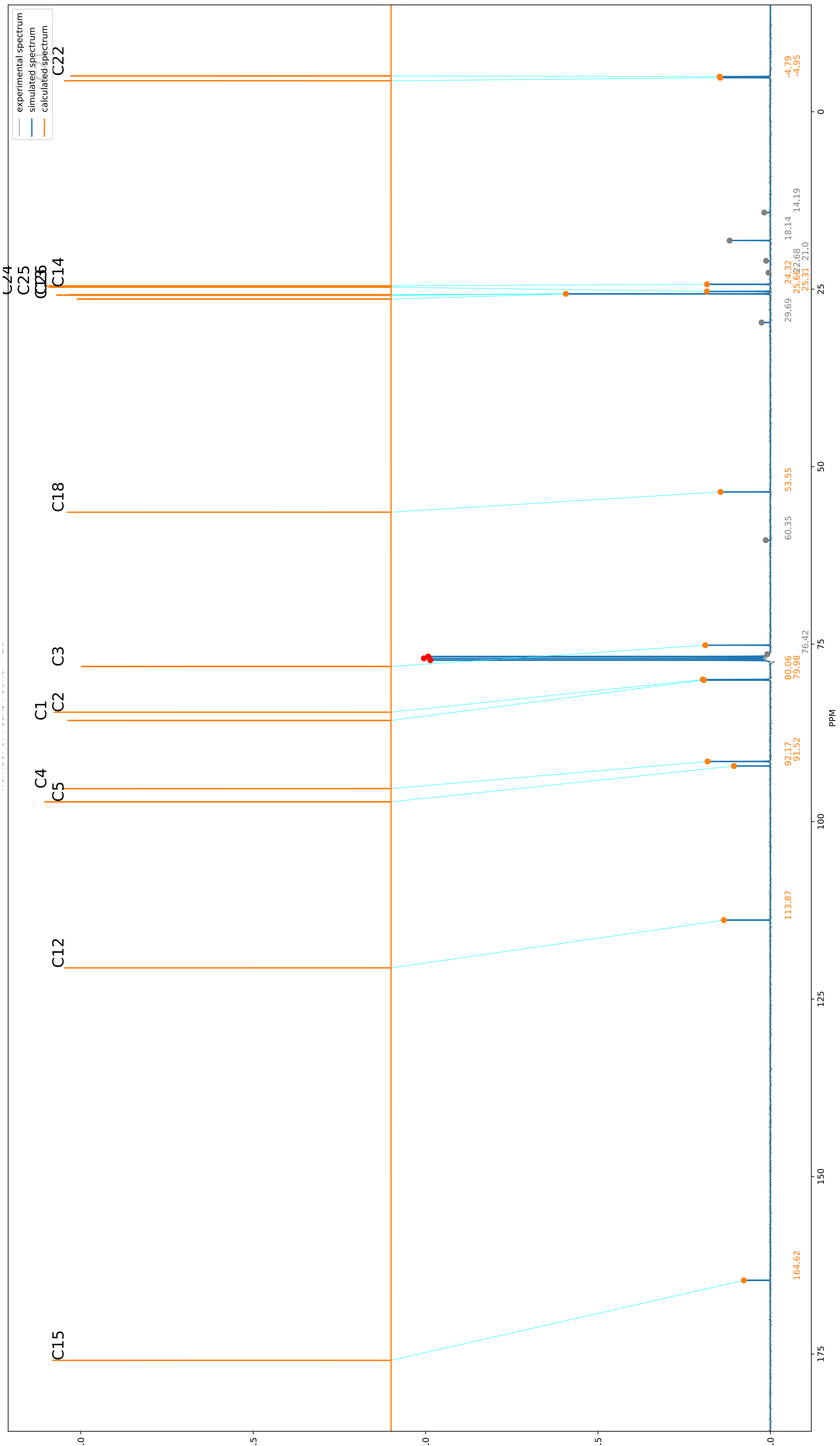

Figure 120: KE3 Carbon Spectra

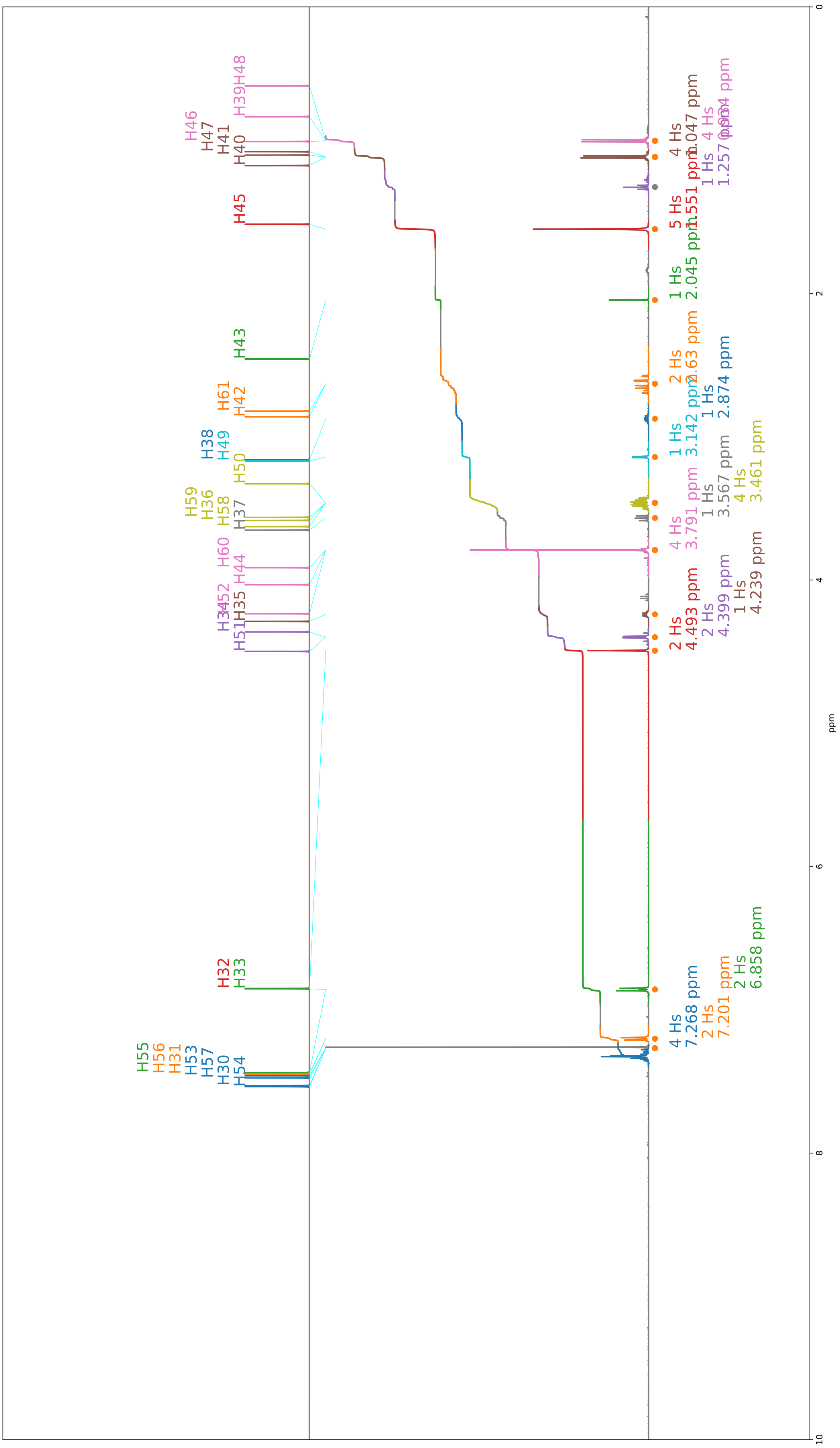

Figure 121: TS1 Proton Spectra

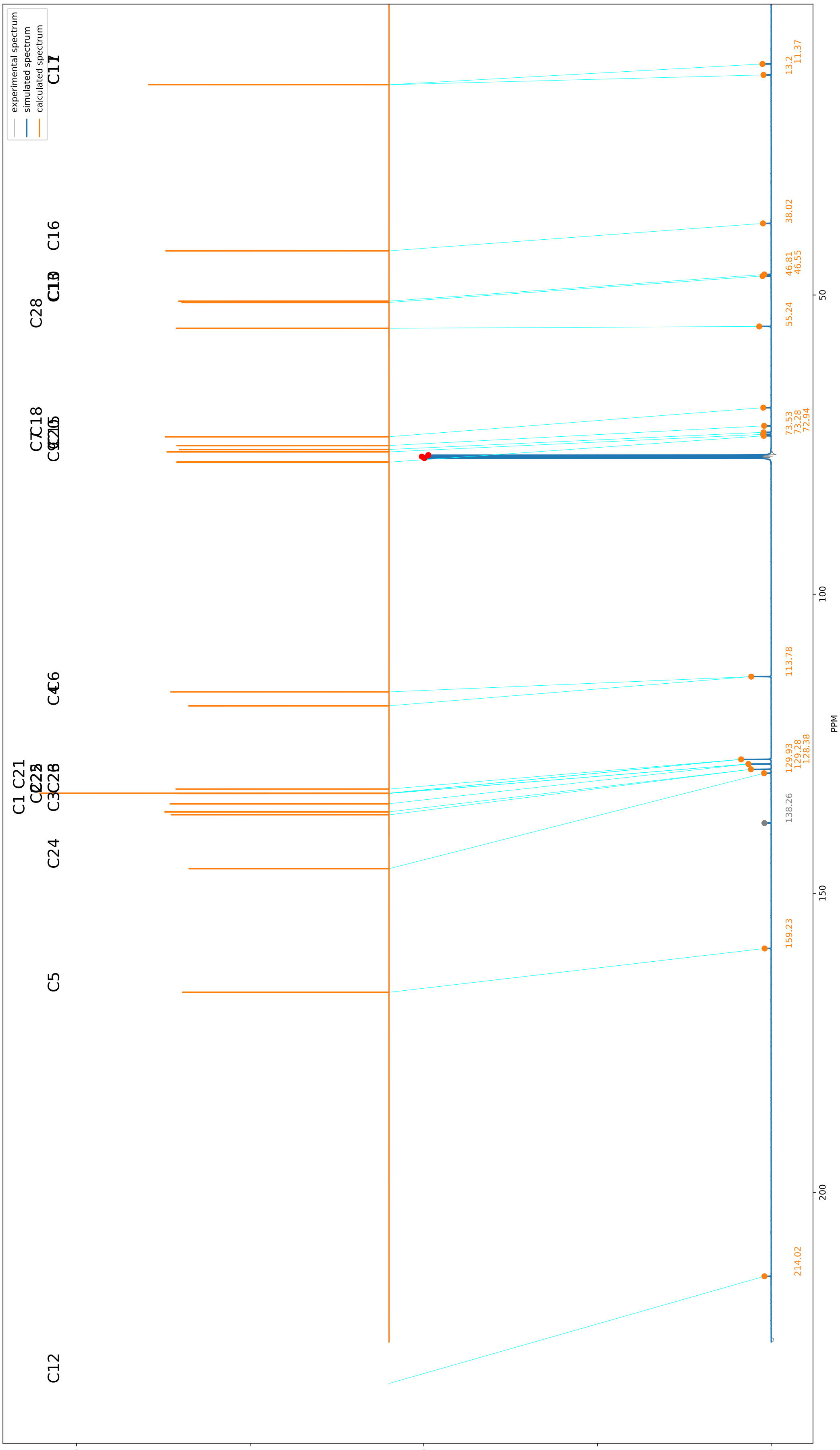

Figure 122: TS1 Carbon Spectra

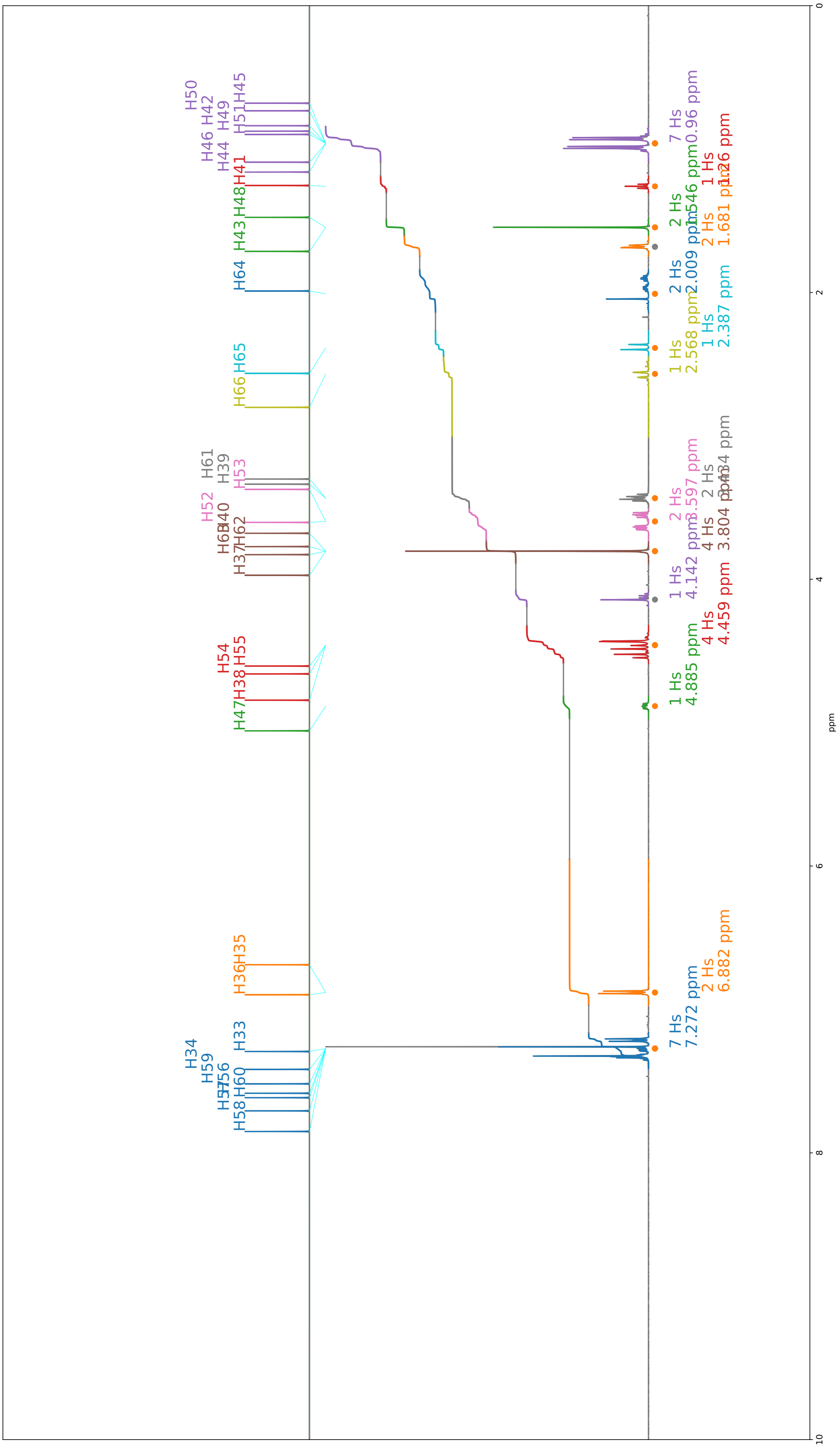

Figure 123: TS3B Proton Spectra

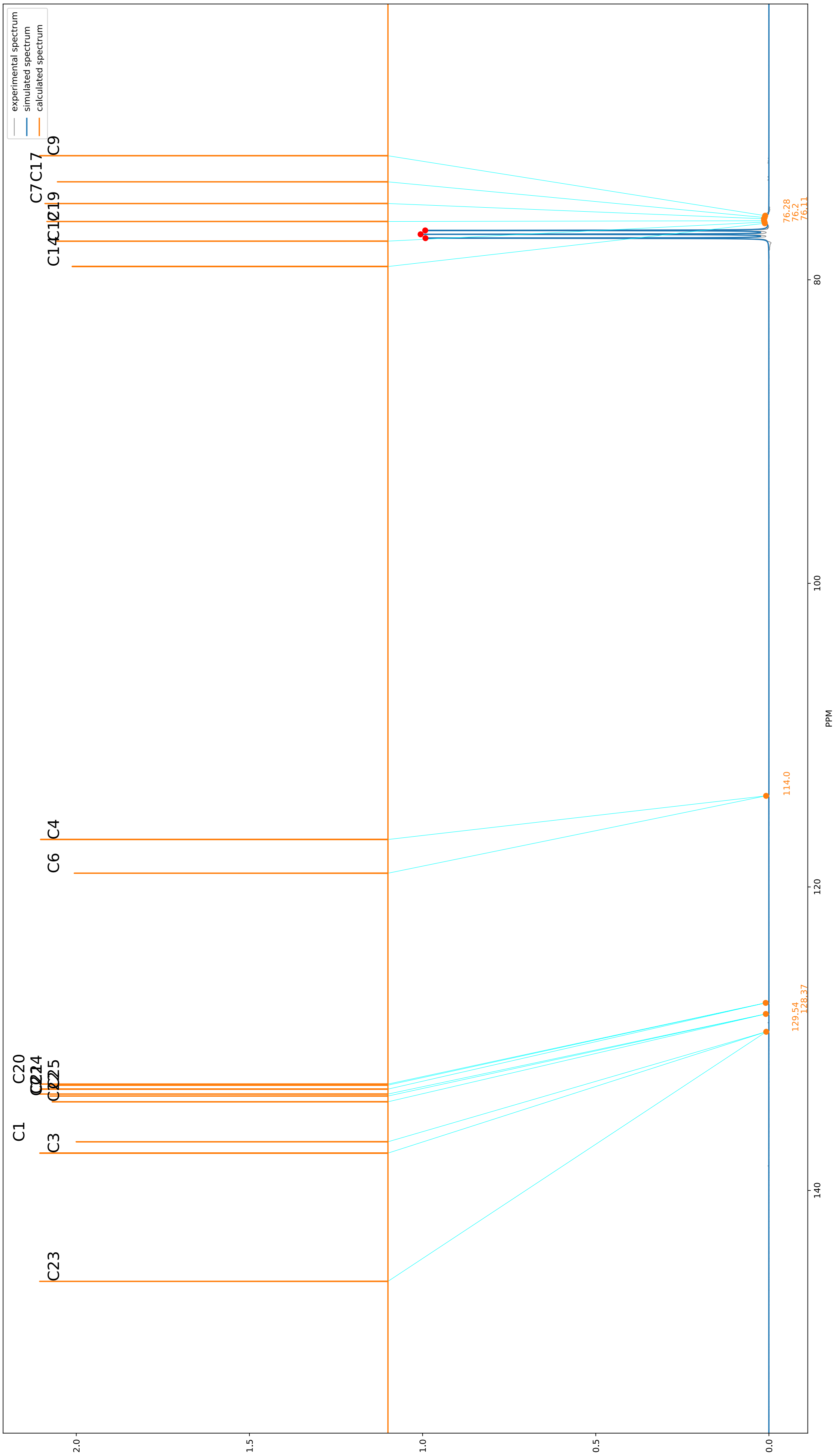

Figure 124: TS3B Carbon Spectra

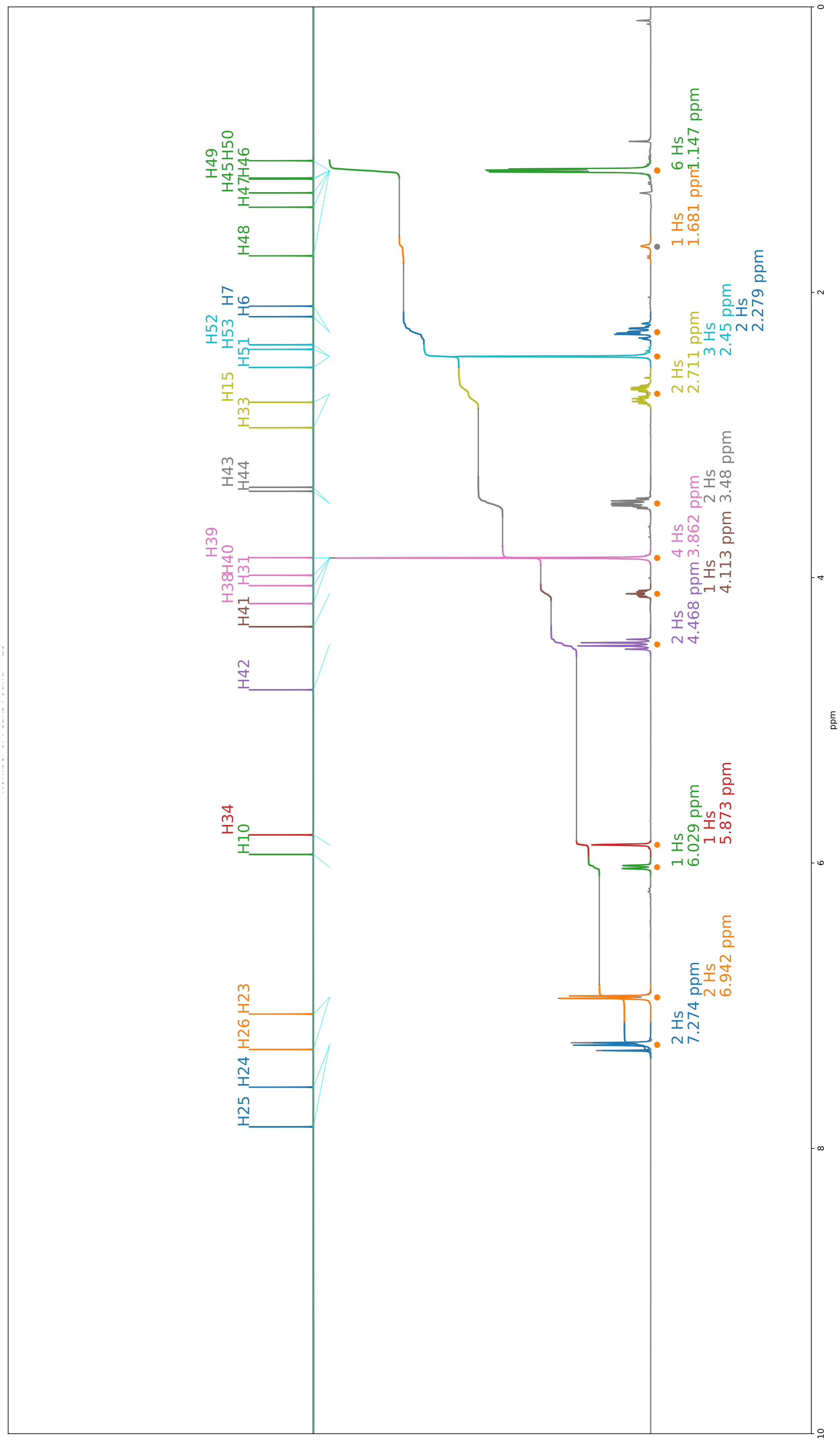

Figure 125: BYH1 Proton Spectra

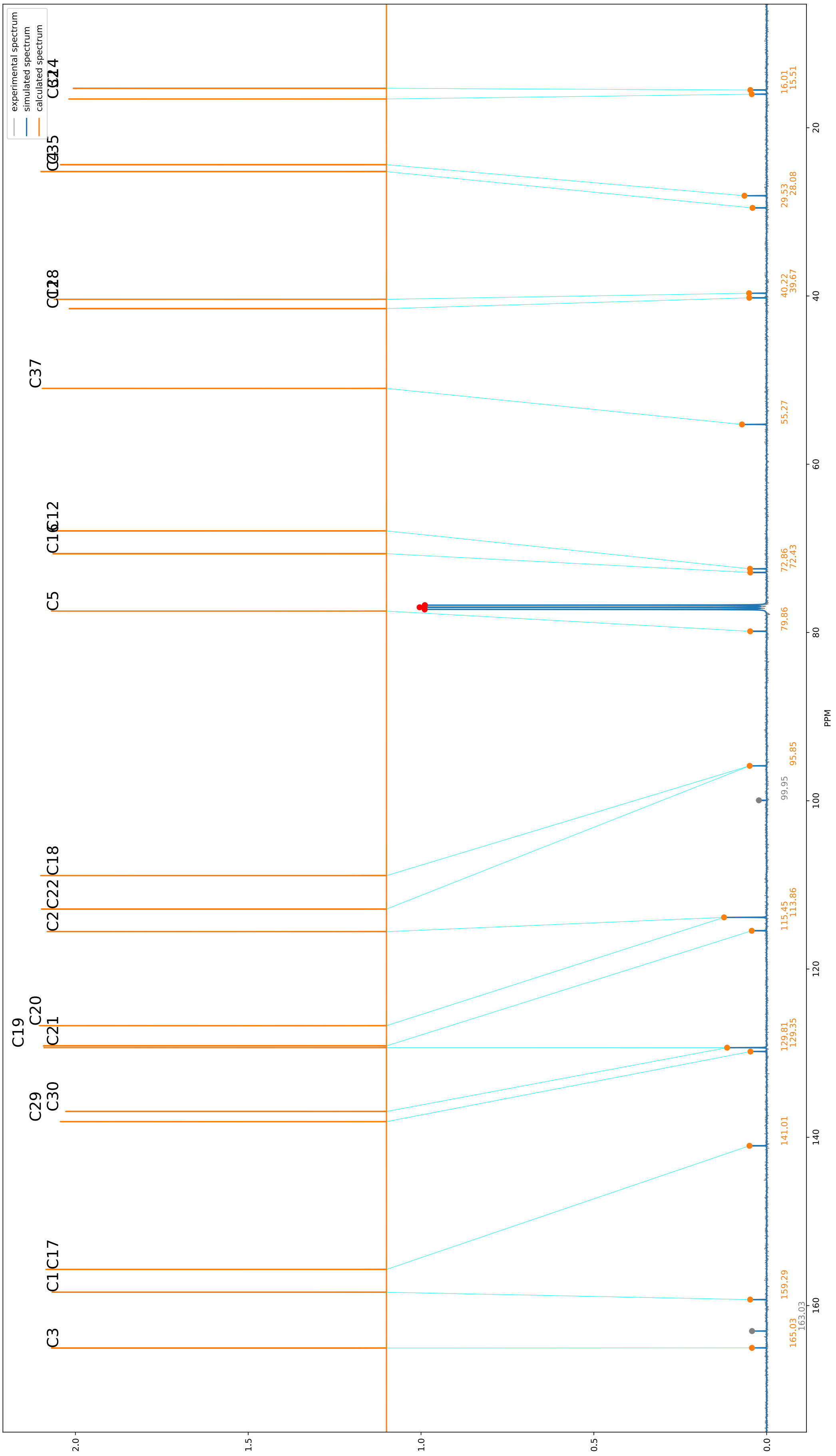

Figure 126: BYH1 Carbon Spectra

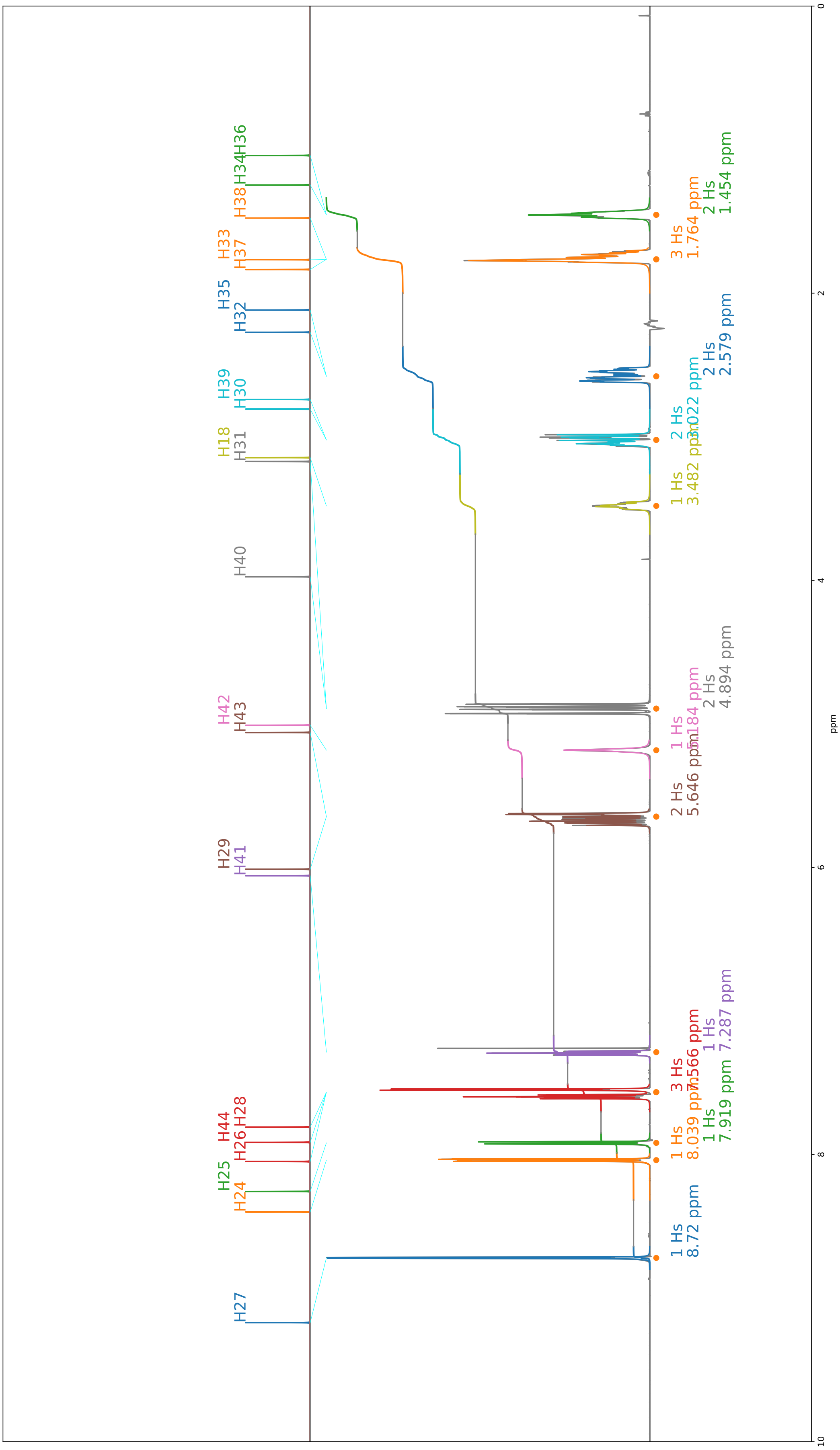

Figure 127: NP4 Proton Spectra

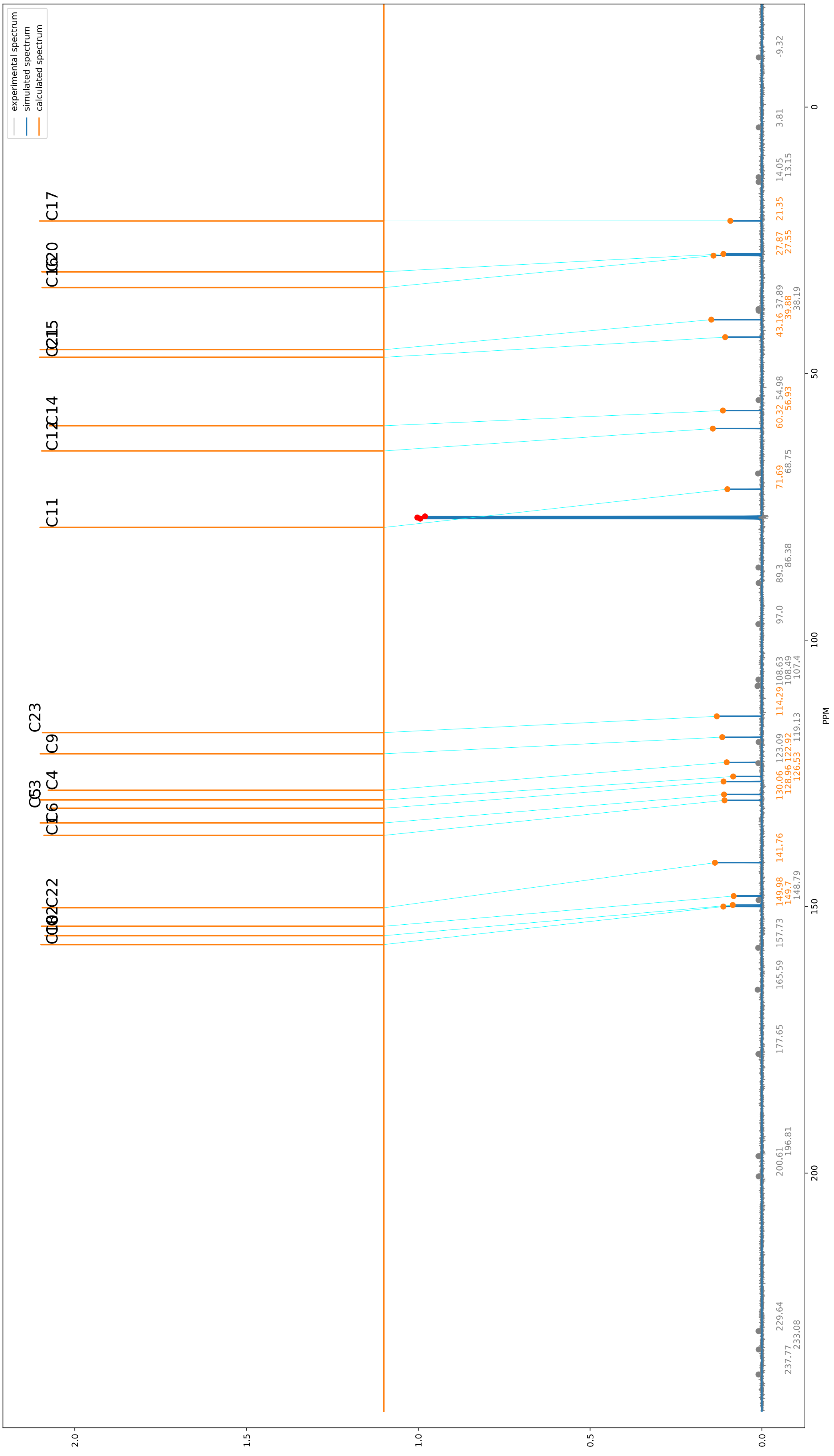

Figure 128: NP4 Carbon Spectra

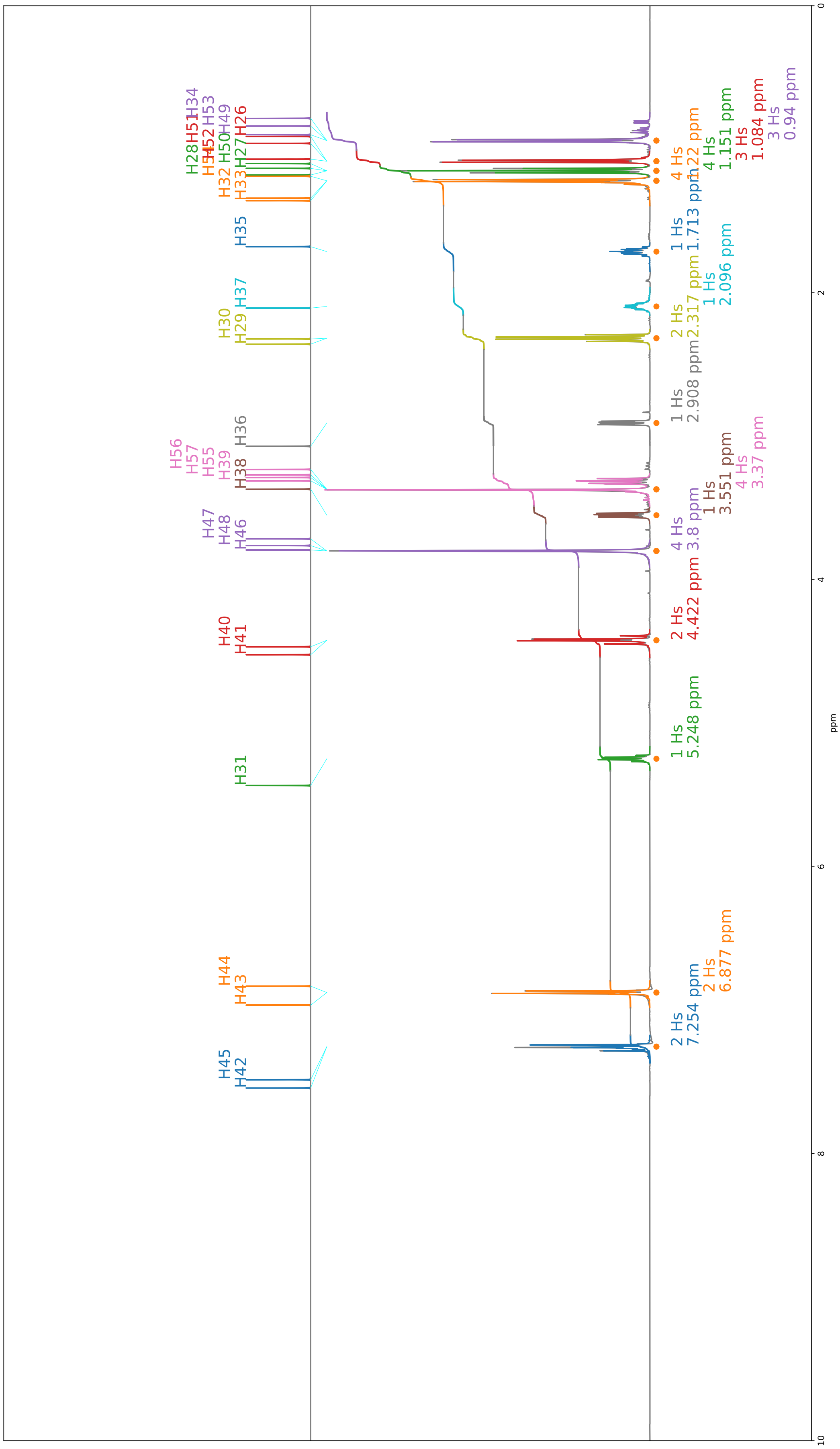

Figure 129: TP3 Proton Spectra

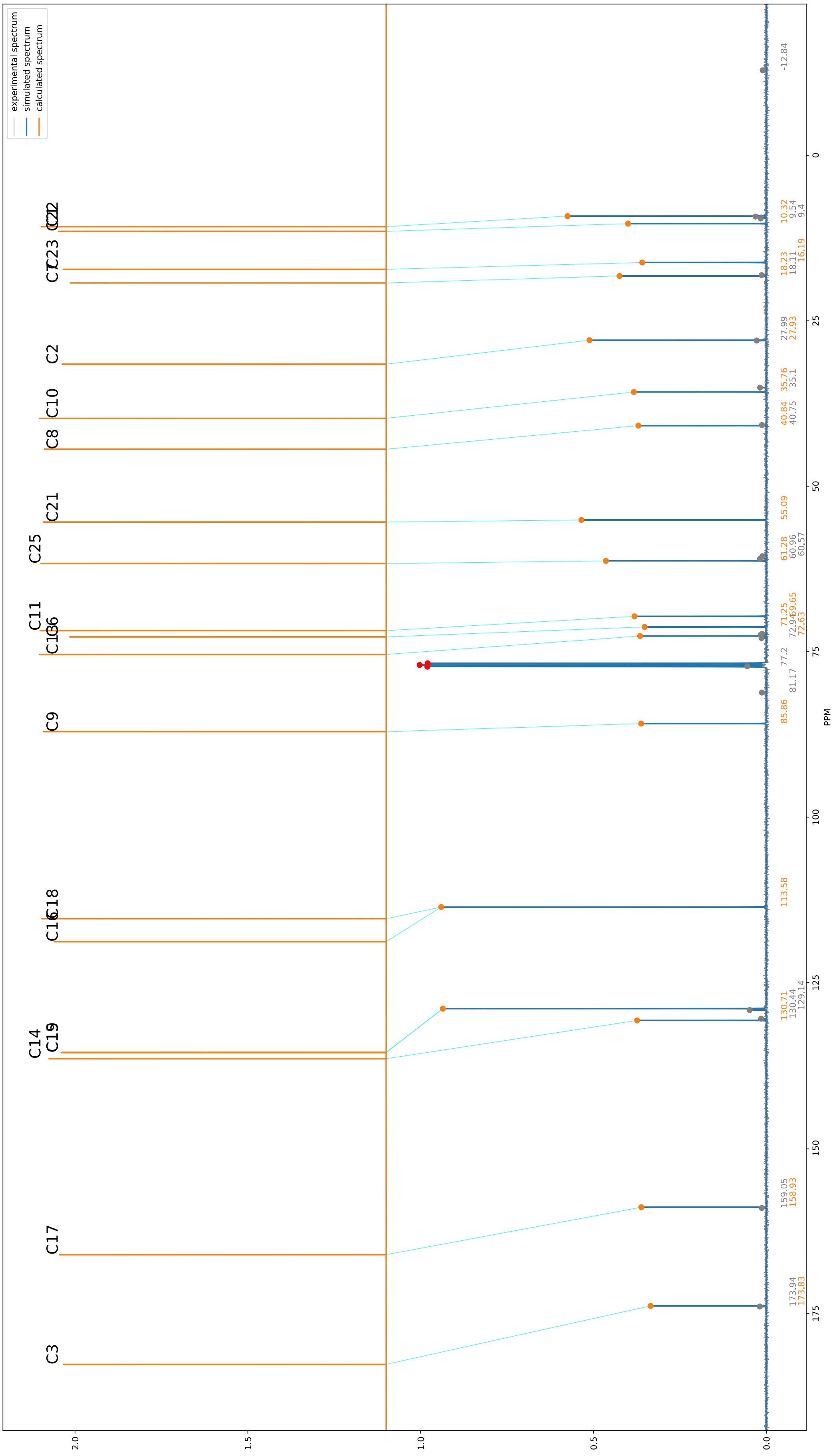

Figure 130: TP3 Carbon Spectra

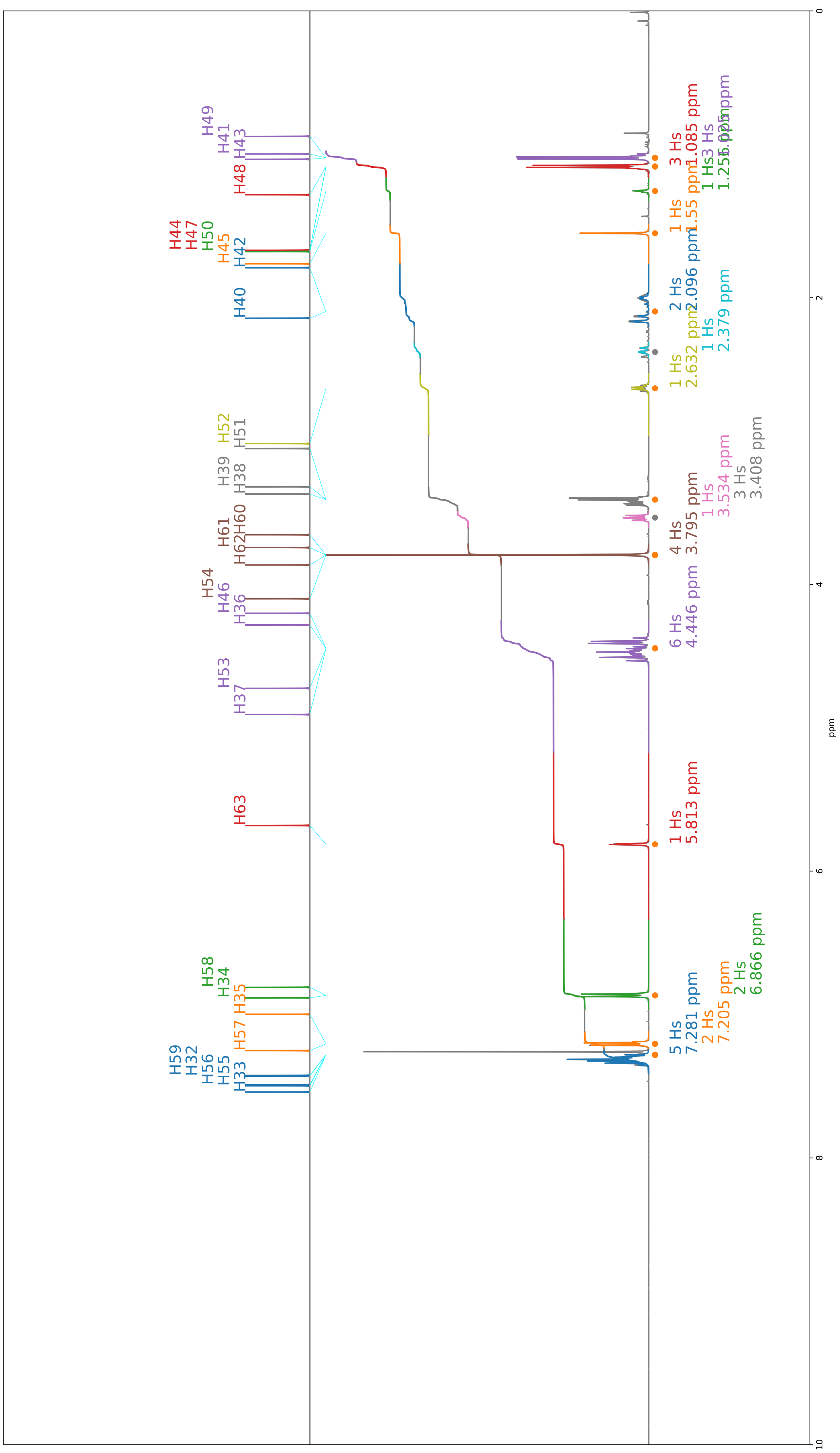

Figure 131: TS2 Proton Spectra

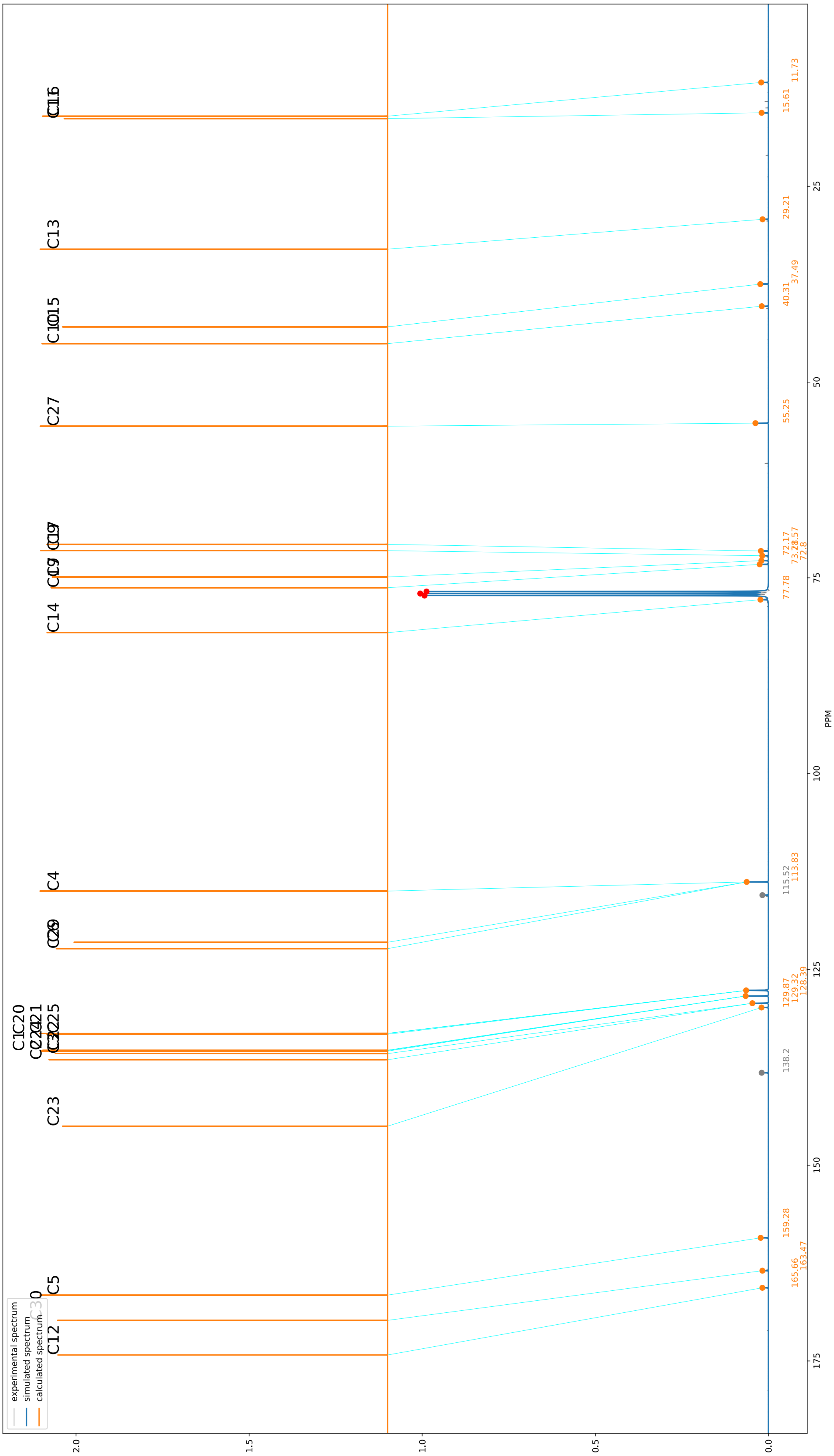

Figure 132: TS2 Carbon Spectra

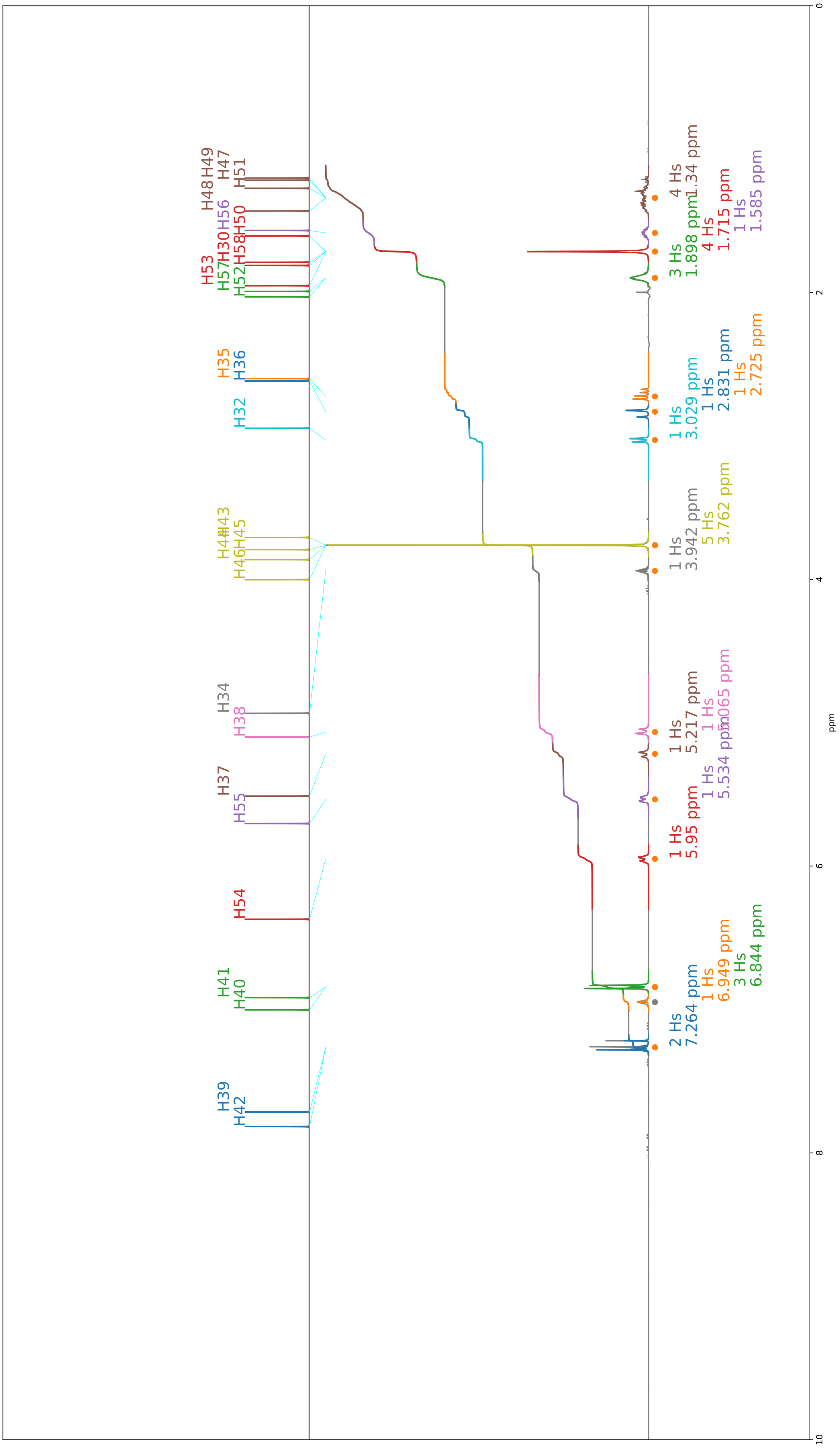

Figure 133: JB6 Proton Spectra

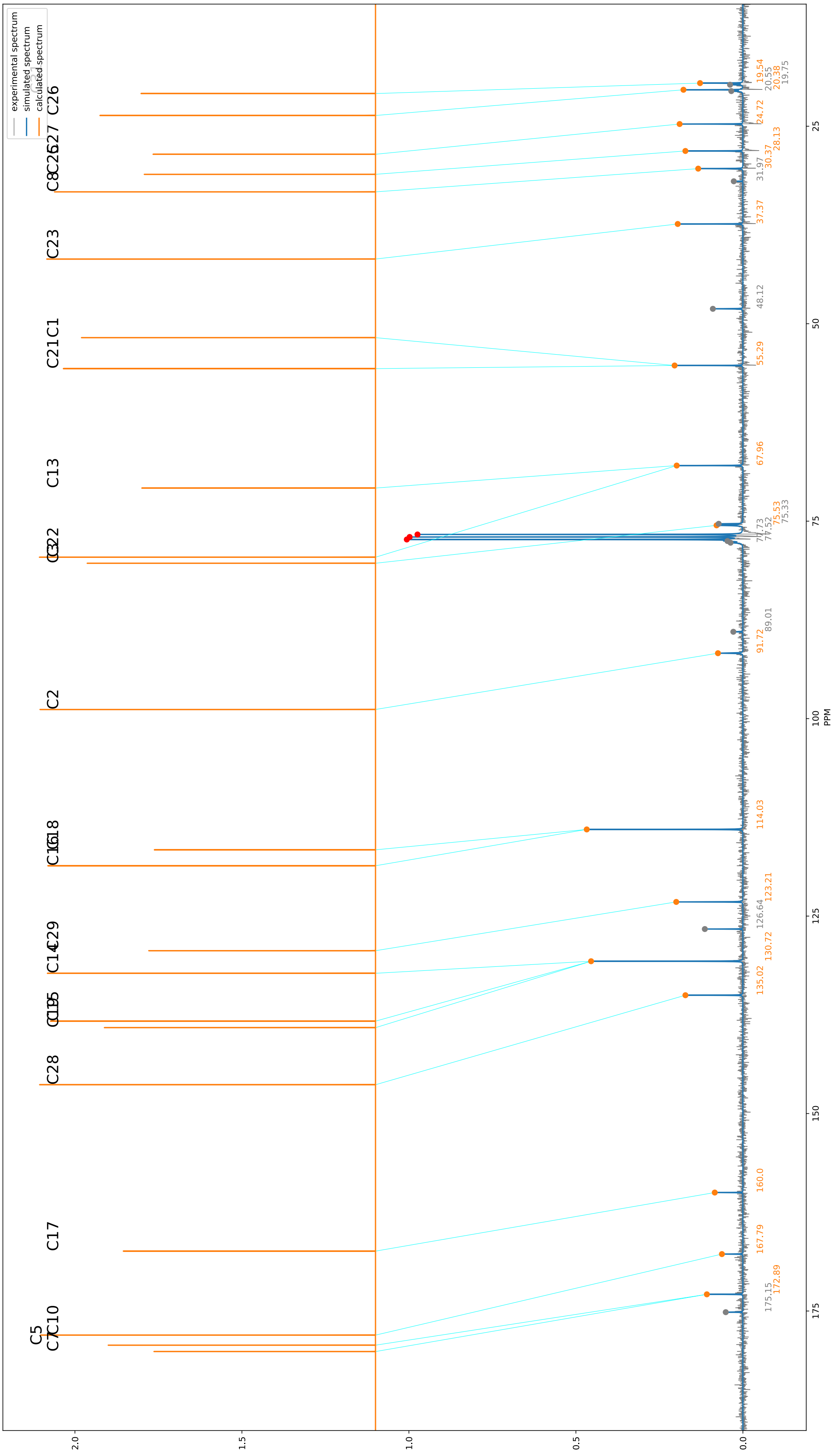

Figure 134: JB6 Carbon Spectra

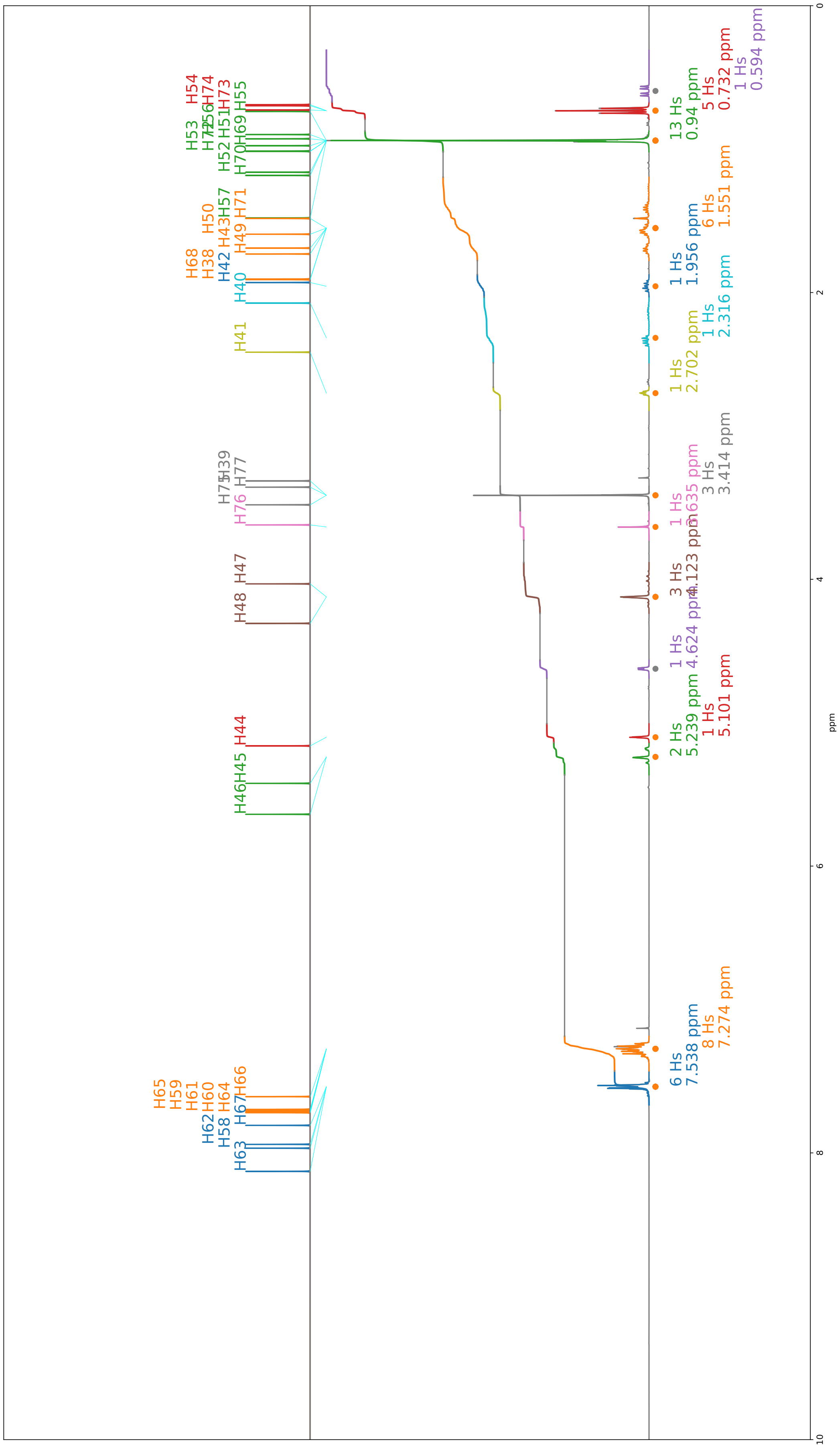

Figure 135: JB12 Proton Spectra

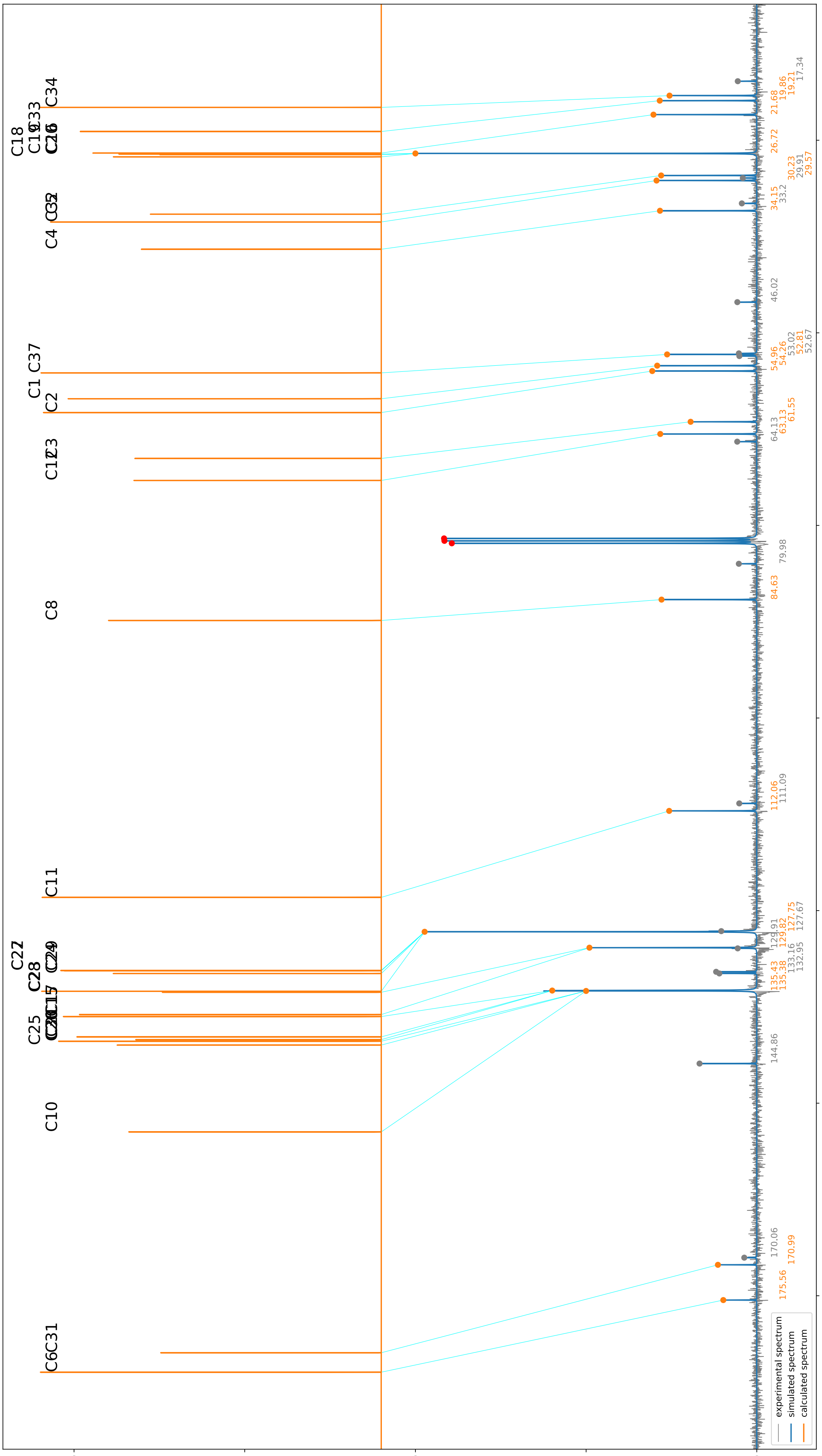

Figure 136: JB12 Carbon Spectra

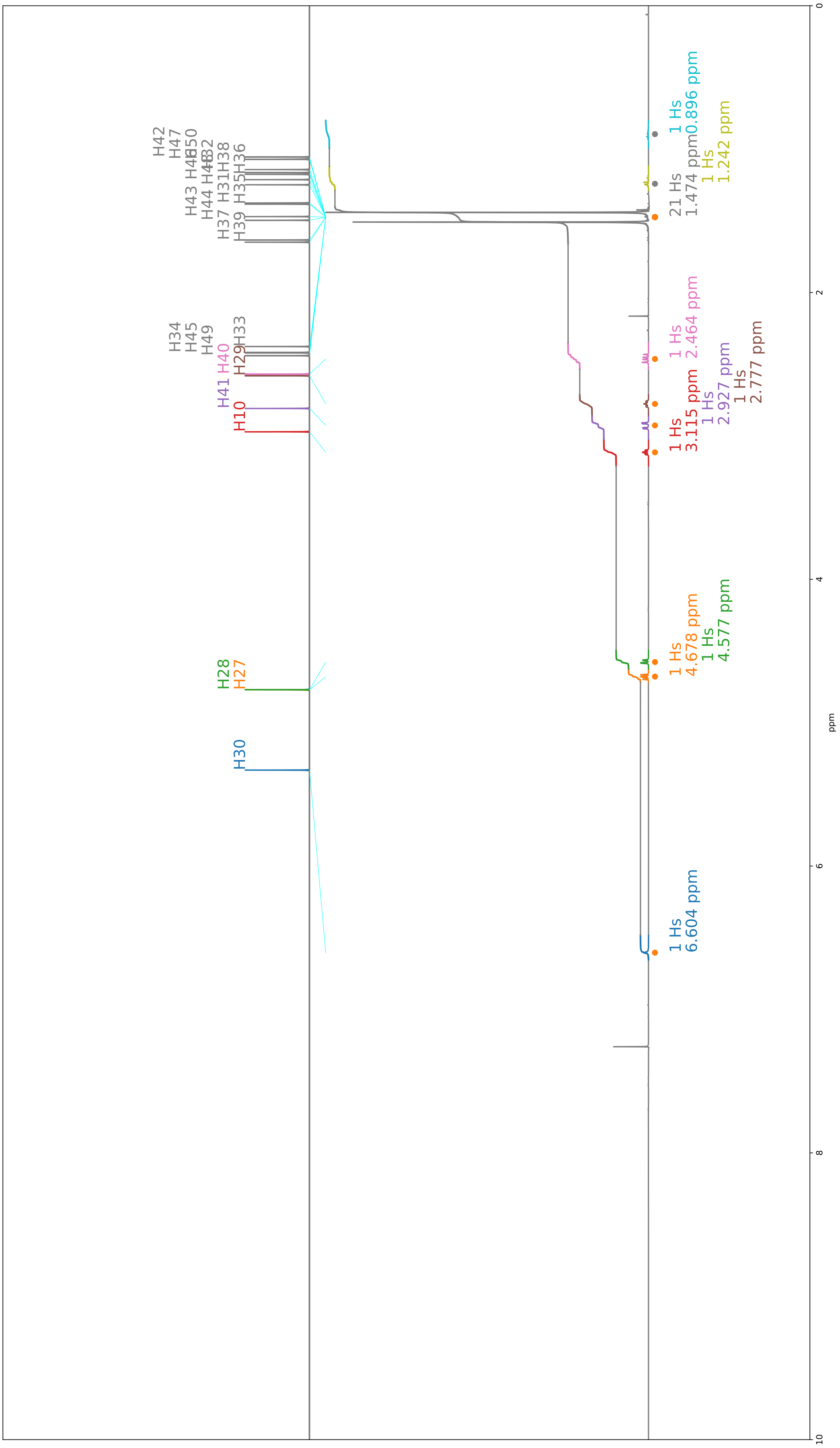

Figure 137: JB2 Proton Spectra

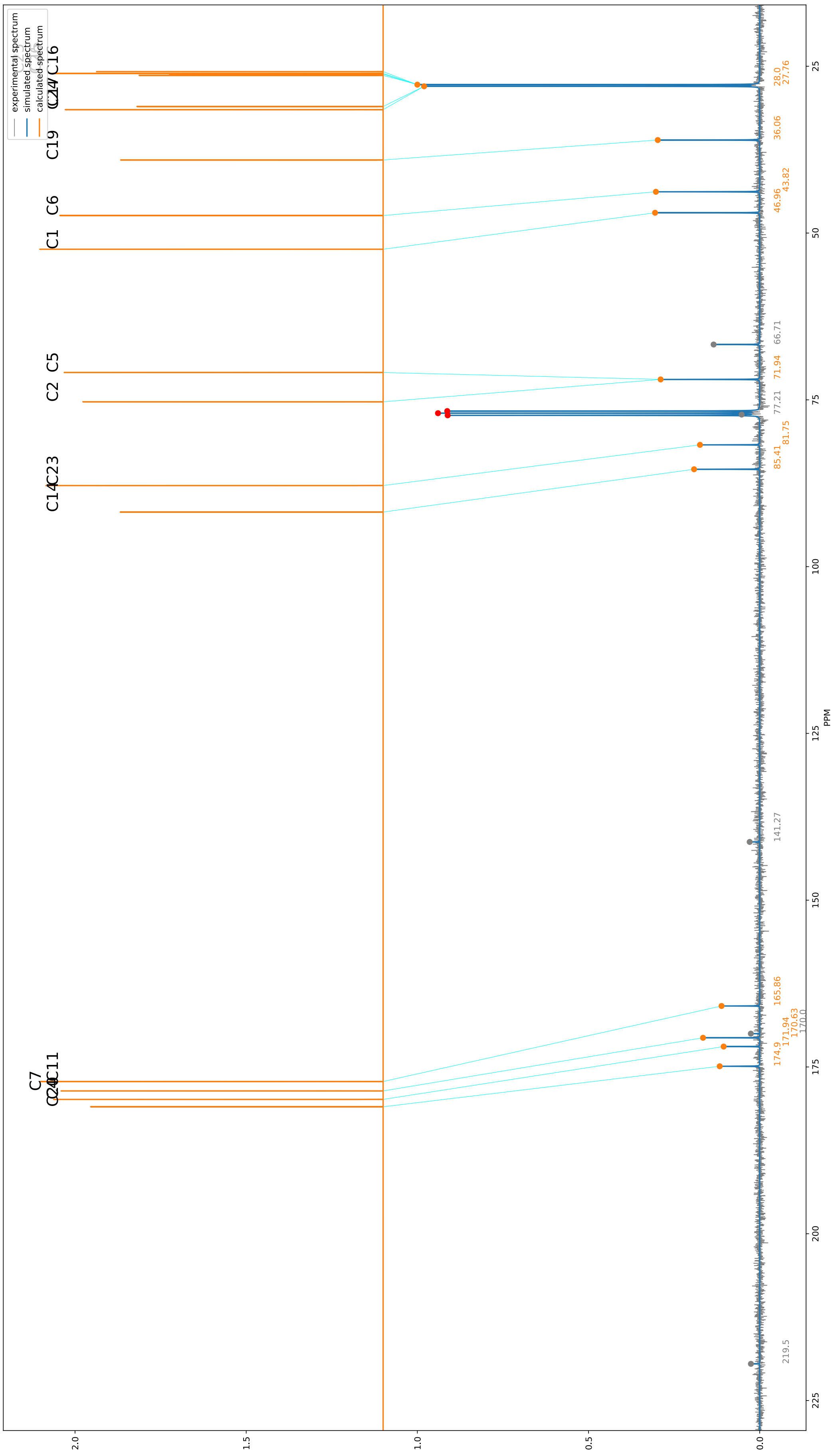

Figure 138: JB2 Carbon Spectra

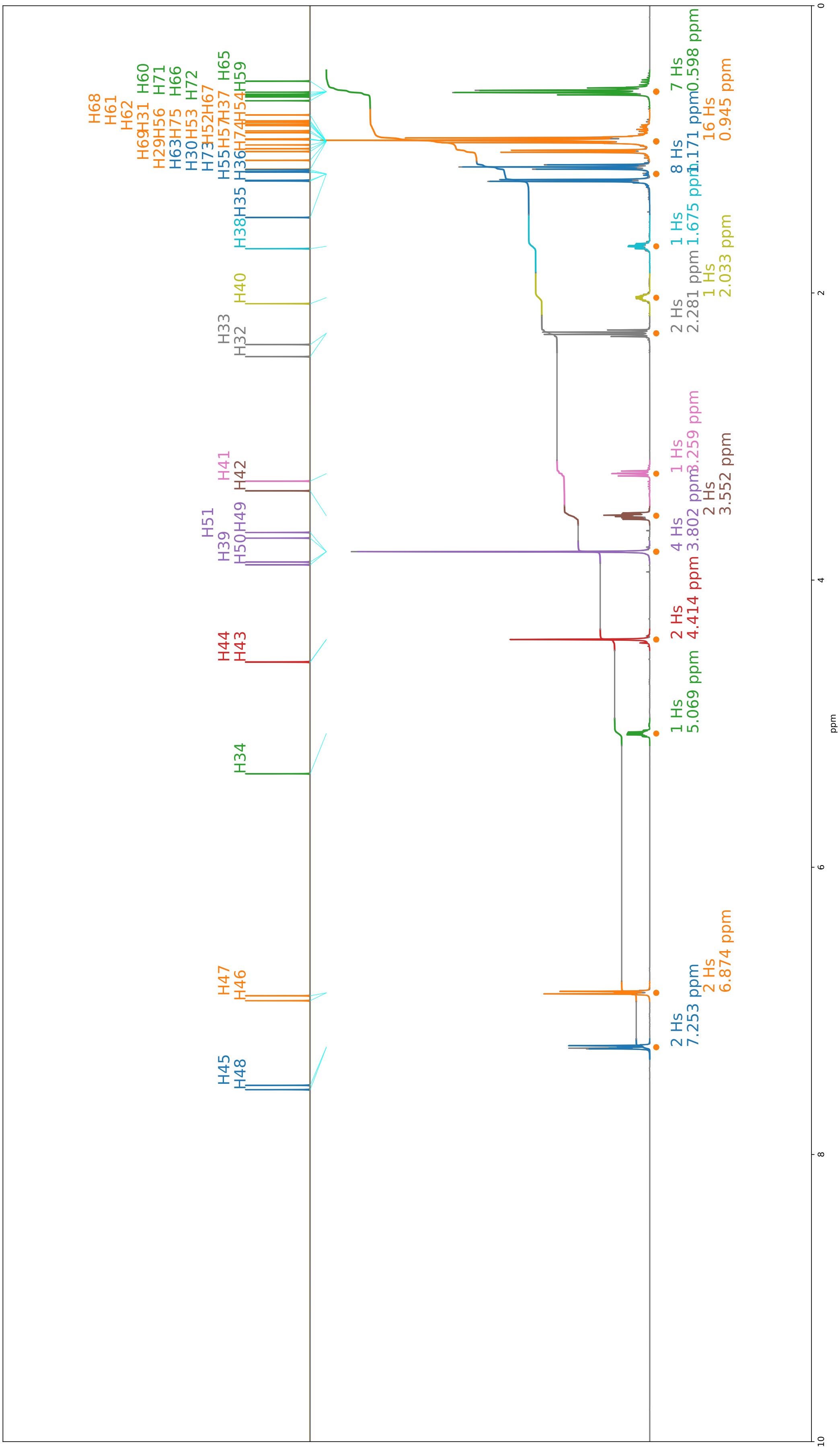

Figure 139: TP2 Proton Spectra

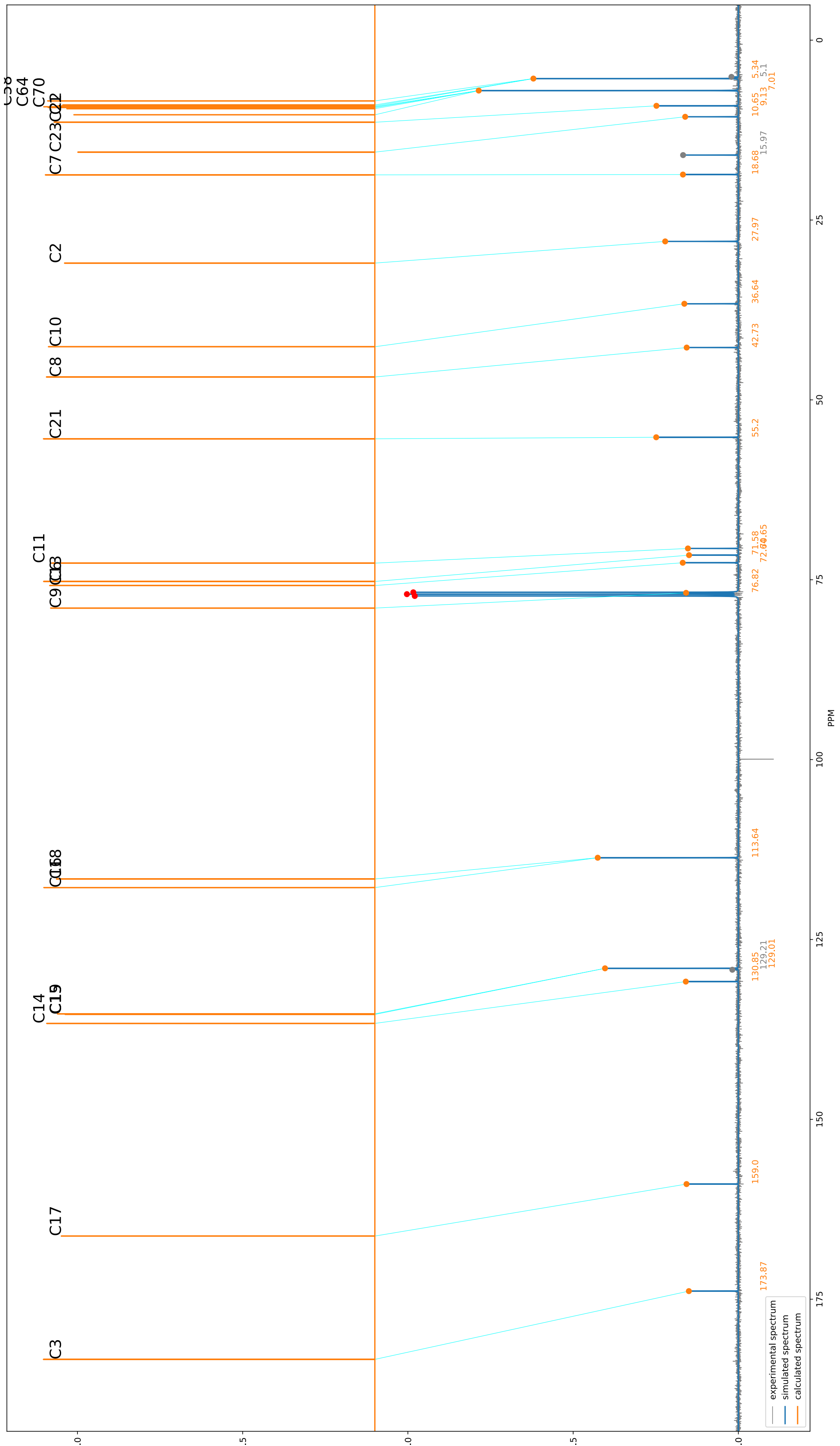

Figure 140: TP2 Carbon Spectra

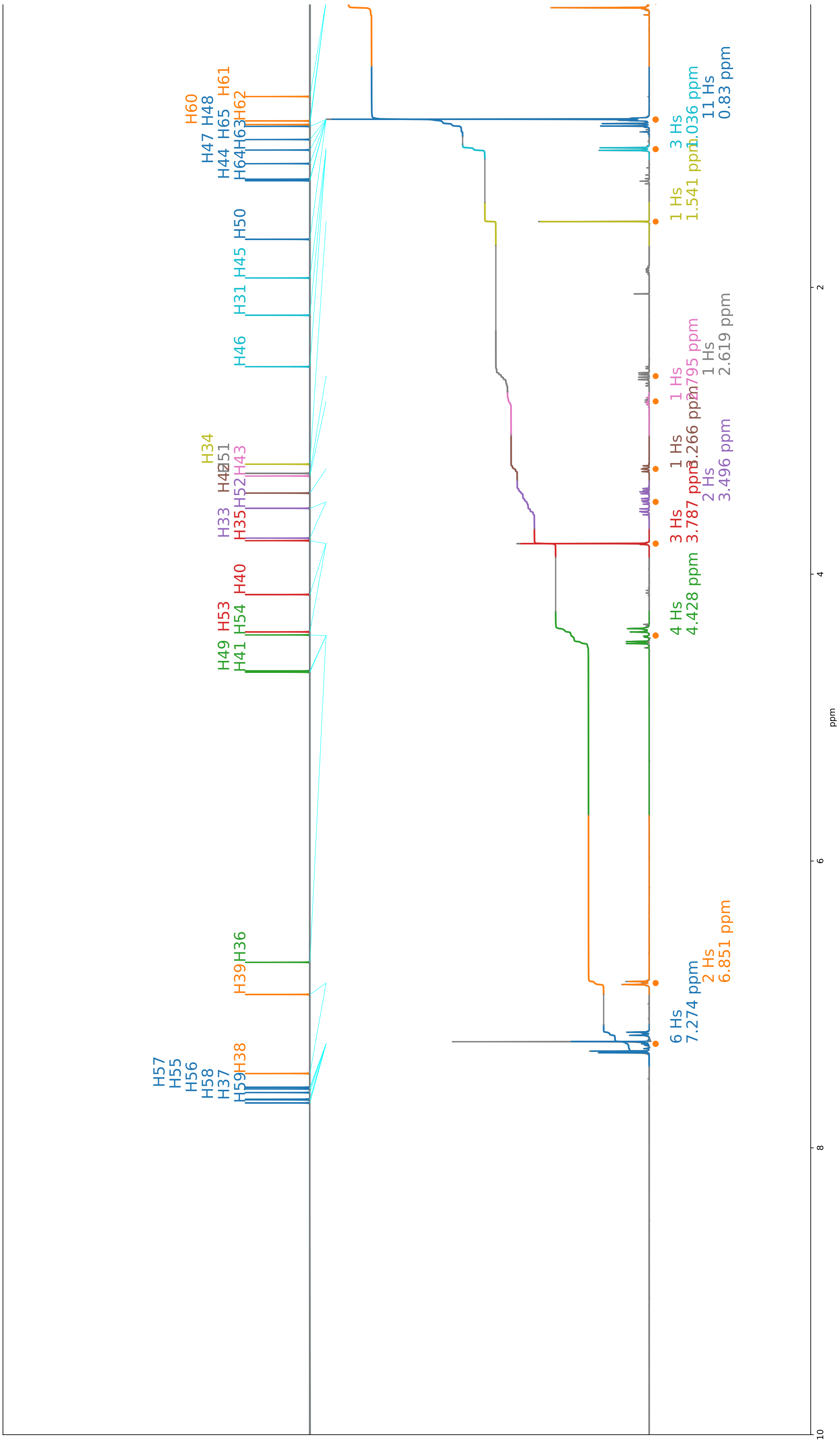

Figure 141: TS4 Proton Spectra

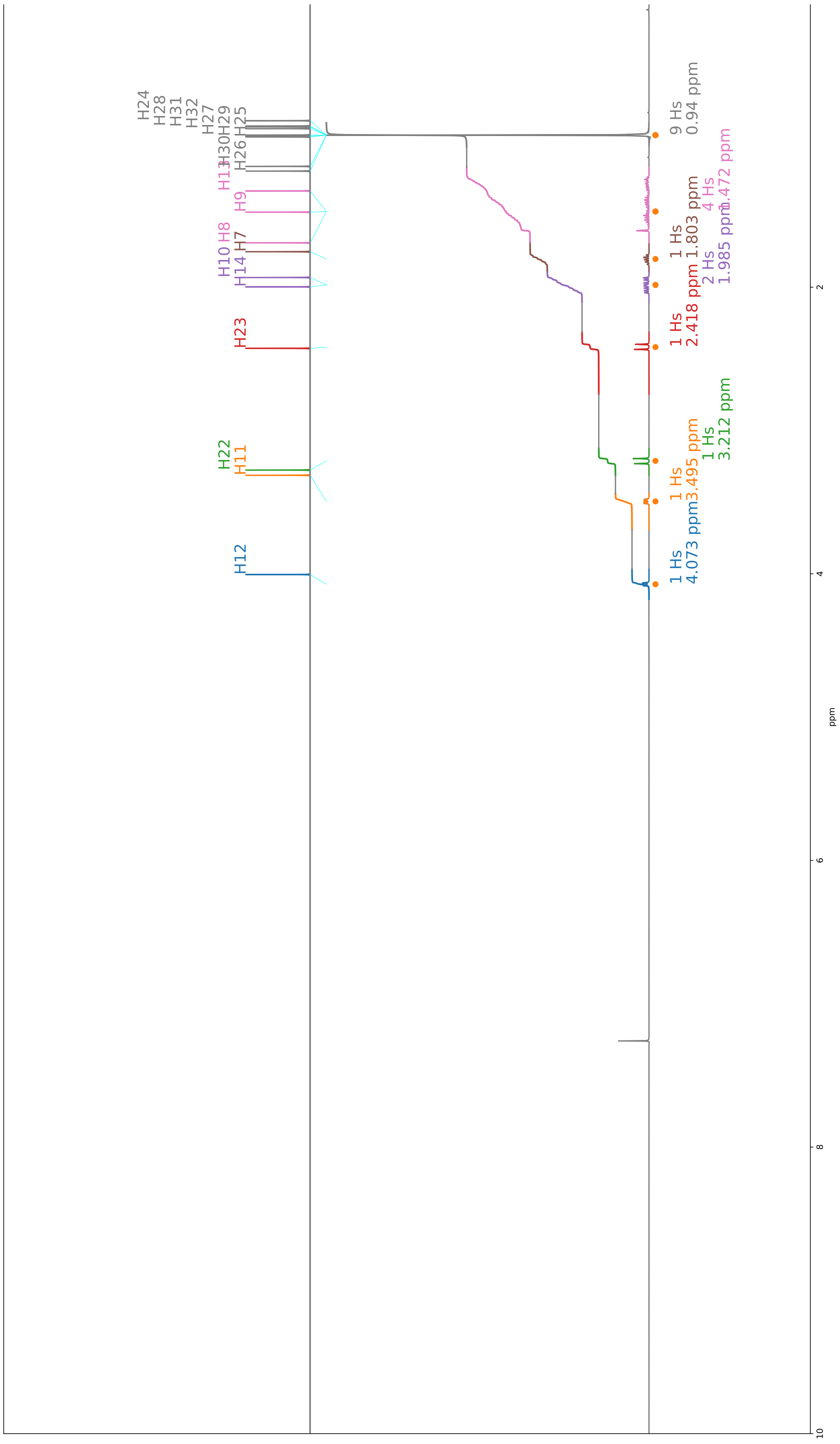

Figure 142: AT2 Proton Spectra

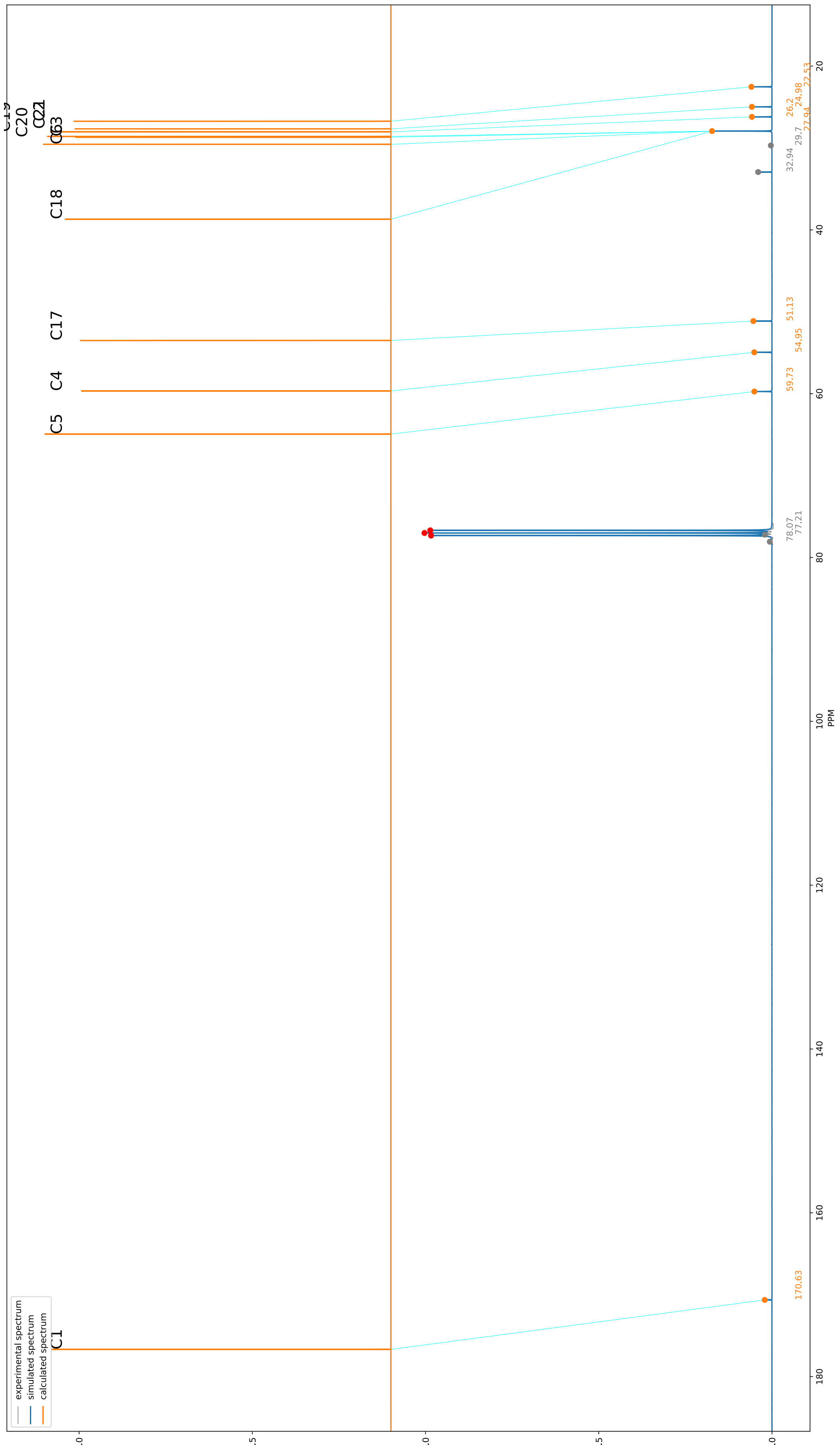

Figure 143: AT2 Carbon Spectra

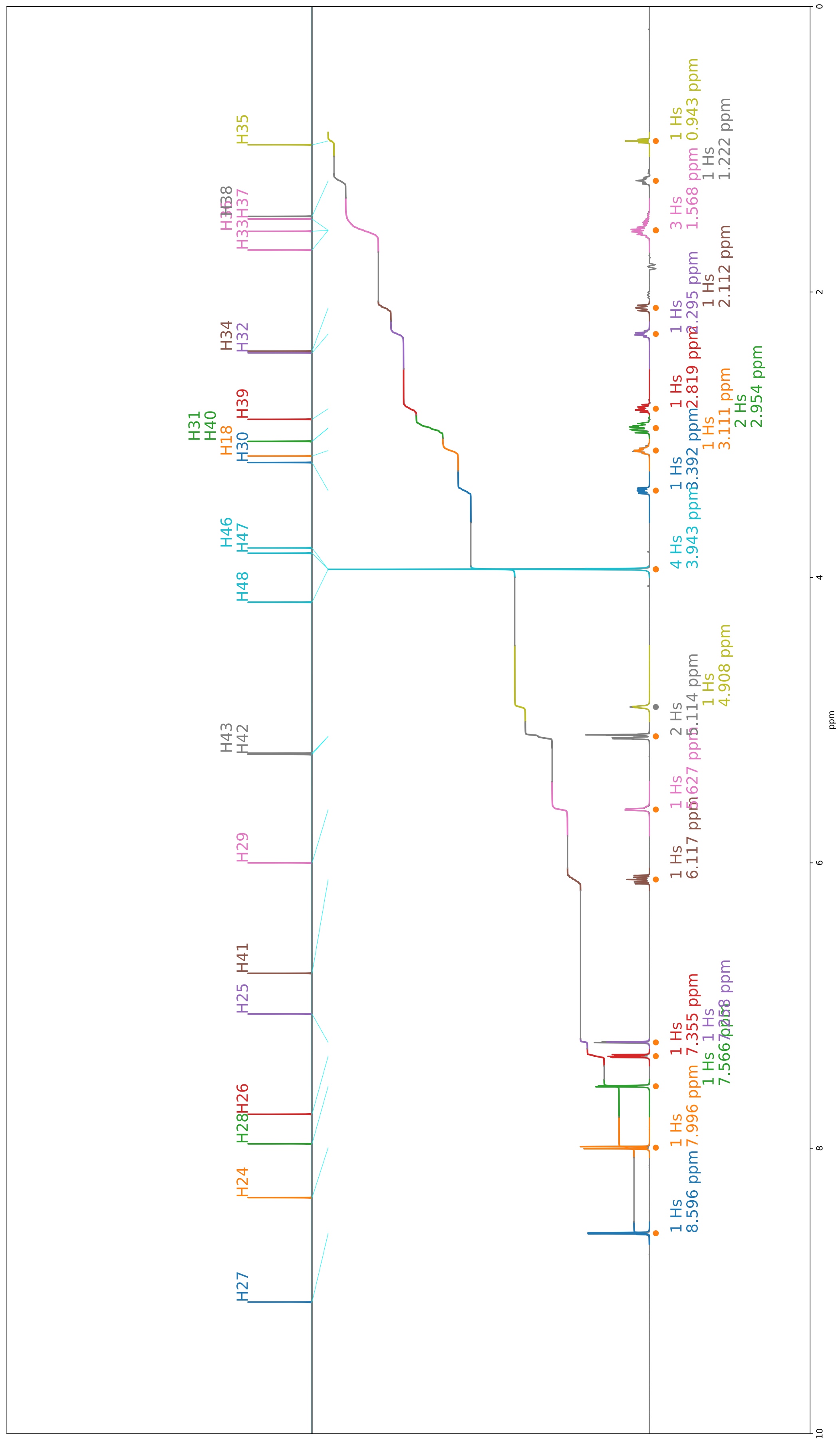

Figure 144: NP3B Proton Spectra

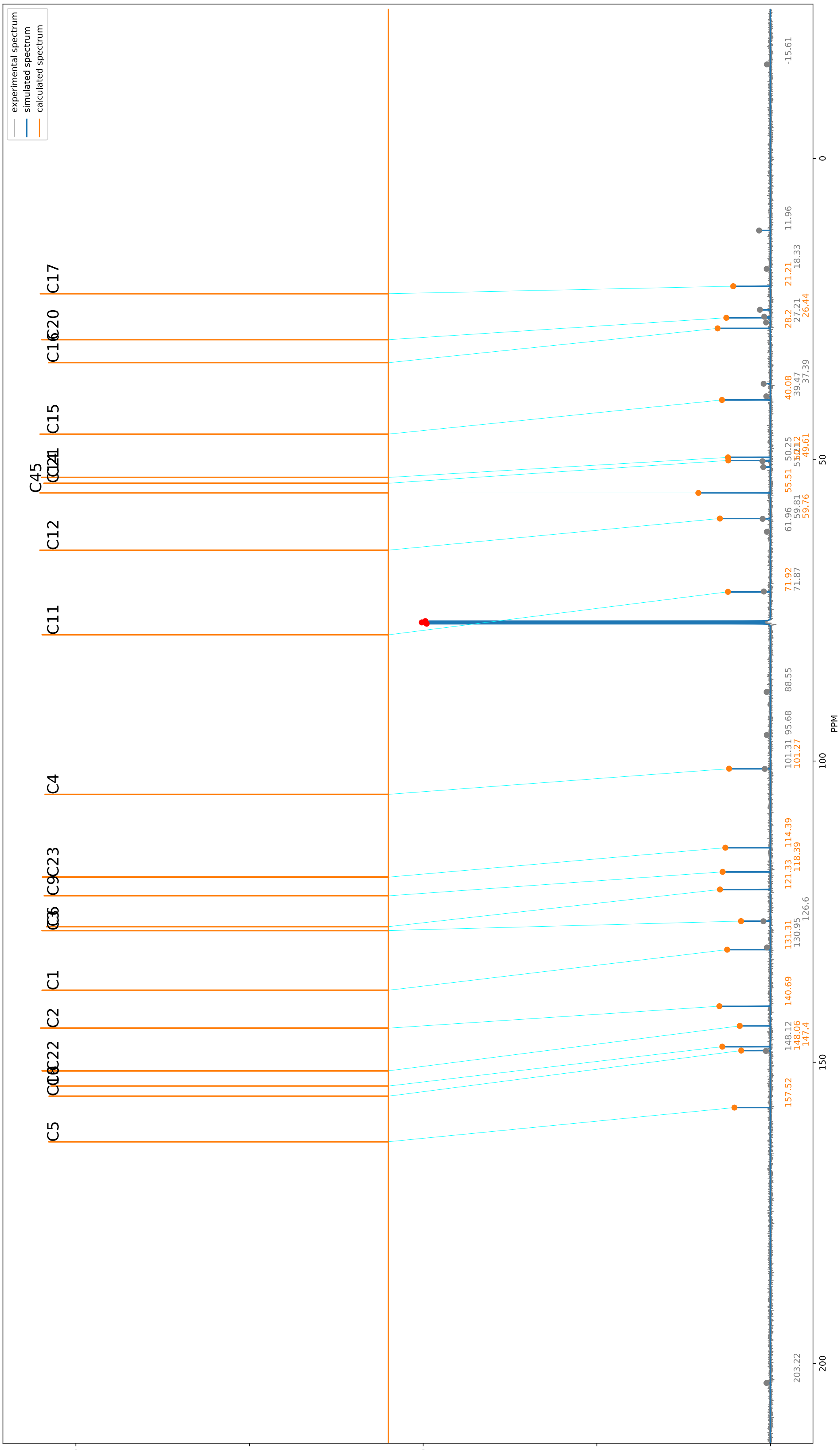

Figure 145: NP3B Carbon Spectra

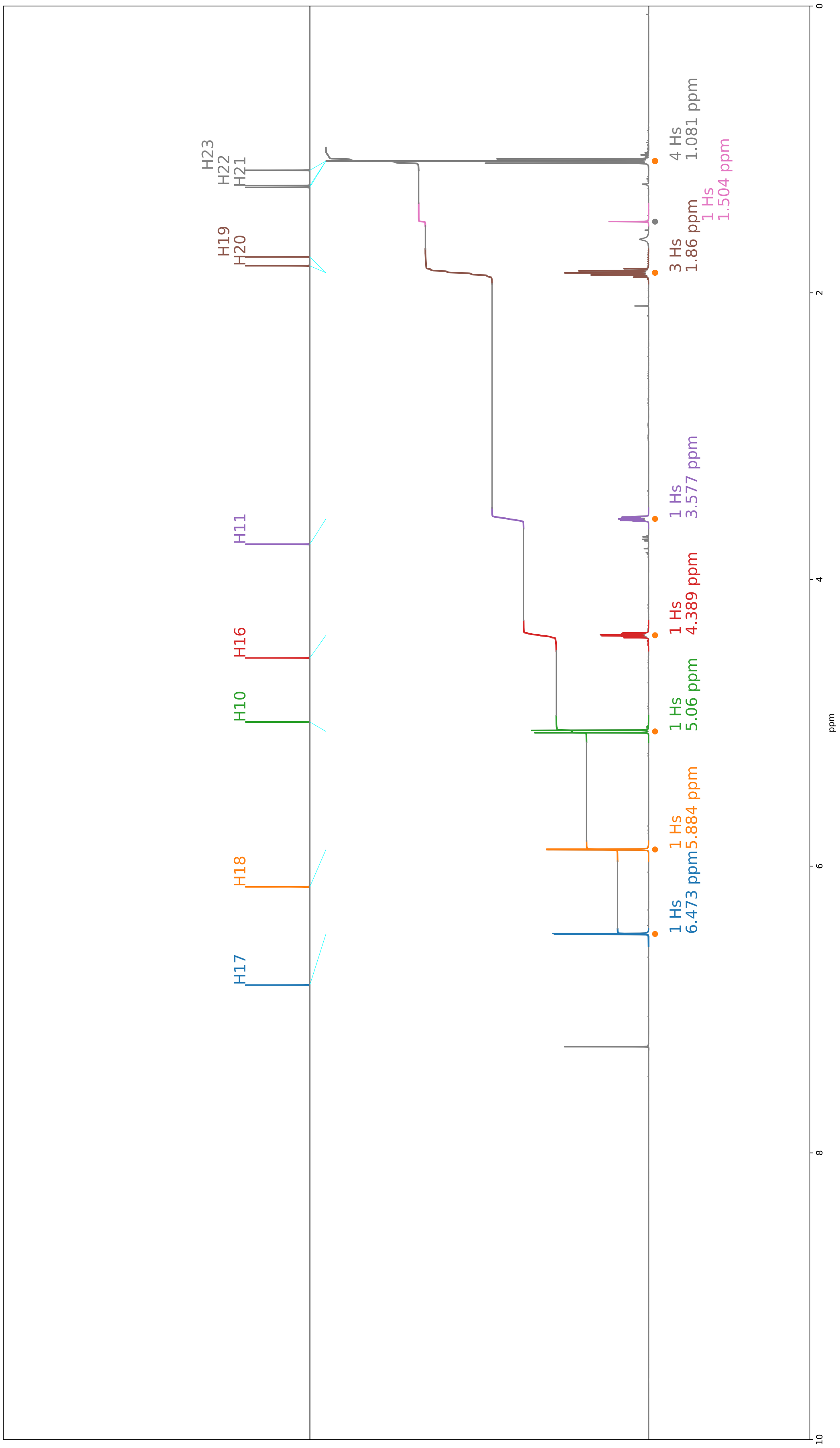

Figure 146: JB13B Proton Spectra

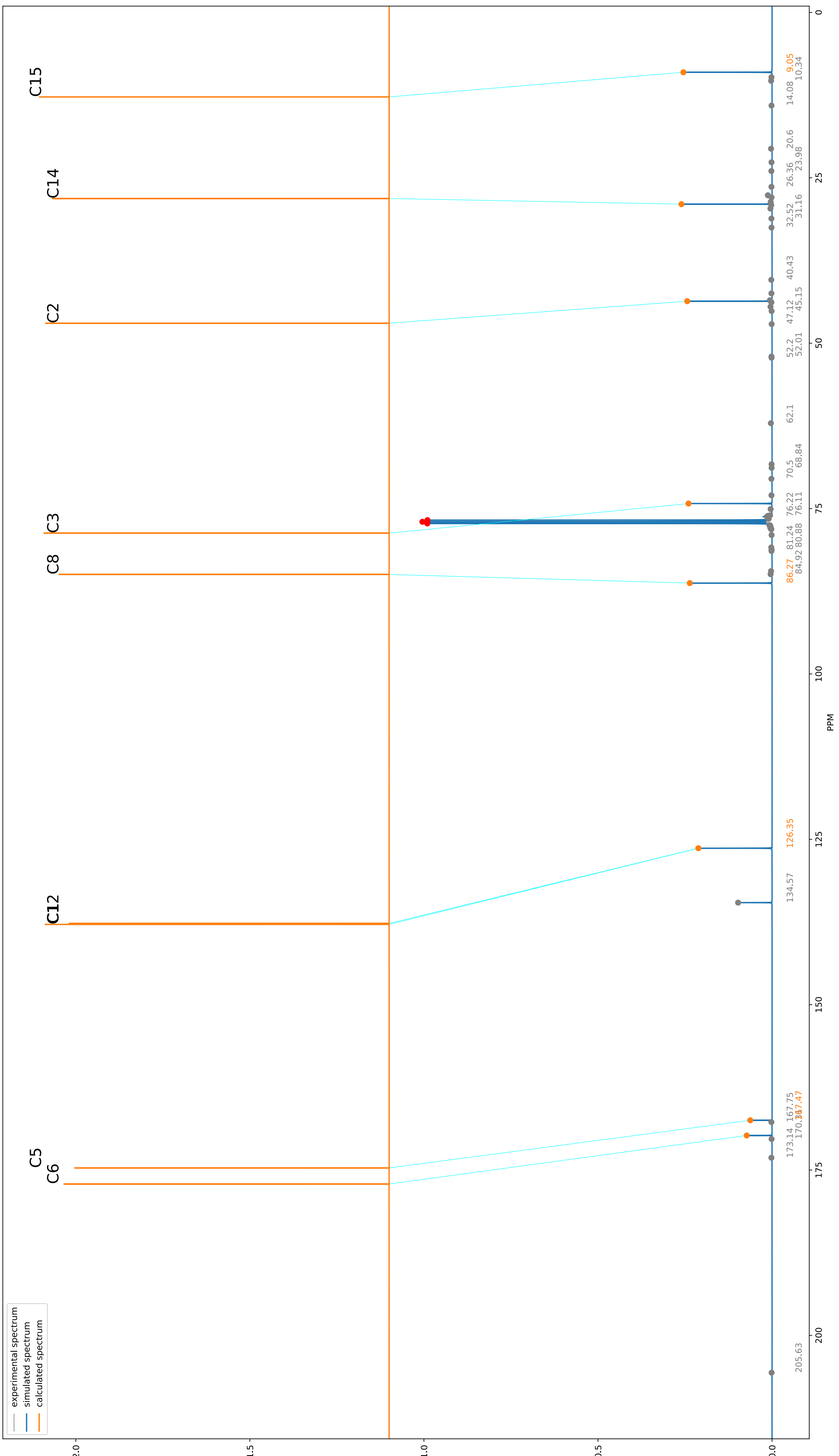

Figure 147: JB13B Carbon Spectra

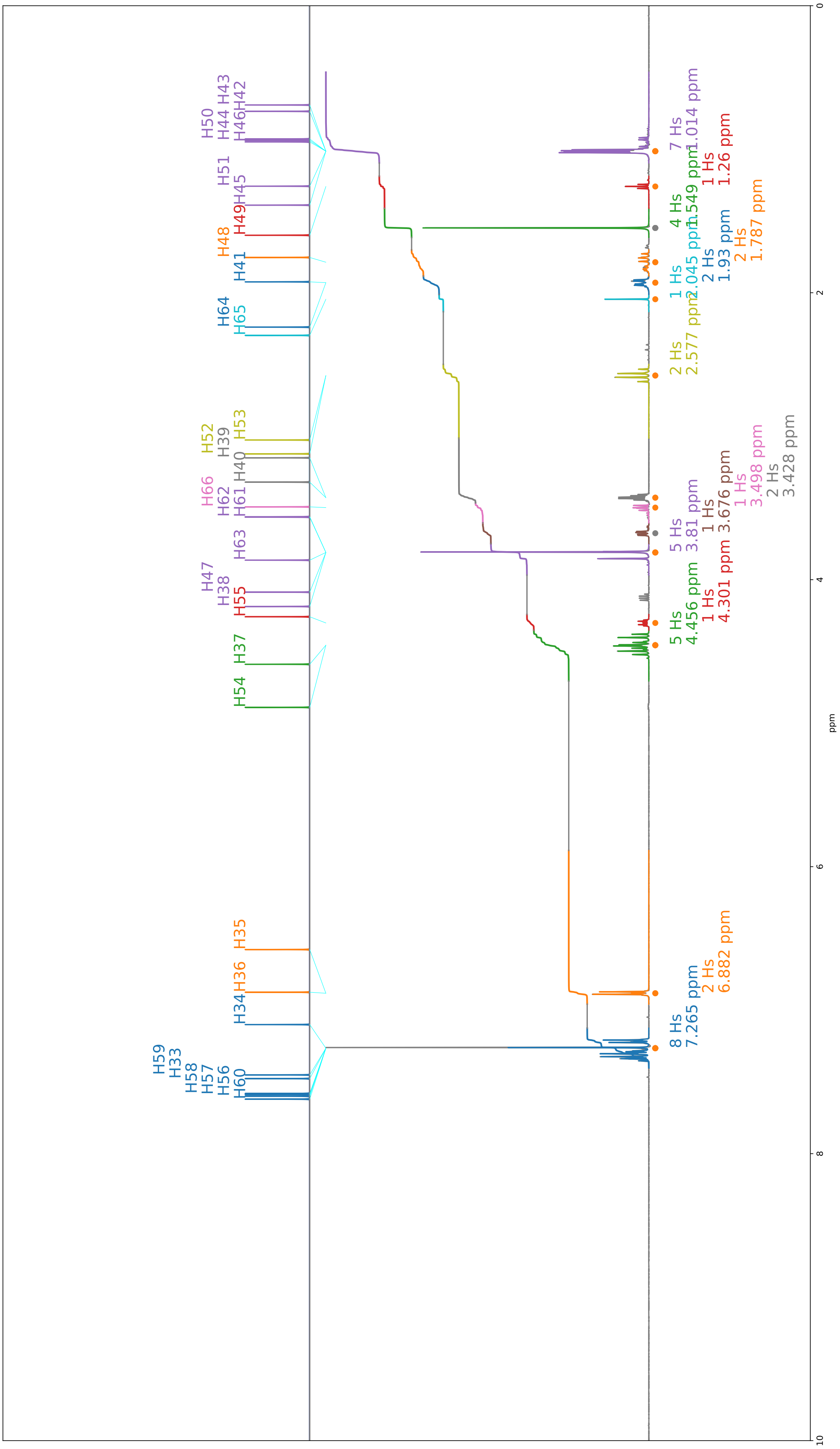

Figure 148: TS3A Proton Spectra

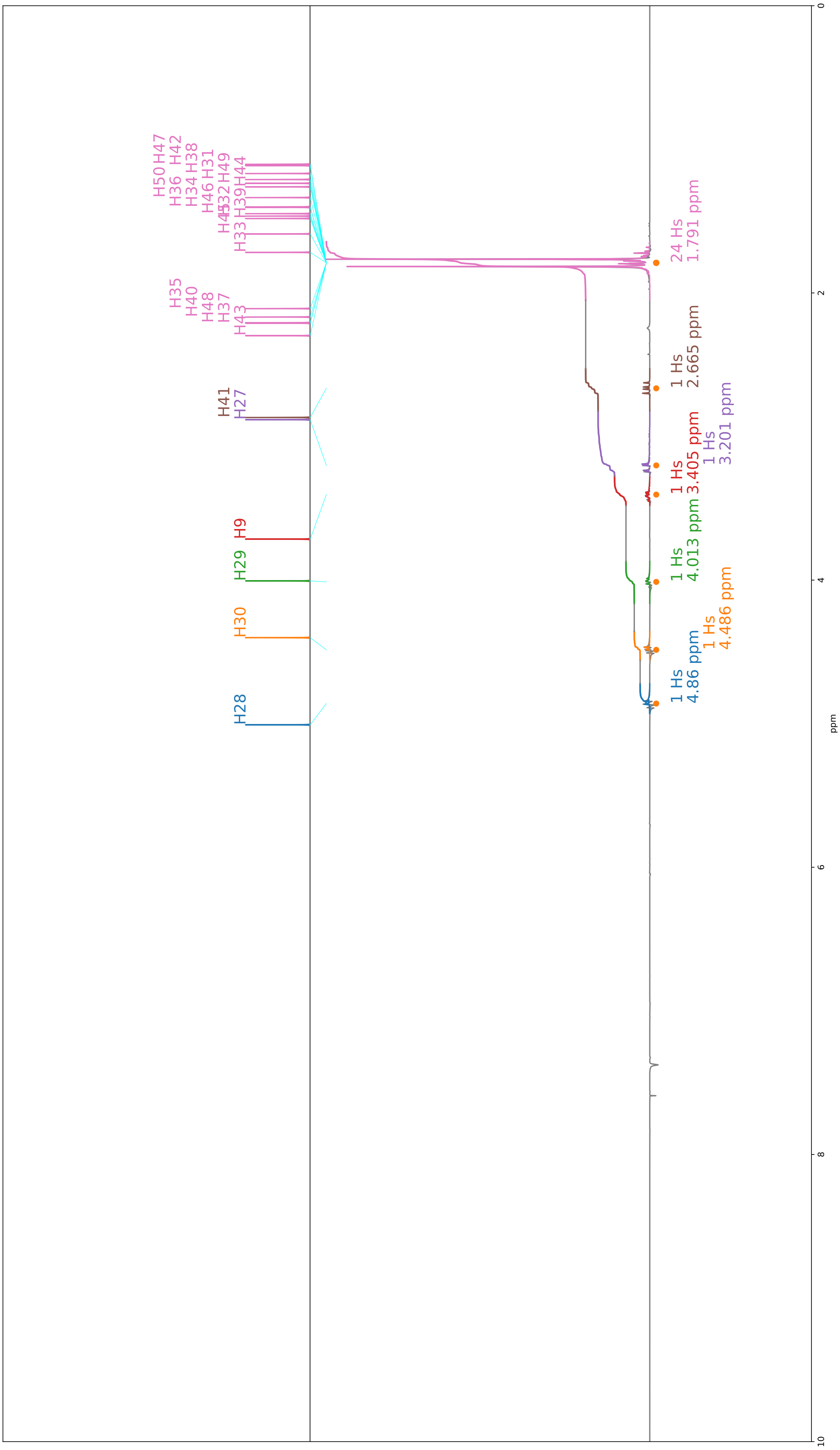

Figure 149: JB3 Proton Spectra

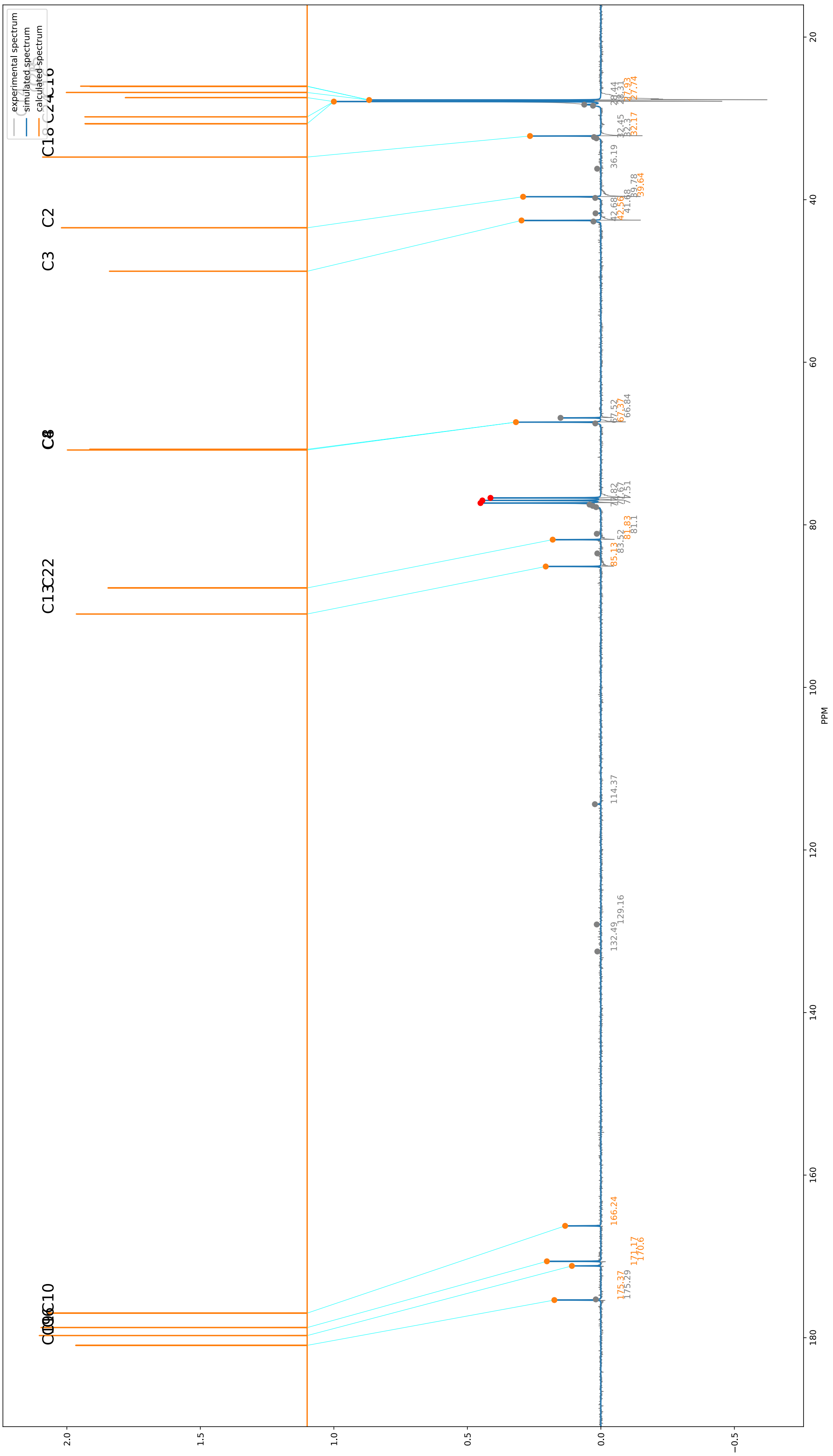

Figure 150: JB3 Carbon Spectra

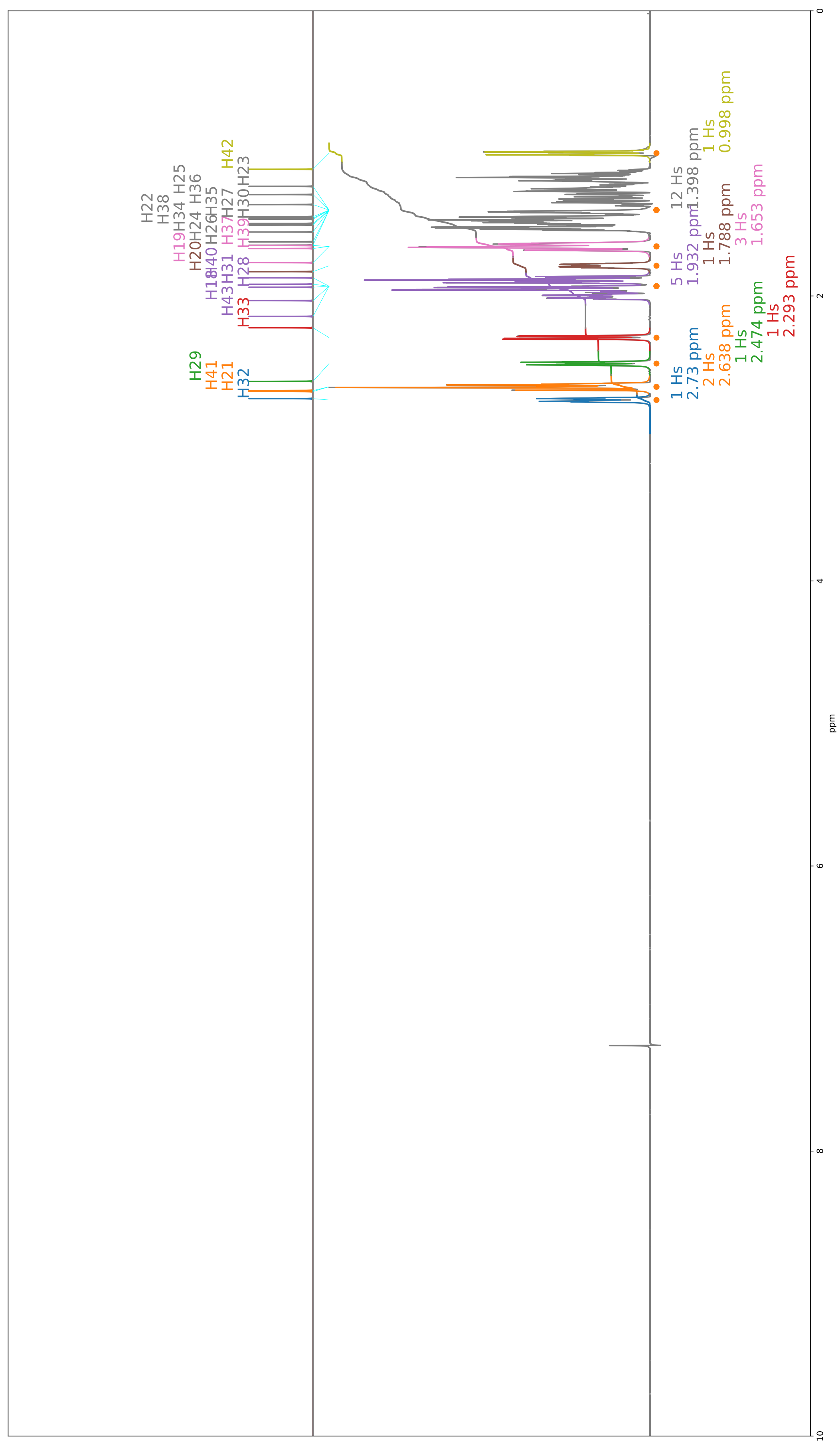

Figure 151: NP5 Proton Spectra

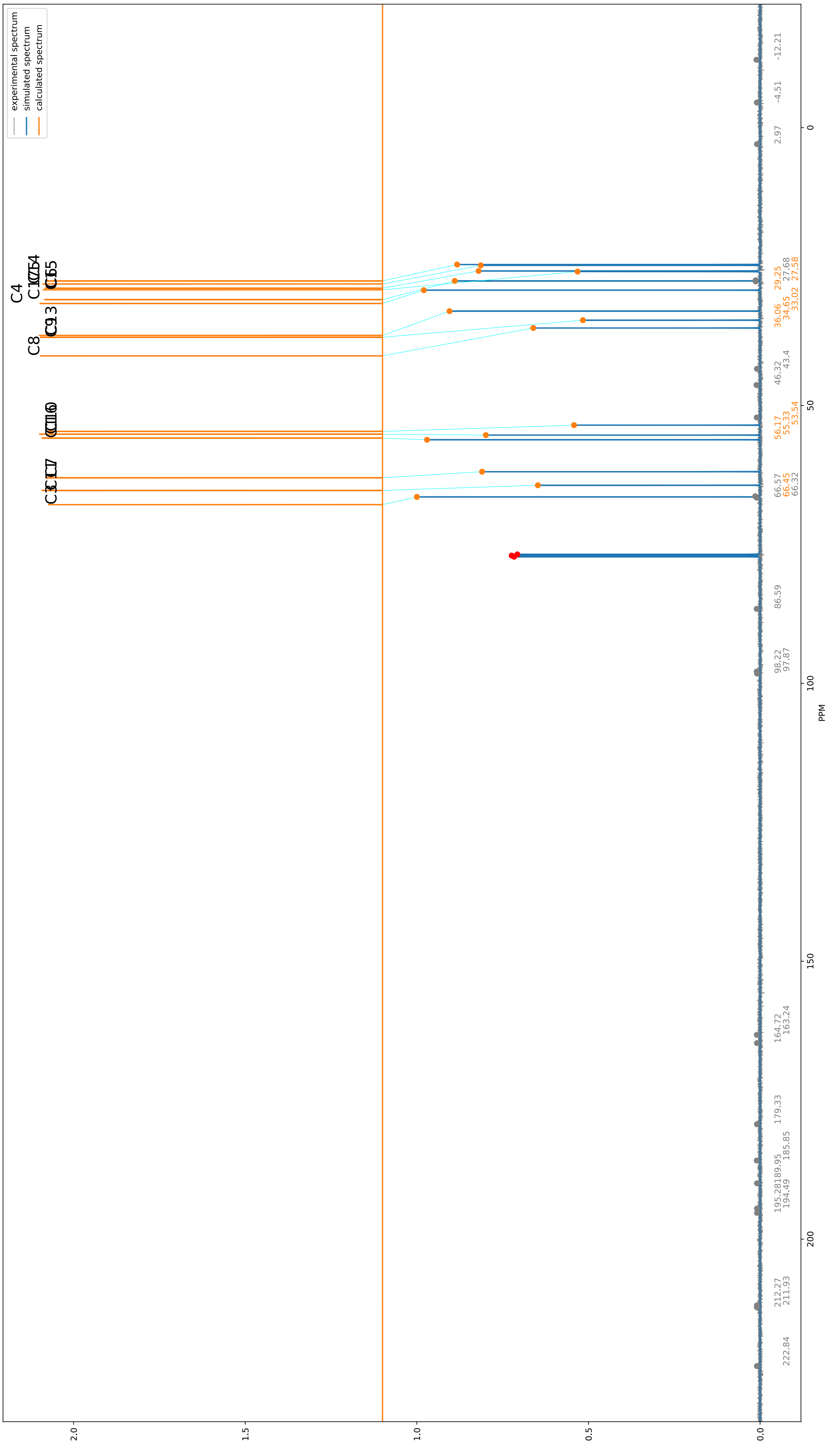

Figure 152: NP5 Carbon Spectra

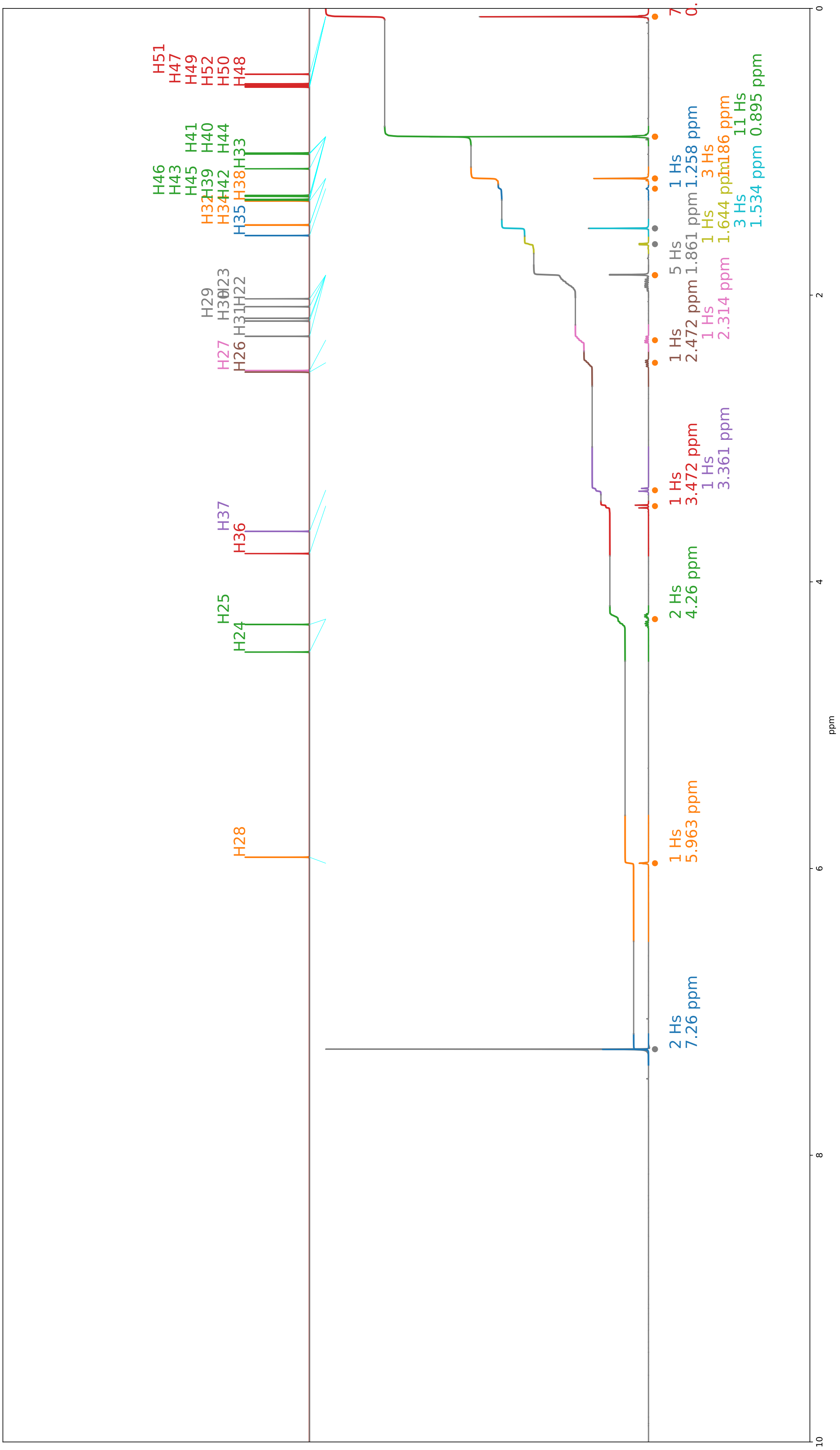

Figure 153: NL1A Proton Spectra

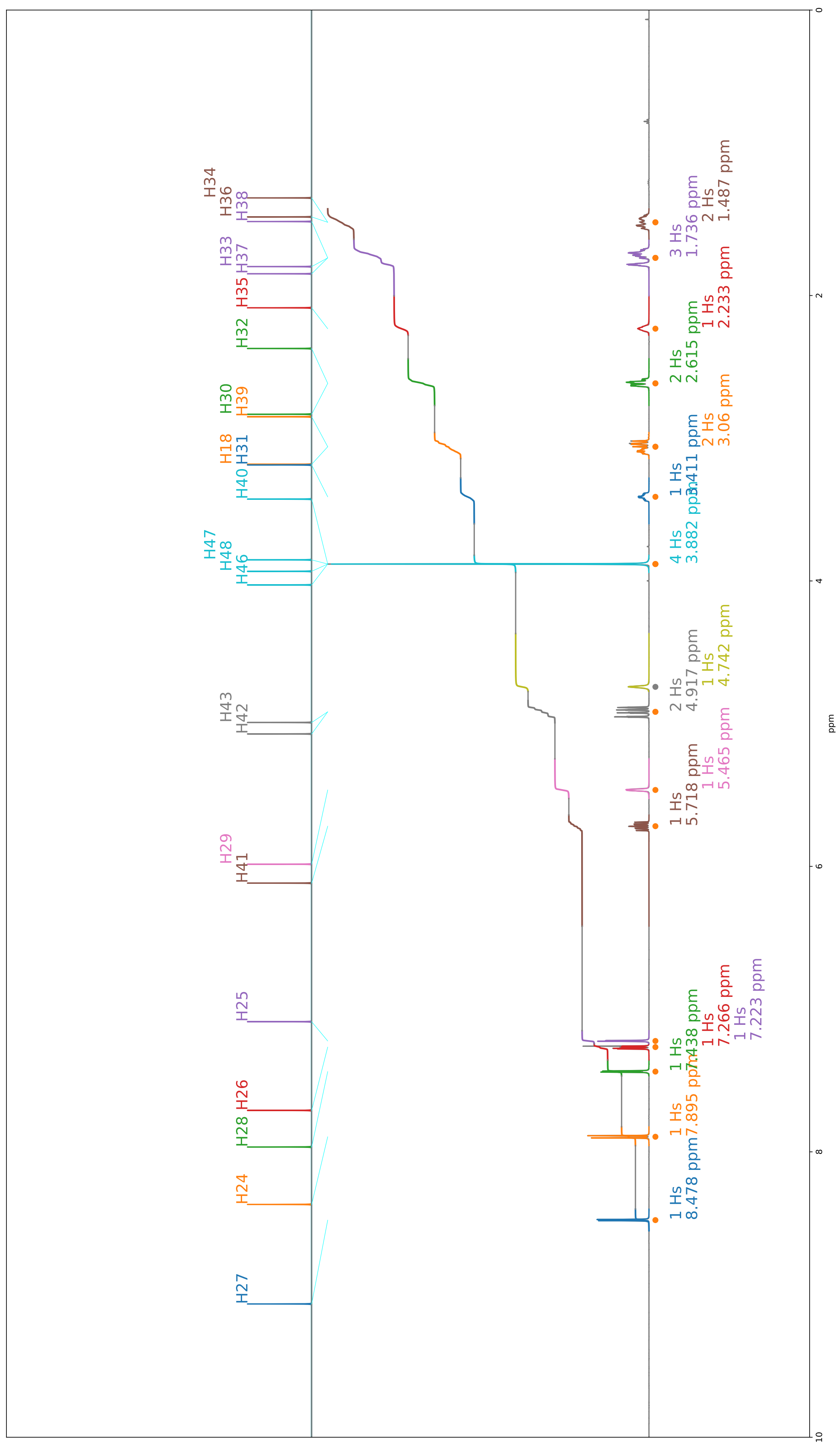

Figure 154: NP3A Proton Spectra

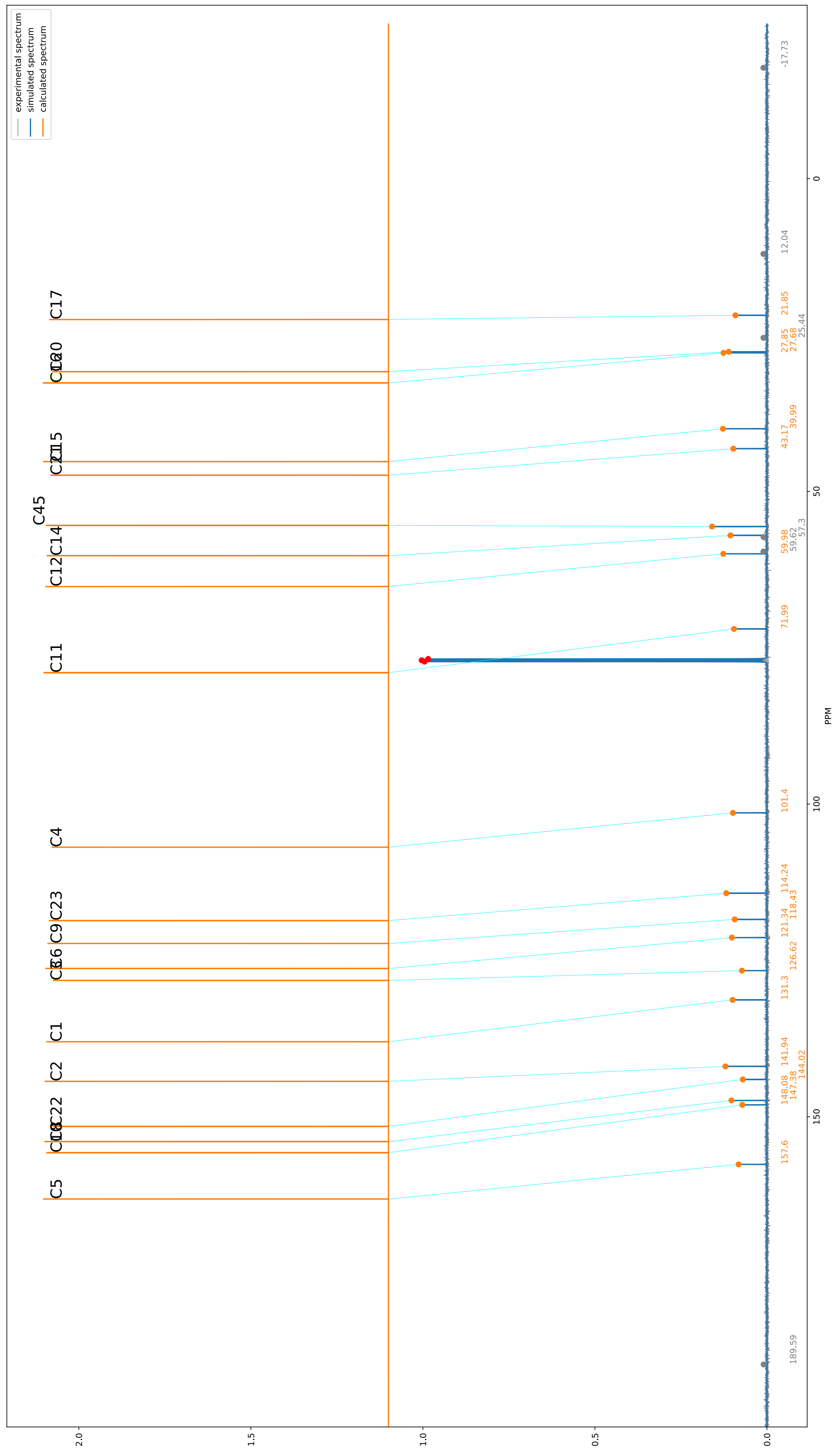

Figure 155: NP3A Carbon Spectra

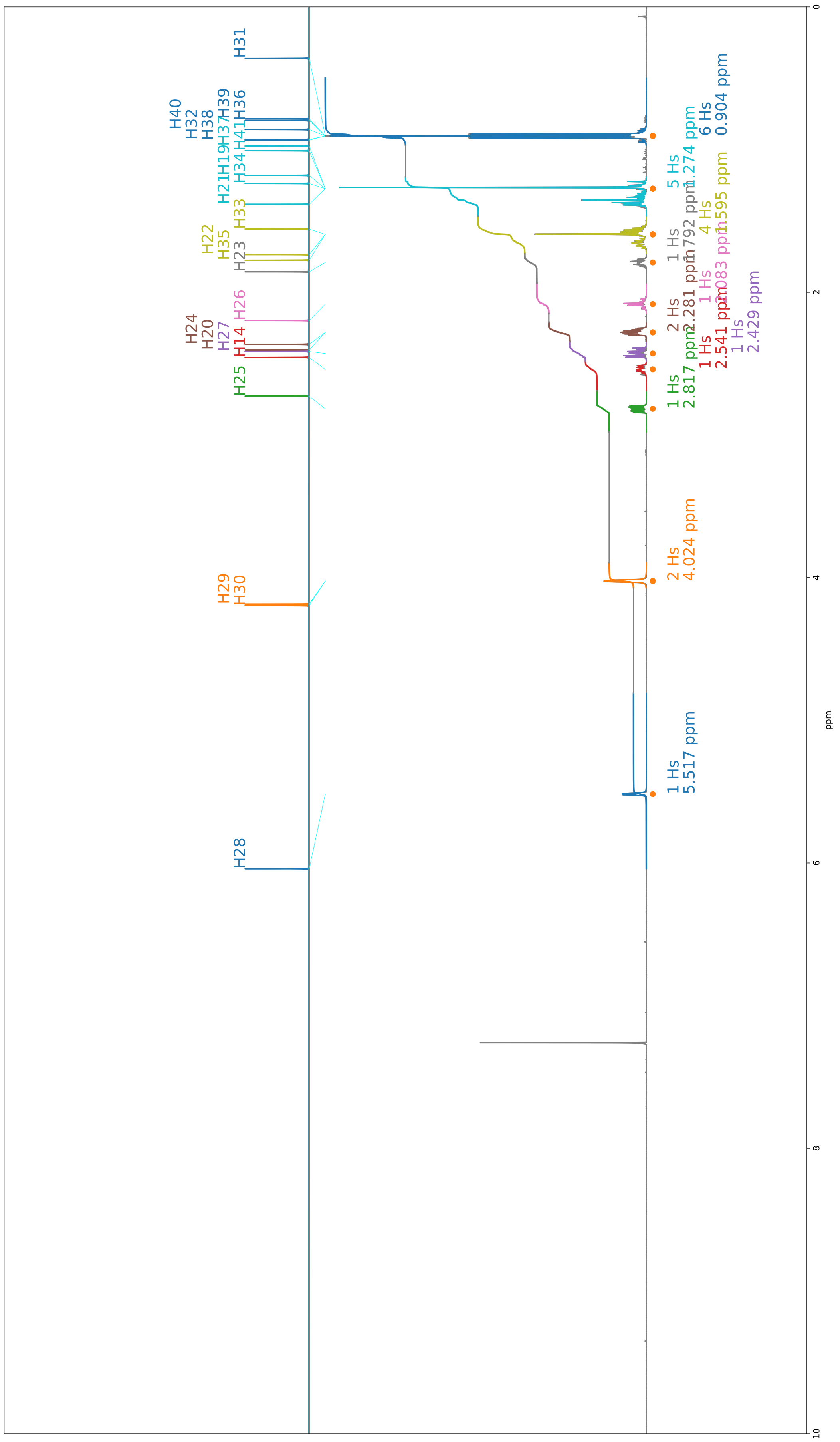

Figure 156: JB9 Proton Spectra

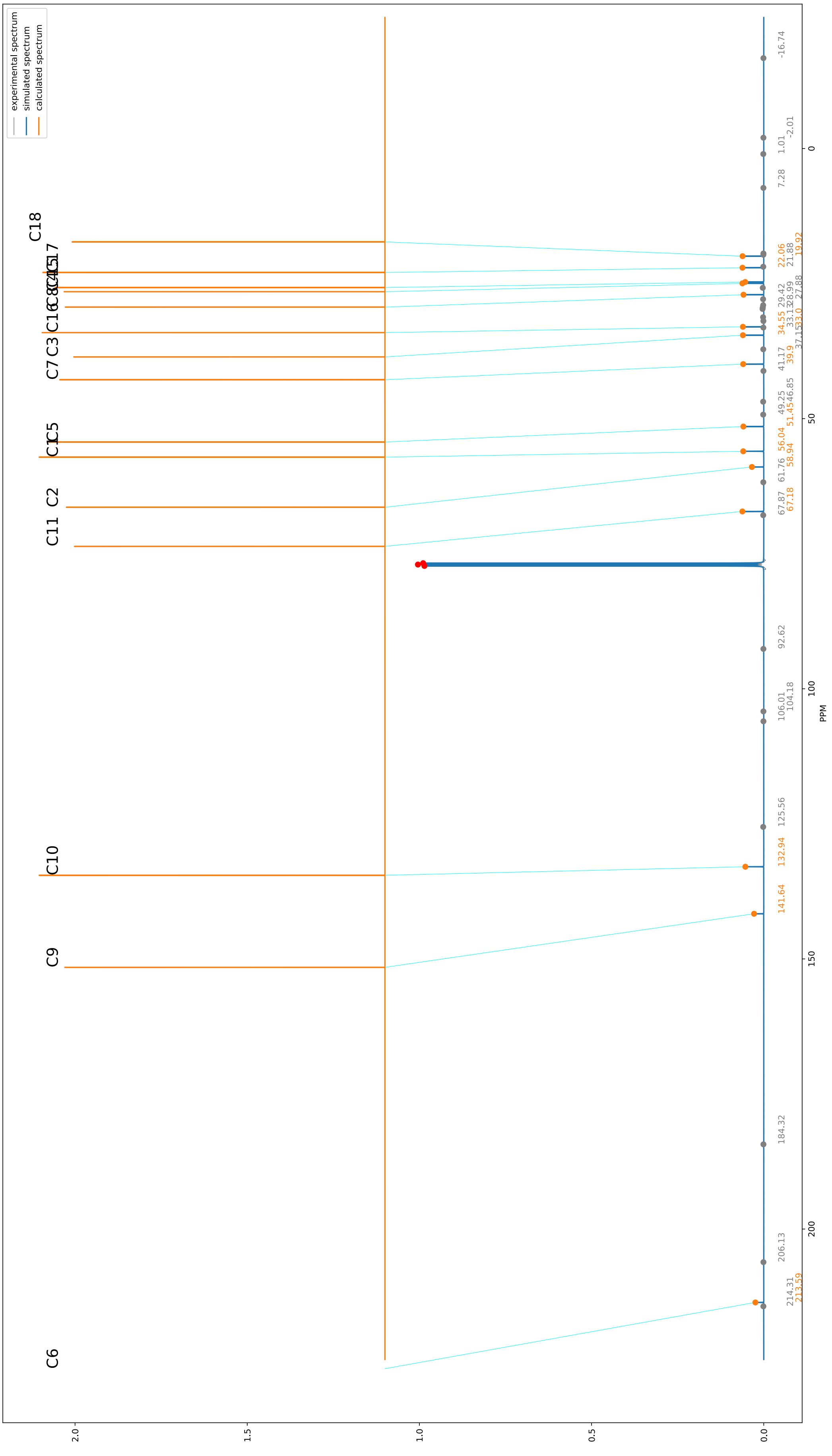

Figure 157: JB9 Carbon Spectra

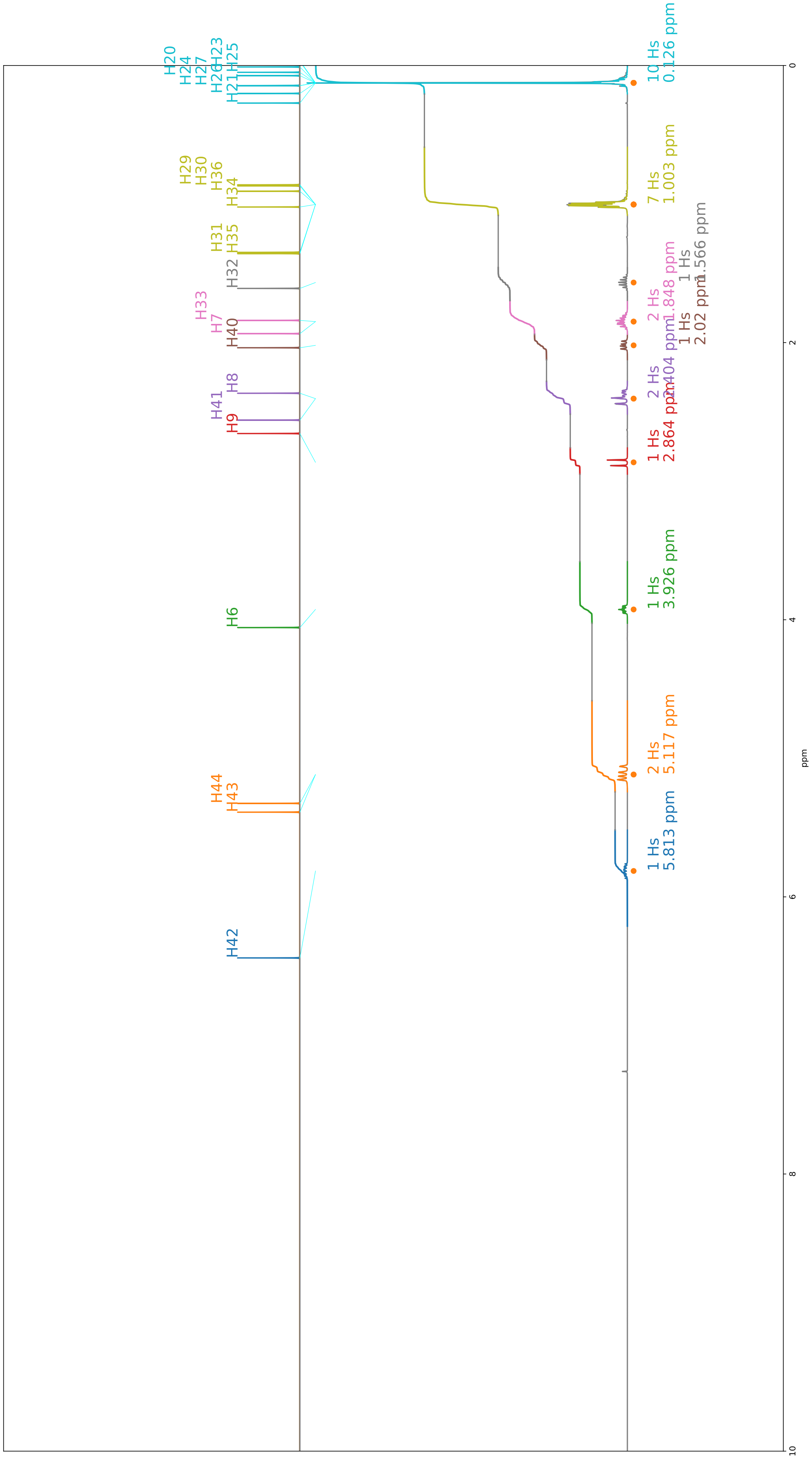

Figure 158: IP1 Proton Spectra

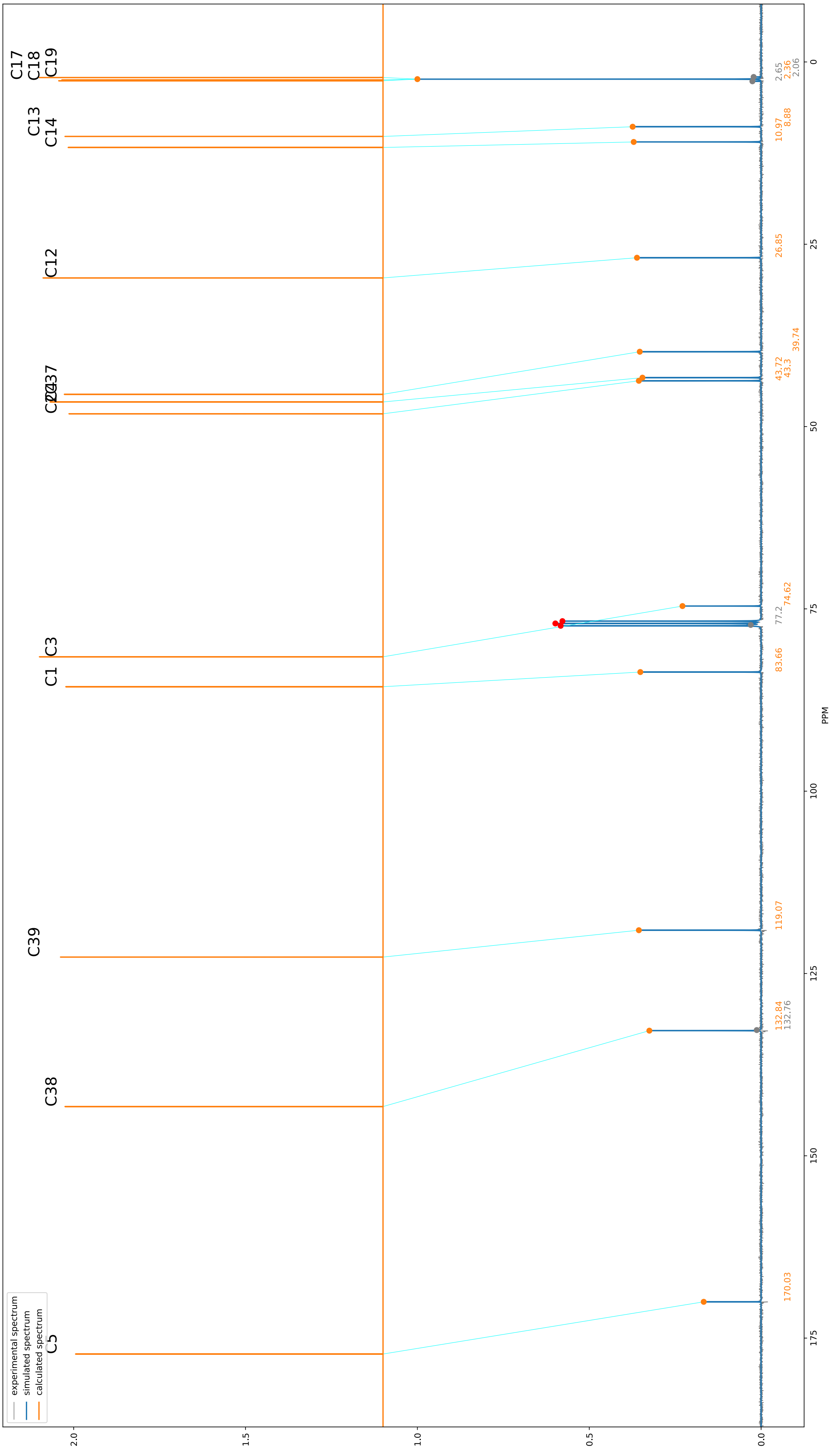

Figure 159: IP1 Carbon Spectra

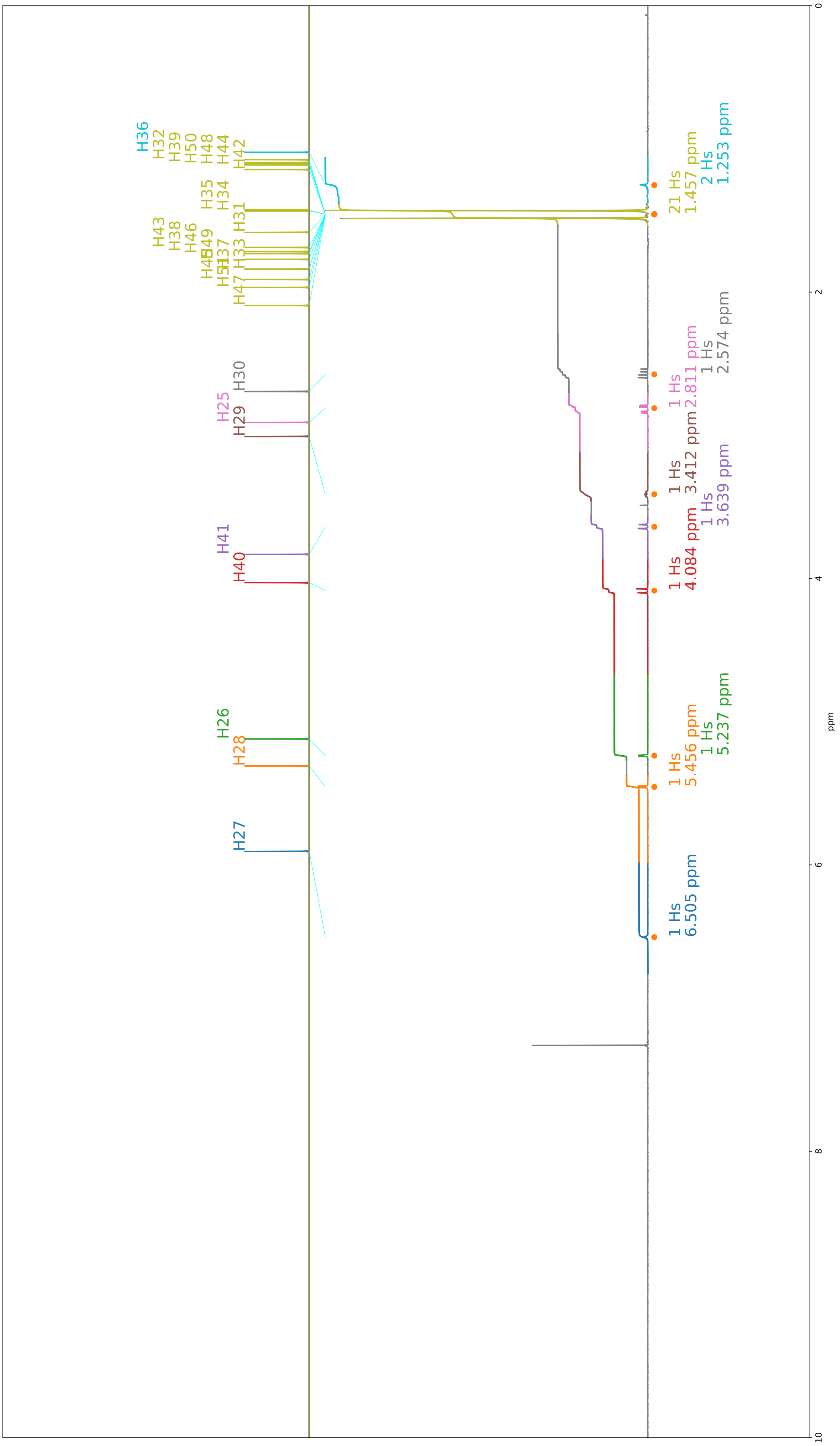

Figure 160: JB4 Proton Spectra

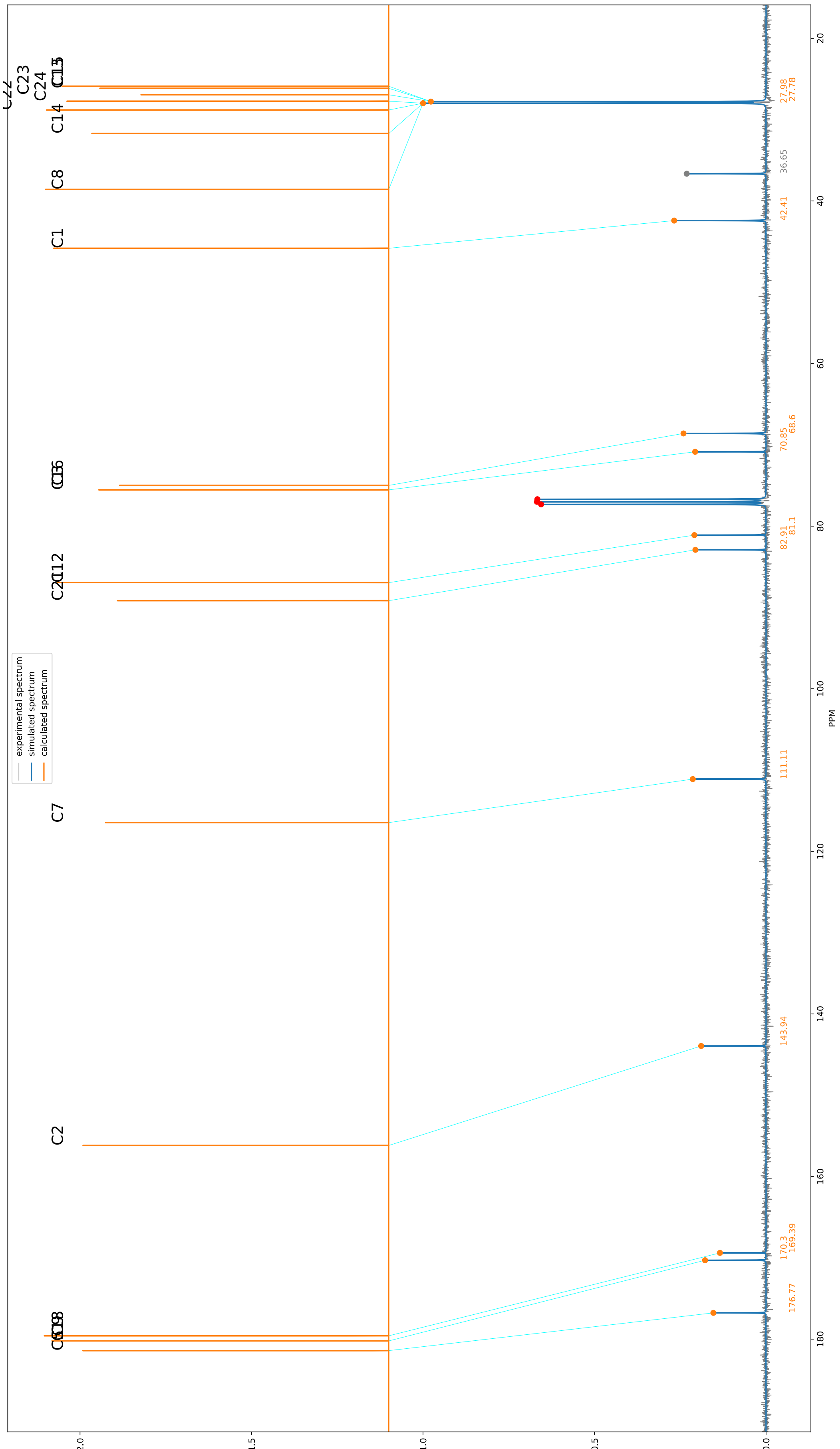

Figure 161: JB4 Carbon Spectra

5 Full Carbon NMR Shift Predictions, Assignments and DP4 Probabilities DP4-AI

Below are tables containing the DFT calculated shifts for each atom in each molecule at the three levels of theory tested and the corresponding experimental shift values assigned by DP4-AI.

5.1 AT1

	MM calc exp	Opt calc exp	Opt E calc exp
C1	61.01 61.74	65.54 61.74	65.28 61.74
C10	22.12 20.35	21.01 20.35	21.04 20.35
C12	16.24 13.64	15.60 13.64	15.64 13.64
C13	29.31 25.18	30.19 25.18	30.04 25.18
C14	12.51 9.60	12.00 9.60	11.98 9.60
C2	178.23 170.87	175.95 170.87	175.92 170.87
C3	54.49 49.49	54.54 49.49	54.57 49.49
C7	49.06 47.66	49.65 47.66	49.30 47.66
C8	30.14 27.60	32.51 27.60	32.42 27.60
C9	21.76 20.20	20.54 20.20	20.51 20.20

	MM calc exp	Opt calc exp	Opt E calc exp
H15	1.44 1.45	1.43 1.45	1.44 1.45
H16	1.12 0.93	1.06 0.93	1.06 0.93
H17	1.31 1.30	1.26 1.30	1.27 1.30
H18	1.11 0.93	1.10 0.93	1.10 0.93
H19	1.00 0.93	0.96 0.93	0.97 0.93
H20	0.90 0.93	0.90 0.93	0.90 0.93
H21	1.97 1.85	1.96 1.85	1.97 1.85
H22	1.40 1.30	1.39 1.30	1.38 1.30
H23	2.76 2.74	3.03 3.12	3.00 3.12
H24	2.98 3.12	2.86 2.74	2.92 2.74
H25	1.86 1.85	1.89 1.85	1.87 1.85
H26	0.77 0.93	0.71 0.93	0.74 0.93
H27	0.96 0.93	0.94 0.93	0.93 0.93
H28	1.07 0.93	1.15 1.30	1.13 1.30
H29	1.21 1.30	1.11 0.93	1.11 0.93
H30	0.90 0.93	0.92 0.93	0.90 0.93
H31	0.83 0.93	0.77 0.93	0.81 0.93
H4	3.04 3.12	3.14 3.12	3.15 3.12
H5	2.73 2.74	2.65 2.74	2.65 2.74

Figure 162: Table displaying the DFT NMR shift predictions (PPM) for molecule AT1 at the three levels of theory tested and the corresponding experimental values (PPM) assigned by DP4-AI

5.2 AT2

	MM	Opt	Opt E
	calc exp	calc exp	calc exp
C1	177.89 170.63	176.64 170.63	176.68 170.63
C17	53.58 51.13	53.34 51.13	53.50 51.13
C18	36.72 27.94	38.73 27.94	38.69 27.94
C19	28.27 27.94	28.01 26.20	28.03 26.20
C2	26.32 22.53	26.73 22.53	26.73 22.53
C20	29.59 27.94	28.70 27.94	28.69 27.94
C21	28.93 27.94	27.63 24.98	27.66 24.98
C3	28.05 26.20	28.59 27.94	28.59 27.94
C4	58.67 54.95	59.71 54.95	59.66 54.95
C5	59.77 59.73	64.92 59.73	64.93 59.73
C6	27.74 24.98	29.46 27.94	29.55 27.94

	MM	Opt	Opt E
	calc exp	calc exp	calc exp
H10	1.91 1.98	1.93 1.98	1.93 1.98
H11	3.37 3.49	3.32 3.49	3.31 3.49
H12	3.77 4.07	4.01 4.07	4.01 4.07
H13	1.43 1.47	1.33 1.47	1.33 1.47
H14	1.95 1.98	2.00 1.98	2.00 1.98
H22	3.12 3.21	3.27 3.21	3.28 3.21
H23	2.33 2.42	2.43 2.42	2.43 2.42
H24	0.85 0.94	0.84 0.94	0.84 0.94
H25	1.06 0.94	0.95 0.94	0.95 0.94
H26	1.20 0.94	1.19 0.94	1.19 0.94
H27	0.94 0.94	0.94 0.94	0.94 0.94
H28	0.84 0.94	0.87 0.94	0.88 0.94
H29	0.99 0.94	0.94 0.94	0.95 0.94
H30	1.26 0.94	1.17 0.94	1.16 0.94
H31	0.82 0.94	0.88 0.94	0.88 0.94
H32	0.87 0.94	0.89 0.94	0.89 0.94
H7	1.75 1.80	1.75 1.80	1.75 1.80
H8	1.56 1.47	1.68 1.47	1.69 1.47
H9	1.46 1.47	1.48 1.47	1.48 1.47

Figure 163: Table displaying the DFT NMR shift predictions (PPM) for molecule AT2 at the three levels of theory tested and the corresponding experimental values (PPM) assigned by DP4-AI

5.3 AT3

	MM		Opt		Opt E	
	calc	exp	calc	exp	calc	exp
H15	7.62	7.22	7.52	7.22	7.52	7.22
H16	6.94	6.87	7.01	6.87	7.00	6.87
H17	6.97	6.87	7.02	6.87	7.04	6.87
H20	0.85	0.85	0.88	0.85	0.80	0.85
H21	1.00	0.85	0.96	0.85	0.94	0.85
H22	0.79	0.85	0.82	0.85	0.78	0.85
H23	1.59	1.50	1.59	1.70	1.62	1.70
H24	1.92	1.70	1.78	1.70	1.76	1.70
H25	1.68	1.70	1.58	1.50	1.59	1.50
H26	3.61	3.49	3.54	3.49	3.45	3.49
H27	3.71	3.72	3.83	3.72	3.82	3.72
H28	4.19	3.93	4.35	3.93	4.24	3.93
H29	3.67	3.72	3.83	3.72	3.91	3.72
H30	3.86	3.72	3.93	3.72	3.89	3.72
H31	2.63	2.51	2.58	2.51	2.53	2.51
H33	0.84	0.85	0.98	0.85	0.86	0.85
H34	0.95	0.85	1.02	0.85	0.98	0.85
H35	0.78	0.85	0.93	0.85	1.01	0.85
H36	1.35	1.50	1.25	1.50	1.38	1.50

Figure 164: Table displaying the DFT NMR shift predictions (PPM) for molecule AT3 at the three levels of theory tested and the corresponding experimental values (PPM) assigned by DP4-AI

5.4 BYH1

	MM		Opt		Opt E	
	calc	exp	calc	exp	calc	exp
C1	162.51	159.29	158.75	159.29	158.42	159.29
C11	41.00	40.22	40.58	40.22	41.50	40.22
C12	70.25	72.86	70.90	72.43	67.92	72.43
C14	14.34	15.51	15.05	15.51	15.31	15.51
C16	69.88	72.43	71.02	72.86	70.63	72.86
C17	153.61	141.01	155.73	141.01	155.71	141.01
C18	110.10	95.85	108.96	95.85	108.90	95.85
C19	128.10	129.35	129.14	129.35	129.34	129.35
C2	115.48	113.86	114.53	113.86	115.56	113.86
C20	128.82	129.35	126.75	113.86	126.74	113.86
C21	127.73	115.45	128.86	115.45	129.14	115.45
C22	111.98	113.86	112.80	95.85	112.87	95.85
C28	39.68	39.67	40.38	39.67	40.39	39.67
C29	140.94	129.81	138.78	129.81	138.14	129.81
C3	164.76	165.03	166.58	165.03	165.05	165.03
C30	132.64	129.35	135.97	129.35	136.93	129.35
C32	15.55	16.01	16.05	16.01	16.58	16.01
C35	22.76	28.08	24.18	28.08	24.39	28.08
C37	50.37	55.27	50.88	55.27	50.98	55.27
C4	26.77	29.53	29.01	29.53	25.21	29.53
C5	79.02	79.86	77.03	79.86	77.45	79.86

	MM		Opt		Opt E	
	calc	exp	calc	exp	calc	exp
H10	5.82	6.03	5.90	5.87	5.94	6.03
H15	2.62	2.71	2.71	2.71	2.77	2.71
H23	7.05	6.94	7.05	6.94	7.06	6.94
H24	7.70	7.27	7.60	7.27	7.57	7.27
H25	7.65	7.27	7.79	7.27	7.85	7.27
H26	7.15	6.94	7.25	6.94	7.31	6.94
H31	3.80	3.86	4.02	3.86	4.06	3.86
H33	2.72	2.71	2.82	2.71	2.95	2.71
H34	5.72	5.87	5.91	6.03	5.80	5.87
H38	3.75	3.86	4.06	3.86	4.18	3.86
H39	3.89	3.86	3.87	3.86	3.86	3.86
H40	3.72	3.86	4.05	3.86	3.98	3.86
H41	4.34	4.47	4.46	4.47	4.34	4.11
H42	4.26	4.11	4.60	4.47	4.79	4.47
H43	3.35	3.48	3.40	3.48	3.37	3.48
H44	3.40	3.48	3.55	3.48	3.39	3.48
H45	1.47	1.15	1.31	1.15	1.30	1.15
H46	1.20	1.15	1.25	1.15	1.20	1.15
H47	1.03	1.15	1.63	1.15	1.40	1.15
H48	1.42	1.15	1.83	1.15	1.74	1.15
H49	1.05	1.15	1.15	1.15	1.21	1.15
H50	0.95	1.15	1.17	1.15	1.08	1.15
H51	2.02	2.45	2.50	2.45	2.53	2.45
H52	1.92	2.45	2.23	2.28	2.37	2.45
H53	1.61	2.28	2.36	2.45	2.40	2.45
H6	1.88	2.28	2.26	2.28	2.17	2.28
H7	2.12	2.45	2.38	2.45	2.10	2.28

Figure 165: Table displaying the DFT NMR shift predictions (PPM) for molecule BYH1 at the three levels of theory tested and the corresponding experimental values (PPM) assigned by DP4-AI

5.5 BYH2

	MM		Opt		Opt E	
	calc	exp	calc	exp	calc	exp
C1	73.16	73.02	71.48	73.02	72.07	71.85
C10	16.48	16.09	16.62	16.09	16.76	16.09
C11	143.09	129.76	141.64	143.07	141.41	143.07
C12	130.36	129.32	134.37	129.76	134.91	129.76
C13	23.84	28.00	24.65	28.00	25.31	28.00
C15	154.19	143.07	155.72	159.28	156.08	159.28
C16	111.45	113.84	110.91	113.84	111.23	113.84
C17	128.28	129.32	128.80	129.32	129.74	129.32
C18	127.18	113.84	126.22	129.32	125.48	113.84
C19	128.47	129.32	128.28	129.32	129.12	129.32
C2	44.86	46.59	45.78	46.59	46.01	46.59
C20	111.07	113.84	110.82	113.84	110.84	113.84
C26	50.24	55.26	50.76	55.26	50.78	55.26
C3	12.42	13.17	12.84	13.17	13.72	13.17
C4	225.58	214.51	219.91	214.51	218.47	214.51
C49	70.41	70.70	70.82	70.70	71.32	70.70
C6	47.50	46.70	47.18	46.70	46.87	46.70
C7	72.38	71.85	71.15	71.85	72.64	73.02
C9	38.86	40.93	40.95	40.93	40.47	40.93

	MM		Opt		Opt E	
	calc	exp	calc	exp	calc	exp
H21	7.11	6.94	7.12	6.94	7.15	6.94
H22	7.81	7.27	7.73	7.27	7.74	7.27
H23	7.54	7.27	7.66	7.27	7.65	7.27
H24	6.99	6.94	7.10	6.94	7.10	6.94
H27	3.70	3.86	3.86	3.86	3.84	3.86
H28	3.73	3.86	4.02	3.86	3.89	3.86
H29	3.83	3.86	4.03	3.86	4.16	3.86
H30	3.62	3.86	3.51	3.51	3.52	3.51
H31	3.41	3.21	3.58	3.62	3.82	3.86
H32	1.03	1.09	1.24	1.09	1.35	1.09
H33	1.07	1.09	1.36	1.09	1.20	1.09
H34	0.88	1.09	1.01	1.09	1.12	1.09
H35	3.53	3.62	3.24	2.93	3.48	3.21
H36	2.35	2.45	3.28	3.21	3.22	2.93
H37	3.06	2.93	2.67	2.45	2.47	2.45
H38	3.86	3.86	3.88	3.86	3.60	3.86
H39	2.34	2.45	2.70	2.77	2.43	2.45
H40	0.88	1.09	0.99	1.09	0.68	1.09
H41	0.63	1.09	1.03	1.09	0.75	1.09
H42	0.51	1.09	1.72	1.09	1.04	1.09
H43	2.09	2.45	2.40	2.45	2.55	2.45
H44	1.90	2.45	2.56	2.45	2.50	2.45
H45	2.24	2.45	2.22	2.45	2.18	2.45
H46	5.72	6.01	5.91	6.01	5.75	6.01
H47	3.45	3.51	3.63	3.86	3.52	3.62
H50	4.26	4.47	4.61	4.47	4.59	4.47
H51	4.32	4.47	4.44	4.47	4.36	4.47

Figure 166: Table displaying the DFT NMR shift predictions (PPM) for molecule BYH2 at the three levels of theory tested and the corresponding experimental values (PPM) assigned by DP4-AI

5.6 IP1

	MM calc exp	Opt calc exp	Opt E calc exp
C1	85.17 83.66	87.25 83.66	85.68 83.66
C12	30.85 26.85	31.09 26.85	29.62 26.85
C13	11.54 8.88	11.66 8.88	10.22 8.88
C14	12.55 10.97	11.88 10.97	11.73 10.97
C17	3.16 2.36	2.33 2.36	2.46 2.36
C18	3.19 2.36	2.72 2.36	2.58 2.36
C19	2.44 2.36	2.11 2.36	2.13 2.36
C2	47.46 43.72	48.36 43.72	48.25 43.72
C3	83.40 74.62	80.99 74.62	81.57 74.62
C37	45.83 39.74	44.07 39.74	45.58 39.74
C38	144.80 132.84	143.08 132.84	143.25 132.84
C39	124.41 119.07	123.01 119.07	122.75 119.07
C4	46.49 43.30	46.38 43.30	46.63 43.30
C5	179.45 170.03	175.83 170.03	177.18 170.03

	MM calc exp	Opt calc exp	Opt E calc exp
H20	0.07 0.13	0.09 0.13	0.07 0.13
H21	0.16 0.13	0.26 0.13	0.27 0.13
H22	-0.12 0.13	-0.02 0.13	-0.03 0.13
H23	0.01 0.13	0.09 0.13	0.01 0.13
H24	0.11 0.13	0.16 0.13	0.15 0.13
H25	-0.03 0.13	-0.03 0.13	0.05 0.13
H26	0.16 0.13	0.21 0.13	0.20 0.13
H27	0.07 0.13	0.06 0.13	0.15 0.13
H28	-0.14 0.13	-0.01 0.13	-0.03 0.13
H29	0.86 1.00	0.88 1.00	0.86 1.00
H30	0.79 1.00	0.71 1.00	0.87 1.00
H31	1.55 1.00	1.55 1.00	1.35 1.00
H32	1.65 1.57	1.55 1.57	1.61 1.57
H33	1.95 1.85	1.81 1.85	1.84 1.85
H34	1.08 1.00	1.08 1.00	1.02 1.00
H35	1.39 1.00	1.47 1.00	1.36 1.00
H36	0.91 1.00	0.92 1.00	0.91 1.00
H40	2.15 2.02	2.04 2.02	2.04 2.02
H41	2.61 2.40	2.54 2.40	2.56 2.40
H42	6.50 5.81	6.42 5.81	6.44 5.81
H43	5.41 5.12	5.39 5.12	5.39 5.12
H44	5.35 5.12	5.33 5.12	5.33 5.12
H6	4.04 3.93	4.03 3.93	4.06 3.93
H7	2.11 1.85	1.90 1.85	1.94 1.85
H8	2.50 2.40	2.34 2.40	2.37 2.40
H9	2.89 2.86	2.74 2.86	2.66 2.86

Figure 167: Table displaying the DFT NMR shift predictions (PPM) for molecule IP1 at the three levels of theory tested and the corresponding experimental values (PPM) assigned by DP4-AI

5.7 IP2

	MM calc exp	Opt calc exp	Opt E calc exp
C1	132.06 127.55	130.68 126.35	130.74 126.35
C12	82.27 76.50	80.30 76.50	80.44 76.50
C13	69.34 58.21	70.17 58.21	69.30 58.21
C14	16.46 14.38	14.54 14.38	14.73 14.38
C16	185.28 176.08	182.98 176.08	183.49 176.08
C17	31.20 27.50	31.95 27.50	32.22 27.50
C18	11.31 9.13	11.16 9.13	11.46 9.13
C2	152.13 142.43	151.49 142.43	151.50 142.43
C3	131.76 126.35	130.32 126.35	130.70 126.35
C32	38.35 27.50	39.37 32.56	38.84 32.56
C4	132.40 128.27	132.38 128.27	132.48 128.27
C5	130.99 126.35	131.41 127.55	131.52 127.55
C6	133.21 128.27	133.17 128.27	133.13 128.27

	MM calc exp	Opt calc exp	Opt E calc exp
H10	7.43 7.26	7.44 7.26	7.45 7.26
H11	7.61 7.26	7.58 7.26	7.58 7.26
H21	5.07 4.49	4.93 4.49	4.90 4.49
H22	5.95 4.49	5.56 4.49	5.43 4.49
H23	3.52 2.83	3.46 2.83	3.54 2.83
H24	1.23 1.02	1.14 1.02	1.12 1.02
H25	2.31 2.72	2.03 2.22	1.94 1.02
H26	1.17 1.02	1.08 1.02	1.02 1.02
H27	2.09 2.22	2.00 1.02	2.08 2.22
H28	2.20 2.22	2.09 2.22	2.12 2.22
H29	1.11 1.02	1.01 1.02	1.00 1.02
H30	0.90 1.02	0.81 1.02	0.82 1.02
H31	1.19 1.02	1.15 1.02	1.17 1.02
H33	2.07 1.02	2.23 2.72	2.39 2.72
H34	2.65 2.72	2.56 2.72	2.53 2.72
H35	2.43 2.72	2.61 2.72	2.60 2.72
H7	7.64 7.26	7.59 7.26	7.57 7.26
H8	7.42 7.26	7.37 7.26	7.39 7.26
H9	7.53 7.26	7.52 7.26	7.53 7.26

Figure 168: Table displaying the DFT NMR shift predictions (PPM) for molecule IP2 at the three levels of theory tested and the corresponding experimental values (PPM) assigned by DP4-AI

5.8 IP3

	MM	Opt	Opt E
	calc exp	calc exp	calc exp
C1	186.60 170.02	180.66 170.02	180.65 170.02
C11	123.25 111.80	130.77 123.40	130.78 123.40
C12	40.15 36.50	42.06 36.50	41.81 36.50
C13	23.83 23.44	28.25 23.44	27.88 23.44
C14	23.87 23.44	28.55 23.44	28.21 23.44
C15	39.77 36.50	42.77 36.50	42.56 36.50
C17	53.86 52.75	54.95 52.75	54.93 52.75
C3	53.71 52.75	55.15 52.75	55.16 52.75
C4	81.41 76.78	82.54 76.78	82.59 76.78
C5	81.87 76.78	81.72 76.78	81.71 76.78
C6	186.38 170.02	180.26 170.02	180.23 170.02

	MM	Opt	Opt E
	calc exp	calc exp	calc exp
H18	3.74 3.81	3.74 3.81	3.74 3.81
H19	3.77 3.81	3.80 3.81	3.80 3.81
H20	3.80 3.81	3.81 3.81	3.81 3.81
H21	4.81 4.76	4.70 4.76	4.68 4.76
H22	3.77 3.81	3.78 3.81	3.77 3.81
H23	3.76 3.81	3.73 3.81	3.73 3.81
H24	3.79 3.81	3.77 3.81	3.77 3.81
H25	2.06 1.91	2.30 1.91	2.29 1.91
H26	1.86 1.71	1.81 1.91	1.80 1.91
H27	1.82 1.71	1.70 1.71	1.71 1.71
H28	1.80 1.71	1.76 1.71	1.76 1.71
H29	1.77 1.71	1.74 1.71	1.74 1.71
H30	1.87 1.91	1.72 1.71	1.73 1.71
H31	1.97 1.91	1.83 1.91	1.84 1.91
H32	2.06 1.91	2.42 1.91	2.41 1.91
H33	4.97 4.76	4.86 4.76	4.86 4.76

Figure 169: Table displaying the DFT NMR shift predictions (PPM) for molecule IP3 at the three levels of theory tested and the corresponding experimental values (PPM) assigned by DP4-AI

5.9 IP4

	MM		Opt		Opt E	
	calc	exp	calc	exp	calc	exp
C1	136.46	125.26	136.21	125.26	136.04	125.26
C12	18.95	16.23	14.45	16.23	19.32	16.23
C2	43.82	39.34	47.18	39.34	45.34	39.34
C3	81.42	75.43	78.87	75.43	81.88	75.43
C4	53.77	47.15	50.17	47.15	56.11	47.15
C5	143.38	133.08	143.08	133.08	142.92	133.08

	MM		Opt		Opt E	
	calc	exp	calc	exp	calc	exp
H10	5.76	5.54	5.92	5.54	5.87	5.54
H11	5.88	5.54	5.81	5.54	5.96	5.54
H13	1.15	0.95	1.15	0.95	0.62	0.95
H14	1.28	1.66	1.24	1.66	1.15	0.95
H15	0.60	0.95	1.05	0.95	1.29	1.66
H17	0.86	0.95	0.51	0.95	0.74	0.95
H6	2.75	2.55	2.79	2.55	2.83	2.55
H7	2.29	2.18	2.37	2.18	2.29	2.18
H8	4.05	3.91	4.12	3.91	4.04	3.91
H9	2.61	2.55	2.85	2.55	2.68	2.55

Figure 170: Table displaying the DFT NMR shift predictions (PPM) for molecule IP4 at the three levels of theory tested and the corresponding experimental values (PPM) assigned by DP4-AI

5.10 IP5

	MM	Opt	Opt E
	calc exp	calc exp	calc exp
C1	134.28 128.30	134.75 128.30	135.04 128.30
C12	72.83 72.34	74.06 72.34	74.36 72.34
C14	80.43 73.91	82.51 73.91	82.58 73.91
C15		235.63 217.68	232.79 217.68
C16	46.79 45.13	46.76 45.13	47.14 45.13
C17	77.26 73.28	77.79 73.28	78.01 73.28
C18	154.71 143.73	153.16 143.73	154.18 143.73
C19	118.63 111.62	117.10 111.62	116.69 111.62
C2	133.20 127.49	133.35 127.49	133.63 127.49
C20	23.42 19.34	22.34 19.34	21.98 19.34
C23	19.10 13.67	18.00 13.67	18.42 13.67
C24	12.46 8.85	11.92 8.85	12.15 8.85
C3	132.77 127.49	133.70 127.49	133.75 127.49
C4	133.87 127.61	133.93 127.61	133.87 127.61
C5	134.06 128.30	134.61 128.30	134.73 128.30
C6	145.45 128.30	144.17 128.30	144.29 128.30

	MM	Opt	Opt E
	calc exp	calc exp	calc exp
H10	7.67 7.27	7.66 7.27	7.64 7.27
H11	7.82 7.27	7.75 7.27	7.68 7.27
H25	4.76 4.44	4.62 4.44	4.65 4.44
H26	4.44 4.44	4.61 4.44	4.63 4.44
H27	3.94 3.63	3.96 3.63	4.02 3.63
H28	1.20 1.04	1.32 1.04	1.36 1.04
H29	1.29 1.04	1.25 1.04	1.24 1.04
H30	1.41 1.04	1.48 1.04	1.44 1.04
H31	0.82 1.04	0.89 1.04	0.82 1.04
H32	1.04 1.04	1.02 1.04	1.01 1.04
H33	1.14 1.04	1.02 1.04	0.92 1.04
H34	4.46 4.44	4.42 4.44	4.23 4.44
H35	3.20 3.01	3.53 3.43	2.71 3.01
H36	5.29 5.05	5.20 4.91	5.13 4.91
H37	5.18 4.91	5.33 5.05	5.20 5.05
H38	1.53 1.67	1.67 1.67	1.65 1.67
H39	1.64 1.67	1.72 1.67	1.63 1.67
H40	1.78 1.67	1.68 1.67	1.68 1.67
H41	2.71 3.01	3.04 3.01	2.96 3.01
H7	7.41 7.27	7.46 7.27	7.52 7.27
H8	7.55 7.27	7.59 7.27	7.60 7.27
H9	7.52 7.27	7.60 7.27	7.59 7.27

Figure 171: Table displaying the DFT NMR shift predictions (PPM) for molecule IP5 at the three levels of theory tested and the corresponding experimental values (PPM) assigned by DP4-AI

5.11 JB10

	MM calc exp	Opt calc exp	Opt E calc exp
C1	54.97 56.05	56.70 56.05	56.76 56.05
C10	134.31 129.62	133.96 127.64	133.25 127.64
C13	26.35 24.68	26.05 24.68	26.09 24.68
C14	32.05 27.11	33.15 33.10	33.53 33.10
C15	23.49 20.02	23.17 20.02	23.18 20.02
C16	15.83 19.24	16.15 19.24	16.57 19.24
C17	141.33 135.51	138.81 130.99	138.85 130.99
C18	34.29 33.10	26.64 26.78	26.68 26.78
C19	141.19 135.48	138.77 129.62	138.72 129.62
C2	62.95 58.84	66.19 58.84	66.25 58.84
C20	27.67 24.80	26.26 24.80	26.15 24.80
C21	27.99 26.78	26.50 26.78	26.47 26.78
C22	28.09 26.78	26.59 26.78	26.63 26.78
C23	140.93 129.62	141.33 135.48	141.22 135.48
C24	132.79 127.63	132.85 127.63	132.88 127.64
C25	134.08 127.64	135.23 127.63	135.21 127.63
C26	132.63 127.63	132.75 127.63	132.72 127.63
C27	140.96 133.59	141.15 133.59	141.09 133.59
C28	141.03 135.51	141.15 135.51	141.10 135.51
C29	132.84 127.63	132.85 127.64	132.92 127.63
C3	39.50 34.69	38.84 34.69	39.01 34.69
C30	134.16 127.64	135.28 129.62	135.29 129.62
C31	132.72 127.64	132.80 127.64	132.82 127.64
C32	140.95 130.99	141.48 135.51	141.41 135.51
C34	72.69 67.26	72.58 67.26	72.10 67.26
C4	24.33 22.04	25.45 22.04	25.85 22.04
C5	52.26 51.30	54.36 51.30	54.53 51.30
C6	229.01 213.54	226.15 213.54	226.22 213.54
C7	42.44 39.57	43.48 39.57	43.61 39.57
C8	28.12 26.78	29.45 27.11	29.39 27.11
C9	152.62 135.48	150.60 135.48	150.16 135.48

	MM calc exp	Opt calc exp	Opt E calc exp
H35	2.71 2.44	2.54 2.44	2.55 2.44
H36	1.31 1.26	1.20 1.05	1.22 1.26
H37	2.62 2.44	2.47 2.44	2.46 2.44
H38	1.35 1.26	1.40 1.26	1.40 1.26
H39	1.68 1.59	1.71 1.26	1.73 1.59
H40	2.14 2.15	2.00 2.15	1.99 2.15
H41	2.52 2.44	2.49 2.44	2.50 2.44
H42	2.73 2.76	2.84 2.76	2.80 2.76
H43	2.23 2.15	2.32 2.15	2.28 2.15
H44	2.25 2.15	2.43 2.28	2.32 2.28
H45	6.30 5.55	6.25 5.55	6.32 5.55
H46	1.66 1.26	1.75 1.59	1.73 1.59
H47	1.01 1.05	0.94 1.05	0.94 1.05
H48	1.21 1.05	1.27 1.05	1.28 1.26
H49	1.94 1.79	1.98 1.79	1.99 1.79
H50	0.85 0.91	0.84 0.91	0.84 0.91
H51	1.04 1.05	1.03 1.05	1.03 1.05
H52	0.92 1.05	0.96 1.05	0.98 1.05
H53	0.75 0.91	0.78 0.91	0.78 0.91
H54	1.10 1.05	1.11 1.05	1.13 1.05
H55	0.85 0.91	0.84 0.91	0.85 0.91
H56	1.28 1.05	1.10 1.05	1.14 1.05
H57	1.20 1.05	1.10 1.05	1.08 1.05
H58	0.74 0.91	0.67 0.91	0.68 0.91
H59	1.28 1.05	1.22 1.05	1.16 1.05
H60	1.59 1.26	1.44 1.26	1.43 1.26
H61	0.84 0.91	0.77 0.91	0.76 0.91
H62	1.61 1.26	1.46 1.26	1.49 1.26
H63	1.38 1.26	1.29 1.26	1.30 1.26
H64	0.89 0.91	0.79 0.91	0.80 0.91
H65	7.87 7.65	7.97 7.65	8.01 7.65
H66	7.58 7.39	7.67 7.39	7.68 7.39
H67	7.60 7.39	7.70 7.39	7.70 7.39
H68	7.56 7.39	7.68 7.39	7.67 7.39
H69	7.82 7.65	8.02 7.65	7.99 7.65
H70	7.68 7.39	7.94 7.65	7.94 7.65
H71	7.54 7.39	7.66 7.39	7.66 7.39
H72	7.61 7.39	7.70 7.39	7.70 7.39
H73	7.63 7.39	7.70 7.39	7.71 7.39
H74	8.07 7.65	8.06 7.65	8.07 7.65
H75	4.00 4.07	4.13 4.07	4.14 4.07
H76	3.98 4.07	4.11 4.07	4.12 4.07

Figure 172: Table displaying the DFT NMR shift predictions (PPM) for molecule JB10 at the three levels of theory tested and the corresponding experimental values (PPM) assigned by DP4-AI

5.12 JB11

	MM<br>calc exp	Opt<br>calc exp	Opt E<br>calc exp
C1	55.12 54.88	56.70 54.07	56.19 54.07
C10	158.38 148.58	156.53 148.58	156.19 148.58
C11	118.34 110.81	117.91 110.81	120.85 110.81
C12	67.66 64.17	67.06 64.17	67.61 64.17
C15	141.00 128.10	138.71 128.10	138.23 128.10
C16	34.29 30.76	26.42 27.30	26.43 27.30
C17	140.75 128.10	138.16 128.10	138.09 128.10
C18	27.56 27.30	26.13 27.30	25.96 27.30
C19	28.14 27.30	26.66 27.30	27.01 27.40
C2	61.37 58.94	62.58 58.94	63.09 58.94
C20	28.40 27.30	27.02 27.40	26.87 27.30
C21	141.04 128.10	141.37 128.10	141.76 128.10
C22	132.58 128.10	132.83 128.10	133.15 128.10
C23	133.74 128.10	134.78 128.10	134.66 128.10
C24	132.39 128.10	132.32 128.10	132.18 128.10
C25	141.00 128.10	141.16 128.10	140.93 128.10
C26	140.88 128.10	141.20 128.10	141.36 128.10
C27	132.46 128.10	132.37 128.10	132.55 128.10
C28	133.78 128.10	134.87 128.10	134.93 128.10
C29	132.66 128.10	132.55 128.10	132.41 128.10
C3	53.84 54.07	59.59 54.88	59.88 54.88
C30	140.97 128.10	141.53 128.10	141.50 128.10
C31	29.24 27.30	26.79 27.30	26.87 27.30
C33	36.41 32.41	37.88 32.41	37.89 32.41
C34	24.75 22.46	24.00 22.46	24.13 22.46
C35	20.51 20.59	20.84 20.59	21.28 20.59
C4	42.99 41.81	44.25 41.81	43.81 41.81
C5	33.31 27.40	34.95 30.76	35.27 30.76
C6	183.88 173.40	181.33 173.40	181.89 173.40
C8	91.10 88.03	92.37 88.03	93.71 88.03
C9	84.90 78.32	80.25 78.32	80.04 78.32

	MM<br>calc exp	Opt<br>calc exp	Opt E<br>calc exp
H32	2.16 1.93	2.44 1.93	2.92 1.93
H36	1.82 1.51	1.87 1.51	1.86 1.51
H37	2.08 1.80	2.06 1.80	2.10 1.80
H38	1.75 1.40	1.74 1.40	1.74 1.40
H39	1.89 1.63	1.98 1.63	2.01 1.63
H40	1.56 1.28	1.64 1.28	1.68 1.28
H41	4.83 5.31	4.90 5.31	4.77 5.31
H42	3.82 3.85	3.67 3.85	3.78 3.85
H43	4.24 4.44	4.22 4.44	4.35 4.44
H44	5.56 7.18	5.48 7.18	5.38 7.18
H45	5.42 5.49	5.35 5.49	5.38 5.49
H46	4.32 4.44	4.36 4.44	4.07 4.44
H47	4.12 4.44	4.29 4.44	4.52 4.44
H48	1.36 1.17	1.23 1.17	1.08 1.17
H49	1.46 1.17	1.24 1.17	1.23 1.17
H50	0.80 0.74	0.70 0.74	0.68 0.74
H51	1.45 1.17	1.19 1.17	1.04 1.17
H52	1.55 1.17	1.46 1.17	1.45 1.17
H53	0.85 0.74	0.75 0.74	0.71 0.74
H54	1.14 1.17	1.23 1.17	1.26 1.17
H55	0.90 0.74	0.79 0.74	0.84 0.74
H56	1.36 1.17	1.38 1.17	1.50 1.17
H57	7.83 7.79	8.03 7.79	8.08 7.79
H58	7.53 7.18	7.64 7.18	7.67 7.18
H59	7.56 7.18	7.65 7.18	7.65 7.18
H60	7.55 7.18	7.65 7.18	7.66 7.18
H61	7.89 7.79	7.99 7.79	7.99 7.79
H62	7.72 7.79	7.94 7.79	7.96 7.79
H63	7.52 7.18	7.62 7.18	7.60 7.18
H64	7.57 7.18	7.64 7.18	7.64 7.18
H65	7.58 7.18	7.66 7.18	7.67 7.18
H66	7.97 7.79	8.13 7.79	8.10 7.79
H67	1.18 1.17	1.20 1.17	1.25 1.17
H68	1.23 1.17	1.32 1.17	1.37 1.17
H69	1.15 1.17	1.12 1.17	1.30 1.17
H70	1.60 1.28	1.68 1.28	1.73 1.28
H71	1.16 1.17	1.33 1.17	1.54 1.17
H72	0.99 1.17	1.06 1.17	1.07 1.17
H73	0.78 0.74	0.79 0.74	0.75 0.74
H74	0.62 0.74	0.68 0.74	0.68 0.74
H75	0.86 0.74	0.95 0.74	0.95 0.74
H76	0.99 1.17	1.14 1.17	1.13 1.17

Figure 173: Table displaying the DFT NMR shift predictions (PPM) for molecule JB11 at the three levels of theory tested and the corresponding experimental values (PPM) assigned by DP4-AI

5.13 JB12

	MM		Opt		Opt E	
	calc	exp	calc	exp	calc	exp
C1	55.22	54.26	58.54	54.26	58.56	54.26
C10	157.47	135.43	154.86	135.43	153.73	135.43
C11	121.77	112.06	120.03	112.06	123.29	112.06
C12	69.47	63.13	67.95	63.13	69.16	63.13
C15	140.19	135.38	138.79	135.38	138.76	135.38
C16	34.58	30.23	26.26	21.68	26.66	21.68
C17	140.02	129.82	138.64	129.82	138.50	129.82
C18	28.21	26.72	27.28	26.72	27.16	26.72
C19	28.05	26.72	27.61	26.72	26.79	26.72
C2	56.58	54.96	58.68	54.96	60.35	54.96
C20	27.51	21.68	26.68	26.72	26.87	26.72
C21	141.52	135.43	141.76	135.38	141.40	135.38
C22	132.66	127.75	132.91	127.75	132.78	127.75
C23	134.42	129.82	135.66	129.82	135.48	127.75
C24	132.80	127.75	132.90	127.75	133.18	127.75
C25	141.74	135.43	142.52	135.43	142.46	135.43
C26	140.98	135.38	142.02	135.43	141.96	135.43
C27	132.83	127.75	132.54	127.75	132.77	127.75
C28	134.41	127.75	135.46	127.75	135.60	129.82
C29	133.04	127.75	132.80	127.75	132.82	127.75
C3	59.64	61.55	65.57	61.55	66.31	61.55
C30	141.45	135.38	141.98	135.38	141.76	135.38
C31	186.18	170.99	181.38	170.99	182.40	170.99
C32	30.88	29.57	32.05	30.23	35.61	30.23
C33	24.36	19.86	23.95	19.86	23.87	19.86
C34	17.54	19.21	16.69	19.21	20.74	19.21
C37	53.94	52.81	55.30	52.81	55.20	52.81
C4	39.71	34.15	39.48	34.15	39.15	34.15
C5	29.98	26.72	30.80	29.57	34.60	29.57
C6	189.44	175.56	184.32	175.56	184.92	175.56
C8	81.71	84.63	86.49	84.63	87.34	84.63

	MM		Opt		Opt E	
	calc	exp	calc	exp	calc	exp
H38	2.02	1.55	2.03	1.55	1.91	1.55
H39	3.02	3.41	2.76	2.70	3.31	3.41
H40	2.26	2.32	2.04	1.96	2.07	2.32
H41	2.29	2.70	2.45	2.32	2.42	2.70
H42	1.70	1.55	1.70	1.55	1.93	1.96
H43	1.69	1.55	1.53	1.55	1.69	1.55
H44	5.38	5.10	5.26	5.10	5.16	5.10
H45	5.40	5.24	5.41	5.24	5.42	5.24
H46	5.43	5.24	5.47	5.24	5.64	5.24
H47	4.03	4.12	4.07	4.12	4.03	4.12
H48	4.12	4.12	4.24	4.12	4.31	4.12
H49	1.48	1.55	1.62	1.55	1.73	1.55
H50	1.57	1.55	1.65	1.55	1.59	1.55
H51	0.90	0.94	0.96	0.94	0.93	0.94
H52	1.41	1.55	1.43	0.94	1.16	0.94
H53	1.20	0.94	1.07	0.94	1.01	0.94
H54	0.80	0.73	0.75	0.94	0.69	0.73
H55	0.78	0.73	0.75	0.94	0.74	0.94
H56	1.26	0.94	1.04	0.94	0.90	0.94
H57	1.34	0.94	1.36	0.94	1.48	0.94
H58	7.92	7.54	8.04	7.54	7.97	7.54
H59	7.60	7.27	7.70	7.27	7.70	7.27
H60	7.64	7.27	7.72	7.27	7.72	7.27
H61	7.58	7.27	7.66	7.27	7.71	7.27
H62	7.77	7.54	7.82	7.54	7.94	7.54
H63	8.16	7.54	8.16	7.54	8.13	7.54
H64	7.68	7.54	7.73	7.27	7.72	7.27
H65	7.65	7.27	7.70	7.27	7.70	7.27
H66	7.55	7.27	7.59	7.27	7.61	7.27
H67	7.54	7.27	7.57	7.27	7.81	7.54
H68	2.16	1.96	1.87	1.55	1.91	1.55
H69	0.98	0.94	0.91	0.94	0.98	0.94
H70	1.20	0.94	1.09	0.94	1.18	0.94
H71	1.11	0.94	1.12	0.94	1.48	1.55
H72	0.96	0.94	0.58	0.73	1.02	0.94
H73	0.85	0.94	0.34	0.73	0.73	0.73
H74	0.73	0.73	0.46	0.73	0.70	0.73
H75	3.51	3.41	3.55	3.41	3.48	3.41
H76	3.55	3.63	3.64	3.41	3.62	3.63
H77	3.45	3.41	3.54	3.41	3.36	3.41

Figure 174: Table displaying the DFT NMR shift predictions (PPM) for molecule JB12 at the three levels of theory tested and the corresponding experimental values (PPM) assigned by DP4-AI

5.14 JB13A

	MM		Opt		Opt E	
	calc	exp	calc	exp	calc	exp
C1	139.12	128.88	137.77	128.88	137.72	128.88
C12	138.51	128.88	137.87	128.88	137.86	128.88
C14	28.82	25.71	28.44	25.71	28.13	25.71
C15	13.53	10.63	13.01	10.63	12.77	10.63
C2	45.16	41.61	47.04	41.61	46.98	41.61
C3	75.47	74.72	78.69	74.72	78.72	74.72
C5	175.97	167.72	174.65	167.72	174.68	167.72
C6	182.76	169.88	177.10	169.88	177.11	169.88
C8	80.53	81.81	84.99	81.81	84.96	81.81

	MM		Opt		Opt E	
	calc	exp	calc	exp	calc	exp
H10	4.85	5.12	4.99	5.12	4.99	5.12
H11	3.59	4.00	3.75	4.00	3.75	4.00
H16	4.46	4.69	4.55	4.69	4.55	4.69
H17	6.86	6.61	6.82	6.61	6.83	6.61
H18	6.20	5.88	6.14	5.88	6.14	5.88
H19	1.82	1.76	1.73	1.76	1.75	1.76
H20	1.80	1.76	1.77	1.76	1.81	1.76
H21	1.30	1.12	1.30	1.12	1.26	1.12
H22	1.19	1.12	1.25	1.12	1.25	1.12
H23	1.14	1.12	1.15	1.12	1.15	1.12

Figure 175: Table displaying the DFT NMR shift predictions (PPM) for molecule JB13A at the three levels of theory tested and the corresponding experimental values (PPM) assigned by DP4-AI

5.15 JB13B

	MM calc exp	Opt calc exp	Opt E calc exp
C1	47.02 43.65	143.31 126.35	137.72 126.35
C10	135.71 126.35		
C13	32.39 28.99		
C14	12.70 9.05	33.67 28.99	28.13 28.99
C2	74.66 74.24	48.45 43.65	46.98 43.65
C4	175.60 167.47		
C5	142.65 126.35	174.43 167.47	174.68 167.47
C6	182.47 169.77	176.71 169.77	177.11 169.77
C8	83.23 86.27	91.82 86.27	84.96 86.27

	MM calc exp	Opt calc exp	Opt E calc exp
H11	4.77 5.06	3.39 3.58	3.75 3.58
H12	3.31 3.58		
H16	4.30 4.39	4.35 4.39	4.55 4.39
H17	6.75 6.47	6.67 6.47	6.83 6.47
H18	6.14 5.88	6.14 5.88	6.14 5.88
H19	1.76 1.86	1.82 1.86	1.75 1.86
H20	1.86 1.86	1.76 1.86	1.81 1.86
H21	1.15 1.08	1.28 1.08	1.26 1.08
H22	1.02 1.08	1.10 1.08	1.25 1.08
H23	1.28 1.08	1.08 1.08	1.15 1.08

Figure 176: Table displaying the DFT NMR shift predictions (PPM) for molecule JB13B at the three levels of theory tested and the corresponding experimental values (PPM) assigned by DP4-AI

5.16 JB1

	MM calc exp	Opt calc exp	Opt E calc exp
C1	52.09 43.82	46.46 43.82	46.94 43.82
C10	45.37 36.09	40.07 36.09	38.18 36.09
C11	190.30 171.43	181.40 174.88	180.61 174.88
C14	86.71 81.87	88.01 81.87	88.08 81.87
C15	35.81 28.02	30.71 28.02	29.52 28.02
C16	31.19 28.02	25.97 28.02	25.92 28.02
C17	32.28 28.02	26.94 28.02	28.18 28.02
C19	189.49 170.58	178.32 167.51	178.36 167.51
C2	58.73 46.76	53.77 46.76	52.51 46.76
C22	59.87 53.95	56.46 53.95	56.46 53.95
C3	73.56 66.27	70.29 66.27	70.30 66.27
C5	186.66 167.51	178.68 170.58	178.76 170.58
C6	193.44 174.88	179.30 171.43	179.30 171.43
C8	77.55 72.15	75.18 72.15	75.00 72.15

	MM calc exp	Opt calc exp	Opt E calc exp
H18	3.40 3.20	2.82 2.93	3.08 3.20
H24	2.60 2.80	2.75 2.80	2.58 2.46
H25	6.04 6.64	5.32 4.75	5.35 4.75
H26	4.87 4.75	4.75 3.88	4.76 4.60
H27	4.86 4.60	4.96 4.60	4.67 3.88
H28	2.52 2.46	2.33 1.44	2.58 2.80
H29	3.10 2.93	2.99 3.20	2.76 2.93
H30	1.64 1.44	1.63 1.44	1.80 1.44
H31	1.16 1.44	1.25 1.44	1.16 1.44
H32	1.31 1.44	1.26 1.44	1.23 1.44
H33	2.36 1.44	2.43 2.46	2.23 1.44
H34	1.19 1.44	1.13 1.44	1.34 1.44
H35	1.14 1.44	1.23 1.44	1.14 1.44
H36	2.13 1.44	2.31 1.44	1.89 1.44
H37	1.10 1.44	1.18 1.44	1.12 1.44
H38	1.24 1.44	1.18 1.44	1.46 1.44
H39	3.83 3.88	3.88 3.88	3.87 3.88
H40	3.83 3.88	3.85 3.88	3.86 3.88
H41	3.86 3.88	3.87 3.88	3.88 3.88

Figure 177: Table displaying the DFT NMR shift predictions (PPM) for molecule JB1 at the three levels of theory tested and the corresponding experimental values (PPM) assigned by DP4-AI

5.17 JB2

	MM		Opt		Opt E	
	calc	exp	calc	exp	calc	exp
C1	58.59	46.96	53.37	46.96	52.43	46.96
C11	186.72	170.63	177.04	165.86	177.18	165.86
C14	88.00	85.41	91.77	85.41	91.82	85.41
C15	30.70	27.76	25.77	27.76	25.81	27.76
C16	30.99	28.00	26.38	28.00	26.37	28.00
C17	36.74	28.00	31.09	28.00	31.04	28.00
C19	45.28	36.06	40.39	36.06	39.05	36.06
C2	77.57	71.94	75.46	71.94	75.30	71.94
C20	190.30	171.94	181.62	174.90	180.97	174.90
C23	86.64	81.75	87.84	81.75	87.85	81.75
C24	37.18	28.00	31.49	28.00	31.50	28.00
C25	30.92	27.76	26.08	27.76	26.08	27.76
C26	30.87	27.76	26.24	27.76	26.17	27.76
C4	193.54	174.90	179.83	171.94	179.86	171.94
C5	73.29	71.94	70.92	71.94	70.90	71.94
C6	52.01	43.82	47.04	43.82	47.38	43.82
C7	186.43	165.86	178.47	170.63	178.59	170.63

	MM		Opt		Opt E	
	calc	exp	calc	exp	calc	exp
H10	3.42	3.11	2.84	2.93	2.97	3.11
H27	4.83	4.68	4.77	4.58	4.77	4.68
H28	4.79	4.58	5.04	4.68	4.77	4.58
H29	2.60	2.78	2.73	2.78	2.58	2.78
H30	6.00	6.60	5.32	6.60	5.33	6.60
H31	1.24	1.47	1.37	1.47	1.38	1.47
H32	1.21	1.47	1.13	1.47	1.14	1.47
H33	2.34	1.47	2.37	1.47	2.38	1.47
H34	2.34	1.47	2.39	1.47	2.42	1.47
H35	1.18	1.47	1.32	1.47	1.38	1.47
H36	1.22	1.47	1.17	1.47	1.18	1.47
H37	1.47	1.47	1.61	1.47	1.63	1.47
H38	1.23	1.47	1.16	1.47	1.16	1.47
H39	1.52	1.47	1.65	1.47	1.65	1.47
H40	2.50	2.46	2.33	1.47	2.57	2.46
H41	3.11	2.93	3.02	3.11	2.81	2.93
H42	1.15	1.47	1.06	1.47	1.05	1.47
H43	1.40	1.47	1.47	1.47	1.47	1.47
H44	1.45	1.47	1.51	1.47	1.50	1.47
H45	2.32	1.47	2.44	1.47	2.42	1.47
H46	1.13	1.47	1.22	1.47	1.21	1.47
H47	1.17	1.47	1.09	1.47	1.07	1.47
H48	1.19	1.47	1.28	1.47	1.25	1.47
H49	2.34	1.47	2.47	2.46	2.44	1.47
H50	1.19	1.47	1.08	1.47	1.07	1.47

Figure 178: Table displaying the DFT NMR shift predictions (PPM) for molecule JB2 at the three levels of theory tested and the corresponding experimental values (PPM) assigned by DP4-AI

5.18 JB3

	MM		Opt		Opt E	
	calc	exp	calc	exp	calc	exp
C1	187.10	166.24	179.71	171.17	179.74	171.17
C10	188.86	170.60	176.97	166.24	176.98	166.24
C13	88.64	85.13	90.96	85.13	90.97	85.13
C14	35.72	27.93	29.69	27.93	29.81	27.93
C15	30.82	27.74	26.11	27.74	26.06	27.74
C16	32.13	27.93	27.54	27.93	27.45	27.93
C18	39.47	32.17	34.67	32.17	34.76	32.17
C19	189.96	171.17	180.89	175.37	180.94	175.37
C2	47.59	39.64	43.44	39.64	43.46	39.64
C22	86.66	81.83	87.76	81.83	87.76	81.83
C23	31.54	27.74	26.67	27.74	26.81	27.74
C24	36.85	27.93	30.79	27.93	30.66	27.93
C25	30.68	27.74	26.06	27.74	26.04	27.74
C4	73.60	67.37	70.78	67.37	70.80	67.37
C6	192.06	175.37	178.70	170.60	178.76	170.60
C8	72.53	67.37	70.68	67.37	70.71	67.37

	MM		Opt		Opt E	
	calc	exp	calc	exp	calc	exp
H27	2.94	2.66	2.89	3.20	2.88	3.20
H28	5.68	4.86	5.00	4.86	5.01	4.86
H29	3.98	3.40	4.00	4.01	4.01	4.01
H30	4.49	4.49	4.39	4.49	4.40	4.49
H31	1.21	1.79	1.22	1.79	1.21	1.79
H32	1.41	1.79	1.45	1.79	1.47	1.79
H33	1.62	1.79	1.74	1.79	1.72	1.79
H34	1.24	1.79	1.41	1.79	1.40	1.79
H35	2.17	1.79	2.08	1.79	2.11	1.79
H36	1.46	1.79	1.43	1.79	1.40	1.79
H37	2.20	1.79	2.19	1.79	2.21	1.79
H38	1.24	1.79	1.17	1.79	1.17	1.79
H39	1.27	1.79	1.49	1.79	1.48	1.79
H40	2.36	1.79	2.16	1.79	2.17	1.79
H41	3.13	3.20	2.86	2.66	2.87	2.66
H42	1.19	1.79	1.12	1.79	1.11	1.79
H43	2.30	1.79	2.32	1.79	2.30	1.79
H44	1.14	1.79	1.25	1.79	1.26	1.79
H45	1.47	1.79	1.56	1.79	1.59	1.79
H46	1.40	1.79	1.45	1.79	1.45	1.79
H47	1.17	1.79	1.10	1.79	1.10	1.79
H48	2.16	1.79	2.18	1.79	2.21	1.79
H49	1.23	1.79	1.20	1.79	1.24	1.79
H50	1.27	1.79	1.40	1.79	1.34	1.79
H9	4.34	4.01	3.72	3.40	3.71	3.40

Figure 179: Table displaying the DFT NMR shift predictions (PPM) for molecule JB3 at the three levels of theory tested and the corresponding experimental values (PPM) assigned by DP4-AI

5.19 JB4

	MM calc exp	Opt calc exp	Opt E calc exp
C1	44.33 42.41	46.25 42.41	45.82 42.41
C12	79.94 81.10	86.94 81.10	86.95 81.10
C13	26.15 27.78	26.08 27.78	26.14 27.78
C14	32.38 27.98	31.48 27.98	31.71 27.98
C15	26.22 27.78	26.14 27.78	25.89 27.78
C16	71.37 68.60	74.95 68.60	75.54 70.85
C18	184.14 170.30	179.83 170.30	180.22 170.30
C2	157.45 143.94	155.97 143.94	156.18 143.94
C21	80.92 82.91	89.35 82.91	89.17 82.91
C22	30.64 27.98	28.16 27.98	28.81 27.98
C23	27.00 27.78	27.08 27.78	26.94 27.78
C24	27.12 27.98	28.09 27.98	27.73 27.98
C3	74.50 70.85	75.18 70.85	74.99 68.60
C5	186.46 176.77	181.86 176.77	181.41 176.77
C7	122.18 111.11	116.29 111.11	116.47 111.11
C8	39.35 27.98	38.74 27.98	38.59 27.98
C9	182.21 169.39	179.76 169.39	179.59 169.39

	MM calc exp	Opt calc exp	Opt E calc exp
H25	3.01 2.81	3.12 3.41	2.91 2.81
H26	5.16 5.24	4.98 5.24	5.12 5.24
H27	5.92 6.50	5.89 6.50	5.91 6.50
H28	5.66 5.46	5.32 5.46	5.31 5.46
H29	3.16 3.41	2.87 2.81	3.01 3.41
H30	2.82 2.57	2.56 2.57	2.69 2.57
H31	1.17 1.46	1.37 1.46	1.58 1.46
H32	1.07 1.46	1.16 1.46	1.08 1.46
H33	2.17 1.46	2.12 1.46	1.84 1.46
H34	1.42 1.46	1.43 1.46	1.43 1.46
H35	1.39 1.46	1.49 1.46	1.43 1.46
H36	1.09 1.46	1.03 1.25	1.02 1.25
H37	2.22 1.46	2.12 1.46	1.84 1.46
H38	1.32 1.46	1.45 1.46	1.72 1.46
H39	1.09 1.46	1.09 1.46	1.10 1.46
H40	3.96 3.64	4.00 4.08	4.03 4.08
H41	4.10 4.08	3.87 3.64	3.83 3.64
H42	1.49 1.46	1.20 1.46	1.14 1.46
H43	1.60 1.46	1.67 1.46	1.69 1.46
H44	1.18 1.46	1.11 1.46	1.11 1.46
H45	1.67 1.46	1.93 1.46	1.91 1.46
H46	1.39 1.46	1.80 1.46	1.73 1.46
H47	2.20 1.46	1.92 1.46	2.09 1.46
H48	1.12 1.46	1.11 1.46	1.11 1.46
H49	1.81 1.46	1.64 1.46	1.77 1.46
H50	1.09 1.46	1.23 1.46	1.10 1.46
H51	2.05 1.46	1.84 1.46	1.97 1.46

Figure 180: Table displaying the DFT NMR shift predictions (PPM) for molecule JB4 at the three levels of theory tested and the corresponding experimental values (PPM) assigned by DP4-AI

5.20 JB5

	MM	Opt	Opt E
	calc exp	calc exp	calc exp
C1	50.47 47.44	52.26 47.44	52.32 47.44
C10	69.40 66.50	68.11 63.32	68.14 63.32
C12	21.96 18.58	20.96 18.58	20.94 18.58
C18	68.70 63.32	71.79 66.50	71.82 66.50
C19	134.61 129.63	131.77 129.63	131.74 129.63
C2	93.30 89.83	98.18 89.83	98.14 89.83
C20	137.90 129.63	138.81 129.63	138.83 129.63
C21	117.90 113.83	117.86 113.83	117.90 113.83
C22	165.16 159.19	167.63 159.19	167.65 159.19
C23	119.26 113.83	118.09 113.83	118.06 113.83
C24	138.07 129.63	138.64 129.63	138.69 129.63
C26	55.40 55.12	56.13 55.12	56.13 55.12
C3	75.49 72.30	77.96 72.30	77.91 72.30
C5	181.03 168.12	179.51 173.66	179.47 173.66
C7	185.61 173.76	181.69 173.76	181.66 173.76
C8	32.15 30.30	33.69 30.30	33.71 30.30
C9	182.22 173.66	177.58 168.12	177.59 168.12

	MM	Opt	Opt E
	calc exp	calc exp	calc exp
H13	3.09 3.01	2.93 3.01	2.93 3.01
H27	5.38 5.51	4.87 5.12	4.88 5.12
H28	2.74 2.51	2.59 2.51	2.58 2.51
H29	2.77 3.01	2.79 3.01	2.78 3.01
H30	4.12 3.76	4.11 3.76	4.12 3.76
H31	3.87 3.76	3.61 3.63	3.63 3.63
H32	1.76 1.59	1.24 1.59	1.19 1.59
H33	1.57 1.59	1.54 1.59	1.53 1.59
H34	1.73 1.59	1.77 1.59	1.77 1.59
H35	1.87 1.59	1.91 1.59	1.89 1.59
H36	5.00 5.12	5.24 5.51	5.25 5.51
H37	5.13 5.12	5.08 5.12	5.06 5.12
H38	7.72 7.34	7.74 7.34	7.74 7.34
H39	7.00 6.94	6.99 6.94	7.00 6.94
H40	7.07 6.94	7.01 6.94	7.01 6.94
H41	7.78 7.34	7.72 7.34	7.73 7.34
H42	3.69 3.76	3.75 3.76	3.76 3.76
H43	3.90 3.76	3.93 3.76	3.93 3.76
H44	3.78 3.76	3.81 3.76	3.79 3.76

Figure 181: Table displaying the DFT NMR shift predictions (PPM) for molecule JB5 at the three levels of theory tested and the corresponding experimental values (PPM) assigned by DP4-AI

5.21 JB6

	MM		Opt		Opt E	
	calc	exp	calc	exp	calc	exp
C1	49.71	55.29	51.72	55.29	51.77	55.29
C10	182.17	172.89	178.05	167.79	178.05	167.79
C13	68.10	67.96	71.02	67.96	70.81	67.96
C14	134.38	130.72	132.01	130.72	132.24	130.72
C15	138.05	130.72	138.21	130.72	138.30	130.72
C16	116.85	114.03	117.61	114.03	118.63	114.03
C17	165.18	160.00	167.39	160.00	167.42	160.00
C18	120.04	114.03	117.65	114.03	116.61	114.03
C19	138.13	130.72	138.79	130.72	139.13	130.72
C2	94.66	91.72	98.83	91.72	98.84	91.72
C21	55.05	55.29	55.65	55.29	55.69	55.29
C22	82.53	75.53	79.89	67.96	79.57	67.96
C23	41.05	37.37	41.52	37.37	41.81	37.37
C25	30.49	28.13	31.84	28.13	31.09	28.13
C26	23.15	20.38	23.22	20.38	23.64	20.38
C27	27.39	24.72	28.63	24.72	28.55	24.72
C28	144.21	135.02	147.12	135.02	146.35	135.02
C29	129.55	123.21	129.06	123.21	129.38	123.21
C3	78.29	67.96	80.58	75.53	80.33	75.53
C31	22.06	19.54	20.86	19.54	20.86	19.54
C5	180.66	167.79	179.23	172.89	179.33	172.89
C7	184.25	172.89	180.27	172.89	180.13	172.89
C8	31.87	30.37	33.37	30.37	33.31	30.37

	MM		Opt		Opt E	
	calc	exp	calc	exp	calc	exp
H30	1.91	1.71	1.88	1.71	1.79	1.71
H32	3.03	3.03	2.94	3.03	2.95	3.03
H34	5.23	5.22	4.90	3.94	4.93	3.94
H35	2.75	2.83	2.60	2.72	2.60	2.72
H36	2.60	2.72	2.62	2.83	2.62	2.83
H37	5.20	5.07	5.45	5.22	5.51	5.22
H38	5.05	3.94	5.08	5.07	5.10	5.07
H39	7.78	7.26	7.71	7.26	7.72	7.26
H40	6.98	6.84	6.96	6.84	7.00	6.84
H41	7.02	6.84	6.95	6.84	6.92	6.84
H42	7.68	7.26	7.77	7.26	7.82	7.26
H43	3.67	3.76	3.70	3.76	3.71	3.76
H44	3.79	3.76	3.85	3.76	3.86	3.76
H45	3.82	3.76	3.81	3.76	3.79	3.76
H46	4.23	3.76	4.07	3.76	4.00	3.76
H47	1.52	1.34	1.34	1.34	1.22	1.34
H48	1.69	1.71	1.54	1.71	1.43	1.34
H49	1.52	1.58	1.39	1.34	1.20	1.34
H50	1.91	1.71	1.76	1.71	1.61	1.71
H51	1.52	1.34	1.39	1.34	1.27	1.34
H52	1.99	1.90	2.06	1.90	2.03	1.90
H53	1.98	1.90	2.02	1.90	1.95	1.71
H54	6.33	5.95	6.42	5.95	6.37	5.95
H55	5.62	5.53	5.67	5.53	5.70	5.53
H56	1.40	1.34	1.51	1.58	1.57	1.58
H57	1.75	1.71	1.96	1.90	1.99	1.90
H58	2.07	1.90	1.91	1.71	1.81	1.71

Figure 182: Table displaying the DFT NMR shift predictions (PPM) for molecule JB6 at the three levels of theory tested and the corresponding experimental values (PPM) assigned by DP4-AI

5.22 JB7

	MM	Opt	Opt E
	calc exp	calc exp	calc exp
C1	53.01 51.09	54.51 51.09	55.08 51.09
C10	67.35 68.12	70.61 68.12	70.35 68.12
C11	135.22 130.62	132.70 130.62	132.85 130.62
C12	138.40 132.19	139.19 132.19	139.13 132.19
C13	118.46 115.03	118.38 115.03	119.52 115.03
C14	165.28 161.59	167.62 161.59	167.61 161.59
C15	118.72 115.03	117.48 115.03	116.22 115.03
C16	138.16 132.19	138.94 132.19	139.19 132.19
C18	55.32 55.89	56.16 55.89	56.16 55.89
C19	82.65 82.70	80.57 68.12	79.80 68.12
C2	88.95 82.70	89.97 82.70	89.61 82.70
C21	41.78 40.53	42.40 40.53	42.67 40.53
C22	30.68 28.58	32.11 28.58	31.56 28.58
C23	23.61 20.53	23.42 23.05	24.02 23.05
C24	27.58 26.01	28.79 27.99	28.69 26.01
C25	143.70 132.19	147.12 132.19	146.74 132.19
C26	130.35 128.96	129.26 128.96	129.52 128.96
C28	23.73 23.05	22.16 20.53	22.94 20.53
C3	81.59 68.12	84.01 82.70	82.97 82.70
C30	28.99 27.99	28.69 26.01	29.51 27.99
C31	63.61 62.24	65.25 62.24	65.11 62.24
C5	187.55 181.43	186.45 181.43	185.27 181.43
C7	183.77 181.43	180.33 181.43	180.41 181.43

	MM	Opt	Opt E
	calc exp	calc exp	calc exp
H27	1.61 1.50	1.72 1.50	1.66 1.50
H33	3.08 2.81	3.07 2.81	2.95 2.81
H34	5.36 5.67	5.23 5.16	5.16 4.97
H35	5.26 5.16	5.10 4.97	5.27 5.16
H36	5.07 4.97	5.43 5.67	5.29 5.67
H37	7.77 7.29	7.80 7.29	7.80 7.29
H38	7.00 6.83	7.00 7.29	7.03 7.29
H39	7.04 7.29	6.99 6.83	6.95 6.83
H40	7.77 7.29	7.75 7.29	7.78 7.29
H41	3.81 3.72	3.79 3.72	3.82 3.72
H42	3.81 3.72	3.83 3.72	3.83 3.72
H43	3.73 3.66	3.83 3.72	3.81 3.72
H44	4.23 3.84	4.12 3.84	4.05 3.84
H45	1.44 1.29	1.33 1.10	1.21 1.10
H46	1.70 1.50	1.59 1.50	1.59 1.50
H47	1.40 1.10	1.40 1.29	1.35 1.29
H48	1.85 1.66	1.78 1.66	1.66 1.50
H49	1.42 1.29	1.35 1.29	1.22 1.29
H50	1.95 1.80	2.05 1.80	2.04 1.80
H51	1.91 1.80	2.00 1.80	1.94 1.80
H52	6.24 6.83	6.39 6.83	6.35 6.83
H53	5.54 5.67	5.65 5.67	5.66 5.67
H54	1.59 1.50	1.72 1.50	1.74 1.50
H55	1.93 1.80	1.87 1.80	1.82 1.66
H56	1.63 1.50	1.48 1.50	1.53 1.50
H57	4.19 3.72	3.41 3.66	3.34 3.66
H58	1.81 1.50	1.78 1.50	1.84 1.80
H59	2.20 1.92	1.95 1.80	1.87 1.80
H60	3.80 3.72	3.75 3.66	3.79 3.72
H61	3.87 3.72	3.87 3.72	3.93 3.72
H62	2.03 1.80	2.51 1.92	2.21 1.92

Figure 183: Table displaying the DFT NMR shift predictions (PPM) for molecule JB7 at the three levels of theory tested and the corresponding experimental values (PPM) assigned by DP4-AI

5.23 JB8

	MM	Opt	Opt E
	calc exp	calc exp	calc exp
C1	47.83 46.69	46.41 43.79	46.32 43.79
C10	20.59 20.51	19.55 20.51	20.11 20.51
C11	75.18 71.49	74.58 71.49	74.54 71.49
C12	41.53 39.81	42.12 39.81	42.33 39.81
C14	30.20 26.98	30.44 26.98	29.81 26.98
C15	23.75 22.24	23.90 22.24	24.06 22.24
C16	27.86 25.87	28.97 25.87	28.86 25.87
C17	144.51 129.60	143.26 129.60	142.29 129.60
C18	130.43 129.19	131.21 129.19	131.72 129.19
C2	88.51 86.82	92.74 86.82	92.43 86.82
C20	33.25 29.51	31.86 29.51	31.90 29.51
C21	47.81 43.79	54.53 46.69	54.65 46.69
C3	86.97 80.86	82.69 80.86	82.68 80.86
C5	182.88 177.44	182.54 177.44	182.27 177.44
C7	179.05 169.93	177.42 169.93	177.29 169.93

	MM	Opt	Opt E
	calc exp	calc exp	calc exp
H19	2.64 2.55	2.55 2.55	2.50 2.55
H23	2.55 2.55	2.53 2.55	2.60 2.55
H24	5.20 5.03	4.92 5.03	4.94 5.03
H25	1.67 1.76	1.77 1.99	1.81 1.99
H26	1.53 1.76	1.56 1.76	1.58 1.76
H27	1.98 2.14	1.90 1.99	1.89 1.99
H28	4.14 4.32	3.94 4.08	3.92 4.19
H29	1.19 1.44	1.02 1.44	0.95 1.44
H30	1.89 1.99	2.01 2.14	2.02 2.14
H31	1.87 1.99	1.68 1.76	1.64 1.76
H32	2.03 2.14	2.01 2.14	1.98 2.14
H33	1.69 1.76	1.67 1.76	1.68 1.76
H34	2.09 2.38	2.18 2.38	2.18 2.38
H35	2.09 2.14	2.16 2.38	2.14 2.38
H36	6.46 6.47	6.40 6.47	6.37 6.47
H37	5.88 5.95	5.93 5.95	6.00 5.95
H38	2.07 2.14	2.02 2.14	2.07 2.14
H39	1.92 2.14	2.00 2.14	2.01 2.14
H40	3.75 4.08	4.32 4.32	4.30 4.32
H41	3.82 4.19	4.01 4.19	3.92 4.08

Figure 184: Table displaying the DFT NMR shift predictions (PPM) for molecule JB8 at the three levels of theory tested and the corresponding experimental values (PPM) assigned by DP4-AI

5.24 JB9

	MM	Opt	Opt E
	calc exp	calc exp	calc exp
C1	55.02 56.04	56.90 56.04	57.12 56.04
C10	133.95 132.94	133.98 132.94	134.52 132.94
C11	71.84 67.18	73.52 67.18	73.63 67.18
C15	26.17 24.96	25.79 24.96	25.73 24.70
C16	32.21 33.00	33.49 33.00	34.07 33.00
C17	23.43 22.06	23.07 22.06	22.94 22.06
C18	16.00 19.92	16.50 19.92	17.28 19.92
C2	63.02 58.94	66.25 58.94	66.40 58.94
C3	39.47 34.55	38.56 34.55	38.57 34.55
C4	24.52 24.70	25.76 24.70	26.50 24.96
C5	52.32 51.45	54.36 51.45	54.32 51.45
C6	228.75 213.59	225.87 213.59	225.85 213.59
C7	42.35 39.90	42.93 39.90	42.79 39.90
C8	28.39 27.06	29.27 27.06	29.35 27.06
C9	153.55 141.64	151.83 141.64	151.56 141.64

	MM	Opt	Opt E
	calc exp	calc exp	calc exp
H14	2.62 2.54	2.46 2.54	2.46 2.54
H19	1.28 1.27	1.17 1.27	1.18 1.27
H20	2.60 2.43	2.42 2.43	2.40 2.28
H21	1.33 1.27	1.38 1.27	1.38 1.27
H22	1.67 1.59	1.69 1.59	1.74 1.59
H23	2.05 1.79	1.87 1.79	1.86 1.79
H24	2.49 2.28	2.38 2.28	2.36 2.28
H25	2.67 2.82	2.72 2.82	2.73 2.82
H26	2.21 2.08	2.20 2.08	2.20 2.08
H27	2.33 2.28	2.39 2.28	2.41 2.43
H28	6.12 5.52	6.01 5.52	6.04 5.52
H29	4.19 4.02	4.18 4.02	4.19 4.02
H30	4.18 4.02	4.19 4.02	4.20 4.02
H31	0.32 0.90	0.32 0.90	0.36 0.90
H32	0.97 0.90	0.91 0.90	0.93 0.90
H33	1.52 1.59	1.59 1.59	1.56 1.59
H34	1.20 1.27	1.23 1.27	1.24 1.27
H35	1.76 1.59	1.79 1.59	1.78 1.59
H36	0.82 0.90	0.81 0.90	0.80 0.90
H37	0.97 1.27	0.98 1.27	0.97 1.27
H38	0.87 0.90	0.90 0.90	0.93 0.90
H39	0.78 0.90	0.78 0.90	0.79 0.90
H40	0.79 0.90	0.84 0.90	0.86 0.90
H41	1.00 1.27	1.02 1.27	1.01 1.27

Figure 185: Table displaying the DFT NMR shift predictions (PPM) for molecule JB9 at the three levels of theory tested and the corresponding experimental values (PPM) assigned by DP4-AI

5.25 KE1

	MM	Opt	Opt E
	calc exp	calc exp	calc exp
C1	83.38 78.56	87.24 81.62	87.48 81.62
C12	116.20 112.63	118.62 112.63	118.80 112.63
C13	29.37 26.63	28.57 27.35	28.38 27.35
C14	29.28 25.83	27.80 26.63	28.29 26.63
C15	173.44 164.50	172.28 164.50	172.24 164.50
C18	52.76 51.91	54.17 51.91	54.20 51.91
C2		83.33 78.56	83.12 78.56
C21	-4.89 -4.89	-5.13 -4.89	-5.20 -4.89
C22	-4.08 -4.47	-3.48 -4.47	-3.24 -4.47
C23	36.05 27.35	25.43 25.83	25.77 25.83
C24	26.32 25.83	26.14 25.83	26.09 25.83
C25	25.85 25.83	26.03 25.83	26.07 25.83
C26	26.75 25.83	26.25 25.83	26.26 25.83
C3	77.80 74.25	79.95 74.25	80.45 74.25
C4	156.56 146.00	157.32 146.00	157.28 146.00
C5	143.13 135.52	142.77 135.52	143.02 135.52

	MM	Opt	Opt E
	calc exp	calc exp	calc exp
H27	3.80 3.61	3.81 3.61	3.81 3.61
H28	3.80 3.61	3.80 3.61	3.81 3.61
H29	3.74 3.61	3.73 3.61	3.73 3.61
H30	1.36 1.20	1.37 1.20	1.32 1.20
H31	1.35 1.06	1.35 1.20	1.28 1.06
H32	1.39 1.20	1.35 1.20	1.37 1.20
H33	1.37 1.20	1.30 1.06	1.34 1.20
H34	1.41 1.20	1.40 1.20	1.37 1.20
H35	1.38 1.20	1.37 1.20	1.40 1.20
H36	0.72 0.73	0.77 0.73	0.79 0.73
H37	1.24 1.06	1.31 1.06	1.31 1.06
H38	1.14 0.73	1.11 0.73	1.14 0.73
H39	1.00 0.73	0.98 0.73	0.97 0.73
H40	1.01 0.73	0.99 0.73	0.96 0.73
H41	0.67 0.73	0.76 0.73	0.75 0.73
H42	0.69 0.73	0.73 0.73	0.74 0.73
H43	1.25 1.06	1.18 1.06	1.14 1.06
H44	1.35 1.20	1.40 1.20	1.41 1.20
H45	-0.06 -0.05	-0.10 -0.05	-0.12 -0.05
H46	0.32 -0.05	0.31 -0.05	0.27 -0.05
H47	0.12 -0.05	0.11 -0.05	0.13 -0.05
H48	0.03 -0.05	-0.01 -0.05	-0.03 -0.05
H49	0.21 -0.05	0.16 -0.05	0.22 -0.05
H50	0.18 -0.05	0.22 -0.05	0.29 -0.05
H6	5.01 4.94	5.09 4.94	5.12 4.94
H7	4.74 4.50	4.74 4.50	4.72 4.50
H8	4.61 4.50	4.74 4.50	4.77 4.50
H9	6.92 6.49	6.98 7.07	7.00 7.07

Figure 186: Table displaying the DFT NMR shift predictions (PPM) for molecule KE1 at the three levels of theory tested and the corresponding experimental values (PPM) assigned by DP4-AI

5.26 KE2

	MM	Opt	Opt E
	calc exp	calc exp	calc exp
C1	88.08 82.74	89.69 82.74	89.07 82.74
C12	115.58 112.14	118.93 112.14	118.76 112.14
C13	26.19 25.87	26.87 25.87	26.87 25.87
C14	27.02 25.87	24.70 25.73	24.64 25.73
C15	187.77 171.94	183.80 171.94	183.42 171.94
C18	53.94 52.71	55.88 52.71	55.66 52.71
C2	81.97 75.35	82.99 79.11	82.86 79.11
C23	-4.19 -4.52	-3.33 -4.52	-3.84 -4.52
C24	-4.66 -4.61	-5.20 -4.61	-4.76 -4.61
C25	35.87 25.87	24.53 24.45	24.56 24.45
C26	26.30 25.87	25.98 25.87	25.97 25.87
C27	26.09 25.73	26.00 25.87	26.02 25.87
C28	25.86 24.45	26.18 25.87	26.14 25.87
C3	83.29 76.32	80.46 75.35	79.99 75.35
C4	83.39 79.11	80.88 76.32	80.93 76.32
C5	84.50 82.74	83.18 82.74	83.11 82.74

	MM	Opt	Opt E
	calc exp	calc exp	calc exp
H29	3.85 3.82	3.90 3.82	3.88 3.82
H30	3.90 3.82	3.91 3.96	3.88 3.82
H31	3.89 3.82	3.88 3.82	3.87 3.82
H32	1.24 1.27	1.54 1.44	1.52 1.44
H33	1.17 0.94	1.45 1.27	1.44 1.27
H34	1.47 1.44	1.68 1.44	1.63 1.44
H35	1.52 1.44	1.14 1.27	1.14 1.27
H36	1.72 1.44	1.48 1.27	1.48 1.27
H37	1.43 1.27	1.20 1.27	1.19 1.27
H38	1.08 0.94	0.74 0.94	0.74 0.94
H39	0.70 0.94	1.52 1.44	1.48 1.44
H40	1.19 1.27	1.18 1.27	1.19 1.27
H41	1.33 1.27	1.00 0.94	1.03 0.94
H42	1.33 1.27	0.96 0.94	0.97 0.94
H43	0.72 0.94	0.76 0.94	0.76 0.94
H44	1.22 1.27	0.73 0.94	0.74 0.94
H45	0.71 0.94	1.08 0.94	1.07 0.94
H46	1.09 0.94	1.44 1.27	1.42 1.27
H47	0.09 0.13	-0.04 0.13	-0.07 0.13
H48	0.15 0.13	0.17 0.13	0.17 0.13
H49	-0.02 0.13	-0.00 0.13	0.03 0.13
H50	-0.16 0.13	-0.13 0.13	-0.11 0.13
H51	0.19 0.13	0.21 0.13	0.19 0.13
H52	0.26 0.13	0.35 0.13	0.24 0.13
H53	2.35 1.60	1.92 1.60	1.87 1.60
H54	3.48 3.41	3.32 3.41	3.17 3.41
H6	4.53 4.34	4.35 4.34	4.38 4.34
H7	4.63 4.48	4.72 4.61	4.69 4.48
H8	3.98 3.96	3.88 3.82	3.90 3.96
H9	5.05 4.61	4.70 4.48	4.71 4.61

Figure 187: Table displaying the DFT NMR shift predictions (PPM) for molecule KE2 at the three levels of theory tested and the corresponding experimental values (PPM) assigned by DP4-AI

5.27 KE3

	MM calc exp	Opt calc exp	Opt E calc exp
C1	83.50 80.06	85.74 80.06	85.76 80.06
C12	115.99 113.87	120.63 113.87	120.60 113.87
C13	26.10 25.66	26.38 25.66	26.39 25.66
C14	27.05 25.66	24.74 25.31	24.72 25.31
C15	181.62 164.62	176.02 164.62	175.89 164.62
C18	54.76 53.55	56.45 53.55	56.41 53.55
C2	82.49 79.98	84.61 79.98	84.59 79.98
C21	-4.74 -4.95	-4.32 -4.79	-4.36 -4.79
C22	-4.47 -4.79	-5.10 -4.95	-5.05 -4.95
C23	35.78 25.66	24.47 24.32	24.52 24.32
C24	26.02 25.31	25.82 25.66	25.83 25.66
C25	26.43 25.66	25.86 25.66	25.86 25.66
C26	25.81 24.32	25.77 25.66	25.78 25.66
C3	79.24 75.15	78.11 75.15	78.14 75.15
C4	92.27 91.52	95.29 91.52	95.35 91.52
C5	92.75 92.17	97.28 92.17	97.22 92.17

	MM calc exp	Opt calc exp	Opt E calc exp
H27	3.93 3.89	3.89 3.89	3.89 3.89
H28	3.87 3.89	3.94 3.89	3.94 3.89
H29	3.92 3.89	3.94 3.89	3.94 3.89
H30	1.32 1.30	1.45 1.44	1.45 1.44
H31	1.20 0.94	1.33 1.30	1.34 1.30
H32	1.41 1.44	1.52 1.44	1.53 1.44
H33	1.38 1.44	1.13 0.94	1.13 0.94
H34	1.48 1.44	1.40 1.44	1.40 1.44
H35	1.37 1.30	1.25 1.30	1.25 1.30
H36	1.13 0.94	0.77 0.94	0.77 0.94
H37	0.72 0.94	1.24 1.30	1.22 1.30
H38	1.28 0.94	1.23 0.94	1.21 0.94
H39	1.23 0.94	1.18 0.94	1.17 0.94
H40	1.31 1.30	1.06 0.94	1.08 0.94
H41	0.72 0.94	0.76 0.94	0.75 0.94
H42	1.05 0.94	0.77 0.94	0.77 0.94
H43	0.69 0.94	1.07 0.94	1.09 0.94
H44	1.02 0.94	1.22 0.94	1.21 0.94
H45	0.14 0.16	-0.01 0.16	-0.01 0.16
H46	0.00 0.16	0.16 0.16	0.16 0.16
H47	0.09 0.16	0.05 0.16	0.05 0.16
H48	-0.07 0.16	-0.06 0.16	-0.07 0.16
H49	0.18 0.16	0.12 0.16	0.13 0.16
H50	0.21 0.16	0.22 0.16	0.21 0.16
H6	4.63 4.64	4.56 4.64	4.57 4.64
H7	4.67 4.64	4.72 4.64	4.72 4.64
H8	4.35 4.64	4.28 4.64	4.29 4.64
H9	5.92 5.66	5.67 5.66	5.67 5.66

Figure 188: Table displaying the DFT NMR shift predictions (PPM) for molecule KE3 at the three levels of theory tested and the corresponding experimental values (PPM) assigned by DP4-AI

5.28 NL1A

	MM		Opt		Opt E	
	calc	exp	calc	exp	calc	exp
H22	1.92	1.86	2.07	1.86	2.08	1.86
H23	2.06	1.86	2.20	1.86	2.03	1.86
H24	4.32	4.26	4.42	4.26	4.49	4.26
H25	4.29	4.26	4.24	4.26	4.30	4.26
H26	2.40	2.47	2.42	2.31	2.54	2.47
H27	2.24	2.31	2.45	2.47	2.53	2.31
H28	5.57	5.96	5.92	5.96	5.92	5.96
H29	1.79	1.86	2.02	1.86	2.16	1.86
H30	1.77	1.64	2.28	1.86	2.18	1.86
H31	2.06	1.86	2.35	1.86	2.29	1.86
H32	1.79	1.86	1.92	1.64	1.51	1.19
H33	1.02	0.89	1.07	0.89	1.12	0.89
H34	1.52	1.19	1.68	1.19	1.51	1.19
H35	1.40	1.19	1.58	1.19	1.58	1.26
H36	3.67	3.47	3.85	3.47	3.80	3.47
H37	3.51	3.36	3.59	3.36	3.65	3.36
H38	1.19	1.19	1.34	1.19	1.34	1.19
H39	1.15	0.89	1.32	0.89	1.33	0.89
H40	0.77	0.89	1.03	0.89	1.01	0.89
H41	0.76	0.89	1.02	0.89	1.01	0.89
H42	1.14	0.89	1.30	0.89	1.34	0.89
H43	1.13	0.89	1.30	0.89	1.31	0.89
H44	0.75	0.89	1.03	0.89	1.02	0.89
H45	1.12	0.89	1.32	0.89	1.31	0.89
H46	1.13	0.89	1.32	0.89	1.30	0.89
H47	0.40	0.06	0.59	0.06	0.53	0.06
H48	0.32	0.06	0.54	0.06	0.55	0.06
H49	0.32	0.06	0.54	0.06	0.54	0.06
H50	0.38	0.06	0.57	0.06	0.55	0.06
H51	0.26	0.06	0.49	0.06	0.46	0.06
H52	0.32	0.06	0.51	0.06	0.54	0.06

Figure 189: Table displaying the DFT NMR shift predictions (PPM) for molecule NL1A at the three levels of theory tested and the corresponding experimental values (PPM) assigned by DP4-AI

5.29 NL1B

	MM calc exp	Opt calc exp	Opt E calc exp
C1	37.80 41.06	40.24 41.06	40.43 41.06
C12	23.07 22.57	22.13 24.43	21.84 24.43
C13	68.57 67.94	67.53 67.94	67.65 67.94
C16	28.05 25.79	18.40 18.08	18.53 18.08
C17	23.61 25.79	24.47 25.79	24.47 25.79
C18	23.36 24.43	24.53 25.79	24.54 25.79
C19	23.64 25.79	24.50 25.79	24.62 25.79
C2	72.60 74.21	73.76 74.21	71.72 74.21
C20	-7.21 -5.39	-5.75 -5.39	-5.71 -5.39
C21	-7.23 -5.67	-5.93 -5.67	-5.76 -5.67
C4	82.66 79.43	83.24 79.43	82.88 79.43
C5	75.28 76.88	77.89 76.88	78.48 76.88
C6	46.27 46.05	44.73 46.05	44.69 46.05
C7	150.52 145.02	148.66 145.02	147.88 145.02
C9	18.99 18.08	20.70 22.57	20.02 22.57

	MM calc exp	Opt calc exp	Opt E calc exp
H22	1.90 1.86	1.87 1.61	1.81 1.61
H23	2.15 1.86	2.34 1.86	2.28 1.86
H24	4.07 4.09	4.16 4.09	4.17 4.09
H25	4.11 4.09	4.19 4.09	4.16 4.09
H26	2.60 2.37	2.74 2.56	2.77 2.56
H27	2.44 2.37	2.51 2.37	2.55 2.37
H28	5.65 5.97	6.01 5.97	6.01 5.97
H29	2.04 1.86	2.27 1.86	2.30 1.86
H30	1.76 1.61	2.13 1.86	2.18 1.86
H31	1.86 1.86	2.32 1.86	2.14 1.86
H32	3.08 2.56	3.80 3.53	3.71 3.53
H33	1.11 0.91	1.16 0.91	1.15 0.91
H34	1.33 1.12	1.46 1.12	1.40 1.12
H35	1.16 0.91	1.34 0.91	1.34 0.91
H36	3.90 3.65	3.94 3.65	3.95 3.65
H37	4.01 3.73	4.02 3.73	3.97 3.73
H38	1.09 0.91	1.27 0.91	1.24 0.91
H39	1.09 0.91	1.27 0.91	1.34 0.91
H40	0.74 0.91	1.01 0.91	1.01 0.91
H41	0.80 0.91	1.05 0.91	1.05 0.91
H42	1.19 0.91	1.36 0.91	1.32 0.91
H43	1.20 1.12	1.39 1.12	1.35 0.91
H44	0.80 0.91	1.04 0.91	1.05 0.91
H45	1.24 1.12	1.45 1.12	1.46 1.12
H46	1.19 0.91	1.32 0.91	1.38 1.12
H47	0.41 0.10	0.63 0.10	0.61 0.10
H48	0.37 0.10	0.55 0.10	0.60 0.10
H49	0.35 0.10	0.56 0.10	0.53 0.10
H50	0.25 0.10	0.46 0.10	0.47 0.10
H51	0.42 0.10	0.64 0.10	0.63 0.10
H52	0.38 0.10	0.60 0.10	0.58 0.10

Figure 190: Table displaying the DFT NMR shift predictions (PPM) for molecule NL1B at the three levels of theory tested and the corresponding experimental values (PPM) assigned by DP4-AI

5.30 NL2A

	MM calc exp	Opt calc exp	Opt E calc exp
C1	30.66 19.15	31.08 19.15	30.82 19.15
C10	17.73 14.18	18.33 14.18	18.39 14.18
C11	67.25 60.40	37.44 46.85	36.74 45.20
C12	79.05 77.21	141.72 143.61	140.91 143.61
C13		19.19 14.18	18.84 14.18
C14	73.08 77.21	123.61 111.70	121.61 111.70
C15	34.26 45.20	68.71 60.40	68.32 60.40
C17	20.74 18.64	78.55 77.21	78.44 77.21
C18	47.47 46.84	73.56 77.21	73.61 76.41
C2	77.28 77.21	78.52 77.21	74.44 77.21
C20	18.78 18.13	94.49 77.21	94.67 77.21
C21	104.92 98.53	28.89 18.64	28.93 18.64
C22		17.02 14.10	17.06 14.10
C23		36.84 45.20	36.87 46.85
C24		73.86 77.21	74.47 77.21
C26	29.05 18.64	20.15 18.13	20.39 18.13
C27	16.39 14.10	46.07 46.84	45.66 46.84
C28	15.58 14.10	147.77 145.66	147.98 145.66
C29	29.17 19.15	20.89 18.13	20.83 18.13
C30	20.47 18.13	111.69 98.53	111.61 98.53
C4	95.17 97.44	95.51 97.44	95.54 97.44
C6	67.44 69.73	69.08 76.41	68.92 69.73
C7	37.97 46.85	15.86 14.10	15.70 14.10
C8	141.68 143.61	29.13 19.15	29.11 19.15
C9		21.60 18.64	21.55 18.64

	MM calc exp	Opt calc exp	Opt E calc exp
H32	3.99 3.27	4.05 3.64	4.23 3.64
H33	3.34 2.04	3.51 2.30	3.52 2.30
H34	3.83 2.30	3.95 3.27	4.00 3.27
H35	2.01 1.55	1.10 0.88	1.24 1.36
H36	2.50 1.74	1.05 0.88	1.03 0.88
H37	5.82 7.26	2.71 1.87	2.75 1.87
H38	1.92 1.55	1.56 1.36	1.53 1.36
H39	1.62 1.55	1.56 1.36	1.53 1.36
H40	1.96 1.55	1.71 1.55	1.65 1.49
H41	4.64 4.49	1.05 0.88	1.02 0.88
H42	4.21 4.11	0.88 0.88	0.88 0.88
H43	1.81 1.55	1.27 1.36	1.25 1.36
H44	2.46 1.74	1.13 1.36	1.12 0.88
H45	4.18 3.64	2.14 1.55	2.13 1.55
H46	1.42 1.36	0.82 0.88	0.81 0.88
H47	0.62 0.88	2.13 1.55	2.14 1.55
H48	1.11 1.36	2.47 1.74	2.45 1.74
H49	2.20 1.55	2.32 1.55	2.24 1.55
H50	3.07 1.87	1.81 1.55	1.80 1.55
H51	1.74 1.55	2.27 1.55	2.22 1.55
H52	2.13 1.55	5.97 7.26	6.15 7.26
H53	1.87 1.55	4.75 7.26	4.78 7.26
H54	5.77 7.26	4.28 4.11	4.30 4.11
H55	1.55 1.49	1.69 1.49	1.71 1.55
H56	1.46 1.36	1.58 1.36	1.58 1.36
H57	1.50 1.36	1.59 1.36	1.60 1.36
H58	2.27 1.74	2.49 1.74	2.54 1.74
H59	1.21 1.36	1.29 1.36	1.30 1.36
H60	1.15 1.36	1.24 1.36	1.26 1.36
H61	1.07 1.03	1.88 1.55	1.88 1.55
H62	0.97 0.88	2.61 1.74	2.62 1.74
H63	2.48 1.74	4.37 4.49	4.36 4.49
H64	1.44 1.36	1.46 1.36	1.49 1.36
H65	1.41 1.36	0.84 0.88	0.84 0.88
H66	1.53 1.36	1.20 1.36	1.21 1.36
H67	0.86 0.88	2.31 1.55	2.24 1.55
H68	0.70 0.88	3.05 2.04	3.13 2.04
H69	1.11 1.36	2.13 1.55	2.39 1.74
H70	0.91 0.88	2.28 1.55	2.22 1.55
H71	2.04 1.55	2.33 1.74	2.27 1.55
H72	0.63 0.88	6.15 7.26	6.10 7.26

Figure 191: Table displaying the DFT NMR shift predictions (PPM) for molecule NL2A at the three levels of theory tested and the corresponding experimental values (PPM) assigned by DP4-AI

5.31 NL2B

	MM	Opt	Opt E
	calc exp	calc exp	calc exp
C10	140.46 138.88	18.17 18.71	18.18 21.79
C11	16.65 18.18	38.84 32.88	39.23 32.88
C12	124.34 120.39	139.83 138.88	138.73 138.88
C13	70.90 71.87	17.50 18.18	16.14 18.18
C15	86.25 77.21	72.63 77.21	68.55 60.39
C16	74.72 77.21		
C18	97.43 98.48	76.32 77.21	73.50 77.21
C19	22.95 29.74		
C2	67.49 60.39	75.80 77.21	73.21 77.21
C20	21.84 25.31	98.11 98.48	94.56 77.21
C22	31.60 32.88	21.47 23.60	17.03 18.82
C23	75.59 77.21	33.83 30.93	36.78 30.93
C24	47.30 46.45	77.12 77.21	73.92 77.21
C25	150.17 145.28		
C26	18.56 21.79	19.32 18.82	20.17 23.60
C27	106.30 100.43	46.71 36.60	46.57 36.60
C29	19.55 23.60	19.95 21.79	20.49 24.20
C3	30.60 30.93		
C30	20.19 24.20	112.10 100.43	111.78 100.43
C31	18.29 18.82		
C4	73.79 77.21	95.52 77.21	95.52 98.48
C6	95.22 77.21	69.06 71.87	69.10 71.87
C7	15.43 18.07	15.69 18.07	15.74 18.07
C8	28.96 29.70	28.94 29.74	28.91 29.70
C9	39.86 36.60	21.65 24.20	21.58 25.31

	MM	Opt	Opt E
	calc exp	calc exp	calc exp
H32	3.33 1.86	4.02 2.17	4.07 2.04
H33	3.76 2.04	3.50 1.86	3.53 1.86
H34	3.87 2.17	3.90 2.04	3.92 1.86
H35	1.00 1.04	1.08 1.04	1.06 1.04
H36	1.09 1.31	1.14 1.31	1.06 1.04
H37	2.42 1.74	2.68 1.74	2.67 1.74
H38	1.42 1.31	1.56 1.31	1.53 1.31
H39	1.51 1.31	1.62 1.31	1.56 1.31
H40	1.53 1.31	1.70 1.31	1.71 1.31
H41	2.35 1.74	0.89 0.85	0.91 0.85
H42	2.23 1.54	1.26 1.31	1.26 1.31
H43	1.77 1.54	0.85 0.85	0.88 0.85
H44	1.81 1.54	0.79 0.85	0.81 0.85
H45	1.96 1.54	2.14 1.54	2.15 1.54
H46	5.45 7.26	1.12 1.04	1.17 1.04
H47	4.63 5.98	2.40 1.54	2.43 1.54
H48	4.01 3.27	2.25 1.54	2.39 1.54
H49	1.90 1.54	1.86 1.31	2.02 1.54
H50	1.38 1.31	2.43 1.54	2.24 1.54
H51	1.25 1.31	2.12 1.54	1.88 1.54
H52	1.73 1.54	5.53 7.26	6.03 5.98
H53	1.18 1.31	4.68 5.98	4.72 5.19
H54	1.39 1.31	4.06 3.27	4.27 2.17
H55	1.96 1.54	2.09 1.54	1.70 1.31
H56	2.31 1.54	1.48 1.31	1.58 1.31
H57	4.40 5.19	1.32 1.31	1.60 1.31
H58	2.52 1.74	1.94 1.54	2.50 1.54
H59	2.84 1.86	1.25 1.31	1.29 1.31
H60	1.97 1.54	1.48 1.31	1.24 1.31
H61	1.66 1.31	2.04 1.54	1.87 1.31
H62	2.00 1.54	2.48 1.74	2.60 1.74
H63	5.76 7.26	4.57 5.19	4.29 3.27
H64	1.29 1.31	1.39 1.31	1.45 1.31
H65	1.15 1.31	1.37 1.31	0.80 0.85
H66	0.92 1.04	1.05 1.04	1.19 1.31
H67	0.67 0.85	2.54 1.74	2.27 1.54
H68	1.10 1.31	2.98 1.86	3.08 1.74
H69	0.67 0.85	2.07 1.54	1.99 1.54
H70	0.61 0.85	2.34 1.54	2.37 1.54
H71	2.03 1.54	2.23 1.54	2.43 1.54
H72	0.92 1.04	6.09 7.26	6.06 7.26

Figure 192: Table displaying the DFT NMR shift predictions (PPM) for molecule NL2B at the three levels of theory tested and the corresponding experimental values (PPM) assigned by DP4-AI

5.32 NP1

	MM calc exp	Opt calc exp	Opt E calc exp
C1	27.58 23.37	26.69 24.82	26.69 24.82
C14	38.75 33.57	37.23 33.57	37.23 33.57
C16	16.20 12.53	14.20 12.53	14.20 12.53
C2	36.67 32.86	36.16 32.86	36.16 32.86
C20	184.49 172.01	178.57 172.01	178.57 172.01
C23	101.56 93.67	97.92 93.67	97.92 93.67
C26	29.42 25.17	28.51 25.17	28.51 25.17
C29	40.34 35.87	38.69 35.87	38.69 35.87
C3	41.54 37.49	41.49 37.49	41.49 37.49
C32	110.08 105.34	110.47 105.34	110.47 105.34
C35	27.60 24.82	26.34 23.37	26.34 23.37
C39	21.35 19.79	21.04 19.79	21.04 19.79
C4	53.93 50.03	53.98 50.03	53.98 50.03
C5	85.89 79.47	84.76 79.47	84.76 79.47
C6	49.39 44.93	48.95 44.93	48.95 44.93

	MM calc exp	Opt calc exp	Opt E calc exp
H10	1.71 1.79	1.70 1.79	1.70 1.79
H11	1.42 1.19	1.54 1.43	1.54 1.43
H12	1.63 1.43	1.49 1.43	1.49 1.43
H13	1.84 1.79	1.75 1.79	1.75 1.79
H15	3.26 3.38	3.25 3.38	3.25 3.38
H17	1.19 1.19	0.98 1.00	0.98 1.00
H18	1.58 1.43	1.70 1.43	1.70 1.43
H19	0.57 1.00	0.61 1.00	0.61 1.00
H25	5.78 5.85	5.84 5.85	5.84 5.85
H27	1.95 2.02	1.94 2.02	1.94 2.02
H28	1.69 1.43	1.59 1.43	1.59 1.43
H30	2.63 2.42	2.43 2.42	2.43 2.42
H31	2.11 2.02	1.99 2.02	1.99 2.02
H36	1.47 1.43	1.33 1.19	1.33 1.19
H37	1.45 1.43	1.43 1.43	1.43 1.43
H38	1.54 1.43	1.43 1.43	1.43 1.43
H40	1.32 1.19	1.36 1.19	1.36 1.19
H41	0.59 1.00	0.65 1.00	0.65 1.00
H42	0.99 1.00	1.02 1.00	1.02 1.00
H7	1.88 1.79	1.76 1.79	1.76 1.79
H8	0.97 1.00	1.12 1.19	1.12 1.19
H9	1.16 1.19	1.20 1.19	1.20 1.19

Figure 193: Table displaying the DFT NMR shift predictions (PPM) for molecule NP1 at the three levels of theory tested and the corresponding experimental values (PPM) assigned by DP4-AI

5.33 NP2

	MM	Opt	Opt E
	calc exp	calc exp	calc exp
C1	131.23 123.79	129.76 123.79	129.76 123.79
C10	58.06 53.86	58.08 53.86	58.08 53.86
C11	52.68 50.43	53.79 50.43	53.79 50.43
C12	47.89 42.76	48.05 42.76	48.05 42.76
C13	39.52 35.61	39.38 35.67	39.41 36.37
C14	24.44 20.59	24.22 20.59	24.22 20.59
C15	84.80 81.52	86.79 81.52	86.78 81.52
C16	33.40 30.36	33.65 30.36	33.72 30.36
C17	26.57 23.28	26.45 23.28	26.46 23.28
C19	12.94 11.00	11.60 11.00	11.58 11.00
C2	179.91 171.31	183.12 171.31	183.13 171.31
C21	19.35 17.36	18.74 17.36	18.74 17.36
C3	44.56 38.61	45.04 38.61	45.04 38.61
C4	40.07 36.37	39.03 35.61	39.03 35.61
C5	35.95 32.75	37.02 32.75	37.02 32.75
C6	205.61 199.57	203.59 199.57	203.59 199.57
C7	36.94 33.89	37.26 33.89	37.26 33.89
C8	35.17 31.49	35.14 31.49	35.14 31.49
C9	39.84 35.67	39.40 36.37	39.40 35.67

	MM	Opt	Opt E
	calc exp	calc exp	calc exp
H20	1.69 1.60	1.75 1.60	1.75 1.60
H22	1.18 0.98	1.10 0.98	1.10 0.98
H23	1.25 1.18	1.18 1.18	1.19 1.18
H25	5.86 5.71	5.74 5.71	5.74 5.71
H26	1.78 1.60	1.70 1.60	1.70 1.60
H27	2.06 2.03	2.00 2.03	2.00 2.03
H28	2.27 2.34	2.19 2.34	2.19 2.34
H29	2.48 2.34	2.47 2.34	2.47 2.34
H30	2.37 2.34	2.32 2.34	2.32 2.34
H31	2.44 2.34	2.59 2.34	2.59 2.34
H32	1.13 0.98	1.18 0.98	1.18 0.98
H33	1.86 1.84	1.85 1.84	1.85 1.84
H34	1.22 0.98	1.20 1.18	1.21 1.18
H35	1.83 1.84	1.87 1.84	1.88 1.84
H36	1.54 1.60	1.53 1.60	1.53 1.60
H37	1.51 1.43	1.53 1.43	1.53 1.43
H38	3.84 3.63	3.75 3.63	3.75 3.63
H39	2.16 2.03	2.09 2.03	2.09 2.03
H40	1.49 1.43	1.44 1.43	1.44 1.29
H41	1.66 1.60	1.64 1.60	1.64 1.60
H42	1.41 1.29	1.44 1.29	1.44 1.43
H43	0.48 0.77	0.40 0.77	0.40 0.77
H44	0.93 0.77	0.94 0.77	0.95 0.77
H45	0.73 0.77	0.64 0.77	0.64 0.77
H46	0.77 0.77	0.73 0.77	0.72 0.77
H47	1.22 1.18	1.16 0.98	1.16 0.98
H48	1.39 1.18	1.30 1.18	1.30 1.18
H49	1.28 1.18	1.22 1.18	1.23 1.18

Figure 194: Table displaying the DFT NMR shift predictions (PPM) for molecule NP2 at the three levels of theory tested and the corresponding experimental values (PPM) assigned by DP4-AI

5.34 NP3A

	MM		Opt		Opt E	
	calc	exp	calc	exp	calc	exp
C1	137.94	131.30	138.03	131.30	138.00	131.30
C10	157.18	148.08	155.86	148.08	155.72	148.08
C11	78.62	71.99	78.96	71.99	78.99	71.99
C12	65.32	59.98	65.29	59.98	65.21	59.98
C14	59.56	57.04	60.20	57.04	60.29	57.04
C15	45.77	39.99	45.81	39.99	45.23	39.99
C16	33.39	27.85	33.39	27.85	32.66	27.85
C17	21.86	21.85	22.48	21.85	22.51	21.85
C2	144.44	141.94	144.37	141.94	144.32	141.94
C20	30.52	27.68	30.79	27.68	30.86	27.68
C21	46.57	43.17	47.31	43.17	47.41	43.17
C22	151.70	144.02	151.77	144.02	151.52	144.02
C23	118.98	114.24	118.99	114.24	118.62	114.24
C3	128.31	126.62	128.17	126.62	128.20	126.62
C4	106.01	101.40	106.07	101.40	106.89	101.40
C45	55.53	55.62	55.48	55.62	55.44	55.62
C5	163.29	157.60	163.19	157.60	163.14	157.60
C6	127.49	121.34	127.16	121.34	126.29	121.34
C8	154.06	147.38	153.96	147.38	153.97	147.38
C9	121.72	118.43	122.19	118.43	122.29	118.43

	MM		Opt		Opt E	
	calc	exp	calc	exp	calc	exp
H18	3.26	3.41	3.21	3.41	3.18	3.06
H24	8.36	7.89	8.37	7.89	8.37	7.89
H25	7.09	7.22	7.06	7.22	7.09	7.22
H26	7.74	7.27	7.73	7.27	7.71	7.27
H27	9.07	8.48	9.07	8.48	9.07	8.48
H28	7.98	7.44	7.97	7.44	7.97	7.44
H29	5.99	5.46	5.99	5.46	5.98	5.46
H30	2.78	2.62	2.83	2.62	2.83	2.62
H31	3.23	3.06	3.18	3.06	3.19	3.41
H32	2.41	2.62	2.38	2.62	2.37	2.62
H33	1.76	1.74	1.76	1.74	1.80	1.74
H34	1.43	1.49	1.32	1.49	1.32	1.49
H35	2.17	2.23	2.11	2.23	2.09	2.23
H36	1.21	1.49	1.43	1.49	1.45	1.49
H37	1.89	1.74	1.85	1.74	1.85	1.74
H38	1.49	1.74	1.49	1.74	1.48	1.74
H39	2.79	3.06	2.84	3.06	2.85	3.06
H40	3.79	3.88	3.47	3.88	3.43	3.88
H41	6.19	5.72	6.13	5.72	6.12	5.72
H42	5.13	4.92	5.10	4.92	5.07	4.92
H43	5.02	4.92	4.99	4.92	4.99	4.92
H46	4.15	3.88	4.04	3.88	4.03	3.88
H47	3.87	3.88	3.84	3.88	3.85	3.88
H48	3.81	3.88	3.94	3.88	3.93	3.88

Figure 195: Table displaying the DFT NMR shift predictions (PPM) for molecule NP3A at the three levels of theory tested and the corresponding experimental values (PPM) assigned by DP4-AI

5.35 NP3B

	MM	Opt	Opt E
	calc exp	calc exp	calc exp
C1	137.94 131.31	138.02 131.31	138.05 131.31
C10	157.09 148.06	155.86 148.06	155.63 148.06
C11	78.73 71.92	79.04 71.92	79.06 71.92
C12	65.03 59.76	65.06 59.76	65.01 59.76
C14	53.62 50.12	54.18 50.12	53.90 50.12
C15	46.43 40.08	46.21 40.08	45.75 40.08
C16	34.34 28.20	34.29 28.20	33.90 28.20
C17	22.28 21.21	22.43 21.21	22.45 21.21
C2	144.38 140.69	144.32 140.69	144.34 140.69
C20	30.50 26.44	30.13 26.44	30.08 26.44
C21	52.28 49.61	52.84 49.61	52.94 49.61
C22	152.41 143.96	151.86 143.96	151.42 143.96
C23	119.19 114.39	119.47 114.39	119.27 114.39
C3	128.34 126.57	128.17 126.57	128.14 126.57
C4	106.30 101.27	106.12 101.27	105.53 101.27
C45	55.51 55.51	55.50 55.51	55.51 55.51
C5	163.25 157.52	163.17 157.52	163.18 157.52
C6	126.97 121.33	126.92 121.33	127.49 121.33
C8	154.11 147.40	153.99 147.40	153.95 147.40
C9	121.99 118.39	122.32 118.39	122.38 118.39

	MM	Opt	Opt E
	calc exp	calc exp	calc exp
H18	3.19 3.11	3.15 3.11	3.15 3.11
H24	8.35 8.00	8.35 8.00	8.35 8.00
H25	7.12 7.26	7.09 7.26	7.06 7.26
H26	7.76 7.35	7.75 7.35	7.76 7.35
H27	9.08 8.60	9.08 8.60	9.08 8.60
H28	7.99 7.57	7.97 7.57	7.97 7.57
H29	6.00 5.63	6.00 5.63	6.00 5.63
H30	3.52 3.39	3.23 3.39	3.20 3.39
H31	3.02 2.95	3.05 2.95	3.05 2.95
H32	2.46 2.11	2.43 2.11	2.43 2.29
H33	1.69 1.57	1.69 1.57	1.71 1.57
H34	2.47 2.29	2.44 2.29	2.41 2.11
H35	1.08 0.94	0.99 0.94	0.97 0.94
H36	1.45 1.22	1.55 1.57	1.57 1.57
H37	1.50 1.57	1.48 1.57	1.49 1.57
H38	1.51 1.57	1.48 1.22	1.47 1.22
H39	2.93 2.82	2.90 2.82	2.89 2.82
H40	3.04 2.95	3.05 2.95	3.05 2.95
H41	6.92 6.12	6.86 6.12	6.77 6.12
H42	5.23 5.11	5.24 5.11	5.24 5.11
H43	5.17 4.91	5.20 4.91	5.23 5.11
H46	3.81 3.94	3.80 3.94	3.79 3.94
H47	3.84 3.94	3.84 3.94	3.83 3.94
H48	4.15 3.94	4.15 3.94	4.17 3.94

Figure 196: Table displaying the DFT NMR shift predictions (PPM) for molecule NP3B at the three levels of theory tested and the corresponding experimental values (PPM) assigned by DP4-AI

5.36 NP4

	MM	Opt	Opt E
	calc exp	calc exp	calc exp
C1	135.69 130.06	136.63 130.06	136.63 130.06
C10	159.71 149.98	157.12 149.98	157.11 149.98
C11	78.55 71.69	78.86 71.69	78.86 71.69
C12	65.94 60.32	64.61 60.32	64.51 60.32
C14	59.66 56.93	60.13 56.93	59.79 56.93
C15	45.81 39.88	46.07 39.88	45.52 39.88
C16	33.51 27.87	34.38 27.87	33.87 27.87
C17	22.04 21.35	21.40 21.35	21.37 21.35
C2	147.33 141.76	153.70 148.01	153.69 148.01
C20	30.63 27.55	30.83 27.55	30.90 27.55
C21	46.60 43.16	46.86 43.16	46.94 43.16
C22	151.78 148.01	150.40 141.76	150.21 141.76
C23	119.02 114.29	117.60 114.29	117.34 114.29
C3	126.50 122.92	129.97 125.58	129.97 125.58
C4	129.15 125.58	128.15 122.92	128.15 122.92
C5	132.36 126.53	131.55 126.53	131.55 126.53
C6	135.50 128.96	134.31 128.96	134.30 128.96
C8	156.93 149.70	155.46 149.70	155.45 149.70
C9	121.81 118.21	121.32 118.21	121.33 118.21

	MM	Opt	Opt E
	calc exp	calc exp	calc exp
H18	3.23 3.48	3.17 3.48	3.15 3.48
H24	8.37 7.92	8.40 8.04	8.40 8.04
H25	8.29 7.57	8.26 7.92	8.26 7.92
H26	8.14 7.57	8.05 7.57	8.05 7.57
H27	9.24 8.72	9.17 8.72	9.17 8.72
H28	8.07 7.57	7.81 7.57	7.81 7.57
H29	6.14 5.65	6.02 5.65	6.01 5.65
H30	2.76 3.02	2.78 3.02	2.81 3.02
H31	3.24 8.04	3.18 4.89	3.17 4.89
H32	2.43 2.58	2.27 2.58	2.27 2.58
H33	1.77 1.76	1.74 1.76	1.77 1.76
H34	1.42 1.76	1.26 1.45	1.25 1.45
H35	2.19 2.58	2.13 2.58	2.12 2.58
H36	1.23 1.45	1.05 1.45	1.04 1.45
H37	1.92 1.45	1.83 1.76	1.84 1.76
H38	1.51 1.76	1.47 1.76	1.48 1.76
H39	2.82 3.02	2.74 3.02	2.74 3.02
H40	3.81 4.89	3.97 4.89	3.98 4.89
H41	6.17 5.65	6.06 7.29	6.06 7.29
H42	5.02 4.89	4.99 5.18	5.01 5.18
H43	5.12 5.18	5.08 5.65	5.06 5.65
H44	8.04 7.29	7.92 7.57	7.92 7.57

Figure 197: Table displaying the DFT NMR shift predictions (PPM) for molecule NP4 at the three levels of theory tested and the corresponding experimental values (PPM) assigned by DP4-AI

5.37 NP5

	MM		Opt		Opt E	
	calc	exp	calc	exp	calc	exp
C1	55.86	56.17	55.86	56.17	55.86	56.17
C10	54.66	53.54	54.61	53.54	54.67	53.54
C11	65.28	64.35	65.26	64.35	65.28	64.35
C13	37.37	33.02	37.28	33.02	37.40	33.02
C14	28.13	24.78	28.14	24.78	28.13	24.78
C15	29.17	25.90	29.10	25.90	29.19	25.90
C16	55.16	55.33	55.15	55.33	55.17	55.33
C17	30.95	27.58	30.96	27.58	30.95	27.58
C3	67.83	66.45	67.82	66.45	67.83	66.45
C4	31.64	29.25	31.65	29.25	31.64	29.25
C5	27.58	24.63	27.59	24.63	27.58	24.63
C6	28.90	25.79	28.90	25.79	28.90	25.79
C7	63.00	61.91	63.00	61.91	63.00	61.91
C8	41.07	36.06	41.07	36.06	41.07	36.06
C9	37.73	34.65	37.75	34.65	37.72	34.65

	MM		Opt		Opt E	
	calc	exp	calc	exp	calc	exp
H18	2.04	1.93	2.04	1.93	2.03	1.93
H19	1.77	1.65	1.77	1.65	1.77	1.65
H20	1.83	1.79	1.83	1.79	1.83	1.79
H21	2.67	2.64	2.67	2.64	2.67	2.64
H22	1.49	1.40	1.49	1.40	1.49	1.40
H23	1.23	1.40	1.23	1.40	1.23	1.40
H24	1.62	1.40	1.62	1.40	1.62	1.40
H25	1.29	1.40	1.29	1.40	1.29	1.40
H26	1.64	1.40	1.64	1.40	1.64	1.40
H27	1.45	1.40	1.45	1.40	1.46	1.40
H28	1.94	1.93	1.94	1.93	1.94	1.93
H29	2.60	2.47	2.60	2.47	2.60	2.47
H30	1.46	1.40	1.46	1.40	1.46	1.40
H31	1.92	1.93	1.91	1.93	1.92	1.93
H32	2.72	2.73	2.72	2.73	2.72	2.73
H33	2.22	2.29	2.23	2.29	2.22	2.29
H34	1.55	1.40	1.54	1.40	1.55	1.40
H35	1.45	1.40	1.45	1.40	1.44	1.40
H36	1.36	1.40	1.36	1.40	1.36	1.40
H37	1.65	1.65	1.65	1.65	1.65	1.65
H38	1.50	1.40	1.50	1.40	1.50	1.40
H39	1.67	1.65	1.67	1.65	1.67	1.65
H40	1.88	1.93	1.88	1.93	1.87	1.93
H41	2.66	2.64	2.67	2.64	2.66	2.64
H42	1.11	1.00	1.11	1.00	1.11	1.00
H43	2.14	1.93	2.14	1.93	2.14	1.93

Figure 198: Table displaying the DFT NMR shift predictions (PPM) for molecule NP5 at the three levels of theory tested and the corresponding experimental values (PPM) assigned by DP4-AI

5.38 OD1

	MM	Opt	Opt E
	calc exp	calc exp	calc exp
C10	51.95 51.36	53.44 51.36	53.05 51.36
C11	40.65 28.41	41.17 28.41	41.43 28.41
C12	36.32 28.41	37.52 28.41	36.49 28.41
C14	55.43 51.61	54.90 51.61	54.44 51.61
C16	69.52 63.32	67.83 63.32	68.26 63.32
C17	189.96 174.97	185.85 174.97	186.11 174.97
C2	152.04 141.55	159.81 141.55	159.95 141.55
C20	52.58 51.36	53.87 51.36	53.97 51.36
C21	43.84 28.41	44.69 28.41	44.62 28.41
C22	144.46 129.17	144.79 129.17	145.24 129.17
C23	135.09 129.17	135.86 129.17	136.43 129.17
C24	133.64 128.36	133.53 128.36	133.74 128.36
C25	131.36 126.70	131.96 128.36	132.28 128.36
C26	133.44 128.36	133.24 128.36	133.34 128.36
C27	134.58 128.36	134.33 129.17	134.59 129.17
C3	104.92 103.74	105.76 103.74	106.06 103.74
C4	143.75 129.17	146.62 141.55	146.65 141.55
C5	129.31 118.22	122.18 118.22	122.24 118.22
C6	165.12 154.52	165.31 154.52	165.62 154.52
C7	45.53 39.76	45.69 39.76	46.03 39.76
C8	52.82 51.61	54.50 51.61	54.51 51.61

	MM	Opt	Opt E
	calc exp	calc exp	calc exp
H28	6.93 6.02	7.36 6.02	7.40 6.41
H29	5.73 3.63	5.83 3.63	5.84 3.63
H30	7.11 6.41	7.39 7.25	7.39 6.02
H31	6.39 3.63	6.22 3.63	6.23 3.63
H32	2.66 1.46	2.62 1.46	2.59 1.46
H33	2.61 1.46	2.61 1.46	2.37 1.46
H34	3.05 2.29	3.06 2.61	2.94 2.29
H35	0.87 1.27	0.98 1.27	0.89 1.27
H36	2.33 1.46	2.32 1.46	2.17 1.46
H37	2.88 1.46	2.87 1.46	2.76 1.46
H38	1.74 1.46	1.74 1.46	1.64 1.46
H39	1.11 1.27	1.09 1.46	1.33 1.46
H40	2.36 1.46	2.33 1.46	2.00 1.46
H41	2.13 1.46	2.04 1.46	2.22 1.46
H42	2.72 1.46	2.76 1.46	2.71 1.46
H43	1.30 1.46	0.95 1.27	1.06 1.27
H44	3.44 2.61	3.28 2.61	3.28 2.61
H45	3.68 3.63	3.68 3.63	3.72 3.63
H46	3.55 2.92	3.55 2.92	3.57 2.92
H47	3.66 3.45	3.65 3.45	3.65 3.45
H48	3.20 2.61	2.94 2.11	2.87 2.11
H49	3.04 2.11	2.98 2.29	3.00 2.61
H50	7.53 7.25	7.48 7.25	7.55 7.25
H51	7.57 7.36	7.71 7.36	7.94 7.36
H52	7.44 7.25	7.48 7.25	7.55 7.25
H53	7.49 7.25	7.51 7.25	7.56 7.25
H54	7.42 7.25	7.39 6.41	7.44 7.25

Figure 199: Table displaying the DFT NMR shift predictions (PPM) for molecule OD1 at the three levels of theory tested and the corresponding experimental values (PPM) assigned by DP4-AI

5.39 TP1

	MM		Opt		Opt E	
	calc	exp	calc	exp	calc	exp
C1	11.67	10.18	10.72	10.18	10.83	10.18
C10	38.85	34.65	38.54	34.65	38.26	34.65
C11	73.68	71.80	73.14	69.96	72.90	69.96
C13	75.39	73.01	75.75	73.01	75.72	73.01
C14	138.05	130.22	136.01	130.22	135.52	129.18
C15	134.86	129.18	135.56	129.18	136.01	129.18
C16	115.85	113.75	115.92	113.75	116.31	113.75
C17	163.87	159.16	166.23	159.16	166.41	159.16
C18	119.54	113.75	118.38	113.75	118.28	113.75
C19	134.98	129.18	135.68	129.18	136.06	130.22
C2	31.15	27.91	31.35	27.91	31.44	27.91
C21	54.93	55.24	55.40	55.24	55.50	55.24
C22	19.30	16.19	18.57	16.19	18.34	16.19
C23	10.32	9.19	10.24	9.19	10.82	9.19
C3	187.16	174.55	184.56	174.55	183.61	174.55
C6	72.50	69.96	75.59	71.80	75.37	71.80
C7	20.46	18.14	19.34	18.14	18.65	18.14
C8	48.25	41.59	45.86	41.59	45.57	41.59
C9	78.87	76.53	78.79	76.53	79.58	76.53

	MM		Opt		Opt E	
	calc	exp	calc	exp	calc	exp
H25	1.02	1.10	0.96	1.10	1.00	1.10
H26	1.20	1.10	1.17	1.10	1.14	1.10
H27	1.18	1.10	1.12	1.10	1.13	1.10
H28	2.48	2.30	2.41	2.30	2.40	2.30
H29	2.44	2.30	2.37	2.30	2.34	2.30
H30	5.41	5.40	5.47	5.40	5.50	5.40
H31	0.83	0.89	0.80	0.89	0.80	0.89
H32	1.38	1.22	1.44	1.22	1.37	1.22
H33	1.47	1.22	1.39	1.22	1.44	1.22
H34	1.82	1.65	1.82	1.65	1.62	1.22
H35	3.19	3.18	3.17	3.18	3.28	3.18
H36	2.09	1.99	2.05	1.99	1.92	1.99
H37	3.43	3.18	3.38	3.51	3.43	3.51
H38	3.57	3.51	3.62	3.51	3.45	3.51
H39	4.37	4.41	4.42	4.41	4.56	4.41
H40	4.37	4.41	4.40	4.41	4.35	4.41
H41	7.53	7.23	7.48	7.23	7.47	7.23
H42	6.85	6.87	6.84	6.87	6.87	6.87
H43	6.99	6.87	6.94	6.87	6.96	6.87
H44	7.59	7.23	7.53	7.23	7.51	7.23
H45	3.65	3.80	3.67	3.80	3.67	3.80
H46	3.85	3.80	3.90	3.80	3.85	3.80
H47	3.67	3.80	3.70	3.80	3.77	3.80
H48	0.85	0.89	0.86	1.10	0.91	1.10
H49	1.59	1.22	1.55	1.22	1.64	1.65
H50	1.07	1.10	1.01	1.10	0.99	1.10
H51	0.53	0.89	0.55	0.89	0.62	0.89
H52	1.00	1.10	1.00	1.10	0.96	1.10
H53	0.91	1.10	0.86	0.89	0.72	0.89
H54	3.57	3.51	3.05	3.18	2.66	3.18

Figure 200: Table displaying the DFT NMR shift predictions (PPM) for molecule TP1 at the three levels of theory tested and the corresponding experimental values (PPM) assigned by DP4-AI

5.40 TP2

	MM calc exp	Opt calc exp	Opt E calc exp
C1	11.31 7.01	10.33 7.01	10.38 7.01
C10	42.09 36.64	43.54 36.64	42.61 36.64
C11	74.44 71.58	73.41 70.65	72.69 70.65
C13	75.11 72.64	75.76 72.64	75.80 72.64
C14	138.88 130.85	136.56 130.85	136.66 130.85
C15	135.05 129.01	135.61 129.01	135.42 129.01
C16	119.56 113.64	118.57 113.64	117.79 113.64
C17	163.76 159.00	166.15 159.00	166.22 159.00
C18	115.64 113.64	115.72 113.64	116.59 113.64
C19	134.95 129.01	135.47 129.01	135.33 129.01
C2	30.94 27.97	30.91 27.97	30.99 27.97
C21	54.94 55.20	55.47 55.20	55.41 55.20
C22	11.69 9.13	11.27 9.13	11.41 9.13
C23	17.34 10.65	14.39 10.65	15.57 10.65
C26	9.86 7.01	8.76 5.34	8.44 5.34
C27	10.49 7.01	9.77 7.01	9.01 5.34
C28	9.86 7.01	8.83 5.34	9.52 7.01
C3	185.30 173.87	183.42 173.87	183.36 173.87
C58	9.41 5.34	9.15 5.34	9.15 5.34
C6	73.95 70.65	75.52 71.58	75.24 71.58
C64	9.28 5.34	9.25 7.01	9.26 7.01
C7	19.81 18.68	18.50 18.68	18.73 18.68
C70	9.49 5.34	9.37 7.01	9.37 7.01
C8	49.71 42.73	46.79 42.73	46.82 42.73
C9	79.80 76.82	77.93 76.82	78.94 76.82

	MM calc exp	Opt calc exp	Opt E calc exp
H29	1.09 0.94	1.12 0.94	1.14 0.94
H30	1.22 1.17	1.15 1.17	1.15 1.17
H31	0.87 0.94	0.88 0.94	0.88 0.94
H32	2.37 2.28	2.44 2.28	2.44 2.28
H33	2.35 2.28	2.36 2.28	2.36 2.28
H34	4.97 5.07	5.37 5.07	5.35 5.07
H35	1.52 1.17	1.50 1.17	1.47 1.17
H36	1.05 0.94	1.22 1.17	1.22 1.17
H37	0.93 0.94	0.78 0.94	0.80 0.94
H38	1.89 1.68	1.69 1.68	1.69 1.68
H39	3.98 3.80	3.96 3.80	3.87 3.80
H40	2.05 2.03	2.12 2.03	2.08 2.03
H41	3.42 3.26	3.40 3.55	3.31 3.26
H42	3.51 3.55	3.32 3.26	3.38 3.55
H43	4.54 4.41	4.63 4.41	4.57 4.41
H44	4.38 4.41	4.43 4.41	4.57 4.41
H45	7.57 7.25	7.52 7.25	7.52 7.25
H46	6.98 6.87	6.96 6.87	6.93 6.87
H47	6.87 6.87	6.87 6.87	6.90 6.87
H48	7.66 7.25	7.56 7.25	7.55 7.25
H49	3.68 3.80	3.70 3.80	3.67 3.80
H50	3.75 3.80	3.82 3.80	3.89 3.80
H51	3.74 3.80	3.76 3.80	3.71 3.80
H52	1.08 0.94	0.94 0.94	0.97 0.94
H53	0.85 0.94	0.92 0.94	0.93 0.94
H54	0.87 0.94	0.81 0.94	0.81 0.94
H55	1.29 1.17	1.11 0.94	1.22 1.17
H56	0.98 0.94	0.88 0.94	0.93 0.94
H57	1.03 0.94	1.13 1.17	1.00 0.94
H59	0.61 0.60	0.65 0.60	0.60 0.60
H60	0.59 0.60	0.62 0.60	0.62 0.60
H61	0.93 0.94	0.91 0.94	0.83 0.94
H62	0.97 0.94	0.96 0.94	0.87 0.94
H63	1.23 1.17	1.16 1.17	1.14 1.17
H65	0.49 0.60	0.55 0.60	0.53 0.60
H66	0.60 0.60	0.61 0.60	0.64 0.60
H67	0.78 0.94	0.78 0.94	0.76 0.94
H68	0.84 0.94	0.85 0.94	0.82 0.94
H69	1.16 1.17	1.11 0.94	1.08 0.94
H71	0.65 0.60	0.66 0.60	0.63 0.60
H72	0.60 0.60	0.65 0.60	0.66 0.60
H73	1.22 1.17	1.16 1.17	1.16 1.17
H74	1.00 0.94	1.03 0.94	1.02 0.94
H75	1.03 0.94	0.99 0.94	0.93 0.94

Figure 201: Table displaying the DFT NMR shift predictions (PPM) for molecule TP2 at the three levels of theory tested and the corresponding experimental values (PPM) assigned by DP4-AI

5.41 TP3

	MM	Opt	Opt E
	calc exp	calc exp	calc exp
C1	11.68 10.32	10.75 9.19	10.78 9.19
C10	40.42 35.76	39.77 35.76	39.73 35.76
C11	73.48 71.25	72.33 69.65	71.82 69.65
C13	75.20 72.63	75.61 72.63	75.41 72.63
C14	138.58 130.71	136.41 130.71	136.49 130.71
C15	134.63 128.92	135.54 128.92	135.57 128.92
C16	120.46 113.58	119.59 113.58	118.80 113.58
C17	163.70 158.93	166.09 158.93	166.10 158.93
C18	114.63 113.58	114.51 113.58	115.34 113.58
C19	134.67 128.92	135.45 128.92	135.53 128.92
C2	31.34 27.93	31.43 27.93	31.55 27.93
C21	54.94 55.09	55.40 55.09	55.41 55.09
C22	11.40 9.19	11.21 10.32	11.49 10.32
C23	18.69 16.19	17.57 16.19	17.23 16.19
C25	62.01 61.28	61.76 61.28	61.68 61.28
C3	185.41 173.83	182.93 173.83	182.67 173.83
C6	71.44 69.65	73.31 71.25	72.76 71.25
C7	19.75 18.23	19.05 18.23	19.29 18.23
C8	47.03 40.84	44.74 40.84	44.41 40.84
C9	88.62 85.86	86.97 85.86	87.09 85.86

	MM	Opt	Opt E
	calc exp	calc exp	calc exp
H26	0.96 1.08	0.91 1.08	0.91 1.08
H27	1.21 1.22	1.16 1.22	1.13 1.15
H28	1.17 1.15	1.15 1.15	1.18 1.15
H29	2.41 2.32	2.37 2.32	2.36 2.32
H30	2.39 2.32	2.34 2.32	2.32 2.32
H31	5.29 5.25	5.46 5.25	5.43 5.25
H32	1.58 1.22	1.38 1.22	1.34 1.22
H33	1.19 1.15	1.33 1.22	1.36 1.22
H34	0.77 0.94	0.78 0.94	0.78 0.94
H35	1.85 1.71	1.75 1.71	1.68 1.71
H36	3.18 2.91	3.03 2.91	3.07 2.91
H37	2.15 2.10	2.13 2.10	2.11 2.10
H38	3.48 3.55	3.50 3.55	3.37 3.55
H39	3.32 3.37	3.28 3.37	3.31 3.37
H40	4.45 4.42	4.44 4.42	4.47 4.42
H41	4.36 4.42	4.45 4.42	4.52 4.42
H42	7.62 7.25	7.55 7.25	7.54 7.25
H43	7.02 6.88	6.99 6.88	6.96 6.88
H44	6.81 6.88	6.80 6.88	6.83 6.88
H45	7.54 7.25	7.48 7.25	7.48 7.25
H46	3.73 3.80	3.76 3.80	3.79 3.80
H47	3.67 3.80	3.72 3.80	3.72 3.80
H48	3.75 3.80	3.78 3.80	3.76 3.80
H49	0.97 1.08	0.97 1.08	0.90 0.94
H50	1.08 1.08	1.06 1.08	1.10 1.15
H51	0.81 0.94	0.83 0.94	0.96 1.08
H52	1.14 1.15	1.08 1.15	1.07 1.08
H53	0.91 0.94	0.87 0.94	0.84 0.94
H54	1.32 1.22	1.15 1.15	1.19 1.22
H55	3.29 3.37	3.30 3.37	3.29 3.37
H56	3.19 3.37	3.25 3.37	3.23 3.37
H57	3.19 3.37	3.31 3.37	3.27 3.37

Figure 202: Table displaying the DFT NMR shift predictions (PPM) for molecule TP3 at the three levels of theory tested and the corresponding experimental values (PPM) assigned by DP4-AI

5.42 TP4

	MM	Opt	Opt E
	calc exp	calc exp	calc exp
C1	11.81 10.34	10.91 10.34	10.97 10.34
C10	38.58 35.98	38.78 35.98	38.91 35.98
C11	65.99 64.55	66.14 64.55	66.11 64.55
C13	11.07 9.23	10.84 9.23	10.90 9.23
C14	18.36 16.04	17.95 16.04	17.87 16.04
C16	62.44 61.70	62.25 61.70	62.23 61.70
C2	31.53 27.98	31.34 27.98	31.45 27.98
C3	185.46 173.92	183.16 173.92	183.12 173.92
C6	71.63 69.60	73.30 69.60	73.35 69.60
C7	19.68 18.33	18.89 18.33	18.84 18.33
C8	47.45 41.32	44.89 41.32	44.83 41.32
C9	91.10 88.56	90.78 88.56	90.58 88.56

	MM	Opt	Opt E
	calc exp	calc exp	calc exp
H17	1.03 1.16	1.07 1.16	1.07 1.16
H18	1.19 1.16	1.06 1.16	1.08 1.16
H19	1.20 1.16	1.16 1.16	1.16 1.16
H20	2.41 2.30	2.37 2.30	2.37 2.67
H21	2.41 2.30	2.37 2.67	2.36 2.30
H22	5.37 5.22	5.56 5.22	5.55 5.22
H23	0.96 1.16	0.92 0.90	0.94 0.90
H24	1.20 1.16	1.34 1.16	1.32 1.16
H25	1.62 1.16	1.46 1.16	1.45 1.16
H26	1.97 1.82	1.85 1.82	1.84 1.82
H27	3.32 3.40	3.12 2.96	3.13 2.96
H28	1.64 1.16	1.58 1.16	1.59 1.16
H29	4.13 3.78	4.17 3.78	4.14 3.78
H30	3.45 3.40	3.49 3.40	3.50 3.40
H31	2.68 2.67	2.14 2.30	2.06 2.30
H32	0.69 0.90	0.69 0.90	0.70 0.90
H33	1.16 1.16	1.14 1.16	1.13 1.16
H34	0.69 0.90	0.65 0.90	0.66 0.90
H35	0.95 0.90	0.94 1.16	0.95 1.16
H36	1.84 1.82	1.77 1.82	1.70 1.82
H37	1.20 1.16	1.21 1.16	1.24 1.16
H38	3.33 3.40	3.40 3.40	3.40 3.40
H39	3.04 3.40	3.29 3.40	3.29 3.40
H40	3.48 3.53	3.36 3.40	3.36 3.40

Figure 203: Table displaying the DFT NMR shift predictions (PPM) for molecule TP4 at the three levels of theory tested and the corresponding experimental values (PPM) assigned by DP4-AI

5.43 TS1

	MM calc exp	Opt calc exp	Opt E calc exp
C1	136.62 129.28	136.41 129.28	136.89 129.28
C10	50.49 46.55	50.57 46.55	51.01 46.55
C11	14.96 13.20	14.06 13.20	14.84 11.37
C12		233.01 214.02	231.93 214.02
C13	53.58 46.81	52.15 46.81	51.23 46.81
C15	74.23 68.83	73.90 68.83	75.15 71.86
C16	42.86 38.02	42.30 38.02	42.61 38.02
C17	14.90 11.37	13.16 11.37	14.84 13.20
C18	75.01 71.86	75.01 71.86	73.68 68.83
C2	136.96 129.28	135.06 128.38	135.03 128.38
C20	75.79 73.28	76.15 73.28	75.81 72.94
C21	131.96 127.61	132.67 127.61	132.58 127.61
C22	133.22 127.61	133.40 128.38	133.32 128.38
C23	133.29 127.61	133.24 127.61	133.27 127.61
C24	146.19 129.93	145.88 129.93	145.88 129.93
C25	133.40 128.38	133.26 127.61	133.27 127.61
C26	133.35 128.38	133.39 128.38	133.30 128.38
C28	55.06 55.24	55.55 55.24	55.56 55.24
C3	136.24 128.38	136.01 129.28	136.39 129.28
C4	118.45 113.78	117.66 113.78	118.65 113.78
C5	164.35 159.23	166.36 159.23	166.55 159.23
C6	118.11 113.78	117.18 113.78	116.34 113.78
C7	75.47 72.94	75.98 72.94	76.22 73.28
C9	78.12 73.53	77.70 73.53	77.93 73.53

	MM calc exp	Opt calc exp	Opt E calc exp
H30	7.54 7.27	7.46 7.20	7.53 7.27
H31	7.55 7.27	7.42 6.86	7.45 7.20
H32	6.79 6.86	6.81 4.49	6.85 4.49
H33	6.73 4.49	6.82 6.86	6.85 6.86
H34	4.41 4.40	4.39 4.40	4.36 4.40
H35	4.15 3.79	4.30 4.24	4.29 4.24
H36	3.55 3.79	3.54 3.46	3.58 3.46
H37	3.54 3.57	3.47 3.46	3.65 3.57
H38	3.30 3.46	3.12 3.14	3.16 2.87
H39	0.76 0.93	0.64 0.93	0.77 0.93
H40	1.20 1.05	1.17 1.05	1.11 1.05
H41	0.97 0.93	0.92 0.93	1.03 1.05
H42	2.93 2.63	2.92 2.63	2.86 2.63
H43	2.46 2.63	2.64 2.63	2.46 2.05
H44	4.24 4.24	4.19 3.79	4.03 3.79
H45	1.67 1.55	1.70 1.55	1.52 1.55
H46	0.98 1.05	0.94 1.05	0.94 0.93
H47	1.07 1.05	1.02 1.05	1.01 1.05
H48	0.59 0.93	0.65 0.93	0.55 0.93
H49	3.31 3.46	3.41 3.46	3.17 3.14
H50	3.34 3.46	3.41 3.46	3.33 3.46
H51	4.44 4.40	4.56 4.49	4.50 4.40
H52	4.19 3.79	4.46 4.40	4.24 3.79
H53	7.44 6.86	7.51 7.20	7.46 7.27
H54	7.52 7.20	7.60 7.27	7.54 7.27
H55	7.50 7.20	7.56 7.27	7.44 6.86
H56	7.61 7.27	7.63 7.27	7.45 7.20
H57	7.54 7.27	7.60 7.27	7.48 7.27
H58	3.28 3.14	3.62 3.79	3.63 3.46
H59	3.27 2.87	3.60 3.57	3.56 3.46
H60	3.66 3.79	3.89 3.79	3.91 3.79
H61	3.39 3.46	3.06 2.87	2.82 2.63

Figure 204: Table displaying the DFT NMR shift predictions (PPM) for molecule TS1 at the three levels of theory tested and the corresponding experimental values (PPM) assigned by DP4-AI

5.44 TS2

	MM		Opt		Opt E	
	calc	exp	calc	exp	calc	exp
C1	135.39	128.39	135.93	129.32	135.44	128.39
C10	45.37	40.31	45.79	40.31	45.09	40.31
C11	17.79	15.61	17.14	15.61	16.36	15.61
C12	176.33	165.66	176.14	165.66	174.24	165.66
C13	33.48	29.21	32.08	29.21	33.03	29.21
C14	81.53	77.78	81.09	77.78	81.99	77.78
C15	42.55	37.49	42.43	37.49	42.94	37.49
C16	15.14	11.73	14.64	11.73	16.04	11.73
C17	74.06	71.57	72.57	71.57	71.53	72.17
C19	75.90	73.28	76.26	73.28	76.26	73.28
C2	137.78	129.32	135.68	128.39	136.54	129.32
C20	132.29	127.68	133.09	127.68	133.29	127.68
C21	133.25	127.68	133.37	127.68	133.17	127.68
C22	133.90	128.39	134.35	128.39	135.36	128.39
C23	145.88	129.87	145.66	129.87	145.02	129.87
C24	133.61	127.68	134.12	128.39	135.31	128.39
C25	133.64	128.39	133.44	127.68	133.16	127.68
C27	55.07	55.25	55.71	55.25	55.63	55.25
C29	122.61	113.83	121.69	113.83	122.35	113.83
C3	135.95	129.32	136.14	129.32	135.76	129.32
C30	171.93	163.47	170.06	163.47	169.82	163.47
C4	115.87	113.83	115.11	113.83	115.00	113.83
C5	164.01	159.28	166.33	159.28	166.59	159.28
C6	120.95	113.83	120.06	113.83	121.54	113.83
C7	74.57	72.17	75.17	72.80	74.88	72.80
C9	75.07	72.80	73.24	72.17	70.72	71.57

	MM		Opt		Opt E	
	calc	exp	calc	exp	calc	exp
H32	7.46	7.20	7.38	7.20	7.43	7.28
H33	7.51	7.28	7.38	7.20	7.54	7.28
H34	6.49	6.87	6.54	6.87	6.88	6.87
H35	6.86	6.87	6.85	6.87	7.00	7.20
H36	4.33	4.45	4.44	4.45	4.28	4.45
H37	4.47	4.45	4.45	4.45	4.91	4.45
H38	3.43	3.53	3.35	3.41	3.37	3.41
H39	3.33	3.41	3.20	2.63	3.32	3.41
H40	2.49	2.38	2.48	2.38	2.14	2.10
H41	1.10	1.02	1.04	1.02	1.00	1.02
H42	1.61	1.09	1.50	1.09	1.79	2.10
H43	1.14	1.02	1.08	1.02	1.03	1.02
H44	2.05	2.10	1.95	2.10	1.67	1.09
H45	2.14	2.10	1.96	2.10	1.76	1.55
H46	4.67	4.45	4.50	4.45	4.20	4.45
H47	1.90	1.55	1.77	1.55	1.67	1.09
H48	1.21	1.09	1.26	1.09	1.28	1.09
H49	0.78	1.02	0.83	1.02	0.87	1.02
H50	1.38	1.09	1.49	1.09	1.68	1.26
H51	3.23	3.41	3.22	3.41	3.05	3.41
H52	3.43	3.41	3.33	3.41	3.02	2.63
H53	4.30	4.45	4.61	4.45	4.72	4.45
H54	4.34	4.45	4.31	4.45	4.10	3.79
H55	7.46	7.20	7.50	7.28	7.50	7.28
H56	7.58	7.28	7.55	7.28	7.49	7.28
H57	7.55	7.28	7.45	7.28	7.25	7.20
H58	7.48	7.28	7.46	7.28	6.81	6.87
H59	7.53	7.28	7.53	7.28	7.42	7.28
H60	3.01	2.63	3.50	3.53	3.65	3.79
H61	3.48	3.79	3.67	3.79	3.74	3.79
H62	3.47	3.79	3.76	3.79	3.86	3.79
H63	5.58	5.81	5.61	5.81	5.68	5.81

Figure 205: Table displaying the DFT NMR shift predictions (PPM) for molecule TS2 at the three levels of theory tested and the corresponding experimental values (PPM) assigned by DP4-AI

5.45 TS3A

	MM		Opt		Opt E	
	calc	exp	calc	exp	calc	exp
H33	7.67	7.26	7.65	7.26	7.48	7.26
H34	7.32	7.26	7.28	7.26	7.10	7.26
H35	6.97	6.88	6.86	6.88	6.58	6.88
H36	7.04	6.88	6.99	6.88	6.87	6.88
H37	4.03	4.30	4.29	4.30	4.59	4.46
H38	4.65	4.46	4.53	4.46	4.19	3.81
H39	3.04	2.58	3.23	3.43	3.15	3.43
H40	3.40	3.43	3.49	3.68	3.32	3.43
H41	1.13	1.55	1.69	1.79	1.92	1.93
H42	0.42	1.01	0.72	1.01	0.74	1.01
H43	1.11	1.26	1.09	1.01	0.69	1.01
H44	0.36	1.01	0.91	1.01	0.94	1.01
H45	1.02	1.01	1.56	1.55	1.39	1.01
H46	1.33	1.55	1.55	1.55	0.95	1.01
H47	3.94	3.81	4.42	4.46	4.09	3.81
H48	1.81	1.79	1.92	1.93	1.75	1.79
H49	1.61	1.55	1.50	1.01	1.60	1.26
H50	0.94	1.01	0.83	1.01	0.93	1.01
H51	1.29	1.55	1.23	1.01	1.26	1.01
H52	3.36	3.43	3.29	3.50	3.12	2.58
H53	3.47	3.50	3.20	3.43	3.03	2.58
H54	4.71	4.46	4.78	4.46	4.89	4.46
H55	4.00	3.81	4.45	4.46	4.26	4.30
H56	7.50	7.26	7.55	7.26	7.60	7.26
H57	7.54	7.26	7.58	7.26	7.59	7.26
H58	7.61	7.26	7.52	7.26	7.58	7.26
H59	7.47	7.26	7.53	7.26	7.45	7.26
H60	7.57	7.26	7.61	7.26	7.62	7.26
H61	3.69	3.81	3.70	3.81	3.56	3.81
H62	3.67	3.81	3.65	3.81	3.56	3.81
H63	3.90	3.81	3.94	3.81	3.86	3.81
H64	2.24	2.05	2.28	2.05	2.24	1.93
H65	1.88	1.93	2.23	1.93	2.30	2.05
H66	3.64	3.68	3.13	2.58	3.49	3.50

Figure 206: Table displaying the DFT NMR shift predictions (PPM) for molecule TS3A at the three levels of theory tested and the corresponding experimental values (PPM) assigned by DP4-AI

5.46 TS3B

	MM		Opt		Opt E	
	calc	exp	calc	exp	calc	exp
C1	135.75	128.37	136.77	129.54	136.79	129.54
C12	76.58	76.20	77.32	76.20	77.45	76.20
C14	77.09	76.28	80.01	76.28	79.13	76.28
C17	75.36	75.98	73.99	75.90	73.55	75.90
C19	75.68	76.11	76.18	76.11	76.16	76.11
C2	136.27	129.54	133.98	128.37	133.78	128.37
C20	132.23	127.64	132.82	127.64	132.99	127.64
C21	133.56	128.37	133.48	128.37	133.65	128.37
C22	134.36	128.37	133.68	128.37	134.16	128.37
C23	146.35	129.54	145.99	129.54	145.99	129.54
C24	133.38	127.64	132.92	127.64	133.06	127.64
C25	133.17	127.64	133.32	127.64	133.32	127.64
C3	136.57	129.54	137.36	129.54	137.54	129.54
C4	117.03	114.00	116.93	114.00	116.88	114.00
C6	120.59	114.00	119.04	114.00	119.10	114.00
C7	74.51	75.90	75.10	75.98	74.97	75.98
C9	74.25	75.78	72.20	75.78	71.83	75.78

	MM		Opt		Opt E	
	calc	exp	calc	exp	calc	exp
H33	7.32	7.27	7.32	7.27	7.29	7.27
H34	7.57	7.27	7.54	7.27	7.42	7.27
H35	6.69	6.88	6.77	6.88	6.69	6.88
H36	6.90	6.88	6.92	6.88	6.90	6.88
H37	3.76	3.80	3.96	3.80	3.97	3.80
H38	4.83	4.46	4.82	4.46	4.84	4.46
H39	3.35	3.43	3.32	3.43	3.34	3.43
H40	3.73	3.80	3.69	3.80	3.68	3.80
H41	1.30	1.26	1.25	1.26	1.25	1.26
H42	0.84	0.96	0.82	0.96	0.84	0.96
H43	1.80	1.55	1.71	1.55	1.71	1.55
H44	1.16	0.96	1.16	0.96	1.16	0.96
H45	0.78	0.96	0.83	0.96	0.68	0.96
H46	1.25	0.96	1.18	0.96	1.09	0.96
H47	4.99	4.88	5.01	4.88	5.06	4.88
H48	1.54	1.55	1.59	1.55	1.48	1.55
H49	0.86	0.96	0.89	0.96	0.88	0.96
H50	0.81	0.96	0.83	0.96	0.73	0.96
H51	0.87	0.96	0.88	0.96	0.90	0.96
H52	3.62	3.80	3.60	3.60	3.60	3.60
H53	3.40	3.60	3.44	3.60	3.37	3.60
H54	4.55	4.46	4.62	4.46	4.66	4.46
H55	4.45	4.46	4.62	4.46	4.61	4.46
H56	7.52	7.27	7.57	7.27	7.58	7.27
H57	7.70	7.27	7.66	7.27	7.71	7.27
H58	7.90	7.27	7.73	7.27	7.85	7.27
H59	7.51	7.27	7.56	7.27	7.52	7.27
H60	7.58	7.27	7.62	7.27	7.62	7.27
H61	3.01	3.43	3.39	3.43	3.30	3.43
H62	3.56	3.60	3.77	3.80	3.77	3.80
H63	3.56	3.80	3.86	3.80	3.83	3.80
H64	2.10	2.01	1.94	2.01	1.99	2.01
H65	2.78	2.57	2.57	2.39	2.56	2.39
H66	3.86	4.14	2.81	2.57	2.80	2.57

Figure 207: Table displaying the DFT NMR shift predictions (PPM) for molecule TS3B at the three levels of theory tested and the corresponding experimental values (PPM) assigned by DP4-AI

5.47 TS4

	MM		Opt		Opt E	
	calc	exp	calc	exp	calc	exp
H31	2.25	1.04	2.15	1.04	2.19	1.04
H33	3.54	3.79	3.75	3.50	3.75	3.50
H34	2.99	1.54	3.23	2.62	3.23	1.54
H35	3.53	3.50	3.77	3.79	3.77	3.79
H36	6.73	4.43	6.70	4.43	6.71	4.43
H37	7.73	7.27	7.63	7.27	7.67	7.27
H38	7.54	7.27	7.36	6.85	7.48	6.85
H39	6.91	6.85	6.91	6.85	6.93	6.85
H40	4.03	3.79	4.15	3.79	4.14	3.79
H41	4.51	4.43	4.78	4.43	4.68	4.43
H42	3.49	3.50	3.32	3.27	3.43	3.27
H43	3.32	2.79	3.22	1.54	3.32	2.79
H44	1.34	0.83	1.18	0.83	1.25	0.83
H45	2.08	1.04	1.89	1.04	1.93	1.04
H46	2.67	1.04	2.51	1.04	2.55	1.04
H47	1.33	0.83	0.92	0.83	1.14	0.83
H48	1.02	0.83	0.80	0.83	0.88	0.83
H49	4.43	4.43	4.72	4.43	4.67	4.43
H50	1.75	0.83	1.60	0.83	1.67	0.83
H51	3.26	2.62	3.30	2.79	3.30	2.62
H52	3.46	3.27	3.60	3.50	3.54	3.50
H53	4.22	4.43	4.66	4.43	4.40	3.79
H54	4.09	3.79	4.54	3.79	4.42	4.43
H55	7.52	7.27	7.60	7.27	7.59	7.27
H56	7.57	7.27	7.62	7.27	7.61	7.27
H57	7.51	6.85	7.59	7.27	7.58	7.27
H58	7.65	7.27	7.68	7.27	7.66	7.27
H59	7.75	7.27	7.72	7.27	7.69	7.27
H60	0.96	0.83	0.86	0.83	0.87	0.01
H61	0.77	0.83	0.61	0.83	0.67	0.01
H62	0.84	0.83	0.90	0.83	0.84	0.01
H63	1.04	0.83	1.06	0.83	1.04	0.83
H64	1.34	0.83	1.17	0.83	1.26	0.83
H65	1.00	0.83	0.93	0.83	0.97	0.83

Figure 208: Table displaying the DFT NMR shift predictions (PPM) for molecule TS4 at the three levels of theory tested and the corresponding experimental values (PPM) assigned by DP4-AI

References

[1] J. J. Helmus and C. P. Jaroniec, *Journal of Biomolecular NMR*, 2013, **55**, 355–367.

[2] L. Chen, Z. Weng, L. Goh and M. Garland, *Journal of Magnetic Resonance*, 2002, **158**, 164–168.

[3] V. Zorin, M. A. Bernstein and C. Cobas, *Magnetic Resonance in Chemistry*, 2017, **55**, 738–746.

[4] A. Heuer and U. Haeberlen, *Journal of Magnetic Resonance (1969)*, 1989, **85**, 79–94.

[5] G. A. Pearson, *Journal of Magnetic Resonance (1969)*, 1977, **27**, 265–272.

[6] D. Chang, C. D. Banack and S. L. Shah, *Journal of Magnetic Resonance*, 2007, **187**, 288–292.

[7] K. C. Wang, S. Y. Wang, C. H. Kuo and Y. J. Tseng, *Analytical Chemistry*, 2013, **85**, 1231–1239.

[8] C. Cobas, F. Seoane and S. Sýkora, *Poster at SMASH Conference*, 2008, 15706.

[9] T. S. Hughes, H. D. Wilson, I. M. S. de Vera and D. J. Kojetin, *PLOS ONE*, 2015, **10**, e0134474.

[10] G. Schwarz, *The Annals of Statistics*, 1978, **6**, 461–464.

[11] C. Cobas, F. Seoane, E. Vaz, M. A. Bernstein, S. Dominguez, M. Pérez and S. Sýkora, *Magnetic Resonance in Chemistry*, 2013, **51**, 649–654.

[12] T. Schoenberger, S. Menges, M. A. Bernstein, M. Pérez, F. Seoane, S. Sýkora and C. Cobas, *Analytical Chemistry*, 2016, **88**, 3836–3843.

[13] M. Newville, T. Stensitzki, D. B. Allen and A. Ingargiola, *LMFIT: Non-Linear Least-Square Minimization and Curve-Fitting for Python*, 2014, `https://zenodo.org/record/11813{#}.XQFn6W9KjyIhttps://doi.org/10.5281/zenodo.11813{#}.XJojF3zwy6w.mendeley{%}0Ahttps://zenodo.org/record/11813{#}.WySYWKdKiUk`.

[14] J.-M. Nuzillard, in *eMagRes*, John Wiley & Sons, Ltd, Chichester, UK, 2014, pp. 287–294.

[15] L. Griffiths, *The Analyst*, 1998, **123**, 1061–1068.

[16] H. W. Kuhn, in *50 Years of Integer Programming 1958-2008: From the Early Years to the State-of-the-Art*, John Wiley & Sons, Ltd, 2010, vol. 2, pp. 29–47.

[17] K. Ermanis, K. E. B. Parkes, T. Agback and J. M. Goodman, *Organic & Biomolecular Chemistry*, 2017, **15**, 8998–9007.

[18] G. K. Pierens, *Journal of Computational Chemistry*, 2014, **35**, 1388–1394.

[19] D. W. Scott, *Multivariate Density Estimation : Theory, Practice, and Visualization.*, Wiley, 2015, p. 381.

[20] J. R. Cabrera-Pardo, A. Trowbridge, M. Nappi, K. Ozaki and M. J. Gaunt, *Angewandte Chemie International Edition*, 2017, **56**, 11958–11962.

[21] K. F. Hogg, A. Trowbridge, A. Alvarez-Pérez and M. J. Gaunt, *Chemical Science*, 2017, **8**, 8198–8203.

[22] B. Y. Han, N. Y. Lam, C. I. MacGregor, J. M. Goodman and I. Paterson, *Chemical Communications*, 2018, **54**, 3247–3250.

[23] L. B. Marx and J. W. Burton, *Chemistry - A European Journal*, 2018, **24**, 6747–6754.

[24] S. Ainsua Martinez, M. Gillard, A. C. Chany and J. W. Burton, *Tetrahedron*, 2018, **74**, 5012–5021.

[25] N. Y. S. Lam, G. Muir, V. R. Challa, R. Britton and I. Paterson, *Chemical Communications*, 2019, **55**, 9717–9720.
